# Comparative effects of drug interventions for the acute management of migraine episodes in adults: systematic review and network meta-analysis

**DOI:** 10.1136/bmj-2024-080107

**Published:** 2024-09-18

**Authors:** William K Karlsson, Edoardo G Ostinelli, Zixuan A Zhuang, Lili Kokoti, Rune H Christensen, Haidar M Al-Khazali, Christina I Deligianni, Anneka Tomlinson, Håkan Ashina, Elena Ruiz de la Torre, Hans-Christoph Diener, Andrea Cipriani, Messoud Ashina

**Affiliations:** 1 Department of Neurology, Danish Headache Centre, Copenhagen University Hospital – Rigshospitalet, Copenhagen, Denmark; 2 Department of Clinical Medicine, Faculty of Health and Medical Sciences, University of Copenhagen, Copenhagen, Denmark; 3 Department of Psychiatry, University of Oxford, Oxford, UK; 4 Oxford Precision Psychiatry Lab, National Institute for Health Research (NIHR) Oxford Health Biomedical Research Centre, University of Oxford, Oxford, UK; 5 Warneford Hospital, Oxford Health NHS Foundation Trust, Oxford, UK; 6 Department of Neurology, Athens Naval Hospital, Athens, Greece; 7 1st Department of Neurology, Aeginition Hospital, National and Kapodistrian University of Athens, Athens, Greece; 8 European Migraine and Headache Alliance, Brussels, Belgium; 9 Department of Neuroepidemiology, Institute for Medical Informatics, Biometry and Epidemiology, University Duisburg-Essen, Essen, Germany; 10 Danish Knowledge Centre on Headache Disorders, Copenhagen University Hospital – Rigshospitalet, Copenhagen, Denmark

## Abstract

**Objective:**

To compare all licensed drug interventions as oral monotherapy for the acute treatment of migraine episodes in adults.

**Design:**

Systematic review and network meta-analysis.

**Data sources:**

Cochrane Central Register of Controlled Trials, Medline, Embase, ClinicalTrials.gov, EU Clinical Trials Register, WHO International Clinical Trials Registry Platform, as well as websites of regulatory agencies and pharmaceutical companies without language restrictions until 24 June 2023.

**Methods:**

Screening, data extraction, coding, and risk of bias assessment were performed independently and in duplicate. Random effects network meta-analyses were conducted for the primary analyses. The primary outcomes were the proportion of participants who were pain-free at two hours post-dose and the proportion of participants with sustained pain freedom from two to 24 hours post-dose, both without the use of rescue drugs. Certainty of the evidence was graded using the confidence in network meta-analysis (CINeMA) online tool. Vitruvian plots were used to summarise findings. An international panel of clinicians and people with lived experience of migraine co-designed the study and interpreted the findings.

**Eligibility criteria for selecting studies:**

Double blind randomised trials of adults (≥18 years) with a diagnosis of migraine according to the International Classification of Headache Disorders.

**Results:**

137 randomised controlled trials comprising 89 445 participants allocated to one of 17 active interventions or placebo were included. All active interventions showed superior efficacy compared with placebo for pain freedom at two hours (odds ratios from 1.73 (95% confidence interval (CI) 1.27 to 2.34) for naratriptan to 5.19 (4.25 to 6.33) for eletriptan), and most of them also for sustained pain freedom to 24 hours (odds ratios from 1.71 (1.07 to 2.74) for celecoxib to 7.58 (2.58 to 22.27) for ibuprofen). In head-to-head comparisons between active interventions, eletriptan was the most effective drug for pain freedom at two hours (odds ratios from 1.46 (1.18 to 1.81) to 3.01 (2.13 to 4.25)), followed by rizatriptan (1.59 (1.18 to 2.17) to 2.44 (1.75 to 3.45)), sumatriptan (1.35 (1.03 to 1.75) to 2.04 (1.49 to 2.86)), and zolmitriptan (1.47 (1.04 to 2.08) to 1.96 (1.39 to 2.86)). For sustained pain freedom, the most efficacious interventions were eletriptan and ibuprofen (odds ratios from 1.41 (1.02 to 1.93) to 4.82 (1.31 to 17.67)). Confidence in accordance with CINeMA ranged from high to very low. Sensitivity analyses on Food and Drug Administration licensed doses only, high versus low doses, risk of bias, and moderate to severe headache at baseline confirmed the main findings for both primary and secondary outcomes.

**Conclusions:**

Overall, eletriptan, rizatriptan, sumatriptan, and zolmitriptan had the best profiles and they were more efficacious than the recently marketed drugs lasmiditan, rimegepant, and ubrogepant. Although cost effectiveness analyses are warranted and careful consideration should be given to patients with a high risk cardiovascular profile, the most effective triptans should be considered as preferred acute treatment for migraine and included in the WHO List of Essential Medicines to promote global accessibility and uniform standards of care.

**Systematic review registration:**

Open Science Framework https://osf.io/kq3ys/.

**Figure:**
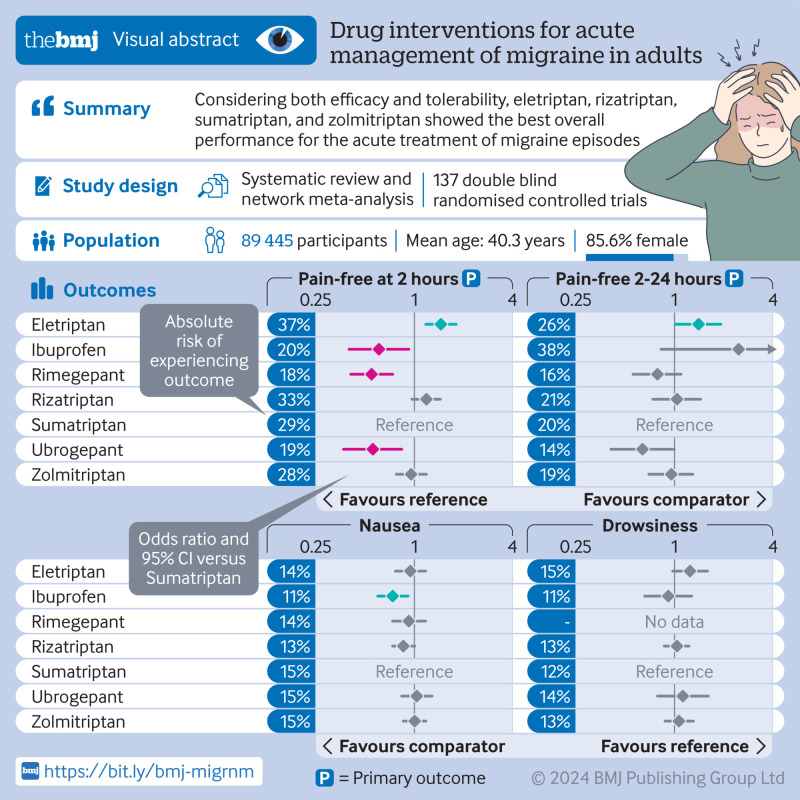


## Introduction

Migraine is a neurological disorder characterised by disabling, recurrent episodes of moderate to severe headache and accompanying symptoms lasting up to 72 hours.^1^ Migraine affects more than one billion people worldwide and is the leading cause of disability in girls and women aged 15 to 49 years.^2^ The burden of migraine extends to personal welfare, reduced productivity, and poor socioeconomic outcomes.^3^

The acute management of migraine episodes consists of drug interventions aimed at providing rapid and sustained pain relief, and, ideally, freedom from pain.^4^ Several drugs with different mechanisms of action are available.^1^ International clinical guidelines generally endorse non-steroidal anti-inflammatory drugs (NSAIDs) as initial treatment, whereas triptans are recommended for moderate to severe episodes or when the response to NSAIDs is insufficient.^5^ ^6^ ^7^ ^8^ In recent years, lasmiditan and gepants have been introduced as further treatment options,^1^ especially for patients with contraindications to triptans owing to potential vasoconstrictive effects and higher risk of ischaemic events.^9^ ^10^ However, no clear consensus exists as to which specific agents from these drug classes should be selected initially.

Given the wide range of drugs for acute treatment of migraine, clinicians and patients need robust evidence to make the best, individualised choice in routine practice. Network meta-analyses allow for estimation of comparative efficacy, providing a comprehensive summary of the evidence base and understanding of the relative merits of the multiple interventions.^11^ Previous network meta-analyses, however, only compared a subset of available drugs.^12^ ^13^ ^14^ ^15^ ^16^ ^17^ ^18^ ^19^ ^20^ ^21^ As part of the AMADEUS (acute migraine attacks: different effects of individual drugs) “project,” we conducted a systematic review and network meta-analysis to compare licensed oral drugs for the acute treatment of migraine episodes in adults.

## Methods

### Information sources and eligibility criteria

Full details about the methods are reported in the protocol (see supplementary appendix 1), which has been registered in Open Science Framework (https://osf.io/kq3ys/). Our reporting of the study adhered to the guidelines outlined in the PRISMA (preferred reporting items for systematic reviews and meta-analyses) statement for systematic reviews incorporating network meta-analyses.^22^

We searched for published and unpublished studies in the Cochrane Central Register of Controlled Trials, Medline, Embase, ClinicalTrials.gov, EU Clinical Trials Register, WHO (World Health Organization) International Clinical Trials Registry Platform, as well as websites of regulatory agencies and pharmaceutical companies without language restrictions until 24 June 2023 (see supplementary appendix 2 for full search strategy).

We included double blind, randomised controlled trials comparing monotherapy using oral drugs with placebo or another eligible active treatment for the acute treatment of migraine episodes in adults (≥18 years). Participants were outpatients with a diagnosis of migraine according to the International Classification of Headache Disorders.^23^ ^24^ ^25^ ^26^ Only drugs and treatment dose ranges licensed for migraine or headache were considered eligible if they were recommended by at least one of the regulatory bodies internationally (also see supplementary appendix 3 and table S1): the British National Formulary (UK), the Federal Institute for Drugs and Medical Devices (Germany), the European Medicines Agency, the National Agency for the Safety of Medicines and Health Products (France), the Pharmaceuticals and Medical Devices Agency (Japan), the Therapeutic Goods Administration (Australia), and the US Food and Drug Administration (FDA). We did not include opiates as clinical guidelines discourage their use for migraine owing to limited efficacy, considerable adverse effects, and risk of dependency.^4^ ^6^ We excluded studies set in emergency departments as people attending these due to migraine usually represent a subgroup with particularly severe or atypical episodes.^27^

Pairs of researchers independently screened and selected the studies, reviewed published and unpublished reports, extracted data from the included trials, and assessed risk of bias.^28^ Any discrepancies were resolved by discussion with the other members of the team.

### Outcomes

We selected outcomes recommended by the International Headache Society.^29^ The primary outcomes were the proportion of participants who were pain-free at two hours post-dose and the proportion of participants with sustained pain freedom from two to 24 hours post-dose, both without the use of rescue drugs.

Secondary outcomes included the proportion of participants with pain relief at two hours post-dose, the proportion with pain relapse within two to 48 hours post-dose, and the proportion using rescue drugs after two hours and up to 24 hours. We also investigated safety and tolerability, assessing the proportion of participants who experienced at least one serious adverse event and the proportion with at least one of 19 specific clinically relevant adverse events predefined in the protocol (see supplementary appendix 1).

### Summary measures and synthesis

The intention-to-treat principle was applied by using the number of patients randomised as the denominator in all analyses and assuming that patients with missing information had a negative outcome. We evaluated the assumption of transitivity (ie, that valid indirect comparisons could be made through the network because the distribution of effect modifiers on average was similar between the compared sets of trials)^30^ by comparing the distribution of the several potential effect modifiers across comparisons for our primary outcomes: mean age,^31^ sex (ie, the proportion of female participants),^32^ headache intensity at baseline (ie, the proportion of participants with moderate or severe pain),^33^ and ongoing use of preventive migraine drugs.^34^ Global and local approaches were used to assess the inconsistency between direct and indirect sources of evidence.^35^ To assess the inconsistency globally, we used a design-by-treatment test,^36^ whereas for local inconsistency we used back calculation and separated indirect from direct design evidence methods to compare direct and indirect evidence for each pairwise treatment comparison.^37^ Statistical heterogeneity was assessed for each pairwise and network meta-analysis comparison using τ^2^ and I^2^ statistics.^11^

We conducted a series of network meta-analyses using a random effects model within a frequentist setting, assuming equal heterogeneity across all comparisons and accounting for correlations induced by multi-arm studies. For studies with rare events (ie, an event rate of <5%), we used a common effect Maentel-Haenszel approach.^37^ We conducted the network meta-analyses using the “netmeta” package in R (version 4.2.2). We estimated effect sizes from pairwise and network meta-analyses by summary odds ratios for dichotomous outcomes with corresponding 95% confidence intervals (CIs).

League tables and vitruvian plots were used to present the findings from the network meta-analyses.^37^ The vitruvian plot is a benefit-harm communication tool to summarise direction, magnitude, and uncertainty of effects over multiple outcomes in network meta-analysis.^38^ For the vitruvian plots, we selected sumatriptan as the reference intervention as it is the most commonly prescribed migraine specific drug and it is included in the WHO Model List of Essential Medicines.^39^ As secondary analyses, we also visualised results using forest plots and vitruvian plots with placebo or ibuprofen as reference treatments.

The risk of bias of individual studies was assessed on each primary outcome with the Cochrane risk of bias tool, version 2.0 (RoB2),^28^ and the certainty of evidence was assessed using the confidence in network meta-analysis (CINeMA) framework.^40^

### Additional analyses

We evaluated possible heterogeneity of treatment effects using bayesian network meta-regressions for sex assigned at birth and presence of aura. To evaluate the robustness of our findings, we carried out the following sensitivity analyses on our primary outcomes: trials only with doses licensed by the FDA, with low risk of bias, with participants experiencing moderate or severe headache, with a diagnosis of menstrual related migraine, splitting nodes with high and low doses, assessed the effect of placebo response, excluding studies with participants with medical comorbidity, or excluding studies that allowed the use of preventive drugs.

### Patient and public involvement

We discussed the aims and design of this study with members of the public, including those who had experienced migraine (one patient representative is a coauthor of this paper and has been involved in all stages of the project). We used their feedback to guide the selection of outcomes for the study and inform the interpretation of the results presented in this manuscript. Three members of the research team conducted statistical analyses and presented the results in a blinded fashion (ie, the names of the interventions were masked to reduce bias from previous experience or knowledge) to two independent panels of expert clinicians and patient representatives from international organisations in Argentina, Canada, Europe, and the US.

## Results

### Study selection and network geometry

**Fig 1:**
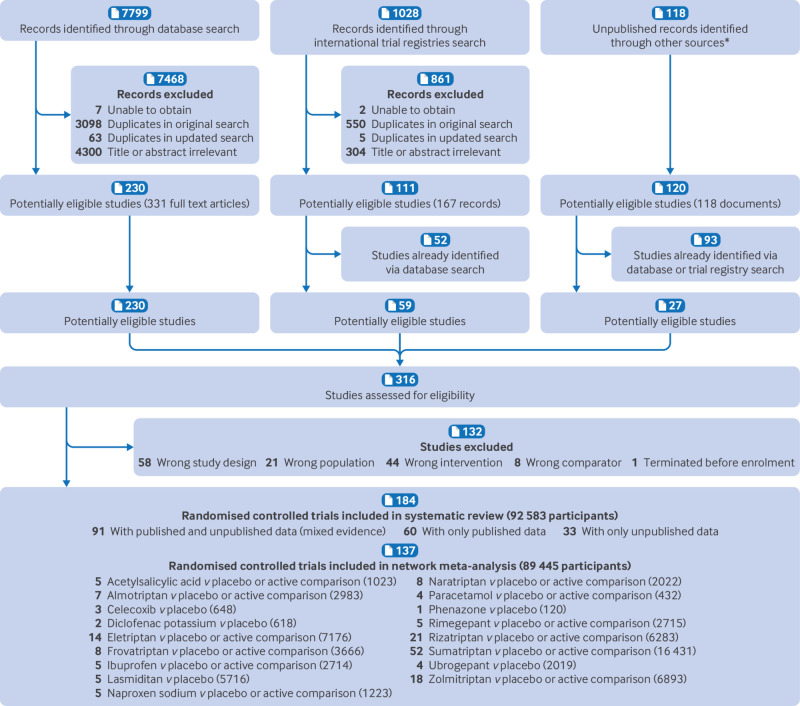
Study selection process. *See supplementary appendix table S2 for full list

Overall, 184 double blind randomised controlled trials published between 1991 and 2023 were identified (fig 1). Supplementary appendix 4 and tables S3 and S4 describe the included studies. Of those studies, 174 (95%) were sponsored by the pharmaceutical industry, 163 (89%) were placebo controlled, and 52 (28%) directly compared at least two eligible active interventions. Seventy six trials were from North America (41%), 47 from Europe (26%), 16 from Asia (9%), and 37 recruited participants from more than one continent (20%). We retrieved unpublished information for 124 (67%) trials. The median study sample size was 378 (interquartile range 132-690) participants, mean age 40.3 (standard deviation 10.9) years, 85.6% of the total sample were female participants, and 32.3% had a history of migraine with aura.

**Fig 2:**
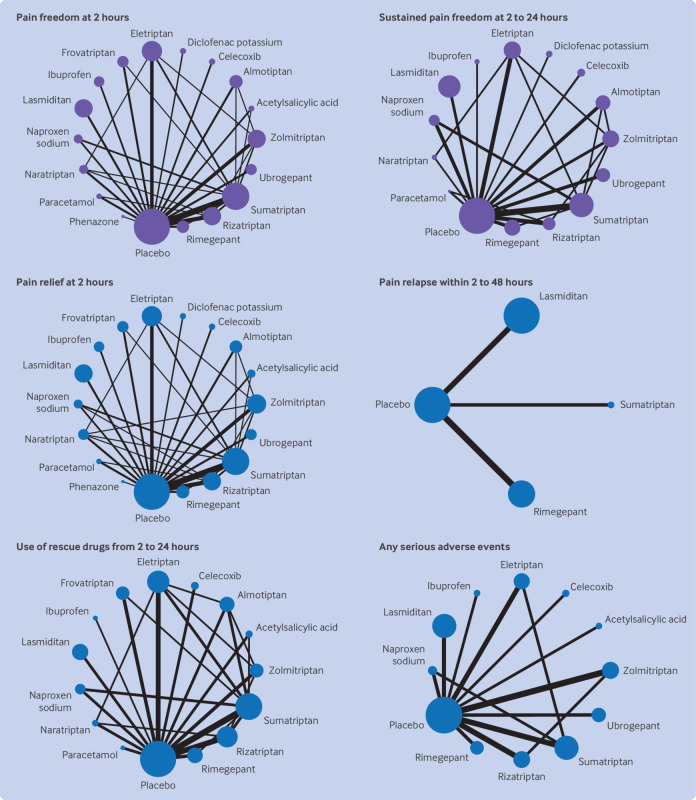
Network plots of eligible direct comparisons for primary and secondary efficacy outcomes and any serious adverse events. Line width is proportional to the number of trials comparing each pair of treatments. Node size is proportional to the number of randomised participants

**Fig 3:**
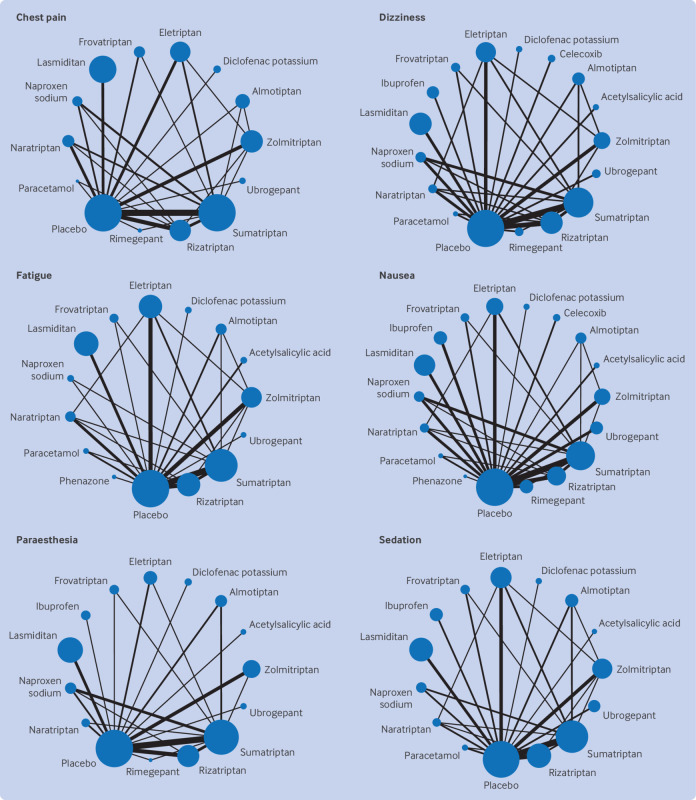
Network plots of eligible direct comparisons for six specific non-serious adverse events considered most important by clinician and patient representative panels. Line width is proportional to the number of trials comparing each pair of treatments. Node size is proportional to the number of randomised participants. See supplementary appendix 5 for network plots of the remaining specific adverse events

Overall, 137 randomised controlled trials were included in the network meta-analyses, with 62 682 participants allocated to drug treatment and 26 763 to placebo. The 17 individual drugs were divided into five categories: antipyretics (paracetamol), ditans (lasmiditan), gepants (rimegepant and ubrogepant), NSAIDs (acetylsalicylic acid, celecoxib, diclofenac potassium, ibuprofen, naproxen sodium, and phenazone), and triptans (almotriptan, eletriptan, frovatriptan, naratriptan, rizatriptan, sumatriptan, and zolmitriptan). All interventions had at least one placebo controlled trial for one or more outcomes (fig 2 and fig 3) and most networks were well connected (see supplementary appendix 5). The full dataset and information for the vitruvian plots are freely available online at GitHub (https://github.com/EGOstinelli/NMA-on-migraine/).

### Synthesis of results and certainty of evidence

**Fig 4:**
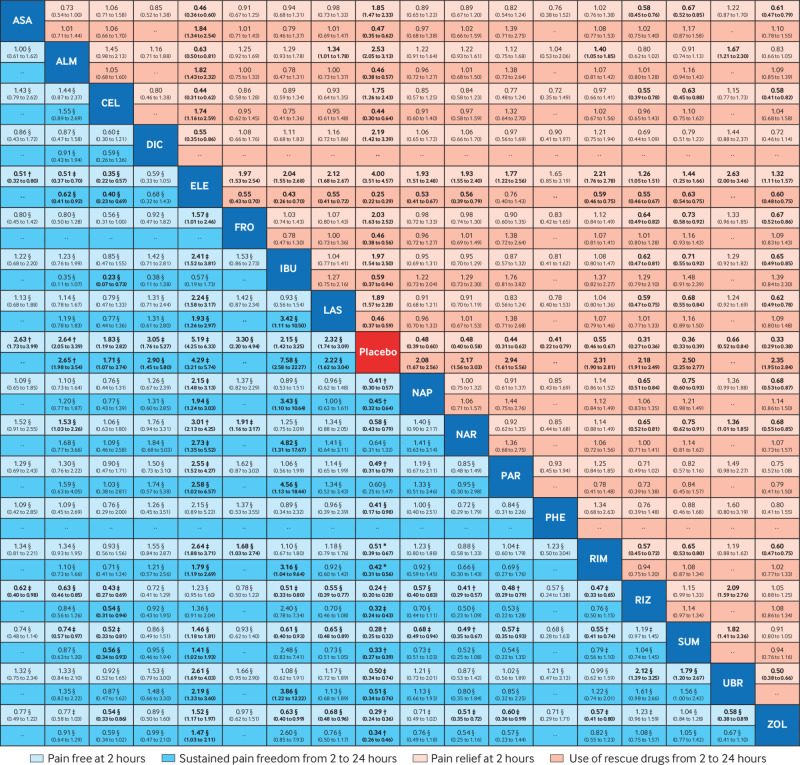
Network meta-analysis for efficacy of drugs (in alphabetical order) for the acute treatment of migraine (freedom from pain, sustained pain freedom, pain relief, and use of rescue drugs). Comparisons should be read from left to right. Comparative estimates (reported as odds ratios with corresponding 95% confidence intervals) are located at the intersection between the treatment defined by the column and the treatment defined by the row. Bottom left rectangle: For pain freedom and sustained pain freedom from two to 24 hours, estimates >1 favour the treatment defined by the column. Top right rectangle: for pain relief, estimates >1 favour the treatment defined by the row. For use of rescue drugs, estimates <1 favour the treatment defined by the row. Emboldened numbers represent estimates where the confidence interval is either >1 or <1. Certainty of the evidence (according to confidence in network meta-analysis (CINeMA)) for the two primary outcomes is presented: *=high certainty of evidence; †=moderate certainty of evidence; ‡=low certainty of evidence; §=very low certainty of evidence. ASA=acetylsalicylic acid; ALM=almotriptan; CEL=celecoxib; DIC=diclofenac potassium; ELE=eletriptan; FRO=frovatriptan; IBU=ibuprofen; LAS=lasmiditan; NAP=naproxen sodium; NAR=naratriptan; PAR=paracetamol; PHE=phenazone; RIM=rimegepant; RIZ=rizatriptan; SUM=sumatriptan; UBR=ubrogepant; ZOL=zolmitriptan

**Fig 5:**
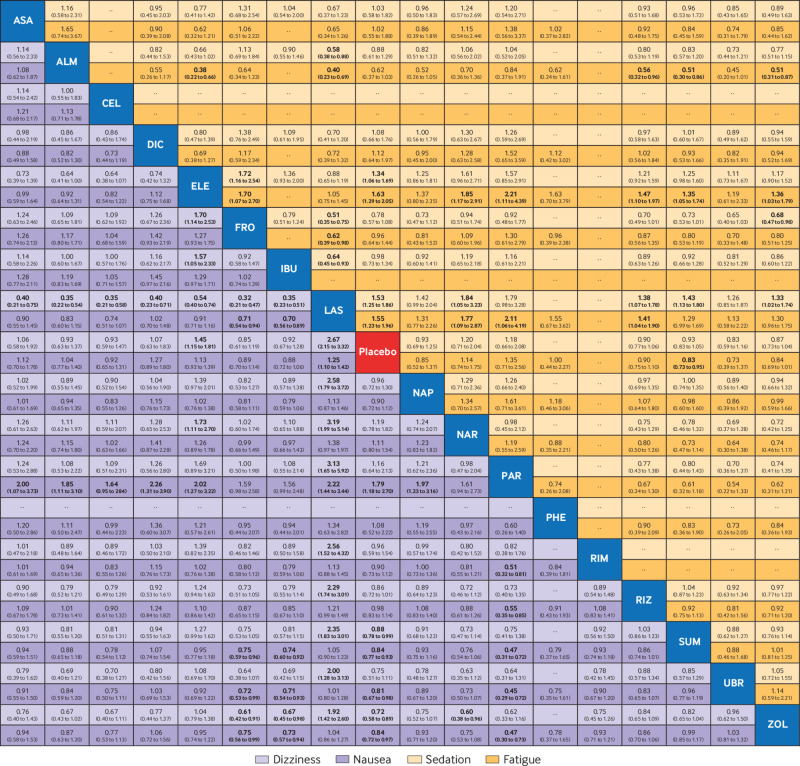
Network meta-analysis for adverse events (dizziness, fatigue, nausea, and sedation) associated with drugs (in alphabetical order) for the acute treatment of migraine. Comparisons should be read from left to right. Comparative estimates are located at the intersection between the treatment defined by the column and the treatment defined by the row. Data are presented as odds ratio with corresponding 95% confidence intervals. Bottom left rectangle: for dizziness and nausea, estimates <1 favour the treatment defined by the column. Top right rectangle: for sedation and fatigue, estimates <1 favour the treatment defined by the row. Emboldened numbers represent estimates where the confidence interval of the comparative estimate is either >1 or <1. ASA=acetylsalicylic acid; ALM=almotriptan; CEL=celecoxib; DIC=diclofenac potassium; ELE=eletriptan; FRO=frovatriptan; IBU=ibuprofen; LAS=lasmiditan; NAP=naproxen sodium; NAR=naratriptan; PAR=paracetamol; PHE=phenazone; RIM=rimegepant; RIZ=rizatriptan; SUM=sumatriptan; UBR=ubrogepant; ZOL=zolmitriptan

Figure 4 and figure 5 show the results of the network meta-analyses. Further results are available in supplementary appendices 6-9. All active interventions were more efficacious than placebo for pain freedom at two hours (odds ratios from 1.73 (95% CI 1.27 to 2.34) for naratriptan to 5.19 (4.25 to 6.33) for eletriptan) and most were also efficacious for sustained pain freedom from two to 24 hours post-dose, except paracetamol and naratriptan (odds ratio 1.66 (0.68 to 4.04) and 1.57 (0.76 to 3.25), respectively). When the active interventions were compared with each other, eletriptan was superior to almost all the other drugs for achieving pain freedom at two hours, followed by rizatriptan, sumatriptan, and zolmitriptan (odds ratios from 1.35 to 3.01). For sustained pain freedom up to 24 hours, the most efficacious interventions were eletriptan (odds ratios from 1.41 to 2.73) and ibuprofen (odds ratios from 3.16 to 4.82). In terms of secondary efficacy outcomes, all interventions were superior to placebo for pain relief at two hours and for use of rescue drugs from two to 24 hours.

When the drugs were compared head to head, eletriptan was associated with better efficacy than nearly all of the other active interventions for pain relief at two hours (odds ratios from 1.26 to 2.63) and use of rescue drugs (odds ratios from 0.43 to 0.63). Outcome data on pain relapse up to 48 hours were only available for lasmiditan, sumatriptan, and rimegepant: all showed greater efficacy than placebo, with comparable performances for lasmiditan (odds ratio 0.42 (95% CI 0.12 to 1.48)) and rimegepant (0.29 (0.08 to 1.03)) relative to sumatriptan. For adverse events, dizziness was more commonly associated with lasmiditan, eletriptan, sumatriptan, and zolmitriptan (odds ratios from 1.14 to 3.19). Fatigue and sedation occurred more frequently with eletriptan (odds ratios from 1.34 to 2.63) and lasmiditan (odds ratios from 1.33 to 2.50). Paraesthesia was more often associated with lasmiditan (odds ratios from 1.28 to 1.50), sumatriptan (odds ratio versus placebo 1.18 (95% CI 1.04 to 1.32)), and zolmitriptan (odds ratios from 1.18 to 1.50). Nausea was also more likely to be experienced with lasmiditan, sumatriptan, zolmitriptan, and ubrogepant (odds ratios from 1.19 to 2.22). Paracetamol was, conversely, less likely to be associated with nausea (odds ratios from 0.44 to 0.56) but more likely to be associated with hepatic toxicity (odds ratios from 6.40 to 7.69). Eletriptan was the only intervention more frequently associated with chest pain or discomfort (odds ratios from 1.42 to 1.78).

**Fig 6:**
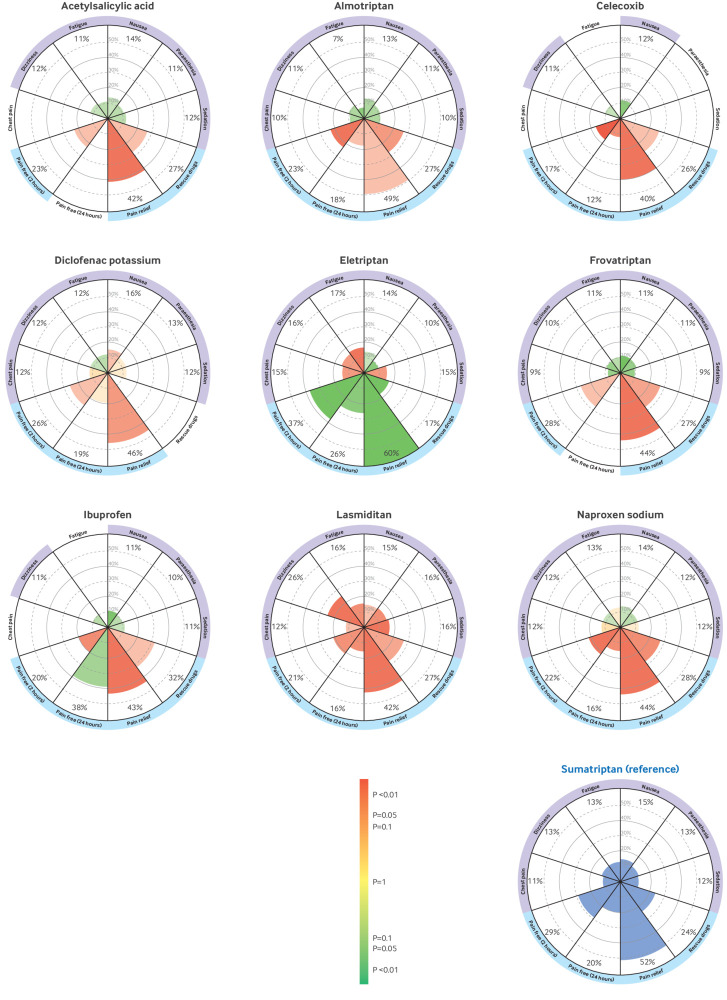
Vitruvian plots of each active intervention (in alphabetical order) compared with sumatriptan (reference drug) across key outcomes. Efficacy is reported in the bottom wedges by four outcomes: freedom from pain at two hours, sustained pain freedom from two to 24 hours, pain relief at two hours, and use of rescue drugs from two to 24 hours. Tolerability is reported in the lateral and top wedges by the specific adverse events of chest pain or discomfort, dizziness, fatigue, nausea, paraesthesia, and sedation. Colour indicates the relative performance of the intervention of interest and the precision of the estimate in comparison with sumatriptan (reference drug), from green (the intervention is better than sumatriptan), to yellow (unclear whether the drug performs better or worse than sumatriptan), and to red (the intervention is worse than sumatriptan). The more precise the estimate is, the more intense the colours. Estimated event rates are expressed as absolute percentages. The wedge titles are coloured to indicate availability of data for the analyses (if no data are available for the analyses, the wedge titles are white (ie, without any colour)). Supplementary appendix 10 provides further details, including vitruvian plots with ibuprofen or placebo as the reference intervention

**Fig 7:**
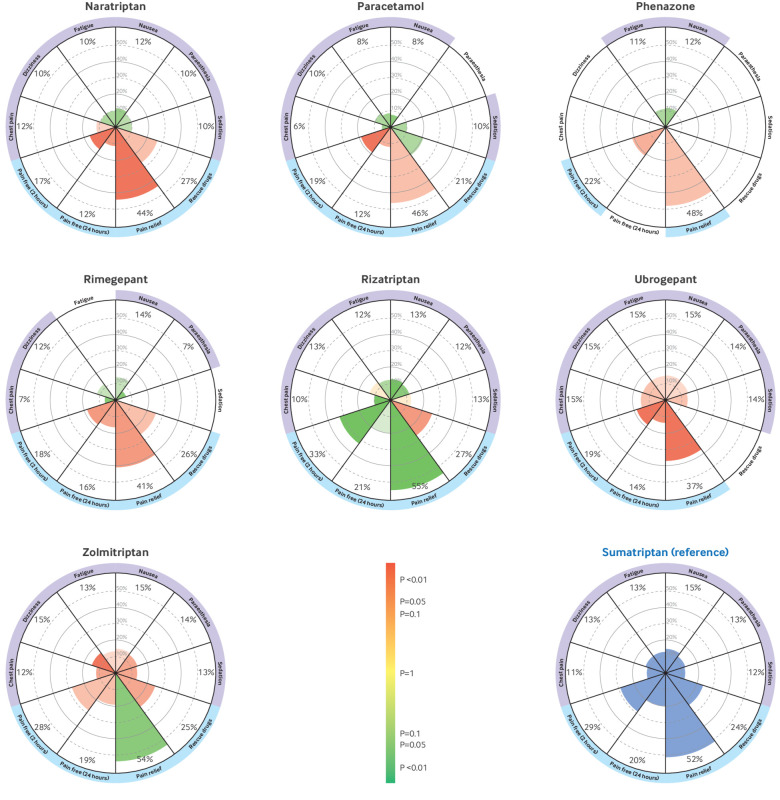
Continued: Vitruvian plots of each active intervention (in alphabetical order) compared with sumatriptan (reference drug) across key outcomes. Efficacy is reported in the bottom wedges by four outcomes: freedom from pain at two hours, sustained pain freedom from two to 24 hours, pain relief at two hours, and use of rescue drugs from two to 24 hours. Tolerability is reported in the lateral and top wedges by the specific adverse events of chest pain or discomfort, dizziness, fatigue, nausea, paraesthesia, and sedation. Colour indicates the relative performance of the intervention of interest and the precision of the estimate in comparison with sumatriptan (reference drug, blue), from green (the intervention is better than sumatriptan), to yellow (unclear whether the drug performs better or worse than sumatriptan), and to red (the intervention is worse than sumatriptan). The more precise the estimate is, the more intense the colours. Estimated event rates are expressed as absolute percentages. The wedge titles are coloured to indicate availability of data for the analyses (if no data are available for the analyses, the wedge titles are white (ie, without any colour)). Supplementary appendix 10 provides further details, including vitruvian plots with ibuprofen or placebo as the reference intervention

The vitruvian plots show the 10 outcomes deemed the most clinically relevant by the panel of expert clinicians and patient representatives (pain freedom at two hours, sustained pain freedom from two to 24 hours, pain relief at two hours, use of rescue drugs within two to 24 hours, chest pain or discomfort, dizziness, fatigue, nausea, paraesthesia, and sedation) using sumatriptan as the reference drug (fig 6 and fig 7). Supplementary appendix 10 shows the vitruvian plots using placebo and ibuprofen as reference interventions.

The certainty of the evidence for the primary outcomes assessed using CINeMA ranged from high to very low. Rimegepant versus placebo was the only comparison rated high certainty for each primary outcome. For pain freedom at two hours, 13 of 153 (8%) comparisons were rated moderate certainty, 26 (17%) were rated low certainty, and 113 (74%) were rated very low certainty. For sustained pain freedom until 24 hours, 4 of 105 (4%) comparisons were rated moderate, 5 (5%) were rated low, and 95 (90%) were rated very low. Supplementary appendix 11 and tables S5, and S6 provide full information about CINeMA. Risk of bias assessed using the Cochrane risk of bias 2 tool (RoB2) was rated low for pain freedom at two hours in 24 of 115 (21%) randomised controlled trials, some concerns in 73 (63%), and high in 18 (16%). For sustained pain freedom, risk of bias was rated low in 16 of 56 (29%) randomised controlled trials, some concerns in 34 (61%), and high in 6 (11%). See supplementary appendix 12 and tables S7 and S8 for further information on risk of bias.

### Credibility assessment and sensitivity analyses

Measures of statistical heterogeneity (τ^2^ and I^2^) and inconsistency for each outcome are shown in supplementary appendix 13 as well as for subgroup and sensitivity analyses in supplementary appendices 14 and 15. No violations of our transitivity assumptions were identified. Inconsistencies were observed among comparisons for the outcomes of pain freedom at two hours (8%), sustained pain freedom (5%), use of rescue drugs (9%), dizziness (8%), chest pain or discomfort (13%), and sedation (5%). We checked the data for potential extraction or entering errors, but no mistakes were identified.

We considered changes in the magnitude of the placebo response as a potential explanation of heterogeneity and inconsistency. To explore this, we did a meta-regression of the log proportion of placebo responders over time for each primary outcome, which showed a structural break corresponding to the year 1997 for pain freedom at two hours. A sensitivity analysis restricted to studies after 1997 resulted in comparable results. Overall, sensitivity analyses on FDA licensed doses only, high versus low doses, risk of bias, and moderate-to-severe headache at baseline confirmed our main findings (see supplementary appendix 15).

## Discussion

Compared with previous studies, our systematic review and network meta-analysis provided comprehensive data synthesis on the acute treatment of migraine in adults.^21^ ^41^ Our findings showed that some triptans—namely, eletriptan, rizatriptan, sumatriptan, and zolmitriptan—had the most favourable overall profiles in terms of efficacy and tolerability. These four triptans were more efficacious than the most recently marketed drugs lasmiditan, rimegepant, and ubrogepant, which, based on our results, showed efficacy comparable to that of paracetamol and most NSAIDs.

Triptans are selective serotonin (5 hydroxytryptamine)_1B_/_1D_ receptor agonists, exhibiting differences in receptor affinity, lipophilicity, metabolism, and pharmacokinetic profiles within the same class.^4^ Despite their low acquisition costs and balanced efficacy and tolerability profiles, however, triptans remain underused among people with migraine.^42^ ^43^ In the US, current use of triptans ranges from 16.8% to 22.7%,^43^ and in Europe from 3.4% to 22.5%.^42^ Triptans are contraindicated in patients with vascular disease, posing an important limitation to their use.^4^ However, concerns about their cardiovascular safety remain difficult to interpret, as cerebrovascular events may present primarily as migraine-like headaches, and misdiagnosis of transient ischaemic attack and minor stroke as migraine is not rare.^44^ ^45^ Moreover, studies assessing the response to high dose intravenous eletriptan or subcutaneous sumatriptan found no clinically significant vasoconstriction in patients undergoing diagnostic coronary angiography.^9^ Future studies revisiting the vascular contraindications of triptans are crucial to minimise potentially missed treatment opportunities.

The most recently marketed drugs, such as lasmiditan, rimegepant, and ubrogepant, are not associated with vasoconstrictive effects and have therefore been promoted as alternatives for patients for whom triptans are contraindicated or not tolerated.^4^ While rimegepant was well tolerated based on the results in our study, ubrogepant showed increased risk of nausea compared with placebo. Lasmiditan was associated with a substantial risk of dizziness, along with paraesthesia and sedation. Restrictions raised by the FDA against driving for eight hours after intake of lasmiditan underscore the challenges to its use.^1^ Moreover, the high costs of these new drugs pose a barrier to their widespread use and necessitate trials to ascertain their cost effectiveness for patients with insufficient response to triptans.^1^ Notably, our search identified one ongoing study, with pending results, in participants for whom triptans were unsuitable owing to lack of efficacy, previous intolerance, or contraindications.^46^

Our results showed wide variation in performance across individual NSAIDs. Diclofenac potassium showed efficacy and tolerability close to that of sumatriptan, but these estimates were imprecise due to the large confidence intervals. For ibuprofen, the high efficacy estimate for sustained pain freedom was driven by a single study with a noticeably low placebo response. Acetylsalicylic acid and naproxen sodium showed moderate efficacy, with tolerability comparable to that of sumatriptan. Celecoxib ranked lowest among NSAIDs, whereas sparse evidence was available for phenazone. Taken together, NSAIDs performed worse than triptans, were comparable to gepants, and were less likely to cause adverse events compared with lasmiditan. Paracetamol, although showing limited effect for pain freedom at two hours, proved to be well tolerated, affirming its role as a viable option for those seeking pain relief with low risk of adverse events.

### Strengths and limitations of this study, and future directions

Using the websites of regulatory agencies and international trial registries, and contacting study authors and pharmaceutical companies, we managed to incorporate a large amount of unpublished data in the analysis. Nowadays, online archives exist where trials are prospectively registered, which makes the study search more reliable; however, these registries only collect transparent information about the most recent studies, and we cannot rule out the possibility that some studies were missing or that the same studies were counted twice in our analyses. By making the dataset fully and freely available, we welcome any information that might help clarify mistakes in our meta-analysis.

Our findings have some limitations. Moderate heterogeneity was found for most outcomes and, according to our ratings in CINeMA, confidence in our findings was low or very low for most comparisons. Lower confidence levels were often due to the lack of prespecified analysis plans (within study bias), imprecision of treatment effects, or lack of information about randomisation and allocation concealment. Considering all this, the risk of bias for many studies may largely be a matter of reporting.^47^ To increase the methodological rigour of the contributing evidence, we included only double blind trials, which are similar in design, patient populations, and conduct.^48^ ^49^ Available networks were in general adequately connected, with placebo or sumatriptan being the most connected interventions and thus increasing the reliance on indirect evidence. We investigated the impact of study year on our primary efficacy outcomes and found no effect on the results of our network meta-analyses. The temporal trend of the placebo response in trials of acute treatments for migraine episodes warrants further investigations owing to its relevance for planning of sample size in future trials and for network meta-analysis. Although our results enhance the choice of drugs based on personal preferences in relation to efficacy and risk of adverse events, our findings were limited to average treatment effects due to the lack of individual patient data. Since monotherapy drugs are generally preferred for treatment, we did not include combination drugs. To avoid violation of transitivity, we restricted our focus to oral treatments, although the drugs can be administered by alternative routes.^4^ Finally, in the present study we did not consider type of oral formulation, consistency in response across migraine episodes, or cost effectiveness. We also did not cover important clinical issues that might inform treatment decision making in routine clinical practice (eg, drug overuse headache or potential withdrawal symptoms). Additionally, because of the paucity of information reported in the original studies, we were not able to quantify some outcomes, such as global functioning.

### Clinical and policy implications

Results on both benefits and harms should inform shared clinical decision making, considering the preferences of patients, caregivers, and healthcare professionals. Our findings should help inform future guidelines and updates to recommendations to ensure that patients receive optimal care. Overall, the results of our network meta-analysis suggest that the best performing triptans should be considered the treatment of choice for migraine episodes owing to their capacity for inducing rapid and sustained pain freedom, which is of key importance for people with migraine.^50^ While the recent introduction of lasmiditan, rimegepant, and ubrogepant has expanded options for the acute treatment of migraine, the high cost of these newer drugs, along with the substantial adverse effects of lasmiditan, suggest their use as third line options, after the less expensive, similarly efficacious, second line options such as ibuprofen, acetylsalicylic acid, diclofenac potassium, almotriptan, and frovatriptan have been considered. However, ranking of treatments in clinical guidelines extends beyond efficacy, tolerability, safety, and acquisition costs alone and must also consider cost effectiveness, of which analyses are warranted, and accessibility. The inclusion of the most effective triptans (available as generic drugs) into the WHO Model List of Essential Medicines should be considered to promote global accessibility and uniform standards of care (currently, sumatriptan is the only triptan included).^39^ Limited access to triptans and their substantial underutilisation represents missed opportunities to offer more effective treatments and deliver better quality of care to people who experience migraine.^3^

### Conclusions

The results of this systematic review and network meta-analysis offer the best available evidence to guide the choice of acute oral drug interventions for migraine episodes. Our results are in line with recent observational evidence.^51^ Careful comparisons between randomised controlled trials and observational evidence represent a productive line of research, as they may complement one another, and both can inform clinical decision making.^52^ Nevertheless, we believe that, making the best use of the available, if limited, randomised evidence, our results and tools are valid and should be used to guide treatment choices, promoting shared, informed decision making between patients and clinicians.

All the statements comparing the performance of one drug with another should be tempered by the potential limitations of the current analyses, the quality of the available evidence, the characteristics of the study population, and the long term management of migraine.^53^ Future network meta-analyses using individual patient data are required to improve personalised guidance for managing acute treatment of migraine episodes.

**What is already known on this topic**

Migraine is a highly prevalent condition and among the leading causes of disability worldwide
Numerous oral drugs with different mechanisms of action are available for the acute management of migraine, but no clear consensus exists among clinical guidelines about the ranking of these treatments
Previous systematic reviews and network meta-analyses have only included a subset of currently licensed drugs

**What this study adds**

Considering both efficacy and tolerability, eletriptan, rizatriptan, sumatriptan, and zolmitriptan showed the best overall performance for the acute treatment of migraine
Eletriptan, rizatriptan, sumatriptan, and zolmitriptan were more efficacious than the recently marketed and more expensive drugs lasmiditan, rimegepant, and ubrogepant, which showed efficacy comparable to paracetamol and most non-steroidal anti-inflammatory drugs
Triptans are currently widely underused, and access to the most effective triptans should be promoted globally and international guidelines updated accordingly

## Data Availability

The full dataset and information for the vitruvian plots are freely available online at GitHub (https://github.com/EGOstinelli/NMA-on-migraine/).

## Web extra

Extra material supplied by authors

Supplementary information: Appendices 1-15, tables S1-S8, and references

## Notes

Contributors: WKK, EGO, HA, HCD, AC, and MA conceived and designed the study. WKK, EGO, HA, ERT, HCD, AC, and MA contributed to the methods of the study. WKK, ZAZ, LK, RHC, HMA, and CID selected the articles and extracted the data. WKK, EGO, and AC analysed the data. EGO, AT, and AC accessed and verified the data. WKK, EGO, HA, HCD, AC, and MA wrote the first draft of the manuscript. All authors interpreted the data and contributed to the writing of the final version of the manuscript. All authors agreed with the results and conclusions of this manuscript and had full access to all the data. WKK and EGO are joint first authors. AC and MA are joint last authors and are responsible for the decision to submit for publication. They are the guarantors. The corresponding author attests that all listed authors meet authorship criteria and that no others meeting the criteria have been omitted.

Transparency: The lead authors (AC and MA) affirm that the manuscript is an honest, accurate, and transparent account of the study being reported; that no important aspects of the study have been omitted; and that any discrepancies from the study as planned (and, if relevant, registered) have been explained.

Dissemination to participants and related patient and public communities: We plan to disseminate the results to relevant patient communities through the media relations department of our institutions. We also plan to use social media, as well as the websites of our organisations and societies involved in research and treatment of headache and migraine.

Provenance and peer review: Not commissioned; externally peer reviewed.
