# Supplementary material for: Comparative effects of drug interventions for the acute management of migraine episodes in adults: systematic review and network meta-analysis
Source: BMJ. 2024 Sep 18;386:e080107. doi: 10.1136/bmj-2024-080107 (PMC11409395; doi:10.1136/bmj-2024-080107)
Supplement: Supplementary file 1 — Supplementary information: Appendices 1-15, tables S1-S8, and references [file karw080107.ww.pdf]

COMPARATIVE EFFECTS OF PHARMACOLOGICAL INTERVENTIONS FOR THE ACUTE MANAGEMENT OF MIGRAINE ATTACKS IN ADULTS: A SYSTEMATIC REVIEW AND NETWORK META-ANALYSIS

William K Karlsson, Edoardo G Ostinelli, Zixuan A Zhuang, Lili Kokoti, Rune H Christensen, Haidar M Al-Khazali, Christina I Deligianni, Anneka Tomlinson, Håkan Ashina, Elena R de la Torre, Hans-Christoph Diener, Andrea Cipriani, Messoud Ashina

Supplementary Appendix

Table of Contents

APPENDIX 1. STUDY PROTOCOL **8**

1.1. Changes to the study protocol 8
1.2. Study protocol 8

APPENDIX 2. SEARCH STRATEGY **17**

2.1 Cochrane Central Register of Controlled Trials (CENTRAL) (via CRSO) 17
2.2 Embase (via Ovid) 17
2.3 Medline (via PubMed) 17
2.4 International Trial Registries 18

2.4.1 ClinicalTrials.Gov 18
2.4.2 European Union Clinical Trials Registry (EUCTR) 18
2.4.3 World Health Organization (WHO) International Clinical Trials Registry Platform 18

2.5 Websites of Regulatory Agencies 18
2.6 Websites of Pharmaceutical Companies 19

APPENDIX 3. ELIGIBLE INTERVENTIONS **20**

APPENDIX 4. STUDIES INCLUDED IN THE SYSTEMATIC REVIEW **21**

APPENDIX 5. NETWORK PLOTS **55**

5.01 Efficacy – Pain freedom at 2 hours 55
5.02 Efficacy – Sustained pain freedom from 2 to 24 hours 55
5.03 Efficacy – Pain relief at 2 hours 55
5.04 Efficacy – Pain relapse within to 2 to 48 hours 56
5.05 Efficacy – Use of rescue medication within 2 to 24 hours 56
5.06 Serious adverse events 56
5.07 Adverse events – Abdominal pain 56
5.08 Adverse events – Allergic reaction 57
5.09 Adverse events – Chest pain/discomfort 57
5.10 Adverse events – Constipation 57
5.11 Adverse events – Diarrhoea 57
5.12 Adverse events – Dizziness 58
5.13 Adverse events – Dry mouth 58
5.14 Adverse events – Dyspepsia 58
5.15 Adverse events – Fatigue 58
5.16 Adverse events – Gastrointestinal bleeding 59
5.17 Adverse events – Hepatic toxicity 59
5.18 Adverse events – Major adverse cardiovascular events 59
5.19 Adverse events – Nausea 59
5.20 Adverse events – Paraesthesia 59
5.21 Adverse events – Pruritus 60
5.22 Adverse events – Sedation 60
5.23 Adverse events – Serotonergic syndrome 60
5.24 Adverse events – Vertigo 60
5.25 Adverse events – Vomiting 61

APPENDIX 6. PAIRWISE META-ANALYSES **62**

6.01 Efficacy – Pain freedom at 2 hours 62

6.02 Efficacy – Sustained pain freedom from 2 to 24 hour ... 62
6.03 Efficacy – Pain relief at 2 hours ... 62
6.04 Efficacy – Pain relapse within to 2 to 48 hours ... 62
6.05 Efficacy – Use of rescue medication within 2 to 24 hours ... 62
6.06 Serious adverse events ... 62
6.07 Adverse events – Abdominal pain ... 62
6.08 Adverse events – Allergic reaction ... 62
6.09 Adverse events – Chest pain/discomfort ... 62
6.10 Adverse events – Constipation ... 62
6.11 Adverse events – Diarrhoea ... 63
6.12 Adverse events – Dizziness ... 63
6.13 Adverse events – Dry mouth ... 63
6.14 Adverse events – Dyspepsia ... 63
6.15 Adverse events – Fatigue ... 63
6.16 Adverse events – Gastrointestinal bleeding ... 63
6.17 Adverse events – Hepatic toxicity ... 63
6.18 Adverse events – Major adverse cardiovascular events ... 63
6.19 Adverse events – Nausea ... 63
6.20 Adverse events – Paraesthesia ... 63
6.21 Adverse events – Pruritus ... 63
6.22 Adverse events – Sedation ... 64
6.23 Adverse events – Serotonergic syndrome ... 64
6.24 Adverse events – Vertigo ... 64
6.25 Adverse events – Vomiting ... 64

APPENDIX 7. FOREST PLOTS (BY INDIVIDUAL DRUGS AND DRUG CLASS) ... 65

7.01 Efficacy – Pain freedom at 2 hours ... 65
7.02 Efficacy – Sustained pain freedom from 2 to 24 hours ... 65
7.03 Efficacy – Pain relief at 2 hours ... 65
7.04 Efficacy – Pain relapse within 2 to 48 hours ... 66
7.05 Efficacy – Use of rescue medication within 2 to 24 hours ... 66
7.06 Serious adverse events ... 66
7.07 Adverse events – Abdominal pain ... 66
7.08 Adverse events – Allergic reaction ... 67
7.09 Adverse events – Chest pain/discomfort ... 67
7.10 Adverse events – Constipation ... 67
7.11 Adverse events – Diarrhoea ... 68
7.12 Adverse events – Dizziness ... 68
7.13 Adverse events – Dry mouth ... 68
7.14 Adverse events – Dyspepsia ... 69
7.15 Adverse events – Fatigue ... 69
7.16 Adverse events – Gastrointestinal bleeding ... 69
7.17 Adverse events – Hepatic toxicity ... 69
7.18 Adverse events – Major adverse cardiovascular events ... 70
7.19 Adverse events – Nausea ... 70
7.20 Adverse events – Paraesthesia ... 70
7.21 Adverse events – Pruritus ... 70
7.22 Adverse events – Sedation ... 71
7.23 Adverse events – Serotonergic syndrome ... 71
7.24 Adverse events – Vertigo ... 71
7.25 Adverse events – Vomiting ... 71

APPENDIX 8. LEAGUE TABLES **72**
8.01 Efficacy – Pain freedom at 2 hours 73
8.02 Efficacy – Sustained pain freedom from 2 to 24 hours 75
8.03 Efficacy – Pain relief at 2 hours 77
8.04 Efficacy – Pain relapse within to 2 to 48 hours 79
8.05 Efficacy – Use of rescue medication within 2 to 24 hours 80
8.06 Serious adverse events 82
8.07 Adverse events – Abdominal pain 83
8.08 Adverse events – Allergic reaction 85
8.09 Adverse events – Chest pain/discomfort 86
8.10 Adverse events - Constipation 88
8.11 Adverse events – Diarrhoea 89
8.12 Adverse events – Dizziness 91
8.13 Adverse events – Dry mouth 93
8.14 Adverse events – Dyspepsia 95
8.15 Adverse events – Fatigue 97
8.16 Adverse events – Gastrointestinal bleeding 99
8.17 Adverse events – Hepatic toxicity 100
8.18 Adverse events – Major adverse cardiovascular events 102
8.19 Adverse events – Nausea 103
8.20 Adverse events – Paraesthesia 105
8.21 Adverse events – Pruritus 107
8.22 Adverse events – Serotonergic syndrome 109
8.23 Adverse events – Sedation 110
8.24 Adverse events – Vertigo 112
8.25 Adverse events – Vomiting 114

APPENDIX 9. CUMULATIVE P-SCORE RANKING **116**
9.01 Efficacy – Pain freedom at 2 hours 116
9.02 Efficacy – Sustained pain freedom from 2 to 24 hours 117
9.03 Efficacy – Pain relief at 2 hours 118
9.04 Efficacy – Pain relapse within to 2 to 48 hours 119
9.05 Efficacy – Use of rescue medication within 2 to 24 hours 120
9.06 Serious adverse events 121
9.07 Adverse events – Abdominal pain 122
9.08 Adverse events – Allergic reaction 123
9.09 Adverse events – Chest pain/discomfort 124
9.10 Adverse events – Constipation 125
9.11 Adverse events – Diarrhoea 126
9.12 Adverse events – Dizziness 127
9.13 Adverse events – Dry mouth 128
9.14 Adverse events – Dyspepsia 129
9.15 Adverse events – Fatigue 130
9.16 Adverse events – Gastrointestinal bleeding 131
9.17 Adverse events – Hepatic toxicity 132
9.18 Adverse events – Major cardiovascular adverse events 133
9.19 Adverse events – Nausea 134
9.20 Adverse events – Paraesthesia 135
9.21 Adverse events – Pruritus 136
9.22 Adverse events – Sedation 137

9.23 Adverse events – Serotonergic syndrome 138
9.24 Adverse events – Vertigo 139
9.25 Adverse events – Vomiting 140

APPENDIX 10. VITRUVIAN PLOTS 141

10.01 Vitruvian plots – Acetylsalicylic acid 141
10.02 Vitruvian plots – Almotriptan 141
10.03 Vitruvian plots – Celecoxib 141
10.04 Vitruvian plots – Diclofenac potassium 141
10.05 Vitruvian plots – Eletriptan 142
10.06 Vitruvian plots – Frovatriptan 142
10.07 Vitruvian plots – Ibuprofen 142
10.08 Vitruvian plots – Lasmiditan 142
10.09 Vitruvian plots – Naproxen sodium 142
10.10 Vitruvian plots – Naratriptan 142
10.11 Vitruvian plots – Paracetamol 142
10.12 Vitruvian plots – Phenazone 142
10.13 Vitruvian plots – Placebo 143
10.14 Vitruvian plots – Rimegepant 143
10.15 Vitruvian plots – Rizatriptan 143
10.16 Vitruvian plots – Sumatriptan 143
10.17 Vitruvian plots – Ubrogepant 143
10.18 Vitruvian plots – Zolmitriptan 143

APPENDIX 11. CINEMA (CERTAINTY OF EVIDENCE) 144

APPENDIX 12. RISK OF BIAS CHARTS 146

12.1 Pain freedom at 2 hours 146
12.2 Sustained pain freedom from 2 to 24 hours 146

APPENDIX 13. EVALUATION OF INCONSISTENCY; MAIN ANALYSIS 147

13.01 Efficacy – Pain freedom at 2 hours 147
13.02 Efficacy – Sustained pain freedom from 2 to 24 hours 147
13.03 Efficacy – Pain relief at 2 hours 147
13.04 Efficacy – Pain relapse within to 2 to 48 hours 147
13.05 Efficacy – Use of rescue medication within 2 to 24 hours 147
13.06 Serious adverse events 147
13.07 Adverse events – Abdominal pain 147
13.08 Adverse events – Allergic reaction 148
13.09 Adverse events – Chest pain/discomfort 148
13.10 Adverse events – Constipation 148
13.11 Adverse events – Diarrhoea 148
13.12 Adverse events – Dizziness 148
13.13 Adverse events – Dry mouth 148
13.14 Adverse events – Dyspepsia 149
13.15 Adverse events – Fatigue 149
13.16 Adverse events – Gastrointestinal bleeding 149
13.17 Adverse events – Hepatic toxicity 149
13.18 Adverse events – Major adverse cardiovascular events 149
13.19 Adverse events – Nausea 149
13.20 Adverse events – Paraesthesia 149

13.21 Adverse events – Pruritus ... 150
13.22 Adverse events – Sedation ... 150
13.23 Adverse events – Serotonergic syndrome ... 150
13.24 Adverse events – Vertigo ... 150
13.25 Adverse events – Vomiting ... 150

APPENDIX 14. SUBGROUP ANALYSES ... 151

14.1 Bayesian network meta-regression for the proportion of female participants ... 151

14.1.1 Pain freedom at 2 hours ... 151
14.1.2 Sustained pain freedom from 2 to 24 hours ... 151

14.2 Bayesian network meta-regression for the proportion of participants with aura at baseline ... 151

14.2.1. Pain freedom at 2 hours ... 151
14.2.2 Sustained pain freedom from 2 to 24 hours ... 151

APPENDIX 15. SENSITIVITY ANALYSES ... 152

15.1 Splitting of nodes that include low and high doses ... 152

15.1.1 Pain freedom at 2 hours ... 152
15.1.2 Sustained pain freedom from 2-24 hours ... 152

15.2 Inclusion of doses licensed by the FDA only ... 153

15.2.1 Pain freedom at 2 hours ... 153
15.2.2 Sustained pain freedom from 2-24 hours ... 153

15.3 Inclusion of studies with low risk of bias only ... 154

15.3.1 Pain freedom at 2 hours ... 154
15.3.2 Sustained pain freedom from 2-24 hours ... 154

15.4 Inclusion of studies where participants had menstrual migraine only ... 154

15.4.1 Pain freedom at 2 hours ... 154
15.4.2 Sustained pain freedom from 2-24 hours ... 154

15.5 Exclusion of studies where participants had comorbidity ... 154

15.5.1 Pain freedom at 2 hours ... 154
15.5.2 Sustained pain freedom from 2-24 hours ... 154

15.6 Exclusion of studies allowing use of preventive migraine medications ... 154

15.6.1 Pain freedom at 2 hours ... 154
15.6.2 Sustained pain freedom from 2-24 hours ... 154

15.7 Inclusion of studies where participants treated moderate or severe headache only ... 155

15.7.1 Pain freedom at 2 hours ... 155
15.7.2 Sustained pain freedom from 2-24 hours ... 155

15.8 Placebo response; only inclusion of studies after 1997 ... 155

15.8.1 Pain freedom at 2 hours ... 155
15.8.2 Sustained pain freedom from 2-24 hours ... 156

TABLE S1. DRUGS AND DOSES LICENSED BY INTERNATIONAL REGULATORY AGENCIES ... 157

TABLE S2. UNPUBLISHED RECORDS IDENTIFIED THROUGH OTHER SOURCES ... 159

TABLE S3. CHARACTERISTICS OF STUDIES INCLUDED THE SYSTEMATIC REVIEW 164

TABLE S4. INCLUSION AND EXCLUSION CRITERIA OF STUDIES INCLUDED IN THE SYSTEMATIC REVIEW 197

TABLE S5. CINEMA (CERTAINTY OF EVIDENCE) – PAIN FREEDOM AT 2 HOURS 251

TABLE S6. CINEMA (CERTAINTY OF EVIDENCE) – SUSTAINED PAIN FREEDOM FROM 2 TO 24 HOURS 257

TABLE S7. RISK OF BIAS – PAIN FREEDOM AT 2 HOURS 261

TABLE S8. RISK OF BIAS – SUSTAINED PAIN FREEDOM FROM 2 TO 24 HOURS 266

REFERENCES 269

Appendix 1. Study Protocol

1.1. Changes to the study protocol

1. **International clinician and patient representative panels:** To enhance the clinical value of the study results, we agreed to use the feedback from two international, independent panels: one consisting of physicians with expertise in migraine and one consisting of representatives from international patient organisations. These two panels provided their feedback regarding the study results to inform the selection of outcomes for the presented figures, and the final interpretation of the results and discussion in the manuscript. The physicians with expertise in migraine were members of the research team not involved in the statistical analysis (CID, HA, HCD, MA). International patient organisations were approached by one co-author (ERT), to identify representatives willing to partake in the interpretation of the results of this study and to provide their feedback on the manuscript. To minimize bias based on previous experience or knowledge of the drug, the three authors who conducted the statistical analyses (WKK, EGO, AC) presented the study results in a blinded fashion (i.e. the names of each intervention were masked) to the panels during a separate meeting for each panel.

2. **Post-hoc sensitivity analyses:** To evaluate transitivity, we agreed to perform three additional *post-hoc* sensitivity analyses of the primary outcomes: (i) inclusion only of doses licensed by the US Food and Drugs administration (FDA), (ii) exclusion of studies in which participants were not instructed to treat at moderate or severe intensity headache, and (iii) the impact of placebo response. To assess the impact of placebo response, we performed a meta-regression on the log-proportion of placebo responders by study year while controlling for the number of centres, proportion of female participants, proportion of participants with moderate or severe migraine, mean age, and recruitment regions. If study year was identified as significant, we decided to perform a time series analysis to identify any structural breaks in the data series, and further explore this by restricting the dataset to studies after any identified structural breaks.

3. **Second dosing:** To ensure transitivity, we agreed not to include outcome data in the analysis from trials that allowed a second dose of study medication for headache recurrence or relapse for rescue medication from 2 to 24 hours and adverse events, if: (i) the cumulative dose of the first and second dose of study medication exceeded the eligible dose range, or (ii) if participants assigned to placebo for the first dose received an active intervention as their second dose.

4. **Inclusion of trials:** Four identified trials enrolled participants below 18 years of age, although the majority of participants in these trials were adults (≥18 years of age).[1–4] We agreed to extract separate data for adults or, if unavailable, contact the authors. If separate data for adults could not be obtained, we agreed to consider the proportion of participants below 18 years of age and, if deemed unlikely to have substantial impact on the outcome data, we included the trial in the systematic review and network meta-analysis.

5. **Outcomes:** We defined hepatic toxicity broadly as any clinical signs of hepatic liver injury, or biochemical (including any elevation of one or more hepatic enzymes), imaging, or histopathological findings suggestive of hepatic injury. For brevity, we condensed ‘Fatigue/asthenia’ to ‘Fatigue’, ‘Sedation/somnolence’ to ‘Sedation’, and ‘Discomfort or pain in chest, throat and/or jaw’ to ‘Chest pain/discomfort’.

6. **Risk of bias:** For assessment of the Risk of Bias 2 (RoB2) domain 2 ‘deviations from intended interventions’, we considered certain modifications to the intention-to-treat (ITT) analysis appropriate due to their common use in clinical trials of acute migraine attacks.[5]. These included the exclusion of participants who did not take study medication (often due to the absence of a migraine attack qualifying for treatment according to study protocols) or participants for whom post-dose outcome data was missing from the analysis.[5]

1.2. Study protocol

The review protocol has been prospectively registered on Open Science Framework and is available at: https://osf.io/kq3ys/ (doi:10.17605/OSF.IO/KQ3YS). The original study protocol is presented on the following pages.

COMPARATIVE EFFECTS OF PHARMACOLOGICAL INTERVENTIONS FOR THE ACUTE MANAGEMENT OF MIGRAINE ATTACKS IN ADULTS: A SYSTEMATIC REVIEW AND NETWORK META-ANALYSIS

Protocol

(August 2022)

William Kristian Karlsson,[1] Edoardo Ostinelli,[2-4] Rune Häckert Christensen,[1] Lili Kokoti,[1] Zixuan Alice Zhuang,[1] Christina Deligianni,[1] Haidar Muhsen Al-Khazali,[1] Anneka Tomlinson,[2-4] Håkan Ashina,[1,5] Elena Ruiz de la Torre,[6] Hans-Christoph Diener,[7]
Andrea Cipriani,[2-4] Messoud Ashina[1]

[1] Danish Headache Centre, Department of Neurology, Righospitalet Glostrup, Faculty of Health and Medical Sciences, University of Copenhagen, Copenhagen, Denmark

[2] Department of Psychiatry, University of Oxford, Oxford, UK

[3] Oxford Health NHS Foundation Trust, Warneford Hospital, OX3 7JX Oxford, UK

[4] Oxford Precision Psychiatry Lab, NIHR Oxford Health Biomedical Research Centre, Oxford, UK

[5] Department of Anesthesia, Critical Care and Pain Medicine, Beth Israel Deaconess Medical Center, Harvard Medical School, Boston, MA, USA.

[6] European Migraine & Headache Alliance, Rue d'Egmont 11, 1000 Brusels, Belgium

[7] Unit of Neuroepidemiology, Institute for Medical Informatics, Biometry and Epidemiology Hufelandstraße 55, 45147 Essen, Germany

OBJECTIVES

To compare and rank acute oral pharmacological treatments for migraine attacks in adults in terms of efficacy.

Types of studies
We will include parallel-group, double-blind randomised trials regardless of publication status, type or language. We will include data from cross-over trials only until the first randomised period in cross-over trials, as suggested in the Cochrane Handbook;[6] however, if pre-cross-over results are not available, we will exclude the trial. Only trials carried out since 1988 will be considered, as this is when the diagnosis of migraine was standardised at an international level.[7]

Types of participants
Patients aged 18 years or older with migraine diagnosed according to the standardised criteria of the International Classification of Headache Disorders will be included.[8–11] There will be no limits in terms of gender or ethnicity. We will exclude studies on migraine including patients with a primary diagnosis of a concomitant somatic or psychiatric disorder. We will also exclude studies based in an emergency department-setting, as migraine attacks treated in emergency departments usually constitute a subgroup of particularly severe or atypical migraine attacks, that are often associated with reduced response to typically used acute oral migraine treatments.[12]

Types of interventions
We will include RCTs evaluating one of the following oral pharmacological interventions as oral monotherapy, compared to placebo and/or another eligible drug (i.e. tablet, oral disintegrating tablet, suspension, etc). All included drugs are licensed by at least one regulatory agency (see below) for the acute abortive treatment of migraine attacks or headache (Table 1). The included drugs will be grouped into the following drug classes.[13]

NSAIDs:
- Acetylsalicylic acid
- Celecoxib
- Diclofenac potassium
- Flurbiprofen
- Ibuprofen
- Ibuprofen lysine
- Ibuprofen sodium
- Ketoprofen
- Naproxen
- Naproxen sodium
- Phenazone
- Tolfenamic acid

Antipyretics:
- Paracetamol (acetaminophen)

Triptans:
- Almotriptan
- Eletriptan
- Frovatriptan
- Naratriptan
- Rizatriptan
- Sumatriptan
- Zolmitriptan

Ditans:
- Lasmiditan

Gepants:
- Rimegepant
- Ubrogepant

Other:
- Ergotamine tartrate

We will include trials where rescue medication (either investigational or non-investigational medications used to abort migraine attacks) was allowed. However, we will exclude trials assessing: (i) combination treatments; (ii) augmentation studies (e.g. drug A+ drug B versus drug A); (iii) all non-pharmacological treatments; (iv) medical devices; (v) one of the included drugs with a drug that is not listed above.
We assume that any patient who meets the inclusion criteria is, in principle, equally likely to be randomised to any of the eligible treatments.

Comparability of dosages

We will include only studies randomising patients to drugs within the licensed dose according to the British National Formulary (BNF, United Kingdom), European Medicines Agency (EMA, Europe), Food and Drugs Administration (FDA, United States of America), Federal Institute for Drugs and Medical Devices (Bundesinstitut für Arzneimittel und Medizinprodukte or BfArM, Germany), National Agency for the Adverse events of Medicines and Health Products (Agence Nationale de Sécurité du Médicament et des Produits de Santé or ANSM, France), Pharmaceuticals and Medical Devices Agency (PMDA, Japan), Therapeutic Goods Administration (TGA, Australia) (see Table 1). Only fixed-dose designs will be allowed. There is the possibility that some trials may compare one agent at the upper limit of its therapeutic range with another agent at the lower limit of its therapeutic range within the same study. We may look at heterogeneity and then add a binary variable (yes/no) to report if dosages are comparable and use this information for analysis.

Table 1. Therapeutic doses for oral forms of medications licensed for the abortive treatment of acute migraine attacks in adults, according to international regulatory agencies. **Legend:** ANSM, Agence Nationale de Sécurité du Médicament et des Produits de Santé (http://agence-prd.ansm.sante.fr/php/ecodex/index.php – France); BfArM, Bundesinstitut für Arzneimittel und Medizinprodukte (https://portal.dimdi.de/amguifree/am/search.xhtml – Germany); BNF, British National Formulary (https://bnf.nice.org.uk/ – United Kingdom); EMA, European Medicines Agency (https://www.ema.europa.eu – Europe); FDA, Food and Drugs Administration (https://www.fda.gov – United States of America); PMDA, Pharmaceuticals and Medical Devices Agency (https://www.pmda.go.jp – Japan); TGA, Therapeutic Goods Administration (https://www.tga.gov.au, https://www.pbs.gov.au/browse/medicine-listing – Australia). *: ANSM, BfArM or EMA; M: licensed for migraine; H: licensed for headache.

Drug	BNF	Europe*	FDA	PMDA	TGA	Therapeutic range (min-max)
Non-steroidal anti-inflammatory drugs (NSAIDs)						
Acetylsalicylic acid	300-900 mg (M)	BfArM: 500-1000 mg (H)	324-975 mg (H)	500-1500 mg (H)	300-1000 mg (M)	300-1500 mg
Celecoxib	-	-	120 mg (M)	-	-	120 mg
Diclofenac potassium	50 mg (M)	BfArM: 50 mg (M)	50 mg (M)	-	50 mg (M)	50 mg
Flurbiprofen	37.5-100 mg (M)	-	-	-	-	37.5-100 mg
Ibuprofen	400-600 mg (M)	ANSM: 200-400 mg (M)<br>BfArM: 200-400 mg (H)	200-400 mg (M)	200 mg (H)	200-400 mg (M)	200-600 mg
Ibuprofen sodium	256-512 mg (M)	ANSM: 256-512 mg (M)	256-512 mg (H)	-	256-512 mg (M)	256-512 mg
Ibuprofen lysine	342-684 mg (M)	ANSM, BfArM: 342-684 mg (M)	-	-	342-684 mg (M)	342-684 mg
Ketoprofen	-	ANSM: 75-150 mg (M)	-	-	-	75-150 mg
Naproxen	-	-	-	-	500 mg (M)	500 mg
Naproxen sodium	-	BfArM: 220-440 mg (H)	220-440 mg (H)		825 mg (M)	220-825 mg
Phenazone	-	BfArM: 1000 mg (M)	-	-	-	1000 mg
Tolfenamic acid	200 mg (M)	-	-	-	-	200 mg
Antipyretics						
Paracetamol	500-1000 mg (M)	ANSM, BfArM: 500-1000 mg (M)	1000-1300 mg (H)	300-1000 mg (H)	480-1330 mg (M)	300-1330 mg
Triptans						
Sumatriptan	50-100 mg (M)	ANSM, BfArM: 50-100 mg (M)	25-100 mg (M)	50-100 mg (M)	50-100 mg (M)	25-100 mg
Eletriptan	40-80 mg (M)	ANSM, BfArM: 40-80 mg (M)	20-40 mg (M)	20-40 mg (M)	40-80 mg (M)	20-80 mg
Rizatriptan	5-10 mg (M)	ANSM, BfArM: 5-10 mg (M)	5-10 mg (M)	10 mg (M)	10 mg (M)	5-10 mg
Zolmitriptan	2.5-5 mg (M)	ANSM, BfArM: 2.5-5 mg (M)	1.25-5 mg (M)	2.5-5 mg (M)	2.5-5 mg (M)	1.25-5 mg
Naratriptan	2.5 mg (M)	ANSM, BfArM: 2.5 mg (M)	1-2.5 mg (M)	2.5 mg (M)	2.5 mg (M)	1-2.5 mg
Almotriptan	12.5 mg (M)	ANSM, BfArM: 12.5 mg (M)	6.25-12.5 mg (M)	-	-	6.25-12.5 mg
Frovatriptan	2.5 mg (M)	ANSM, BfArM: 2.5 mg (M)	2.5 mg (M)	-	-	2.5 mg
Ditans						
Lasmiditan	-	EMA: 50-200 mg (M)	50-200 mg (M)	50-200 mg (M)	-	50-200 mg
Gepants						
Rimegepant	75 mg (M)	EMA: 75 mg (M)	75 mg (M)	-	-	75 mg
Ubrogepant	-	-	50-100 mg (M)	-	-	50-100 mg
Other						
Ergotamine tartrate	-	BfArM: 2 mg (M)	2 mg (M)	-	-	2 mg

OUTCOMES

We will assess all outcomes according to the current guidelines for controlled trials of acute treatment of migraine attacks in adults.[5] In studies evaluating the treatment of more than one migraine attack, the first treated attack will be used for evaluation of efficacy.

Primary outcomes

- 2-hour pain freedom, defined as the proportion of participants who become pain free at 2 hours after treatment, without the use of any rescue medication.
- Sustained pain freedom, defined as the proportion of participants who are pain free at 2 hours and do not relapse within 24 hours, without the use of any rescue medication.

Secondary outcomes

- Headache relief, defined as the proportion of participants that experience a decrease in headache pain from moderate or severe at baseline to mild or none at 2 hours after treatment, without the use of any rescue medication.
- Headache recurrence, defined as the proportion of participants who experience headache of any severity within 48 hours of the administration of the investigational drug among patients who were pain free 2 hours after the investigational drug was administered.
- Use of rescue medication, defined as the proportion of participants taking rescue medication from 2 to 24 hours after taking the initial trial medication.
- Proportion of participants that experience at least one of the following individual adverse events (see below for details about categorisation using MedDRA, https://www.meddra.org/): (i) abdominal pain, (ii) allergic reaction, (iii) constipation, (iv) diarrhoea, (v) dizziness, (vi) discomfort or pain in chest, throat and/or jaw, (vii) dry mouth, (viii) dyspepsia, (ix) hepatic toxicity, (x) fatigue/asthenia, (xi) gastrointestinal bleeding, (xii) major cardiovascular adverse event (i.e. non-fatal stroke, non-fatal myocardial infarction, fatal cardiovascular event), (xiii) nausea, (xiv) paraesthesia, (xv) pruritus, (xvi) Sedation, (xvii) serotonergic syndrome, (xviii) vertigo, (xix) vomiting.
- Proportion of participants experiencing at least one serious adverse event as defined by the FDA (https://www.fda.gov/).

SEARCH STRATEGY

As publication bias and selective outcome reporting can severely influence the findings of a systematic review, we will seek to include both published and unpublished studies. For identified unpublished studies, we will search for data from trial registries. If all relevant results are not available from trial registries, we will contact trial authors or sponsors in order to obtain additional data.

We will search the following databases from their inception without language restrictions:

- Cochrane Central Register of Controlled Trials (CENTRAL): (Migraine) AND ("Antipyrine" OR NSAID OR Acetylsalicylic acid OR Celecoxib OR Diclofenac OR Ibuprofen OR Ketoprofen OR Naproxen OR Phenazone OR Tolfenamic acid OR Antipyretic OR Paracetamol OR Acetaminophen OR Triptan OR Sumatriptan OR Eletriptan OR Rizatriptan OR Zolmitriptan OR Naratriptan OR Almotriptan OR Frovatriptan OR Ditan OR Lasmiditan OR Gepant OR Rimegepant OR Ubrogepant OR Ergotamine OR Aspirin OR Elyxyb OR Celebrex OR Cambia OR Zipsor OR Ibu OR Motrin OR Rufen OR Nuprin OR Midon OR Advil OR Orudis OR Naprosyn OR Anaprox OR Aleve OR Naprelan OR Clotam OR Tylenol OR Imitrex OR Relpax OR Maxalt OR Zomig OR Amerge OR Axert OR Frova OR Reyvow OR Nurtec OR Ubrelvy OR Ergostat OR Wigrettes OR Ergomar)
- Medline (via PubMED): ((Migraine[all fields] OR "Migraine Disorders"[Mesh]) AND ("Aspirin"[Mesh] OR "Celecoxib"[Mesh] OR "Diclofenac"[Mesh] OR "Ibuprofen"[Mesh] OR "Ketoprofen"[Mesh] OR "Naproxen"[Mesh] OR "Antipyrine"[Mesh] OR "tolfenamic acid"[Supplementary Concept] OR "Acetaminophen"[Mesh] OR "Sumatriptan"[Mesh] OR "eletriptan"[Supplementary Concept] OR "rizatriptan"[Supplementary Concept] OR "zolmitriptan"[Supplementary Concept] OR "naratriptan"[Supplementary Concept] OR "almotriptan"[Supplementary Concept] OR "frovatriptan"[Supplementary Concept] OR "lasmiditan"[Supplementary Concept] OR "rimegepant sulfate"[Supplementary Concept] OR "ubrogepant"[Supplementary Concept] OR "Ergotamine"[Mesh] OR NSAID[all fields] OR Acetylsalicylic acid[all fields] OR Celecoxib[all fields] OR Diclofenac[all fields] OR Ibuprofen[all fields] OR Ketoprofen[all fields] OR Naproxen[all fields] OR Phenazone[all fields] OR Tolfenamic acid[all fields] OR Antipyretic[all fields] OR Paracetamol[all fields] OR Acetaminophen[all fields] OR Triptan[all fields] OR Sumatriptan[all fields] OR Eletriptan[all fields] OR Rizatriptan[all fields] OR Zolmitriptan[all fields] OR Naratriptan[all fields] OR Almotriptan[all fields] OR Frovatriptan[all fields] OR

Ditan[all fields] OR Lasmiditan[all fields] OR Gepant[all fields] OR Rimegepant[all fields] OR Ubrogepant[all fields] OR Ergotamine[all fields] OR Aspirin[all fields] OR Celebrex[all fields] OR Cambia[all fields] OR Zipsor[all fields] OR Ibu[all fields] OR Motrin[all fields] OR Rufen[all fields] OR Nuprin[all fields] OR Midon[all fields] OR Advil[all fields] OR Orudis[all fields] OR Naprosyn[all fields] OR Anaprox[all fields] OR Aleve[all fields] OR Naprelan[all fields] OR Clotam[all fields] OR Tylenol[all fields] OR Imitrex[all fields] OR Relpax[all fields] OR Maxalt[all fields] OR Zomig[all fields] OR Amerge[all fields] OR Axert[all fields] OR Frova[all fields] OR Reyvow[all fields] OR Nurtec[all fields] OR Ubrelvy[all fields] OR Ergostat[all fields] OR Wigrettes[all fields] OR Ergomar[all fields]) AND (randomized controlled trial[pt] OR controlled clinical trial[pt] OR random[tiab] OR placebo[tiab] OR clinical trials as topic[mesh:noexp] OR trial[ti])) NOT (animals[mh] NOT humans[mh])

- Embase (via OvidSP): ((Migraine.mp.) AND (NSAID*.mp. OR Acetylsalicylic acid*.mp. OR Celecoxib*.mp. OR Dexketoprofen*.mp. OR Diclofenac*.mp. OR Ibuprofen*.mp. OR Ketoprofen*.mp. OR Naproxen*.mp. OR Phenazone*.mp. OR Tolfenamic acid*.mp. OR Antipyretic*.mp. OR Paracetamol*.mp. OR Acetaminophen*.mp. OR Triptan*.mp. OR Sumatriptan*.mp. OR Eletriptan*.mp. OR Rizatriptan*.mp. OR Zolmitriptan*.mp. OR Naratriptan*.mp. OR Almotriptan*.mp. OR Frovatriptan*.mp. OR Ditan*.mp. OR Lasmiditan*.mp. OR Gepant*.mp. OR Rimegepant*.mp. OR Ubrogepant*.mp. OR Ergotamine*.mp. OR Aspirin.mp. OR Celebrex.mp. OR Cambia.mp. OR Zipsor.mp. OR Ibu.mp. OR Motrin.mp. OR Rufen.mp. OR Nuprin.mp. OR Midon.mp. OR Advil.mp. OR Orudis.mp. OR Naprosyn.mp. OR Anaprox.mp. OR Aleve.mp. OR Naprelan.mp. OR Clotam.mp. OR Tylenol.mp. OR Imitrex.mp. OR Relpax.mp. OR Maxalt.mp. OR Zomig.mp. OR Amerge.mp. OR Axert.mp. OR Frova.mp. OR Reyvow.mp. OR Nurtec.mp. OR Ubrelvy.mp. OR Ergostat.mp. OR Wigrettes.mp. OR Ergomar.mp.)) NOT ((exp animal/ or animal experiment/ or nonhuman/) not (exp human/ or human experiment/))
  limit 1 to (clinical trial or randomized controlled trial or controlled clinical trial or phase 1 clinical trial or phase 2 clinical trial or phase 3 clinical trial or phase 4 clinical trial)

To identify ongoing or unpublished trials, we will supplement the electronic database searches with manual searches of the following websites:

- ClinicalTrials.gov
- World Health Organization (WHO) International Clinical Trials Registry Platform (ICTRP)
- Regulatory agencies' websites (ANSM, BfArM, EMA, FDA, PMDA, TGA)
- Pharmaceutical companies' registries/websites

In addition, we will hand search the reference lists of the included trials and relevant systematic reviews and meta-analyses, editorials and guidelines on acute treatment of adults with migraine to identify additional studies missed from the original electronic searches. We will also contact trialists and migraine experts for information on unpublished or ongoing studies. We will not restrict our search by language, date or publication status.

DATA EXTRACTION

At least two review authors will independently screen titles and abstracts retrieved by the search strategy. Full texts of potentially relevant studies will then be assessed independently by at least two authors, who will also extract relevant information from each study. Disagreements will be resolved through discussion with a third member of the review team.

Categorisation of adverse events

We will categorise adverse events using MedDRA (https://www.meddra.org/) as detailed in Tomlinson et al., 2019.[14] Briefly, at least two independent researchers will extract the number of participants experiencing each adverse effect, as reported in the trials, and will then use preferred terms to categorise each adverse event. There are five levels to the MedDRA hierarchy, arranged from very specific to very general. At the most specific level, called "Lowest Level Terms" (LLTs), there are more than 70,000 terms which parallel how information is communicated. These LLTs reflect how an observation might be reported in practice (i.e. in a specific study). This level directly supports assigning MedDRA terms within a user database. Each member of the next level, "Preferred Terms" (PTs), is a distinct descriptor (single medical concept) for a symptom, sign or disease diagnosis. Each LLT is linked to only one PT and each PT has at least one LLT (itself) as well as synonyms and lexical variants (e.g., abbreviations, different word order). If we find different MedDRA terms to identify similar adverse events, these synonyms will be merged using clinical judgement into broader categories (as applicable) and validated by another clinician. Any discrepancies will be solved by consensus within the review team.

Assessment of risk of bias in included studies
At least two review authors will independently assess the risk of bias in primary outcomes using the Cochrane Collaborations Risk of Bias 2.0 (RoB 2) and the criteria outlined in the Cochrane Handbook for Systematic Reviews of Interventions.[6] We will resolve any disagreements by discussion or by involving another author. RoB 2 is structured into a fixed set of domains of bias, focusing on different aspects of trial design, conduct and reporting: bias arising from the randomization process; bias due to deviations from intended interventions; bias due to missing outcome data; bias in measurement of the outcome; and bias in selection of the reported result. Within each domain, a series of questions ('signalling questions') aim to elicit information about features of the trial that are relevant to risk of bias. Judgements can be 'Low', or 'High' risk of bias, or can express 'Some concerns'. The study-specific overall risk of bias for each primary outcome will be determined following the instructions provided in the RoB 2 handbook.

DATA ANALYSIS
We will estimate summary odds ratios (ORs) for dichotomous outcomes with their 95% confidence intervals (CIs) using pairwise and network meta-analysis.[15] We will use the netmeta package in R. We will assess statistical heterogeneity in each pairwise and network meta-analysis comparison with tau^2 and I^2 statistics.[6] We will carry out network meta-analyses using a random effects model within a frequentist setting, assuming equal heterogeneity across all comparisons and accounting for correlations induced by multi-arm studies. For rare events (e.g. for studies with no events in some of the treatment groups), we will use a fixed-effect Mantel-Haenszel approach.[16]

Dealing with missing data
We will contact study authors when there are missing or unclear data. If dichotomous outcome data are still missing, they will be managed according to the intention-to-treat (ITT) principle, and we will assume that patients who dropped out after randomisation had a negative outcome. For serious adverse events, we will assume patients who dropped out did not have a negative outcome.

Assessment of transitivity across treatment comparisons
We expect that the transitivity assumption will hold assuming that all pairwise comparisons do not differ on average with respect to the distribution of effect modifiers (e.g. age). The assumption of transitivity will be evaluated in each primary outcome by comparing the clinical and methodological characteristics, such as age and studies reported as high risk of bias.

Assessment of reporting biases
The possibility of reporting bias will be evaluated for each outcome by means of the contour-enhanced funnel plots if enough studies (at least 10) are available.[17] These are funnel plots showing areas of statistical significance and they can help to distinguish publication bias from other possible reasons for asymmetry. In a network of interventions each study estimates the relative effect of different interventions, so asymmetry in the funnel plot cannot be judged. To account for this, we will use an adaptation of the funnel plot by subtracting from each study-specific effect size the mean of meta-analysis of the study-specific comparison and plot it against the study's standard error.[18,19] We will draw the comparison-adjusted funnel plot for all placebo-controlled trials (if at least 10 trials are available). Any asymmetry in the plot indicates the presence of small study effects and not necessarily reporting bias.

Assessment of heterogeneity, transitivity, inconsistency and certainty of evidence
In the context of the network meta-analysis, we will assume a common within-network heterogeneity and the generalised Q-statistic estimator will be used for the heterogeneity variance.[20] To evaluate the presence of heterogeneity deriving from different trial designs or different clinical characteristics of study participants, we will generate descriptive statistics for trial and study population characteristics across all eligible trials that compare each pair of interventions. We will assess the presence of clinical heterogeneity within each pairwise comparison by comparing these characteristics. We will evaluate the transitivity assumption by comparing the distribution of key study characteristics across studies grouped by comparison. We will assess inconsistency between direct and indirect sources of evidence using global and local approaches.[21] We will assess global inconsistency using a design-by-treatment test.[22] We will evaluate local inconsistency using the back calculation and separate indirect from direct design evidence methods, comparing direct and indirect evidence for each pairwise treatment comparison.[23] A hierarchy of treatments will be calculated for each outcome, on the basis of the p-scores.[24] We will assess existence of small-study effects and publication bias for each treatment pair using a contour-enhanced funnel plot if at least ten studies that did the analysis were available (see above for further details).[17]
We will assess the certainty of evidence using the Confidence in Network Meta-Analysis framework (CINeMA).[25] CINeMA is a software which uses the netmeta R-package to perform network meta-analysis of the data. We will assess each network estimate according to study limitation, indirectness, inconsistency, imprecision, and publication bias.

Finally, we will assign to each comparison an overall qualitative judgment based on four levels of quality of evidence: high, moderate, low, very low.

Subgroup and sensitivity analyses

We will evaluate possible heterogeneity of treatment effects and the robustness of our findings about the primary outcomes with subgroup network meta-analyses,[26] using gender (male vs female) and presence of aura (with aura vs without aura) as covariates. We will carry out the following sensitivity analyses: (i) splitting nodes that included high and low doses, (ii) including only studies with low risk of bias or (iii) diagnosis of menstrual migraine, (iv) excluding studies with concomitant medical/psychiatric comorbidity or (v) with participants using preventive medication. We will present the findings from network meta-analyses using league tables and Vitruvian plots. Vitruvian plots are radial bar benefit-risk communication tools that synthesise the results of multiple outcomes.[27]

Appendix 2. Search Strategy

2.1 Cochrane Central Register of Controlled Trials (CENTRAL) (via CRSO)

We searched the Cochrane Central Register of Controlled Trials (CENTRAL) from inception and onwards (last update: 24th June 2023) via The Cochrane Register of Studies Online (CRS-O) using the following search string:
“(Migraine) AND ("Antipyrine" OR NSAID OR Acetylsalicylic acid OR Celecoxib OR Diclofenac OR Ibuprofen OR Ketoprofen OR Naproxen OR Phenazone OR Tolfenamic acid OR Antipyretic OR Paracetamol OR Acetaminophen OR Triptan OR Sumatriptan OR Eletriptan OR Rizatriptan OR Zolmitriptan OR Naratriptan OR Almotriptan OR Frovatriptan OR Ditan OR Lasmiditan OR Gepant OR Rimegepant OR Ubrogepant OR Ergotamine OR Aspirin OR Elyxyb OR Celebrex OR Cambia OR Zipsor OR Ibu OR Motrin OR Rufen OR Nuprin OR Midon OR Advil OR Orudis OR Naprosyn OR Anaprox OR Aleve OR Naprelan OR Clotam OR Tylenol OR Imitrex OR Relpax OR Maxalt OR Zomig OR Amerge OR Axert OR Frova OR Reyvow OR Nurtec OR Ubrelvy OR Ergostat OR Wigrettes OR Ergomar)”.

2.2 Embase (via Ovid)

We searched Embase via Ovid Medline from inception and onwards (last update: 24th June 2023) using the following search string:
“((Migraine.mp.) AND (NSAID*.mp. OR Acetylsalicylic acid*.mp. OR Celecoxib*.mp. OR Dexketoprofen*.mp. OR Diclofenac*.mp. OR Ibuprofen*.mp. OR Ketoprofen*.mp. OR Naproxen*.mp. OR Phenazone*.mp. OR Tolfenamic acid*.mp. OR Antipyretic*.mp. OR Paracetamol*.mp. OR Acetaminophen*.mp. OR Triptan*.mp. OR Sumatriptan*.mp. OR Eletriptan*.mp. OR Rizatriptan*.mp. OR Zolmitriptan*.mp. OR Naratriptan*.mp. OR Almotriptan*.mp. OR Frovatriptan*.mp. OR Ditan*.mp. OR Lasmiditan*.mp. OR Gepant*.mp. OR Rimegepant*.mp. OR Ubrogepant*.mp. OR Ergotamine*.mp. OR Aspirin.mp. OR Celebrex.mp. OR Cambia.mp. OR Zipsor.mp. OR Ibu.mp. OR Motrin.mp. OR Rufen.mp. OR Nuprin.mp. OR Midon.mp. OR Advil.mp. OR Orudis.mp. OR Naprosyn.mp. OR Anaprox.mp. OR Aleve.mp. OR Naprelan.mp. OR Clotam.mp. OR Tylenol.mp. OR Imitrex.mp. OR Relpax.mp. OR Maxalt.mp. OR Zomig.mp. OR Amerge.mp. OR Axert.mp. OR Frova.mp. OR Reyvow.mp. OR Nurtec.mp. OR Ubrelvy.mp. OR Ergostat.mp. OR Wigrettes.mp. OR Ergomar.mp.)) NOT ((exp animal/ or animal experiment/ or nonhuman/) not (exp human/ or human experiment/))
limit 1 to (clinical trial or randomized controlled trial or controlled clinical trial or phase 1 clinical trial or phase 2 clinical trial or phase 3 clinical trial or phase 4 clinical trial)”.

2.3 Medline (via PubMed)

We searched PubMed from inception and onwards (last update: 24th June 2023) using the following search string:
“((Migraine[all fields] OR "Migraine Disorders"[Mesh]) AND ("Aspirin"[Mesh] OR "Celecoxib"[Mesh] OR "Diclofenac"[Mesh] OR "Ibuprofen"[Mesh] OR "Ketoprofen"[Mesh] OR "Naproxen"[Mesh] OR "Antipyrine"[Mesh] OR "tolfenamic acid"[Supplementary Concept] OR "Acetaminophen"[Mesh] OR "Sumatriptan"[Mesh] OR "eletriptan"[Supplementary Concept] OR "rizatriptan"[Supplementary Concept] OR "zolmitriptan"[Supplementary Concept] OR "naratriptan"[Supplementary Concept] OR "almotriptan"[Supplementary Concept] OR "frovatriptan"[Supplementary Concept] OR "lasmiditan"[Supplementary Concept] OR "rimegepant sulfate"[Supplementary Concept] OR "ubrogepant"[Supplementary Concept] OR "Ergotamine"[Mesh] OR NSAID[all fields] OR Acetylsalicylic acid[all fields] OR Celecoxib[all fields] OR Diclofenac[all fields] OR Ibuprofen[all fields] OR Ketoprofen[all fields] OR Naproxen[all fields] OR Phenazone[all fields] OR Tolfenamic acid[all fields] OR Antipyretic[all fields] OR Paracetamol[all fields] OR Acetaminophen[all fields] OR Triptan[all fields] OR Sumatriptan[all fields] OR Eletriptan[all fields] OR Rizatriptan[all fields] OR Zolmitriptan[all fields] OR Naratriptan[all fields] OR Almotriptan[all fields] OR Frovatriptan[all fields] OR Ditan[all fields] OR Lasmiditan[all fields] OR Gepant[all fields] OR Rimegepant[all fields] OR Ubrogepant[all fields] OR Ergotamine[all fields] OR Aspirin[all fields] OR Celebrex[all fields] OR Cambia[all fields] OR Zipsor[all fields] OR Ibu[all fields] OR Motrin[all fields] OR Rufen[all fields] OR Nuprin[all fields] OR Midon[all fields] OR Advil[all fields] OR Orudis[all fields] OR Naprosyn[all fields] OR Anaprox[all fields] OR Aleve[all fields] OR Naprelan[all fields] OR Clotam[all fields] OR Tylenol[all fields] OR Imitrex[all fields] OR Relpax[all fields] OR Maxalt[all fields] OR Zomig[all fields] OR Amerge[all fields] OR Axert[all fields] OR Frova[all fields] OR Reyvow[all fields] OR Nurtec[all fields] OR Ubrelvy[all fields] OR Ergostat[all fields] OR Wigrettes[all fields] OR Ergomar[all fields]) AND (randomized controlled trial[pt] OR controlled clinical trial[pt]

OR random[tiab] OR placebo[tiab] OR clinical trials as topic[mesh:noexp] OR trial[ti])) NOT (animals[mh] NOT humans[mh])”.

2.4 International Trial Registries

2.4.1 ClinicalTrials.Gov

We performed individual searches on ClinicalTrials.Gov (https://clinicaltrials.gov/) for all available years (last update: 24th June 2023) using the advanced search function using the following strategy:

CONDITION = migraine
and
OTHER TERMS = individual names of included pharmaceuticals (i.e., "acetaminophen" OR "paracetamol" OR "acetylsalicylic acid" OR "aspirin" OR "almotriptan" OR "celecoxib" OR "diclofenac potassium" OR "eletriptan" OR "ergotamine" OR "ergotamine-tartrate" OR "flurbiprofen" OR "frovatriptan" OR "ibuprofen" OR "ketoprofen" OR "lasmiditan" OR "naproxen" OR "naratriptan" OR "phenazone" OR "rimegepant" OR "rizatriptan" OR "sumatriptan" OR "tolfenamic acid" OR "ubrogepant" OR "zolmitriptan").

2.4.2 European Union Clinical Trials Registry (EUCTR)

We performed individual searches on EUCTR (https://www.clinicaltrialsregister.eu/ctr-search/search) for all available years (last update: 24th June 2023) for each intervention combining each drug name with the keyword: "migraine" (as above for ClinicalTrials.Gov).

2.4.3 World Health Organization (WHO) International Clinical Trials Registry Platform

We performed individual searches on the WHO International clinical Trials Registry Platform (https://trialsearch.who.int/) for all available years (last update: 24th June 2023) for each intervention combining each drug name with the keyword: "migraine" (as above for ClinicalTrials.Gov).

2.5 Websites of Regulatory Agencies

We searched the websites of regulatory agencies to identify relevant documents on drugs indicated for the acute treatment of migraine or headache in adults (last update: 24th June 2023). We performed the search for each included drug on the following regulatory agency websites:

- **British National Formulary (BNF); United Kingdom**
  https://www.medicinescomplete.com/
- **L’Agence Nationale de Sécurité du Médicament et des Produits de Santé (ANSM); France**
  https://ansm.sante.fr/
- **Bundesinstitut für Arzneimittel und Medizinprodukte (BfArM); Germany**
  https://www.bfarm.de/
- **European Medicines Agency (EMA); Europe**
  https://www.ema.europa.eu/
- **Food and Drugs Administration (FDA); USA**
  https://www.fda.gov/
- **Pharmaceuticals and Medical Devices Agency (PMDA); Japan**
  https://www.pmda.go.jp/
- **Therapeutic Goods Administration (TGA); Australia**
  https://www.tga.gov.au/

2.6 Websites of Pharmaceutical Companies

We searched the websites of pharmaceutical companies to identify relevant documents on drugs for headache or migraine in adults (last update: 24th June 2023). The following pharmaceutical company websites contained lists or search engines to identify information on ongoing or completed clinical trials, or clinical study reports and other study results. We searched for each individual included drug on the following pharmaceutical company websites:

- **Abbvie**
  https://www.abbvieclinicaltrials.com/clinical-trials-near-me/
- **AstraZeneca**
  https://www.astrazenecaclinicaltrials.com/
- **Bayer**
  https://clinicaltrials.bayer.com/
- **Biohaven**
  https://www.biohaven.com/pipeline/clinical-trials/
- **Eli Lilly**
  https://www.lilly.com/clinical-research/clinical-trials
- **Endo Pharma**
  https://www.endo.com/r-d/clinical-research
- **Glaxo-Smith Kline**
  https://www.gsk-studyregister.com/en/
- **Janssen**
  https://www.janssen.com/clinical-trials/transparency
  https://yoda.yale.edu/
- **Merck**
  https://www.merckclinicaltrials.com/
- **Novartis**
  https://www.novctrd.com/
- **Pfizer**
  https://www.pfizer.com/science/clinical-trials/data-and-results

Appendix 3. Eligible interventions

We included RCTs evaluating one of the following oral pharmacological interventions as oral monotherapy compared to placebo and/or another eligible drug (i.e. tablet, oral disintegrating tablet, suspension, etc.). All included drugs had to be licensed by at least one regulatory agency (see below) for the acute abortive treatment of migraine attacks or headache (Table S1). The included drugs were grouped into the following drug classes:[13]

Antipyretics:

- Paracetamol (acetaminophen)

Ditans:

- Lasmiditan

Gepants:

- Rimegepant
- Ubrogepant

NSAIDs:

- Acetylsalicylic acid
- Celecoxib
- Diclofenac potassium
- Flurbiprofen
- Ibuprofen
- Ibuprofen lysine
- Ibuprofen sodium
- Ketoprofen
- Naproxen
- Naproxen sodium
- Phenazone
- Tolfenamic acid

Triptans:

- Almotriptan
- Eletriptan
- Frovatriptan
- Naratriptan
- Rizatriptan
- Sumatriptan
- Zolmitriptan

Other:

- Ergotamine tartrate

We included trials where rescue medication (either investigational or non-investigational medications used to abort migraine attacks) was allowed. However, we excluded trials assessing: (i) combination treatments; (ii) augmentation studies (e.g. drug A+ drug B versus drug A); (iii) all non-pharmacological treatments; (iv) medical devices; (v) one of the included drugs with a drug not listed above.

We assumed that any patient meeting the inclusion criteria were, in principle, equally likely to be randomised to any of the eligible treatments.

Appendix 4. Studies included in the systematic review

References of published and unpublished data are listed below for each of the studies included in the systematic review.

#: Included in the systematic review but not in the network meta-analysis

*: Included in the systematic review and network meta-analysis

1. 0462-039 1996 *

- US Food and Drug Administration. Drug approval package: Rizatriptan / Maxalt RPD (20-865) - Clinical Review. 1998 p. 1–48.

2. 103 UN *

- Sanis Health Inc. Product Monograph: Eletriptan. 2021 p. 1–34.
- US Food and Drug Administration. Approved Labeling: Eletriptan / Relpax (21-016). 1999 p. 1–18.
- US Food and Drug Administration. Drug approval package: Eletriptan / Relpax (21-016) - Medical Review. 1999 p. 1–121.
- US Food and Drug Administration. Administrative Documents: Eletriptan / Relpax (21-016). 2002.

3. 311CIL0099 1999 *

- AstraZeneca pharmaceuticals. Clinical Study Report: A Multicentre, Randomised, Double-Blind Trial to Compare the Efficacy and Safety of ZOMIG 2.5 mg, NARAMIG 2.5 mg and Placebo in the Acute Treatment of Adult Patients with Migraine (311CIL/0099). 2000 p. 1–5.

4. 311CUS0003 2002 #

- AstraZeneca. Clinical Study Report: A Multicenter, 2-phase, Double-blind, Randomized, Placebo-controlled, Parallel Trial to Evaluate the Efficacy of a Single Dose of Zolmitriptan (ZOMIG®) as Acute Treatment in Phase I and Repeated Doses as Preemptive Treatment in Phase II for Menstrual Migraine. 2005 p. 1–10.

5. 97-030 UN *

- US Food and Drug Administration. Drug approval package: Ibuprofen / Motrin Migraine (19-012) - Medical Review. 1999 p. 1–248.
- US Food and Drug Administration. Drug approval package: Ibuprofen / Motrin Migraine (19-012) - Statistical Review. 1999 p. 1–20.

6. Adwan 2004 #

- Adwan Z. Zolmitriptan 5 mg orally disintegrating tablets deliver a 30-min onset of action and high sustained headache response in a placebo-controlled trial. Cephalalgia. 2004;24:792–3.

7. Ahrens 1999 *

- Ahrens SP, Farmer MV, Williams DL, Willoughby E, Jiang K, Block GA, et al. Efficacy and safety of rizatriptan wafer for the acute treatment of migraine. Cephalalgia. 1999;19(5):525–30.

8. Allais 2010 #

- Allais G, D’Andrea G, Moschiano F, D’Onofrio F, Valguarnera F, Manzoni G, et al. A randomized, prospective, cross-over, double blind, placebo-controlled multicentre study to assess the efficacy and tolerability of almotriptan 12.5 mg in menstrually-related migraine. Cephalalgia. 2009;29:13.
- Allais G, Acuto G, Benedetto C, D’Andrea G, Grazzi L, Manzoni GC, et al. Evolution of migraine-associated symptoms in menstrually related migraine following symptomatic treatment with almotriptan. Neurological Sciences. 2010;31(SUPPL.1):2–6.
- Allais G, Bussone G, D’Andrea G, Moschiano F, D’Onofrio F, Valguarnera F, et al. Almotriptan 12.5 mg in menstrually related migraine: A randomized, double-blind, placebo-controlled study. Cephalalgia. 2011;31(2):144–51.
- European Union Clinical Trials Registry. 2005-000244-90 [Internet]. 2005 [cited 2023 Jun 24]. Available from: https://www.clinicaltrialsregister.eu/ctr-search/trial/2005-000244-90/IT
- Prodesfarma SAA. Study synopsis: A randomized, prospective, cross-over, double blind, placebo-controlled multicentre study to assess the efficacy and tolerability of Almotriptan 12.5 mg in the mild pain phase of Menstrual Migraine (MM) followed by an open follow-up evalua. 2009.
- WHO ITCRP. EUCTR2005-000244-90 [Internet]. 2005 [cited 2023 Jun 24]. Available from: https://trialsearch.who.int/Trial2.aspx?TrialID=EUCTR2005-000244-90-IT

9. ANODYNE-2 2018 #

- ClinicalTrials.gov. NCT03185143 [Internet]. 2017 [cited 2023 Jun 24]. Available from: https://clinicaltrials.gov/study/NCT03185143

10. Ashina 2021 *

- Ashina M, Krikke-Workel J, Krege J, Smith T, Lin Q, Klise S, et al. Randomized, Controlled Trial of Lasmiditan over Four Migraine Attacks: Findings from the CENTURION study. Neurology. 2021;96(15 Supplement).
- Ashina M, Reuter U, Smith T, Krikke-Workel J, Klise SR, Bragg S, et al. Randomized, controlled trial of lasmiditan over four migraine attacks: Findings from the CENTURION study. Cephalalgia. 2021;41(3):294–304.
- Ashina M, Reuter U, Smith T, Krikke-Workel J, Klise SR, Bragg S, et al. Supplemental Material: List of Ethical Review Boards for the CENTURION Study. Cephalalgia [Internet]. 2021;41. Available from: https://journals.sagepub.com/doi/full/10.1177/0333102421989232?rfr_dat=cr_pub++0pubmed&url_ver=Z39.88-2003&rfr_id=ori%3Arid%3Acrossref.org
- Ashina M, Reuter U, Smith T, Krikke-Workel J, Klise SR, Bragg S, et al. Supplemental Material: Patient Eligibility Criteria. Cephalalgia [Internet]. 2021;41. Available from: https://journals.sagepub.com/doi/full/10.1177/0333102421989232?rfr_dat=cr_pub++0pubmed&url_ver=Z39.88-2003&rfr_id=ori%3Arid%3Acrossref.org
- Ashina M, Reuter U, Smith T, Krikke-Workel J, Klise SR, Bragg S, et al. Supplemental Material: Supplemental Figures. Cephalalgia [Internet]. 2021;41. Available from: https://journals.sagepub.com/doi/full/10.1177/0333102421989232?rfr_dat=cr_pub++0pubmed&url_ver=Z39.88-2003&rfr_id=ori%3Arid%3Acrossref.org
- Ashina M, Reuter U, Smith T, Krikke-Workel J, Klise SR, Bragg S, et al. Supplemental Material: Supplemental Tables. Cephalalgia [Internet]. 2021;41. Available from: https://journals.sagepub.com/doi/full/10.1177/0333102421989232?rfr_dat=cr_pub++0pubmed&url_ver=Z39.88-2003&rfr_id=ori%3Arid%3Acrossref.org
- Ashina M, Reuter U, Smith T, Krikke-Workel J, Klise SR, Bragg S, et al. Supplemental Material: Visual Abstract. Cephalalgia [Internet]. 2021;41. Available from: https://journals.sagepub.com/doi/full/10.1177/0333102421989232?rfr_dat=cr_pub++0pubmed&url_ver=Z39.88-2003&rfr_id=ori%3Arid%3Acrossref.org
- ClinicalTrials.gov. NCT03670810 [Internet]. 2018 [cited 2023 Jun 24]. Available from: https://clinicaltrials.gov/study/NCT03670810

- Eli Lilly, Company. Statistical analysis plan: H8H-MC-LAIJ (version 2): Randomized Controlled Trial of Lasmiditan Over Four Migraine Attacks: NCT03670810. 2020 p. 1–76.
- Eli Lilly, Company. Protocol: H8H-MC-LAIJ (b): Randomized Controlled Trial Over Four Attacks: NCT03670810. 2020 p. 1–84.
- European Medicines Agency. Assessment Report: Lasmiditan / Raywow. 2022 p. 1–141.
- European Union Clinical Trials Registry. 2018-001661-17 [Internet]. 2018 [cited 2023 Jun 24]. Available from: https://www.clinicaltrialsregister.eu/ctr-search/trial/2018-001661-17/DE
- India (CTRI) CTR. CTRI/2019/05/019167: Lasmiditan study on patients Over Four Migraine Attacks [Internet]. 2019 [cited 2023 Jun 24]. Available from: https://ctri.nic.in/
- Reuter U, Krege JH, Lombard L, Valderas EG, Krikke-Workel J, Dell-Agnello G, et al. Lasmiditan efficacy in the acute treatment of migraine was independent of prior response to triptans: Findings from the CENTURION study. Cephalalgia. 2022;42(1):20–30.
- Reuter U, Lombard L, Krege J, Krikke-Workel J, Wilhelm S, Kovacik AJ, et al. Lasmiditan is Effective in the Acute Treatment of Migraine in Patients with Insufficient Response to Triptans: Findings from the CENTURION Study. Cephalalgia. 2021;96(15 Supplement).
- Smith T, Krikke-Workel J, Krege J, Ashina M, Lin Q, Klise S, et al. Randomized, Controlled Trial of Lasmiditan over Four Migraine Attacks: First Attack Findings. Neurology. 2021;96(15 Supplement).
- Tassorelli C, Bragg S, Krege J, Doty E, Ardayfio P, Ruff D, et al. Safety findings from CENTURION, a phase 3 consistency study of lasmiditan for the acute treatment of migraine. Journal of Headache and Pain. 2021;22(1):1–8.
- WHO ITCRP. EUCTR2018-001661-17-NL [Internet]. 2018 [cited 2023 Jun 24]. Available from: https://trialsearch.who.int/Trial2.aspx?TrialID=CTRI/2019/05/019167
- Yu T, He L, Yang X, Zhou J, Luo G, Wang H, et al. Efficacy and Safety of Lasmiditan as a Novel Acute Treatment in Chinese Patients with Migraine: A Subpopulation Analysis of the Randomized Controlled Phase 3 CENTURION Trial. Neurology and Therapy. 2022;11(3):1269–83.

11. Barbanti 2012 *

- Barbanti P, Fofi L, Dall'Armi V, Aurilia C, Egeo G, Vanacore N, et al. Rizatriptan in migraineurs with unilateral cranial autonomic symptoms: A double-blind trial. Journal of Headache and Pain. 2012;13(5):407–14.
- ClinicalTrials.gov. NCT00753311 [Internet]. 2009 [cited 2023 Jun 24]. Available from: https://clinicaltrials.gov/study/NCT00753311
- European Union Clinical Trials Registry. 2008-007967-18 [Internet]. 2012 [cited 2023 Jun 24]. Available from: https://www.clinicaltrialsregister.eu/ctr-search/trial/2008-007967-18/IT
- WHO ITCRP. EUCTR2008-007967-18-IT [Internet]. 2012 [cited 2023 Jun 24]. Available from: https://trialsearch.who.int/Trial2.aspx?TrialID=EUCTR2008-007967-18-IT

12. Bartolini 2011 #

- Bartolini M, Giamberardino MA, Lisotto C, Martelletti P, Moscato D, Panascia B, et al. Frovatriptan versus almotriptan for acute treatment of menstrual migraine: Analysis of a double-blind, randomized, cross-over, multicenter, Italian, comparative study. Journal of Headache and Pain. 2012;13(5):401–6.
- Bartolini M, Giamberardino MA, Lisotto C, Martelletti P, Moscato D, Panascia B, et al. A double-blind, randomized, multicenter, Italian study of frovatriptan versus almotriptan for the acute treatment of migraine. Journal of Headache and Pain. 2011;12(3):361–8.
- European Union Clinical Trials Registry. 2006-006574-21 [Internet]. 2007 [cited 2023 Jun 24]. Available from: https://www.clinicaltrialsregister.eu/ctr-search/trial/2006-006574-21/IT
- The Menarini Group. Synopsis of Clinical Study Report: Gui/06/Fro-pp/002; EUCTR2006-006574-21. 2011.
- WHO ITCRP. EUCTR2006-006574-21-IT [Internet]. 2007 [cited 2023 Jun 24]. Available from: https://trialsearch.who.int/Trial2.aspx?TrialID=EUCTR2006-006574-21-IT

13. **Bomhof 1999 ***

- Bomhof M, Paz J, Legg N, Allen C, Vandormael K, Patel K, et al. Comparison of rizatriptan 10 mg versus zolmitriptan 2.5 mg in migraine. European Neurology. 1999;42(3):173–9.

14. **Boureau 1994 #**

- Boureau F, Joubert JM, Lasserre V, Prum B, Delecoeuillerie G. Double-blind comparison of an acetaminophen 400 mg-codeine 25 mg combination versus aspirin 1000 mg and placebo in acute migraine attack. Cephalalgia. 1994;14:156–61.

15. **Brandes 2005 ***

- Brandes J, Hilliard B, Sikes C, Tiseo P. Treatment of migraine in the early stages of a attack: a prospective, double-blind, placebo-controlled trial of eletriptan. Cephalalgia. 2005;25:641–83.
- Brandes J, Sikes C, Hilliard B. Eletriptan in the early treatment of migraine: a prospective, double-blind, placebo-controlled trial. European Journal of Neurology. 2004;11:93.
- Brandes J, Sikes C, J H. Early treatment with eletriptan: a prospective, double-blind, placebo-controlled trial. Cephalalgia. 2004;24:775–814.
- Brandes JL, Kudrow D, Cady R, Tiseo PJ, Sun W, Sikes CR. Eletriptan in the early treatment of acute migraine: Influence of pain intensity and time of dosing. Cephalalgia. 2005;25(9):735–42.

16. **Brandes 2007a ***

- Anonymous. Sumatriptan + naproxen: Better than either alone for acute migraine? The Journal of Family Practice. 2007;56(7):536.
- Brandes JLJ, Kudrow D, Stark SR, Carroll CPO, Adelman JU, Donnell FJO, et al. Sumatriptan-naproxen for acute treatment of migraine. JAMA. 2007;297(13):1443–54.
- ClinicalTrials.gov. NCT00434083 [Internet]. 2007 [cited 2023 Jun 24]. Available from: https://clinicaltrials.gov/study/NCT00434083
- GlaxoSmithKline. Clinical Study Report: TXA112496. 2008 p. 1–5.
- Landy S, DeRossett SE, Rapoport A, Rothrock J, Ames MH, McDonald SA, et al. Two double-blind, multicenter, randomized, placebo-controlled, single-dose studies of sumatriptan/naproxen sodium in the acute treatment of migraine: Function, productivity, and satisfaction outcomes. MedGenMed Medscape General Medicine. 2007;9(2):1–15.

17. **Brandes 2007b ***

- Anonymous. Sumatriptan + naproxen: Better than either alone for acute migraine? The Journal of Family Practice. 2007;56(7):536.
- Brandes JLJ, Kudrow D, Stark SR, Carroll CPO, Adelman JU, Donnell FJO, et al. Sumatriptan-naproxen for acute treatment of migraine. JAMA. 2007;297(13):1443–54.
- ClinicalTrials.gov. NCT00433732 [Internet]. 2007 [cited 2023 Jun 24]. Available from: https://clinicaltrials.gov/study/NCT00433732
- Landy S, DeRossett SE, Rapoport A, Rothrock J, Ames MH, McDonald SA, et al. Two double-blind, multicenter, randomized, placebo-controlled, single-dose studies of sumatriptan/naproxen sodium in the acute treatment of migraine: Function, productivity, and satisfaction outcomes. MedGenMed Medscape General Medicine. 2007;9(2):1–15.

18. **Brauneis 1994 ***

- Brauneis S, Marzano M, Collini S, Occhigrossi F, Pinto G. L'impiego del sumatriptan nel trattamento dell'emicrania e della cefalea a grappolo. Confinia Cephalalgica. 1994;3(4):159–64.

19. Bussone 2000 *

- Bussone G, Manzoni GC, Cortelli P, Roncolato M, Fabbri L, Benassuti C. Efficacy and tolerability of sumatriptan in the treatment of multiple migraine attacks. Neurological Sciences. 2000;21(5):272–8.

20. Cady 2004 #

- Cady R, Elkind A, Goldstein J, Keywood C. Randomized, placebo-controlled comparison of early use of frovatriptan in a migraine attack versus dosing after the headache has become moderate or severe. Current Medical Research and Opinion. 2004 Sep;20(9):1465–72.

21. Cady 2006a *

- Cady R, Martin V, Mauskop A, Rodgers A, Hustad CM, Ramsey KE, et al. Symptoms of cutaneous sensitivity pre-treatment and post-treatment: Results from the rizatriptan TAME studies. Cephalalgia. 2007;27(9):1055–60.
- Cady R, Martin V, Mauskop A, Rodgers A, Hustad CM, Ramsey KE, et al. Efficacy of rizatriptan 10 mg administered early in a migraine attack. Headache. 2006;46(6):914–24.
- ClinicalTrials.gov. NCT00092963 [Internet]. 2004 [cited 2023 Jun 24]. Available from: https://clinicaltrials.gov/study/NCT00092963
- Martin V, Cady R, Mauskop A, Seidman LS, Rodgers A, Hustad CM, et al. Efficacy of rizatriptan for menstrual migraine in an early intervention model: A prospective subgroup analysis of the rizatriptan TAME (Treat a Migraine Early) studies. Headache. 2008;48(2):226–35.

22. Cady 2006b *

- Cady R, Martin V, Mauskop A, Rodgers A, Hustad CM, Ramsey KE, et al. Symptoms of cutaneous sensitivity pre-treatment and post-treatment: Results from the rizatriptan TAME studies. Cephalalgia. 2007;27(9):1055–60.
- Cady R, Martin V, Mauskop A, Rodgers A, Hustad CM, Ramsey KE, et al. Efficacy of rizatriptan 10 mg administered early in a migraine attack. Headache. 2006;46(6):914–24.
- ClinicalTrials.gov. NCT00092963 [Internet]. 2004 [cited 2023 Jun 24]. Available from: https://clinicaltrials.gov/study/NCT00092963
- Martin V, Cady R, Mauskop A, Seidman LS, Rodgers A, Hustad CM, et al. Efficacy of rizatriptan for menstrual migraine in an early intervention model: A prospective subgroup analysis of the rizatriptan TAME (Treat a Migraine Early) studies. Headache. 2008;48(2):226–35.

23. Cady 2009 *

- Cady RK, Martin VT, Géraud G, Rodgers A, Zhang Y, Ho AP, et al. Rizatriptan 10-mg ODT for early treatment of migraine and impact of migraine education on treatment response. Headache. 2009;49(5):687–96.
- ClinicalTrials.gov. NCT00516737 [Internet]. 2007 [cited 2023 Jun 24]. Available from: https://clinicaltrials.gov/study/NCT00516737
- European Union Clinical Trials Registry. 2007-003361-40 [Internet]. 2008 [cited 2023 Jun 24]. Available from: https://www.clinicaltrialsregister.eu/ctr-search/trial/2007-003361-40/DE
- WHO ITCRP. EUCTR2007-003361-40-DE [Internet]. 2008 [cited 2023 Jun 24]. Available from: https://trialsearch.who.int/Trial2.aspx?TrialID=EUCTR2007-003361-40-DE

24. Carpay 2004 *

- Barbanti P, Carpay JA, Kwong WJ, Ahmad F, Boswell D. Effects of a fast disintegrating/rapid release oral formulation of sumatriptan on functional ability in patients with migraine. Current Medical Research and Opinion. 2004;20(12):2021–9.

- Carpay J, Schoenen J, Ahmad F, Kinrade F, Boswell D. Efficacy and tolerability of sumatriptan tablets in a fast-disintegrating, rapid-release formulation for the acute treatment of migraine: Results of a multicenter, randomized, placebo-controlled study. Clinical Therapeutics. 2004;26(2):214–23.
- GlaxoSmithKline. Clinical Study Report: SUM30046. 2005 p. 1–5.
- GlaxoSmithKline. Clinical Study Report: SUM30046 (full report; document code: GM2003/00460/01). 2005.

25. Chung 2006 #

- Chung CS, Park KY, Moon HS, Joo SA, Song KS, Kim JA. Almotriptan for acute treatment of migraine: a randomized, double-blind, placebo-controlled study in Korea. Cephalalgia : an international journal of headache. 2006;26(11):1392–1392.

26. Codispoti 2001 *

- Codispoti JR, Prior MJ, Fu M, Harte CM, Nelson EB. Efficacy of nonprescription doses of ibuprofen for treating migraine headache. A randomized controlled trial. Headache. 2001;41(7):665–79.
- US Food and Drug Administration. Drug approval package: Ibuprofen / Motrin Migraine (19-012) - Medical Review. 1999 p. 1–248.
- US Food and Drug Administration. Drug approval package: Ibuprofen / Motrin Migraine (19-012) - Statistical Review. 1999 p. 1–20.

27. Croop 2019 *

- Biohaven Pharmaceuticals. Clinical Protocol (version 4.0): BHV3000-303. 2018 p. 1–81.
- Biohaven Pharmaceuticals. Statistical Analysis Plan (version 2.0): BHV3000-303. 2018.
- ClinicalTrials.gov. NCT03461757 [Internet]. 2018 [cited 2023 Jun 24]. Available from: https://clinicaltrials.gov/study/NCT03461757
- Croop R, Goadsby PJ, Stock DA, Conway CM, Forshaw M, Stock EG, et al. Efficacy, safety, and tolerability of rimegepant orally disintegrating tablet for the acute treatment of migraine: a randomised, phase 3, double-blind, placebo-controlled trial. The Lancet. 2019;394(10200):737–45.
- European Medicines Agency. Assessment Report: Rimegepant / Vydura. 2022 p. 1–135.
- McCarthy L. Commentary. Annals of Internal Medicine. 2019;171(10):JC58–9.
- US Food and Drug Administration. Drug approval package: Rimegepant / Nurtec-ODT (212728Orig1s000) - Medical Review. 2020 p. 1–118.
- US Food and Drug Administration. Drug approval package: Rimegepant / Nurtec-ODT (212728Orig1s000) - Statistical Review. 2020 p. 1–42 and 1–21.
- US Food and Drug Administration. Drug approval package: Rimegepant / Nurtec-ODT (212728Orig1s000) - Summary Review. 2020 p. 1–28.

28. CTRI/2010/091/001157 2010 #

- WHO ITCRP. CTRI/2010/091/001157 [Internet]. 2011 [cited 2023 Jun 24]. Available from: https://trialsearch.who.int/Trial2.aspx?TrialID=CTRI/2010/091/001157

29. CTRI/2023/001/048905 2023 #

- WHO ITCRP. CTRI/2023/01/048905 [Internet]. 2023 [cited 2023 Jun 24]. Available from: https://trialsearch.who.int/Trial2.aspx?TrialID=CTRI/2023/01/048905

30. Cutler 1995 *

- Cutler N, Mushet GR, Davis R, Clements B, Whitcher L. Oral sumatriptan for the acute treatment of migraine: Evaluation of three dosage strengths. Neurology. 1995;45(suppl 7):S5–9.

- Sramek JJ, Hussey EK, Clements B, Cutler NR. Oral sumatriptan pharmacokinetics in the migraine state. Clinical Drug Investigation. 1999;17(2):137–44.

31. **Dahlöf 1993 #**

- Dahlöf C, Björkman R. Diclofenac-K (50 and 100 mg) and placebo in the acute treatment of migraine. Cephalalgia. 1993;13:117–23.

32. **Dahlöf 1998 ***

- Dahlöf C, Diener HC, Goadsby PJ, Massiou H, Olesen J, Schoenen J, et al. Zolmitriptan, a 5-HT1B/1D recceptor agonist for the acute oral treatment of migraine: a multicentre, dose-range finding study. European Journal of Neurology. 1998;5(6):535–43.
- US Food and Drug Administration. Drug approval package: Zolmitriptan (20-768) - Clinical Review. 1997 p. 1–85.
- US Food and Drug Administration. Drug approval package: Zolmitriptan (20-768) - Statistical Review. 1997 p. 1–39.

33. **Dahlöf 2001 ***

- Dahlöf C, Tfelt-Hansen P, Massiou H, Fazekas A. Dose finding, placebo-controlled study of oral almotriptan in the acute treatment of migraine. Neurology. 2001;57(10):1811–7.
- US Food and Drug Administration. Drug approval package: Almotriptan/Axert (21-001) - Medical Review. 2000 p. 1–97.
- US Food and Drug Administration. Drug approval package: Almotriptan/Axert (21-001) - Statistical Review. 2000.

34. **Dahlöf 2009 ***

- Dahlöf CGH, Hauge AW, Olesen J. Efficacy and safety of tonabersat, a gap-junction modulator, in the acute treatment of migraine: A double-blind, parallel-group, randomized study. Cephalalgia. 2009;29(SUPPL. 2):7–16.

35. **Dib 2002 #**

- Dib M, Massiou H, Weber M, Henry P, Garcia-Acosta S, Bousser MG, et al. Efficacy of oral ketoprofen in acute migraine A double-blind randomized clinical trial. Neurology. 2002;58:1660–5.
- Dib M, Massiou H, Weber M, Henry P, Garcia-Acosta S, Bousser MG. Efficacy of oral ketoprofen in acute migraine: a double-blind randomized clinical trial. Headache: The Journal of Head and Face Pain. 2003 Mar;43(3):299.

36. **Diener 2002 ***

- Diener H, Jansen J, Reches A, Pascual J, Pitei D, Steiner T. Efficacy, tolerability and safety of oral eletriptan and ergotamine plus caffeine (Cafergot) in the acute treatment of migraine: a multicentre, randomised, double-blind, placebo-controlled comparison. Headache: the journal of head and face pain. 2003;43(3):301.
- Diener HC, Jansen JP, Reches A, Pascual J, Pitei D, Steiner TJ. Efficacy, tolerability and safety of oral eletriptan and ergotamine plus caffeine (Cafergot®) in the acute treatment of migraine: A multicentre, randomised, double-blind, placebo-controlled comparison. European Neurology. 2002;47(2):99–107.
- US Food and Drug Administration. Drug approval package: Eletriptan / Relpax (21-016) - Medical Review. 1999 p. 1–121.

37. **Diener 2004a ***

- Eikermann A, Diener HC, Voelker M, Gessne U. Placebo-controlled comparison of efficacy of effervescent acetylsalicylic acid (ASA) and sumatriptan on accompanying symptoms of migraine attacks. Cephalalgia. 2003;23(7):704–5.
- M. Diener G; V HC; Eikermann, A; Gessner, U; Gobel, H; Haag, G; Lange, R; May, A; Muller Schwefe. Efficacy of 1,000 mg effervescent acetylsalicylic acid and sumatriptan in treating associated migraine symptoms. Eur Neurol. 2004;52(1):50–6.

38. Diener 2004b #

- Diener HC, Bussone G, Liano HD, Eikermann A, Englert R, Floeter T, et al. Placebo-controlled comparison of effervescent acetylsalicylic acid, sumatriptan and ibuprofen in the treatment of migraine attacks. Cephalalgia. 2004 Nov;24(11):947–54.
- Diener HC, Voelker M, Group ES. Placebo-controlled comparison of effervescent Acetylsalicylic Acid (ASA), sumatriptan and ibuprofen in the treatment of migraine attacks. Cephalalgia. 2003;23:692.

39. Diener 2005 *

- Diener HC, Montagna P, Gács G, Lyczak P, Schumann G, Zöller B, et al. Efficacy and tolerability of diclofenac potassium sachets in migraine: A randomized, double-blind, cross-over study in comparison with diclofenac potassium tablets and placebo. Cephalalgia. 2006;26(5):537–47.
- Diener HC, Motagna P, Gács G, Lyczak P, Schumann G, Zoeller B, et al. Efficacy of Diclofenac-Potassium Sachets in migraine: a randomized, double blind, cross-over study with Diclofenac-Potassium Tablets and placebo. Cephalalgia. 2005;25:917.
- Novartis. Clinical Study Report: CCAT458C2301; A double-blind, double-dummy, randomized, multi-center, cross-over study to assess the efficacy and tolerability of single doses of CAT458 sachets (50 mg diclofenac-K powder for oral solution) as an acute treatment for. 2004 p. 1–17.
- US Food and Drug Administration. Drug approval package: Diclofenac potassium / Cambia (22-165) - Medical Review. 2008 p. 1–84.
- US Food and Drug Administration. Drug approval package: Diclofenac potassium / Cambia (22-165) - Statistical Review. 2008 p. 1–33.

40. Diener 2011 *

- ClinicalTrials.gov. NCT00751803 [Internet]. 2008 [cited 2023 Jun 24]. Available from: https://clinicaltrials.gov/study/NCT00751803
- Diener HC, Barbanti P, Dahlöf C, Reuter U, Habeck J, Podhorna J. BI 44370 TA, an oral CGRP antagonist for the treatment of acute migraine attacks: Results from a phase II study. Cephalalgia. 2011;31(5):573–84.
- European Union Clinical Trials Registry. 2008-000079-31 [Internet]. 2008 [cited 2023 Jun 24]. Available from: https://www.clinicaltrialsregister.eu/ctr-search/trial/2008-000079-31/FR
- Ingelheim B. Clinical Study Synopsis: BI Trial No. 1246.4. 2009 p. 1–7.
- Ingelheim B. This clinical study synopsis is provided in line with Boehringer Ingelheim's 1321.3 [Internet]. 2017. Available from: https://trials.boehringer-ingelheim.com/public/trial_results_documents/1321/1321.3_13213c127239651pdf.pdf#page=1
- WHO ITCRP. EUCTR2008-000079-31-DE [Internet]. 2008 [cited 2023 Jun 24]. Available from: https://trialsearch.who.int/Trial2.aspx?TrialID=EUCTR2008-000079-31-DE

41. Dodick 2019 *

- Allergan. Protocol and Statistical Analysis Plan: UBR-MD-01. 2018.
- Allergan. Statistical Analysis Plan: UBR-MD-01 (NCT02828020). 2018.
- ClinicalTrials.gov. NCT02828020 [Internet]. 2018 [cited 2023 Jun 24]. Available from: https://www.clinicaltrials.gov/study/NCT02828020

- Dodick DW, Lipton RB, Ailani J, Lu K, Lakkis H, Finnegan M, et al. Efficacy, safety, and tolerability of ubrogepant for the acute treatment of migraine: a single-attack phase 3 study ACHIEVE I. Canadian Journal of Neurological Sciences / Journal Canadien des Sciences Neurologiques. 2019;Suppl. 1:S9–10.
- Dodick DW, Lipton RB, Ailani J, Lu K, Finnegan M, Trugman JM, et al. Supplementary M. Vol. 381, New England Journal of Medicine. 2019.
- Dodick DW, Lipton RB, Ailani J, Lu K, Finnegan M, Trugman JM, et al. Ubrogepant for the Treatment of Migraine. New England Journal of Medicine. 2019;381(23):2230–41.
- US Food and Drug Administration. Drug approval package: Ubrogepant / AGN 241668; MK-1602 (211765Orig1s000) - Clinical Review. 2019 p. 1–119.
- US Food and Drug Administration. Drug approval package: Ubrogepant / AGN 241668; MK-1602 (211765Orig1s000) - Statistical Review. 2019 p. 1–5.
- US Food and Drug Administration. Drug approval package: Ubrogepant / AGN 241668; MK-1602 (211765Orig1s000) - Summary Review. 2019 p. 1–27.

42. Dowson 2002a *

- Dowson AJ, Massiou H, Laínez JM, Cabarrocas X. Almotriptan is an effective and well-tolerated treatment for migraine pain: Results of a randomized, double-blind, placebo-controlled clinical trial. Cephalalgia. 2002;22(6):453–61.
- US Food and Drug Administration. Drug approval package: Almotriptan/Axert (21-001) - Medical Review. 2000 p. 1–97.
- US Food and Drug Administration. Drug approval package: Almotriptan/Axert (21-001) - Statistical Review. 2000.

43. Dowson 2002b *

- Astrazeneca. Clinical Study Report: An International, Randomized, Placebo-controlled, Double-blind Trial to Evaluate the Efficacy and Tolerability of ZOMIG^{TM} 2.5 mg (Orally Dispersible Tablet) in the Acute Treatment of Adult Patients with Migraine (311CIL/0107). 2000.
- Dowson AJ, MacGregor EA, Purdy RA, Becker WJ, Green J, Levy SL. Zolmitriptan orally disintegrating tablet is effective in the acute treatment of migraine. Cephalalgia. 2002;22(2):101–6.
- US Food and Drug Administration. Drug approval package: Zolmitriptan orally disintegrating tablets / Zomig - ZMT (21-231) - Clinical Review. 2001 p. 1–17.
- US Food and Drug Administration. Drug approval package: Zolmitriptan orally disintegrating tablets / Zomig - ZMT (21-231) - Statistical Review. 2001 p. 1–12.

44. Dowson 2005 #

- Dowson AJ, Massiou H, Aurora SK. Managing migraine headaches experienced by patients who self-report with menstrually related migraine: A prospective, placebo-controlled study with oral sumatriptan. Journal of Headache and Pain. 2005 Apr;6(2):81–7.

45. Eletriptan Steering Committee in Japan 2002 *

- Eletriptan Steering Commitee in Japan. Efficacy and safety of eletriptan 20 mg, 40 mg and 80 mg in Japanese migraineurs. Cephalalgia. 2002;22(6):416–23.

46. Ensink 1991 *

- The Oral Sumatriptan International Multiple-Dose Study Group. Evaluation of a Multiple-Dose Regimen of Oral Sumatriptan for the Acute Treatment of Migraine. Eur Neurol. 1991;31(5):306–13.

47. EUCTR2006-000785-36 2008 #

- European Union Clinical Trials Registry. 2006-000785-36 [Internet]. 2007 [cited 2023 Jun 24]. Available from: https://www.clinicaltrialsregister.eu/ctr-search/trial/2006-000785-36/DE
- The Menarini Group. Synopsis of Clinical Study Report: EUCTR2006-000785-36. 2010.
- WHO ITCRP. EUCTR2006-00785-36-IT [Internet]. 2007 [cited 2023 Jun 24]. Available from: https://trialsearch.who.int/Trial2.aspx?TrialID=EUCTR2006-000785-36-FI

48. EUCTR2006-000805-42 2008 #

- European Union Clinical Trials Registry. 2006-000805-42 [Internet]. 2007 [cited 2023 Jun 24]. Available from: https://www.clinicaltrialsregister.eu/ctr-search/trial/2006-000805-42/DK
- The Menarini Group. Synopsis of Clinical Study Report: EUCTR2006-000805-42. 2014 p. 1–14.
- WHO ITCRP. EUCTR2006-000805-42-IT [Internet]. 2007 [cited 2023 Jun 24]. Available from: https://trialsearch.who.int/Trial2.aspx?TrialID=EUCTR2006-000805-42-IE

49. Freitag 2007 *

- ClinicalTrials.gov. NCT00250458 [Internet]. 2005 [cited 2023 Jun 24]. Available from: https://clinicaltrials.gov/study/NCT00250458
- 2.
- Freitag F, Taylor FR, Hamid MA, Rodgers A, Hustad CM, Ramsey KE, et al. Elimination of migraine-associated nausea in patients treated with rizatriptan orally disintegrating tablet (ODT): A randomized, double-blind, placebo-controlled study. Headache. 2008;48(3):368–77.

50. Freitag 2008 *

- Freitag F, Diamond M, Diamond S, Skobieranda F, Janssen I. Placebo controlled study to evaluate the efficacy of rizatriptan with acetaminophen for the treatment of migraine. In: Cephalalgia: Abstracts of the XIII Congress of the International Headache Society/13th IHC 2007. 2007. p. 575–759.
- Freitag F, Diamond M, Diamond S, Janssen I, Rodgers A, Skobieranda F. Efficacy and tolerability of coadministration of rizatriptan and acetaminophen vs rizatriptan or acetaminophen alone for acute migraine treatment. Headache. 2008;48(6):921–30.

51. Färkkila 2003 *

- ClinicalTrials.gov. NCT01989936 [Internet]. 2013 [cited 2023 Jun 24]. Available from: https://www.clinicaltrials.gov/study/NCT01989936
- Färkkilä M, Olesen J, Dahlöf C, Stovner LJ, Bruggen JPT, Rasmussen S, et al. Eletriptan for the treatment of migraine in patients with previous poor response or tolerance to oral sumatriptan. Cephalalgia. 2003;23(6):463–71.

52. Färkkila 2012 *

- ClinicalTrials.gov. NCT00883051 [Internet]. 2009 [cited 2023 Jun 24]. Available from: https://clinicaltrials.gov/study/NCT00883051
- CoLucid Pharmaceuticals Inc. Synopsis version 1.0: COL-144, COL MIG-202. 2010 p. 3–13.
- European Medicines Agency. Assessment Report: Lasmiditan / Raywow. 2022 p. 1–141.
- Färkkilä M, Diener HC, Géraud G, Láinez M, Schoenen J, Harner N, et al. Efficacy and tolerability of lasmiditan, an oral 5-HT 1F receptor agonist, for the acute treatment of migraine: A phase 2 randomised, placebo-controlled, parallel-group, dose-ranging study. The Lancet Neurology. 2012;11(5):405–13.
- US Food and Drug Administration. Drug approval package: Lasmiditan / Reywow (211280Orig1s000) - Clinical Review. 2019.
- US Food and Drug Administration. Drug approval package: Lasmiditan / Reywow (211280Orig1s000) - Statistical Review. 2019 p. 1–33.

53. Gallagher 2001 *

- Becker WJ. Zolmitriptan Versus Sumatriptan Comparison Trial. Headache. 2019;41(3):321–2.
- Gallagher RM, Dennish G, Spierings ELH, Chitra R. A comparative trial of zolmitriptan and sumatriptan for the acute oral treatment of migraine. Headache. 2000;40(2):119–28.

54. Garcia-Ramos 2003 *

- Garcia-Ramos G, MacGregor EA, Hilliard B, Bordini CA, Leston J, Hettiarachchi J. Comparative efficacy of eletriptan vs. naratriptan in the acute treatment of migraine. Cephalalgia: an international journal of headache. 2003 Nov;23(9):869–76.

55. Geraud 2000 *

- Diener HC, Dowson AJ, Ferrari M, Nappi G, Tfelt-Hansen P, Group on behalf of the S. Unbalanced randomization infleunces placebo response: scientific versus ethical issues around the use of placebo in migraine trials. Cephalalgia. 1999;19:699–700.
- Geraud G, Olesen J, Pfaffenrath V, Tfelt-Hansen P, Zupping R, Diener HC, et al. Comparison of the efficacy of zolmitriptan and sumatriptan: Issues in migraine trial design. Cephalalgia. 2000;20(1):30–8.
- US Food and Drug Administration. Drug approval package: Zolmitriptan (20-768) - Clinical Review. 1997 p. 1–85.
- US Food and Drug Administration. Drug approval package: Zolmitriptan (20-768) - Statistical Review. 1997 p. 1–39.

56. Gijsmant 1997 *

- Ferrari MD, Gijsman H, Block GA, Kramer MS, Matzura-Wolfe D, Teall J, et al. Double-blind, placebo-controlled, dose-finding study of rizatriptan (MK-462) in acute migraine. Functional Neurology. 1996;2/3(11):149.
- Gijsmant H, Kramer MS, Sargent J, Tuchman M, Maczura-Wolfe D, Polis A, et al. Double-blind, placebo-controlled, dose-finding study of rizatriptan (MK-462) in the acute treatment of migraine. Cephalalgia. 1997;17:647–51.
- Santanello NC, Polis AB, Hartmaier SL, Kramer MS, Block GA, Silberstein SD. Improvement in migraine-specific quality of life in a clinical trial of rizatriptan. Cephalalgia. 1997;17(8):867–72.
- US Food and Drug Administration. Drug approval package: Rizatriptan / Maxalt (20-864) - Clinical Review. 1998 p. 1–149.
- US Food and Drug Administration. Drug approval package: Rizatriptan / Maxalt (20-864) - Letter and label. 1998.
- US Food and Drug Administration. Drug approval package: Rizatriptan / Maxalt (20-864) - Medical Review. 1998 p. 1–6 and 1–20.

57. Goadsby 1991 #

- GlaxoSmithKline. Clinical Study Report: S2BT16. 2005 p. 1–5.
- Goadsby PJ, Zagami AS, Anthony M, Lance JW, Donnan GA, Bladin PF, et al. Oral sumatriptan in acute migraine. The Lancet. 1991 Sep;338(8770):782–3.

58. Goadsby 2000 *

- Goadsby PJ, Ferrari MD, Stovner LJ, Senard JM, Jackson NC, Poole PH. Eletriptan in acute migraine: A double-blind, placebo-controlled comparison to sumatriptan. Neurology. 2000;55(5):736.
- Jackson NC. Clinical measures of efficacy, safety and tolerability for the acute treatment of migraine: a comparison of eletriptan (20-80mg), sumatriptan (100mg) and placebo. Neurology. 1998;50(4 Suppl 4):A376.

- Spierings ELH. Eletriptan in acute migraine: A double-blind, placebo-controlled comparison to sumatriptan. Neurology. 2000;55(5).
- US Food and Drug Administration. Drug approval package: Eletriptan / Relpax (21-016) - Medical Review. 1999 p. 1–121.

59. Goadsby 2007 *

- Allais G, Acuto G, Cabarrocas X, Esbri R, Benedetto C, Bussone G. Efficacy and tolerability of almotriptan versus zolmitriptan for the acute treatment of menstrual migraine. Neurological Sciences. 2006;27(SUPPL. 2).
- Goadsby PJ, Massiou H, Pascual J, Diener HC, Dahlöf CGH, Mateos V, et al. Almotriptan and zolmitriptan in the acute treatment of migraine. Acta Neurologica Scandinavica. 2007;115(1):34–40.

60. Goadsby 2008 *

- European Union Clinical Trials Registry. 2004-005285-20 [Internet]. 2006 [cited 2023 Jun 24]. Available from: https://www.clinicaltrialsregister.eu/ctr-search/trial/2004-005285-20/DE
- Goadsby PJ. The 'Act when Mild' (AwM) Study: A step forward in our understanding of early treatment in acute migraine. Cephalalgia. 2008;28(SUPPL. 2):36–41.
- Goadsby PJ, Zanchin G, Geraud G, Klippel ND, Diaz-Insa S, Gobel H, et al. Early vs. non-early intervention in acute migraine - 'Act when Mild (AwM)'. A double-blind, placebo-controlled trial of almotriptan. Cephalalgia. 2008;28(4):383–91.
- Prodesfarma SAA. Clinical Study Report: Treatment of Acute Migraine when Pain is Mild versus when Pain is Moderate to Severe: an Almotriptan Parallel, Placebo Controlled Clinical Trial. "Act when mild?" 2007.
- WHO ITCRP. EUCTR2004-005285-20-GB [Internet]. 2005 [cited 2023 Jun 24]. Available from: https://trialsearch.who.int/Trial2.aspx?TrialID=EUCTR2004-005285-20-GB

61. Goadsby 2019 *

- ClinicalTrials.gov. NCT02605174 [Internet]. 2007 [cited 2023 Jun 24]. Available from: https://clinicaltrials.gov/study/NCT02605174
- Eli Lilly, Company. Synopsis: COL MIG-302. 2017 p. 2–8.
- European Medicines Agency. Assessment Report: Lasmiditan / Raywow. 2022 p. 1–141.
- Goadsby PJ, Wietecha LA, Dennehy EB, Kuca B, Case MG, Aurora SK, et al. Phase 3 randomized, placebo-controlled, double-blind study of lasmiditan for acute treatment of migraine. Brain. 2019;142(7):1894–904.
- US Food and Drug Administration. Drug approval package: Lasmiditan / Reywow (211280Orig1s000) - Clinical Review. 2019.
- US Food and Drug Administration. Drug approval package: Lasmiditan / Reywow (211280Orig1s000) - Statistical Review. 2019 p. 1–33.

62. Goldstein 1998 *

- Goldstein J, Ryan R, Jiang K, Getson A, Norman B, Block GA, et al. Crossover comparison of rizatriptan 5 mg and 10 mg versus sumatriptan 25 mg and 50 mg in migraine. Headache. 1998;38(10):737–47.
- Norman BA, Block GA, Jiang K, Ahrens S. Two-period crossover comparison of rizatriptan 5 mg and 10 mg to sumatriptan 25 mg and 50 mg for the acute treatment of migraine. Neurology. 1998;50(4):341.

63. Goldstein 2005 *

- Goldstein J, Silberstein SD, Saper JR, Elkind AH, Smith TR, Gallagher RM, et al. Acetaminophen, aspirin, and caffeine versus sumatriptan succinate in the early treatment of migraine: Results from the ASSET trial. Headache. 2005;45(8):973–82.

64. Goldstein 2006 *

- Goldstein J, Silberstein SD, Saper JR, Ryan RE, Lipton RB. Acetaminophen, aspirin, and caffeine in combination versus ibuprofen for acute migraine: Results from a multicenter, double-blind, randomized, parallel-group, single-dose, placebo-controlled study. Headache. 2006;46(3):444–53.

65. Gomez-Mancilla 2014 *

- ClinicalTrials.gov. NCT00892203 [Internet]. 2009 [cited 2023 Jun 24]. Available from: https://www.clinicaltrials.gov/study/NCT00892203
- European Union Clinical Trials Registry. 2008-005392-10 [Internet]. 2008 [cited 2023 Jun 24]. Available from: https://www.clinicaltrialsregister.eu/ctr-search/trial/2008-005392-10/ES
- Gomez-Mancilla B, Brand R, Jürgens TP, Göbel H, Sommer C, Straube A, et al. Randomized, multicenter trial to assess the efficacy, safety and tolerability of a single dose of a novel AMPA receptor antagonist BGG492 for the treatment of acute migraine attacks. Cephalalgia. 2014;34(2):103–13.
- Novartis. Clinical Study Report: BGG492. 2012 p. 1–6.
- WHO ITCRP. EUCTR2008-005392-10 [Internet]. 2012 [cited 2023 Jun 24]. Available from: https://trialsearch.who.int/Trial2.aspx?TrialID=EUCTR2008-005392-10-DE

66. Gruffyd-Jones 2001 *

- Gruffyd-Jones K, Kies B, Middleton A, Mulder LJMM, Røsjø, Millson DS. Zolmitriptan versus sumatriptan for the acute oral treatment of migraine: A randomized, double-blind, international study. European Journal of Neurology. 2001;8(3):237–45.
- Zeneca Pharmaceticals. Clinical Study Report: A Randomised, Double-Blind, Parallel Group Multicentre Trial to Compare the Efficacy and Safety of Zolmitriptan 2.5 mg, Zolmitriptan 5.0 mg and Sumatriptan 50 mg in the Treatment of Migraine Headache (311CIL/0070 [ZEUS]) (6-Attack Analysis). 1999 p. 1–5.

67. Göbel 2000 #

- GlaxoSmithKline. Clinical Study Report: S2WB3011. 2005 p. 1–4.
- Göbel H, Winter P, Boswell D, Crisp A, Becher W, Hange T, et al. Comparison of naratriptan and sumatriptan in recurrence-prone migraine patients. Clinical Therapeutics. 2000;22(8):981–9.
- US Food and Drug Administration. Drug approval package: Naratriptan / Amerge (20-763) - Medical Review. 1997.
- US Food and Drug Administration. Drug approval package: Naratriptan / Amerge (20-763) - Statistical Review. 1997.

68. Göbel 2004 *

- Göbel H, Heinze A, Niederberger U, Witt T, Zumbroich V. Efficacy of phenazone in the treatment of acute migraine attacks: A double-blind, placebo-controlled, randomized study. Cephalalgia. 2004;24(10):888–93.

69. Havanka 2000 *

- Dahlöf C, Winter P, Whitehouse H, Hassani H. Randomized, double-blind, placebo-controlled comparison of oral naratriptan and oral sumatriptan in the acute treatment of migraine. Neurology. 1997;48(3, supplement 2):85–6.
- GlaxoSmithKline. Clinical Study Report: S2WB2004. 2005 p. 1–8.

- Havanka H, Dahlöf C, Pop PHM, Diener HC, Winter P, Whitehouse H, et al. Efficacy of naratriptan tablets in the acute treatment of migraine: A dose-ranging study. Clinical Therapeutics. 2000;22(8):970–80.
- Saiers J, Jones M, Kane K, Thaventhiran L, O'Quinn S. Naratriptan tablets 2.5 mg exhibit prolonged action and are well-tolerated in non-severe migraine attacks: data from a comparator study with sumatriptan. European Journal of Neurology. 1999;6(suppl 3):105–6.
- US Food and Drug Administration. Drug approval package: Naratriptan / Amerge (20-763) - Medical Review. 1997.
- US Food and Drug Administration. Drug approval package: Naratriptan / Amerge (20-763) - Statistical Review. 1997.

70. Ho 2008a *

- ClinicalTrials.gov. NCT00246337 [Internet]. 2005 [cited 2023 Jun 24]. Available from: https://clinicaltrials.gov/study/NCT00246337
- Ho TW, Mannix LK, Fan X, Assaid C, Furtek C, Jones CJ, et al. Randomized controlled trial of an oral CGRP receptor antagonist, MK-0974, in acute treatment of migraine. Neurology. 2008;70(16):1304–12.

71. Ho 2008b *

- ClinicalTrials.gov. NCT00442936 [Internet]. 2007 [cited 2023 Jun 24]. Available from: https://clinicaltrials.gov/study/NCT00442936
- European Union Clinical Trials Registry. 2006-004257-14 [Internet]. 2006. Available from: https://www.clinicaltrialsregister.eu/ctr-search/trial/2006-004257-14/DK
- Ho TW, Ferrari MD, Dodick DW, Galet V, Kost J, Fan X, et al. Efficacy and tolerability of MK-0974 (telcagepant), a new oral antagonist of calcitonin gene-related peptide receptor, compared with zolmitriptan for acute migraine: a randomised, placebo-controlled, parallel-treatment trial. The Lancet. 2008;372(9656):2115–23.
- WHO ITCRP. EUCTR2006-004257-14-DK [Internet]. 2006 [cited 2023 Jun 24]. Available from: https://trialsearch.who.int/Trial2.aspx?TrialID=EUCTR2006-004257-14-DK

72. Jelinski 2006 *

- Becker WJ, Christie S, Ahmad FF, Pryse-Phillips W, SD S. Pain free efficacy of Sumatriptan in the treatment of migraine at the first sign of pain: prospective, double- blind, placebo-controlled, Canadian multicenter study of Sumatriptan 50 mg and 100 mg vs. placebo. Cephalalgia. 2003;23:692.
- GlaxoSmithKline. Clinical Study Report: SUM40291. 2005 p. 1–4.
- Jelinski SE, Becker WJ, Christie SN, Ahmad FF, Pryse-Phillips W, Simpson SD. Pain Free Efficacy of Sumatriptan in the Early Treatment of Migraine. Canadian Journal of Neurological Sciences. 2006;33:73–9.

73. Kaniecki 2006 *

- GlaxoSmithKline. Clinical Study Report: SUM40312 (full report; document code: RM2003/00456/00). 2004.
- GlaxoSmithKline. Clinical Study Report: SUM40312. 2005 p. 1–6.
- Kaniecki R, Ruoff G, Smith T, Barrett PS, Ames MH, Byrd S, et al. Prevalence of migraine and response to sumatriptan in patients self-reporting tension/stress headache. Current Medical Research and Opinion. 2006;22(8):1535–44.

74. Kellstein 2000 *

- Kellstein DE, Lipton RB, Geetha R, Koronkiewicz K, Evans FT, Stewart WF, et al. Evaluation of a novel solubilized formulation of ibuprofen in the treatment of migraine headache: A randomized, double-blind, placebo-controlled, dose-ranging study. Cephalalgia. 2000;20(4):233–43.

75. Klapper 2000 *

- Allen C, Dayno J, Lines C, Mccarroll K. Rizatriptan Wafer—Sublingual vs. Placebo at the Onset of Acute Migraine. Cephalalgia. 2001;21(1):77.
- Klapper, JA C, O'Connor S. Rizatriptan Wafer—Sublingual vs. Placebo at the Onset of Acute Migraine. Cephalalgia. 2000;20(6):585–7.

76. Klapper 2004 *

- Astrazeneca. Clinical Study Report: A Multicentre, Randomised, Double-Blind, Placebo-Controlled, Parallel-Group Trial to Assess the Efficacy of Oral Zolmitriptan 2.5 mg in the Acute Treatment of Migraine During the Mild Intensity Phase of an Attack in Patients Highly. 2002 p. 1–6.
- Klapper J, Lucas C, Røsjø, Charlesworth B. Benefits of treating highly disabled migraine patients with zolmitriptan while pain is mild. Cephalalgia. 2004;24(11):918–24.

77. Klassen 1997 *

- Elkind A, Laurenza A, Austin R, Asgharnejad M. Efficacy and tolerability of naratriptan tablets in the treatment of migraine: Results of a double-blind, placebo-controlled, parallel-group trial. Neuropharmacology. 1997;S262.
- GlaxoSmithKline. Clinical Study Report: S2WA3001. 2005 p. 1–7.
- Klassen A, Elkind A, Asgharnejad M, Webster C, Laurenza A. Naratriptan is Effective and Well Tolerated in the Acute Treatment of Migraine. Results of a Double-Blind, Placebo-Controlled, Parallel-Group Study. Headache. 1997;37:640–5.
- Klassen A, Webster C, Laurenza A, Austin R, Asgharnejad M. Naratriptan Tablets are Effective and Well-Tolerated in the Acute Treatment of Migraine: Results of a Double-Blind, Placebo-Controlled, Parallel-Group Trial. Neurology. 1997;48(3):66–7.
- US Food and Drug Administration. Drug approval package: Naratriptan / Amerge (20-763) - Medical Review. 1997.
- US Food and Drug Administration. Drug approval package: Naratriptan / Amerge (20-763) - Statistical Review. 1997.

78. Kolodny 2004 *

- Kolodny A, Polis A, Battisti WP, Johnson-Pratt L, Skobieranda F. Comparison of rizatriptan 5 mg and 10 mg tablets and sumatriptan 25 mg and 50 mg tablets. Cephalalgia. 2004;24(7):540–6.

79. Kramer 1998 *

- ClinicalTrials.gov. NCT00899379 [Internet]. 2009 [cited 2023 Jun 24]. Available from: https://clinicaltrials.gov/study/NCT00899379
- Dasbach EJ, Carides GW, Gerth WC, Santanello NC, Pigeon JG, Kramer MS. Work and productivity loss in the rizatriptan multiple attack study. Cephalalgia. 2000;20:830–4.
- Kramer MS, Matzura-Wolfe D, Polis A, Getson A, Amaraneni PG, Solbach MP, et al. A placebo-controlled crossover study of rizatriptan in the treatment of multiple migraine attacks. Neurology. 1998;51(3):773–81.
- MS K, Matzura-Wolfe D, Getson A, Polis A, Reines SA. Rizatriptan (MK-0462) for the acute treatment of migraine and migraine recurrence. Neurology. 1997;48(3):68.
- US Food and Drug Administration. Drug approval package: Rizatriptan / Maxalt (20-864) - Clinical Review. 1998 p. 1–149.

- US Food and Drug Administration. Drug approval package: Rizatriptan / Maxalt (20-864) - Letter and label. 1998.
- US Food and Drug Administration. Drug approval package: Rizatriptan / Maxalt (20-864) - Medical Review. 1998 p. 1–6 and 1–20.

80. Kuca 2018 *

- ClinicalTrials.gov. NCT02439320 [Internet]. 2019 [cited 2023 Jun 24]. Available from: https://clinicaltrials.gov/study/NCT02439320
- CoLucid Pharmaceuticals Inc. Synopsis: COL MIG-301. 2017 p. 2–8.
- European Medicines Agency. Assessment Report: Lasmiditan / Raywow. 2022 p. 1–141.
- Kuca B, Silberstein SD, Wietecha L, Berg PH, Dozier G, Lipton RB. Lasmiditan is an effective acute treatment for migraine: A phase 3 randomized study. Neurology. 2018;91(24):E2222–32.
- Kuca B, Wietecha L, Berg P, Aurora S, Dehenny E. PAINWeek Abstract Book 2017. Postgraduate Medicine. 2017;129(sup1):1–85.
- US Food and Drug Administration. Drug approval package: Lasmiditan / Reywow (211280Orig1s000) - Clinical Review. 2019.
- US Food and Drug Administration. Drug approval package: Lasmiditan / Reywow (211280Orig1s000) - Statistical Review. 2019 p. 1–33.

81. Kudrow 2005 *

- Kudrow D, Thomas HM, Ruoff G, Ishkanian G, Sands G, Le VH, et al. Valdecoxib for treatment of a single, acute, moderate to severe migraine headache. Headache. 2005;45(9):1151–62.

82. Landy 2004 *

- Dowson A, Landy S, Kwong J, Ames M, Richardson M. Randomized, double-blind, placebo-controlled parallel-group evaluation of patient satisfaction with oral sumatriptan administered in mild phase during acute treatment of Menstrually Associated Migraine (MAM). Cephalalgia. 2003;23:698.
- GlaxoSmithKline. Clinical Study Report: SUM40282 (full report; document code: GM2002/00022/00). 2002.
- GlaxoSmithKline. Clinical Study Report: SUM40282. 2005 p. 1–4.
- Landy S, Savani N, Shackelford S, Loftus J, Jones M. Efficacy and tolerability of sumatriptan tablets administered during the mild-pain phase of menstrually associated migraine. International Journal of Clinical Practice. 2004;58(10):913–9.

83. Lange 2000 *

- Lange R, Schwarz JA, Hohn M. Acetylsalicylic acid effervescent 1000 mg (Aspirin®) in acute migraine attacks; a multicentre, randomized, double-blind, single-dose, placebo-controlled parallel group study. Cephalalgia. 2000;20(7):663–7.
- US Food and Drug Administration. Drug approval package: Aspirin / Extra Strength Bayer (21-317) - Medical Review. 2001 p. 1–12.

84. Lee 2001 *

- Lee S bong, Kim Y in, Choi Y bin, Chung S woo, Yang D won, Lee K soo, et al. Double-Blind Placebo-Controlled Randomized Clinical Trial of Zolmitriptan in Acute Treatment of Migraine. J Korean Neurol Assoc. 2001;19:29–35.

85. Lines 2001 *

- ClinicalTrials.gov. NCT00897104 [Internet]. 2009 [cited 2023 Jun 24]. Available from: https://clinicaltrials.gov/study/NCT00897104
- Lines CR, Vandormael K, Malbecq W. A comparison of visual analog scale and categorical ratings of headache pain in a randomized controlled clinical trial with migraine patients. Pain. 2001;93(2):185–90.
- US Food and Drug Administration. Drug approval package: Rizatriptan / Maxalt (20-864) - Clinical Review. 1998 p. 1–149.
- US Food and Drug Administration. Drug approval package: Rizatriptan / Maxalt (20-864) - Letter and label. 1998.
- US Food and Drug Administration. Drug approval package: Rizatriptan / Maxalt (20-864) - Medical Review. 1998 p. 1–6 and 1–20.

86. Lipton 2000a *

- Lipton RB, Baggish JS, Stewart WF, Codispoti JR, Fu M. Efficacy and Safety of Acetaminophen in the Treatment of Migraine. Archives of Internal Medicine. 2000;160(22):3486.

87. Lipton 2000b #

- Cady RK, Lipton RB, Hall C, Stewart WF, O'Quinn S, Gutterman D. Treatment of Mild Headache in Disabled Migraine Sufferers: Results of the Spectrum Study. Headache. 2000 Nov;40(10):792–7.
- Lipton RB, Stewart WF, Cady R, Hall C, O'Quinn S, Kuhn T, et al. Sumatriptan for the Range of Headaches in Migraine Sufferers: Results of the Spectrum Study. 2000;

88. Lipton 2005 *

- Lipton RB, Goldstein J, Baggish JS, Yataco AR, Sorrentino JV, Quiring JN. Aspirin Is Efficacious for the Treatment of Acute Migraine. 2005;
- US Food and Drug Administration. Drug approval package: Aspirin / Extra Strength Bayer (21-317) - Statistical Review. 2000 p. 1–29.
- US Food and Drug Administration. Drug approval package: Aspirin / Extra Strength Bayer (21-317) - Medical Review. 2001 p. 1–12.

89. Lipton 2010 *

- ClinicalTrials.gov. NCT00330850 [Internet]. 2006 [cited 2023 Jun 24]. Available from: https://www.clinicaltrials.gov/study/NCT00330850
- Lipton RB, Grosberg B, Singer RP, Pearlman SH, Sorrentino JV, Quiring JN, et al. Efficacy and tolerability of a new powdered formulation of diclofenac potassium for oral solution for the acute treatment of migraine: Results from the International Migraine Pain Assessment Clinical Trial (IMPACT). Cephalalgia. 2010;30(11):1336–45.
- Rothrock JF. PRO-513 for acute migraine treatment. Headache. 2007;47(10):1459.
- US Food and Drug Administration. Drug approval package: Diclofenac potassium / Cambia (22-165) - Medical Review. 2008 p. 1–84.
- US Food and Drug Administration. Drug approval package: Diclofenac potassium / Cambia (22-165) - Statistical Review. 2008 p. 1–33.

90. Lipton 2019a *

- ClinicalTrials.gov. NCT03006276 [Internet]. 2016 [cited 2023 Jun 24]. Available from: https://www.clinicaltrials.gov/study/NCT03006276
- Dr Reddy's Laboratories Ltd. Clinical Study Protocol version 2.0: NCT03006276; DFN-15-CD-007; A Multicenter, Randomized, Double-Blind, Placebo-Controlled, Efficacy, Tolerability, and Safety Study of DFN-15 in Episodic Migraine with or without Aura. 2017.

- Dr Reddy's Laboratories Ltd. Statistical Analysis Plan version 3.0: NCT03006276; DFN-15-CD-007; A Multicenter, Randomized, Double-Blind, Placebo-Controlled, Efficacy, Tolerability, and Safety Study of DFN-15 in Episodic Migraine with or without Aura. 2017 p. 1–60.
- Lipton RB, Munjal S, Brand-Schieber E, Tepper SJ, Dodick DW. Efficacy, Tolerability, and Safety of DFN-15 (Celecoxib Oral Solution, 25 mg/mL) in the Acute Treatment of Episodic Migraine: A Randomized, Double-Blind, Placebo-Controlled Study. Headache. 2020;60(1):58–70.
- Lipton RB, Munjal S, Dodick DW, Tepper SJ, Serrano D, Iaconangelo C. Acute treatment of migraine with celecoxib oral solution: Results of a randomized, placebo-controlled clinical trial. Journal of Pain Research. 2021;14:549–60.
- US Food and Drug Administration. Drug approval package: Celecoxib / Elyxyb (212157Orig1s000) - Clinical Review. 2020. p. 1–92.
- US Food and Drug Administration. Drug approval package: Celecoxib / Elyxyb (212157Orig1s000) - Statistical Review. 2020 p. 1–22.

91. Lipton 2019b *

- Allergan. Protocol: UBR-MD-02. 2018 p. 1–105.
- Allergan. Statistical Analysis Plan: UBR-MD-02 (NCT02867709). 2018.
- ClinicalTrials.gov. NCT02867709 [Internet]. 2016 [cited 2023 Jun 24]. Available from: https://www.clinicaltrials.gov/study/NCT02867709
- Lipton RB, Dodick DW, Ailani J, Lu K, Lakkis H, Finnegan M, et al. Efficacy, safety, and tolerability of ubrogepant for the acute treatment of migraine: a single-attack phase 3 study ACHIEVE II. Canadian Journal of Neurological Sciences / Journal Canadien des Sciences Neurologiques. 2019;(Suppl. 1):S16.
- Lipton RB, Dodick DW, Ailani J, Lu K, Finnegan M, Szegedi A, et al. Effect of ubrogepant vs placebo on pain and the most bothersome associated symptom in the acute treatment of migraine: The achieve ii randomized clinical trial. JAMA - Journal of the American Medical Association. 2019;322(19):1887–98.
- Lipton RB, Dodick DW, Ailani J, Lu K, Finnegan M, Szegedi A, et al. Supplementary Online Content. JAMA - Journal of the American Medical Association. 2019;322(19):1887–98.
- Trugman JM, W DD, Ailani J, Lu K, Lakkis H, Finnegan M, et al. Efficacy, Safety, and Tolerability of Ubrogepant for the Acute Treatment of Migraine: Results From a Single-Attack Phase 3 Study, ACHIEVE II (S38.008). Neurology. 2019;92(15 Supplement).
- US Food and Drug Administration. Drug approval package: Ubrogepant / AGN 241668; MK-1602 (211765Orig1s000) - Clinical Review. 2019 p. 1–119.
- US Food and Drug Administration. Drug approval package: Ubrogepant / AGN 241668; MK-1602 (211765Orig1s000) - Statistical Review. 2019 p. 1–5.
- US Food and Drug Administration. Drug approval package: Ubrogepant / AGN 241668; MK-1602 (211765Orig1s000) - Summary Review. 2019 p. 1–27.

92. Lipton 2019c *

- Biohaven Pharmaceuticals. Clinical Protocol (version 1.0) and Statistical Analysis Plan (version 1.0): BHV3000-302. 2017.
- Biohaven Pharmaceuticals. Clinical Protocol (version 4.0): BHV3000-302. 2018.
- Biohaven Pharmaceuticals. Statistical analysis plan (version 2.0): BHV3000-302. 2018.
- ClinicalTrials.gov. NCT03237845 [Internet]. 2017 [cited 2023 Jun 24]. Available from: https://clinicaltrials.gov/study/NCT03237845
- European Medicines Agency. Assessment Report: Rimegepant / Vydura. 2022 p. 1–135.
- Lipton RB, Croop R, Stock EG, Stock DA, Morris BA, Frost M, et al. Rimegepant, an Oral Calcitonin Gene–Related Peptide Receptor Antagonist, for Migraine. New England Journal of Medicine. 2019;381(2):142–9.
- Lipton RB, Croop R, Stock EG, Stock DA, Morris BA, Frost M, et al. Supplementary Appendix. New England Journal of Medicine. 2019;381(2):142–9.

- US Food and Drug Administration. Drug approval package: Rimegepant / Nurtec-ODT (212728Orig1s000) - Medical Review. 2020 p. 1–118.
- US Food and Drug Administration. Drug approval package: Rimegepant / Nurtec-ODT (212728Orig1s000) - Statistical Review. 2020 p. 1–42 and 1–21.
- US Food and Drug Administration. Drug approval package: Rimegepant / Nurtec-ODT (212728Orig1s000) - Summary Review. 2020 p. 1–28.

93. Lipton 2021 *

- ClinicalTrials.gov. NCT03009019 [Internet]. 2017 [cited 2023 Jun 24]. Available from: https://www.clinicaltrials.gov/study/NCT03009019
- Dr Reddy's Laboratories Ltd. Clinical Study Protocol version 2.0: NCT03009019; DFN-15-CD-006; A Multicenter, Randomized, Double-Blind, Placebo-Controlled, Efficacy, Tolerability, and Safety Study of DFN-15 in Episodic Migraine with or without Aura. 2017 p. 1–61.
- Dr Reddy's Laboratories Ltd. Statistical Analysis Plan version 3.0: NCT03009019; DFN-15-CD-006; A Multicenter, Randomized, Double-Blind, Placebo-Controlled, Efficacy, Tolerability, and Safety Study of DFN-15 in Episodic Migraine with or without Aura. 2017 p. 1–60.
- Lipton RB, Munjal S, Tepper SJ, Iaconangelo C, Serrano D. A multicenter, randomized, double-blind, placebo-controlled study of the efficacy, tolerability, and safety of celecoxib oral solution (Elyxyb) in acute treatment of episodic migraine with or without aura. Journal of Pain Research. 2021;14(June):2529–42.
- US Food and Drug Administration. Drug approval package: Celecoxib / Elyxyb (212157Orig1s000) - Clinical Review. 2020 p. 1–92.
- US Food and Drug Administration. Drug approval package: Celecoxib / Elyxyb (212157Orig1s000) - Statistical Review. 2020 p. 1–22.

94. Loder 2005 *

- Loder E, Freitag FG, Adelman J, Pearlman S, Abu-Shakra S. Pain-free rates with zolmitriptan 2.5 mg ODT in the acute treatment of migraine: Results of a large double-blind placebo-controlled trial. Current Medical Research and Opinion. 2005;21(3):381–9.

95. MacGregor 2002 #

- MacGregor EA, Dowson A, Davies PTG. Mouth-dispersible aspirin in the treatment of migraine: A placebo-controlled study. Headache. 2002;42(4):249–55.

96. Mannix 2007a *

- ClinicalTrials.gov. NCT00111709 [Internet]. 2005 [cited 2023 Jun 24]. Available from: https://clinicaltrials.gov/study/NCT00111709
- Mannix LK, Loder E, Nett R, Mueller L, Rodgers A, Hustad CM, et al. Rizatriptan for the acute treatment of ICHD-II proposed menstrual migraine: Two prospective, randomized, placebo-controlled, double-blind studies. Cephalalgia. 2007;27(5):414–21.
- Nett R, Mannix LK, Mueller L, Rodgers A, Hustad CM, Skobieranda F, et al. Rizatriptan efficacy in ICHD-II pure menstrual migraine and menstrually related migraine. Headache. 2008;48(8):1194–201.

97. Mannix 2007b *

- ClinicalTrials.gov. NCT00111722 [Internet]. 2005 [cited 2023 Jun 24]. Available from: https://clinicaltrials.gov/study/NCT00111722
- Mannix LK, Loder E, Nett R, Mueller L, Rodgers A, Hustad CM, et al. Rizatriptan for the acute treatment of ICHD-II proposed menstrual migraine: Two prospective, randomized, placebo-controlled, double-blind studies. Cephalalgia. 2007;27(5):414–21.

- Nett R, Mannix LK, Mueller L, Rodgers A, Hustad CM, Skobieranda F, et al. Rizatriptan efficacy in ICHD-II pure menstrual migraine and menstrually related migraine. Headache. 2008;48(8):1194–201.

98. Marcus 2014 *

- ClinicalTrials.gov. NCT01430442 [Internet]. 2011 [cited 2023 Jun 24]. Available from: https://clinicaltrials.gov/search?term=NCT01430442
- European Medicines Agency. Assessment Report: Rimegepant / Vydura. 2022 p. 1–135.
- Marcus R, Goadsby PJ, Dodick D, Stock D, Manos G, Fischer TZ. BMS-927711 for the acute treatment of migraine: A double-blind, randomized, placebo controlled, dose-ranging trial. Cephalalgia. 2014;34(2):114–25.

99. Massiou 2005 *

- GlaxoSmithKline. Clinical Study Report: S2W40031. 2005 p. 1–4.
- Massiou H, Jamin C, Hinzelin G, Bidaut-Mazel C. Efficacy of oral naratriptan in the treatment of menstrually related migraine. European Journal of Neurology. 2005;12(10):774–81.

100. Mathew 1997 *

- GlaxoSmithKline. Clinical Study Report: S2WA3003. 2005 p. 1–7.
- Mathew NT, Peykamian M, Laurenza A, Austin R, Asgharnejad M. Efficacy and tolerability of naratriptan tablets in the treatment of migraine: Results from a double-blind, placebo-controlled, crossover trial. Headache. 1997;S35.
- Mathew NT, Asgharnejad M, Peykamian M, Laurenza A. Naratriptan is effective and well tolerated in the acute treatment of migraine. Neurology. 1997;49:1485–90.
- US Food and Drug Administration. Drug approval package: Naratriptan / Amerge (20-763) - Medical Review. 1997.
- US Food and Drug Administration. Drug approval package: Naratriptan / Amerge (20-763) - Statistical Review. 1997.

101. Mathew 2003 *

- Mathew NT, Schoenen J, Winner P, Muirhead N, Sikes CR. Comparative Efficacy of Eletriptan 40 mg Versus Sumatriptan 100 mg. Headache. 2003 Mar;43(3):214–22.
- US Food and Drug Administration. Drug approval package: Eletriptan / Relpax (21-016) - Medical Review. 1999 p. 1–121.

102. Mathew 2004 #

- Mathew NT, Kailasam J, Meadors L. Early Treatment of Migraine With Rizatriptan : A Placebo-Controlled Study. 2004;44:669–73.

103. Mathew 2007 *

- ClinicalTrials.gov. NCT00210509 [Internet]. 2005 [cited 2023 Jun 24]. Available from: https://www.clinicaltrials.gov/study/NCT00210509
- Freitag F, Smith T, Mathew N, Rupnow M, Greenberg S, Mao L, et al. Effect of early intervention with almotriptan vs placebo on migraine-associated functional disability: Results from the AEGIS trial. Headache. 2008;48(3):341–54.
- Mathew NT, Finlayson G, Smith TR, Cady RK, Adelman J, Mao L, et al. Early intervention with almotriptan: Results of the AEGIS trial (AXERT® early migraine intervention study). Headache. 2007;47(2):189–98.

104. Mitsikostas 2010 #

- Mitsikostas DD, Vikelis M, Kodounis A, Zaglis D, Xifaras M, Doitsini S, et al. Migraine recurrence is not associated with depressive or anxiety symptoms. Results of a randomized controlled trial. Cephalalgia. 2010 Jun;30(6):690–5.

105. Misra 2010 *

- Misra M, Sharma T, Kalra J, Goel D, Dhasmana DC. Comparative efficacy and tolerability of sumatriptan, ergotamine, naproxen and rizatriptan in moderate to severe acute attack of migraine. JK Science. 2010;12(4):175–9.

106. MOMENTUM 2019 *

- ClinicalTrials.gov. NCT03896009 [Internet]. 2019 [cited 2023 Jun 24]. Available from: https://www.clinicaltrials.gov/study/NCT03896009
- O'Gorman C, Jones A, Lipton RB, Tepper SJ, Tabuteau H. Comparative efficacy of AXS-07 (MoSEIC Meloxicam/Rizatriptan) in the Acute Treatment of Migraine: Results from the MOMENTUM Phase 3, Randomized, Double-blind, Active- and Placebo-Controlled Trial. Annals of neurology: 145th Annual Meeting American Neurological Association. 2020;88(suppl 25):S1–280.
- O'Gorman C, Jones A, Lipton RB, Tepper SJ, Tabuteau H. Presentation: Efficacy and safety of AXS-07 (MoSEIC™ meloxicam/rizatriptan) in the acute treatment of migraine: results from the momentum phase 3, randomized, double-blind, active- and placebo- controlled trial. American Academy of Neurology Science Highlights: Emerging Science Presentation [Internet]. 2020; Available from: https://www.axsome.com/publications/Axsome_AXS-07-Presentation_AAN-Emerging-Science-2020.pdf
- O'Gorman C, Jones A, Lipton RB, Tepper SJ, Tabuteau H. Comparative efficacy of AXS-07 (MoSEIC Meloxicam/Rizatriptan) versus rizatriptan in the acute treatment of migraine. Headache. 2021;61(SUPPL 1):115-116.

107. Moon 2010 *

- Moon HS, Chu MK, Park JW, Oh K, Chung JM, Cho YJ, et al. Frovatriptan is effective and well tolerated in Korean migraineurs: A double-blind, randomized, placebo-controlled trial. Journal of Clinical Neurology (Korea). 2010;6(1):27–32.

108. Munjal 2017 *

- Bennett A, Munjal S. A multi-center, randomized, placebo-controlled, double-blind, crossover study evaluating DFN-15, a liquid celecoxib formulation, for the acute treatment of migraine with or without aura in adults. Headache: the journal of head and face pain. 2017;57(Supplement 3):131.
- ClinicalTrials.gov. NCT02472418 [Internet]. 2015 [cited 2023 Jun 24]. Available from: https://www.clinicaltrials.gov/study/NCT02472418
- Munjal S, Bennett A. Efficacy and safety of DFN-15, an oral liquid formulation of celecoxib, in adults with migraine: A multicenter, randomized, placebo-controlled, double-blind, crossover study. Neuropsychiatric Disease and Treatment. 2017;13:2797–802.
- S. Bennett A; M. A multi-center, randomized, placebo-controlled, double-blind, crossover study evaluating DFN-15, a liquid celecoxib formulation, for the acute treatment of migraine with or without aura in adults. Neurology. 2019;92(15 Supplement 1).
- US Food and Drug Administration. Drug approval package: Celecoxib / Elyxyb (212157Orig1s000) - Clinical Review. 2020 p. 1–92.

109. Myllylä 1998 *

- Myllylä VV, Havanka H, Herrala L, Kangasniemi P, Rautakorpi I, Turkka J, et al. Tolfenamic acid rapid release versus sumatriptan in the acute treatment of migraine: Comparable effect in a double-blind, randomized, controlled, parallel-group study. Headache. 1998;38(3):201–7.

110. Nappi 1994 *

- Centonze V, Polito MB, Bari MD, Fabbri L, Cassiano MA, Bassi A, et al. Valutazione dell'efficacia di sumatriptan nella terapia orale dell'attacco di emicrania. La Clinica Terapeutica. 1995;146:721–8.
- GlaxoSmithKline. Clinical Study Report: S2CT34. 2006 p. 1–5.
- Nappi G, Sicuteri F, Byrne M, Roncolato M, Zerbini O. Oral sumatriptan compared with placebo in the acute treatment of migraine. Journal of Neurology. 1994;241(3):138–44.

111. NCT00471952 2008 #

- ClinicalTrials.gov. NCT00471952 [Internet]. 2007 [cited 2023 Jun 24]. Available from: https://clinicaltrials.gov/study/NCT00471952

112. NCT00821483 2008 #

- ClinicalTrials.gov. NCT00821483 [Internet]. 2009 [cited 2023 Jun 24]. Available from: https://clinicaltrials.gov/study/NCT00821483

113. NCT00920686 2010 *

- ClinicalTrials.gov. NCT00920686 [Internet]. 2009 [cited 2023 Jun 24]. Available from: https://clinicaltrials.gov/study/NCT00920686

114. NCT01248468 2011 *

- ClinicalTrials.gov. NCT01248468 [Internet]. 2010 [cited 2023 Jun 24]. Available from: https://www.clinicaltrials.gov/study/NCT01248468

115. NCT01657370 2012 *

- ClinicalTrials.gov. NCT01657370 [Internet]. 2012 [cited 2023 Jun 24]. Available from: https://www.clinicaltrials.gov/study/NCT01657370
- US Food and Drug Administration. Drug approval package: Ubrogepant / AGN 241668; MK-1602 (211765Orig1s000) - Clinical Review. 2019 p. 1–119.
- US Food and Drug Administration. Drug approval package: Ubrogepant / AGN 241668; MK-1602 (211765Orig1s000) - Statistical Review. 2019 p. 1–5.
- US Food and Drug Administration. Drug approval package: Ubrogepant / AGN 241668; MK-1602 (211765Orig1s000) - Summary Review. 2019 p. 1–27.

116. NCT01986270 1998 *

- ClinicalTrials.gov. NCT01986270 [Internet]. 2013 [cited 2023 Jun 24]. Available from: https://www.clinicaltrials.gov/study/NCT01986270
- US Food and Drug Administration. Drug approval package: Eletriptan / Relpax (21-016) - Medical Review. 1999 p. 1–121.

117. NCT03235479 2018 *

- Biohaven Pharmaceuticals. Clinical Protocol (version 4.0): BHV-3000-301. 2018 p. 1–81.
- Biohaven Pharmaceuticals. Statistical analysis plan (version 2.0): BHV3000-301. 2018 p. 1–64.

- ClinicalTrials.gov. NCT03235479 [Internet]. 2017 [cited 2023 Jun 24]. Available from: https://clinicaltrials.gov/study/NCT03235479
- European Medicines Agency. Assessment Report: Rimegepant / Vydura. 2022 p. 1–135.
- US Food and Drug Administration. Drug approval package: Rimegepant / Nurtec-ODT (212728Orig1s000) - Medical Review. 2020 p. 1–118.
- US Food and Drug Administration. Drug approval package: Rimegepant / Nurtec-ODT (212728Orig1s000) - Statistical Review. 2020 p. 1–42 and 1–21.
- US Food and Drug Administration. Drug approval package: Rimegepant / Nurtec-ODT (212728Orig1s000) - Summary Review. 2020 p. 1–28.

118. NCT04218162 2020 #

- ClinicalTrials.gov. NCT04218162 [Internet]. 2020 [cited 2023 Jun 24]. Available from: https://clinicaltrials.gov/study/NCT04218162

119. NCT04384367 2022 #

- ClinicalTrials.gov. NCT04384367 [Internet]. 2020 [cited 2023 Jun 24]. Available from: https://clinicaltrials.gov/study/NCT04384367

120. NCT05399459 2022 #

- ClinicalTrials.gov. https://clinicaltrials.gov/show/NCT05399459. 2022. NCT05399459.

121. NCT05509400 2022 #

- ClinicalTrials.gov. NCT05509400 [Internet]. 2022 [cited 2023 Jun 24]. Available from: https://clinicaltrials.gov/study/NCT05509400
- European Union Clinical Trials Registry. 2022-001175-14 [Internet]. 2022 [cited 2023 Jun 24]. Available from: https://www.clinicaltrialsregister.eu/ctr-search/trial/2022-001175-14/SE

122. NCT05685225 2023 #

- ClinicalTrials.gov. NCT05685225 [Internet]. 2023 [cited 2023 Jun 24]. Available from: https://clinicaltrials.gov/study/NCT05685225

123. Nett 2003 *

- Dowson A, Landy S, Kwong J, Ames M, Richardson M. Randomized, double-blind, placebo-controlled parallel-group evaluation of patient satisfaction with oral sumatriptan administered in mild phase during acute treatment of Menstrually Associated Migraine (MAM). Cephalalgia. 2003;23:698.
- GlaxoSmithKline. Clinical Study Report: SUM40285 (full repor; document code: RM2001/00174/00). 2002.
- GlaxoSmithKline. Clinical Study Report: SUM40285. 2005 p. 1–4.
- Landy S, Savani N, Shackelford S, Loftus J, Jones M. Efficacy and tolerability of sumatriptan tablets administered during the mild-pain phase of menstrually associated migraine. International Journal of Clinical Practice. 2004;58(10):913–9.
- Nett R, Landy S, Shackelford S, Richardson MS, Ames M, Lener M, et al. Sumatriptan taken during the mild pain phase was more effective than Placebo for relief of menstrual migraine. Evidence-based Obstetrics and Gynecology. 2004;6(3):139–40.
- Nett R, Landy S, Shackelford S, Richardson MS, Ames M, Lener M. Pain-free efficacy after treatment with sumatriptan in the mild pain phase of menstrually associated migraine. Obstetrics and Gynecology. 2003;102(4):835–42.

124. NTR33 2006 #

- ISRCTN. ISRCTN18216584 [Internet]. 2005 [cited 2023 Jun 24]. Available from: https://www.isrctn.com/ISRCTN57387771
- WHO ITCRP. ISRCTN18216584 [Internet]. 2005 [cited 2023 Jun 24]. Available from: https://trialsearch.who.int/Trial2.aspx?TrialID=ISRCTN18216584

125. NTR34 2006 #

- ISRCTN. ISRCTN57387771 [Internet]. 2005 [cited 2023 Jun 24]. Available from: https://www.isrctn.com/ISRCTN57387771
- WHO ITCRP. ISRCTN57387771 [Internet]. 2005 [cited 2023 Jun 24]. Available from: https://trialsearch.who.int/Trial2.aspx?TrialID=ISRCTN57387771

126. Padma 1998 #

- Padma MV, Jain S, Maheshwari MC, Misra S, A. K. S, A. K. M, et al. Efficacy and Tolerability of Oral Sumatriptan in Indian Patients with Acute Migraine; A Multicentre Study. Neurology India. 1998;46:105–8.

127. Pascual 2000a *

- Pascual J, Falk RM, Piessens F, Prusinski A, Docekal P, Robert M, et al. Consistent efficacy and tolerability of almotriptan in the acute treatment of multiple migraine attacks: Results of a large, randomized, double-blind, placebo-controlled study. Cephalalgia. 2000;20(6):588–96.
- US Food and Drug Administration. Drug approval package: Almotriptan/Axert (21-001) - Medical Review. 2000 p. 1–97.
- US Food and Drug Administration. Drug approval package: Almotriptan/Axert (21-001) - Statistical Review. 2000.

128. Pascual 2000b *

- Pascual J, Vega P, Diener HC, Allen C, Vrijens F, Patel K. Comparison of rizatriptan 10 mg vs. zolmitriptan 2.5 mg in the acute treatment of migraine. Cephalalgia. 2000;20(5):455–61.

129. Pfaffenrath 1998 *

- Pfaffenrath V, Cunin G, Sjonell G, Prendergast S. Efficacy and safety of sumatriptan tablets (25 mg, 50 mg, and 100 mg) in the acute treatment of migraine: Defining the optimum doses of oral sumatriptan. Headache. 1998;38(3):184–90.

130. Pini 1995 *

- Pini LA, Sternieri E, Fabbri L, Zerbint O, Bamfi F. High Efficacy and Low Frequency of Headache Recurrence after Oral Sumatriptan. Journal of International Medical Research. 1995;23(2):96–105.

131. Prior 2010 *

- Prior MJ, Codispoti JR, Fu M. A randomized, placebo-controlled trial of acetaminophen for treatment of migraine headache. Headache. 2010;50(5):819–33.

132. Rapoport 1997 *

- Rapoport AM, Ramadan NM, Adelman JU, Mathew NT, Elkind AH, Kudrow DB, et al. Optimizing the dose of zolmitriptan (Zomig,* 311C90) for the acute treatment of migraine: A multicenter, double-blind, placebo-controlled dose range-findings study. Neurology. 1997;49(5):1210–8.
- US Food and Drug Administration. Drug approval package: Zolmitriptan (20-768) - Clinical Review. 1997 p. 1–85.
- US Food and Drug Administration. Drug approval package: Zolmitriptan (20-768) - Statistical Review. 1997 p. 1–39.

133. Rapoport 2002 *

- Goldstein J, Keywood C. Frovatriptan for the Acute Treatment of Migraine: A Dose-Finding Study. Headache. 2002;42(4):262–4.
- Rapoport A, Ryan R, Goldstein J, Keywood C. Dose range-finding studies with frovatriptan in the acute treatment of migraine. Headache. 2002;42(SUPPL. 2):74–83.
- Ryan, R R, Keywood, C. A preliminary study of VML251 (SB209509) a novel 5HT1B/1D agonist for the treatment of acute migraine. Cephalalgia. 1997;17:418.
- US Food and Drug Administration. Drug approval package: Frovatriptan / Miguard (21-006) - Medical Review. 1999 p. 1–110.

134. Rederich 1995 #

- Rederich G, Rapoport A, Cutler N, Hazelrigg R, Jamerson B. Oral sumatriptan for the long-term treatment of migraine: clinical findings. Neurology. 1995;8(Suppl 7):15–20.

135. Ryan 2002a *

- Géraud G, Spierings ELH, Keywood C. Tolerability and safety of frovatriptan with short- and long-term use for treatment of migraine and in comparison with sumatriptan. Headache. 2002;42(SUPPL. 2):93–9.
- Ryan R, Géraud G, Goldstein J, Cady R, Keywood C. Clinical efficacy of frovatriptan: Placebo-controlled studies. Headache. 2002;42(SUPPL. 2):84–92.
- Tfelt-Hansen P. Frovatriptan and Data Publication. Headache. 2008;48(9):1382–3.
- US Food and Drug Administration. Drug approval package: Frovatriptan / Miguard (21-006) - Medical Review. 1999 p. 1–110.

136. Ryan 2002b *

- Géraud G, Spierings ELH, Keywood C. Tolerability and safety of frovatriptan with short- and long-term use for treatment of migraine and in comparison with sumatriptan. Headache. 2002;42(SUPPL. 2):93–9.
- Ryan R, Géraud G, Goldstein J, Cady R, Keywood C. Clinical efficacy of frovatriptan: Placebo-controlled studies. Headache. 2002;42(SUPPL. 2):84–92.
- Tfelt-Hansen P. Frovatriptan and Data Publication. Headache. 2008;48(9):1382–3.
- US Food and Drug Administration. Drug approval package: Frovatriptan / Miguard (21-006) - Medical Review. 1999 p. 1–110.

137. Ryan 2002c *

- Géraud G, Spierings ELH, Keywood C. Tolerability and safety of frovatriptan with short- and long-term use for treatment of migraine and in comparison with sumatriptan. Headache. 2002;42(SUPPL. 2):93–9.
- Ryan R, Géraud G, Goldstein J, Cady R, Keywood C. Clinical efficacy of frovatriptan: Placebo-controlled studies. Headache. 2002;42(SUPPL. 2):84–92.
- Tfelt-Hansen P. Frovatriptan and Data Publication. Headache. 2008;48(9):1382–3.
- US Food and Drug Administration. Drug approval package: Frovatriptan / Miguard (21-006) - Medical Review. 1999 p. 1–110.

138. S2WA4003 1998 #

- GlaxoSmithKline. Clinical Study Report: S2WA4003. 2005 p. 1–6.

139. S2WA4004 1998 #

- GlaxoSmithKline. Clinical Study Report: S2WA4004. 2005 p. 1–6.

140. S2WB3002 1996 *

- GlaxoSmithKline. Clinical Study Report: S2WB3002. 2005 p. 1–72.
- US Food and Drug Administration. Drug approval package: Naratriptan / Amerge (20-763) - Medical Review. 1997.
- US Food and Drug Administration. Drug approval package: Naratriptan / Amerge (20-763) - Statistical Review. 1997.

141. S2WB4001 1998 #

- GlaxoSmithKline. Clinical Study Report: S2WB4001. 2005 p. 1–5.

142. S2WB4003 UN #

- Ferrari MD, Goadsby PJ, Roon KI, Lipton RB, Ferrari MD. Triptans (serotonin, 5-HT 1B/1D agonists) in migraine: detailed results and methods of a meta-analysis of 53 trials.

143. S98-073 UN *

- US Food and Drug Administration. Drug approval package: Aspirin / Extra Strength Bayer (21-317) - Statistical Review. 2000 p. 1–29.
- US Food and Drug Administration. Drug approval package: Aspirin / Extra Strength Bayer (21-317) - Medical Review. 2001 p. 1–12.

144. S98-074 UN *

- US Food and Drug Administration. Drug approval package: Aspirin / Extra Strength Bayer (21-317) - Statistical Review. 2000 p. 1–29.
- US Food and Drug Administration. Drug approval package: Aspirin / Extra Strength Bayer (21-317) - Medical Review. 2001 p. 1–12.

145. Sakai 2002 *

- Sakai F, Iwata M, Tashiro K, Itoyama Y, Tsuji S, Fukuuchi Y, et al. Zolmitriptan is effective and well tolerated in Japanese patients with migraine: A dose-response study. Cephalalgia. 2002;22(5):376–83.

146. Sakai 2021 *

- ClinicalTrials.gov. NCT03962738 [Internet]. 2019 [cited 2023 Jun 24]. Available from: https://clinicaltrials.gov/study/NCT03962738
- Eli Lilly, Company. Protocol (a): H8H-JE-LAIH; RandoMized, DOuble-bliNd, PlacebO-coNtrolled Trial Of Lasmiditan in a Single Migraine Attack in Japanese Patients SuFfering from Migraine With or WithoUt Aura – the MONONOFU study. 2019 p. 1–70.
- Eli Lilly, Company. Clinical Study Report Synopsis: H8H-JE-LAIH. 2020 p. 1–6.

- Eli Lilly, Company. Statistical analysis plan (version 2): H8H-JE-LAIH; RandoMized, DOuble-bliNd, PlacebO-coNtrolled Trial Of Lasmiditan in a Single Migraine Attack in Japanese Patients SuFfering from Migraine With or WithoUt Aura – the MONONOFU study. 2020 p. 1–56.
- European Medicines Agency. Assessment Report: Lasmiditan / Raywow. 2022 p. 1–141.
- Hirata K, Matsushita Y, Tanji Y, Khanna R, Ozeki A, Komori M. Safety profile of lasmiditan in patients with migraine in an Asian population. Expert Opinion on Drug Safety. 2022;61(5):1–12.
- Matsumori Y, Komori M, Tanji Y, Ozeki A, Sakai F. Rapid Onset and Sustained Efficacy of Lasmiditan Among Japanese Patients with Migraine: Prespecified Analyses of a Randomized Controlled Trial. Neurology and Therapy. 2022;11(4):1721–34.
- Sakai F, Takeshima T, Homma G, Tanji Y, Katagiri H, Komori M. Phase 2 randomized placebo-controlled study of lasmiditan for the acute treatment of migraine in Japanese patients. Headache. 2021;61(5):755–65.
- Sakai F, Takeshima T, Homma G, Tanji Y, Katagiri H, Komori M. Supplementary Material: Graph showing freedom from most bothersome-symptom. Headache [Internet]. 2021;61. Available from: https://www.ncbi.nlm.nih.gov/pmc/articles/PMC8252620/
- Sakai F, Takeshima T, Homma G, Tanji Y, Katagiri H, Komori M. Supplementary Material: Study Flowchart. Headache. 2021;61.
- Sakai F, Takeshima T, Homma G, Tanji Y, Katagiri H, Komori M. Supplementary Material: Supplementary Tables. Headache [Internet]. 2021;61. Available from: https://www.ncbi.nlm.nih.gov/pmc/articles/PMC8252620/
- Sakai F, Takeshima T, Homma G, Tanji Y, Katagiri H, Komori M. Supplementary Material: Visual abstract. Headache [Internet]. 2021;61. Available from: https://www.ncbi.nlm.nih.gov/pmc/articles/PMC8252620/

147. Sandrini 2002 *

- Anonymous. Medical options in acute migraine attacks: Two triptans in placebo-controlled double-blind comparison. Gynakologie fur Hausarzte. 2003;8(2):19.
- ClinicalTrials.gov. NCT01986088 [Internet]. 2013 [cited 2023 Jun 24]. Available from: https://www.clinicaltrials.gov/study/NCT01986088
- Deleu D. Eletriptan vs sumatriptan: A double-blind, placebo-controlled, multiple migraine attack study. Neurology. 2003;60(7):1221.
- Pryse-Phillips W. Oral eletriptan (40-80 mg) versus oral sumatriptan (50-100 mg) for the treatment of acute migraine in sumatriptan-naïve patients. European Journal of Neurology. 1999;6(Suppl 3):21.
- Sandrini G, Färkkilä M, Burgess G, Forster E, Haughie S. Eletriptan vs sumatriptan: A double-blind, placebo-controlled, multiple migraine attack study. Neurology. 2002;59:1210–7.
- US Food and Drug Administration. Drug approval package: Eletriptan / Relpax (21-016) - Medical Review. 1999. p. 1–121.

148. Saper 2006 *

- Saper J, Dahlof C, So Y, Tfelt-Hansen P, Malbecq W, Loeys T, et al. Rofecoxib in the acute treatment of migraine: A randomized controlled clinical trial. Headache. 2006;46(2):264–75.

149. Sargent 1995 *

- GlaxoSmithKline. Clinical Study Report: S2B216. 2005 p. 1–6.
- Sargent J, Kirchner JR, Davis R, Kirkhart B. Oral sumatriptan is effective and well tolerated for the acute treatment of migraine: Results of a multicenter study. Neurology. 1995;45(suppl 7):S10–4.

150. Savani 1999 *

- GlaxoSmithKline. Clinical Study Report: S2CM07. 2005 p. 1–8.

- Savani N, Brautaset NJ, Reunanen M, Szirmai I, Ashford EA, Hassani H, et al. A double-blind placebo-controlled study assessing the efficacy and tolerability of 50 mg sumatriptan tablets in the acute treatment of migraine. International Journal of Clinical Practice. 1999;Supplement:1–5.
- The S2BM07 Study Group. The efficacy, tolerability and safety of oral sumatriptan 50 mg in the acute treatment of migraine. European Journal of Neurology. 1996;3(Suppl 5):149.

151. Savi 2011 #

- European Union Clinical Trials Registry. 2006-002572-17 [Internet]. 2007 [cited 2023 Jun 24]. Available from: https://www.clinicaltrialsregister.eu/ctr-search/trial/2006-002572-17/IT
- Lisotto C, Guidotti M, Zava D, Savi L. Frovatriptan and rizatriptan economic EVAluation: the FREEVA study. The journal of headache and pain. 2013;14:96.
- Savi L, Omboni S, Lisotto C, Zanchin G, Ferrari MD, Zava D, et al. A double-blind, randomized, multicenter, Italian study of frovatriptan versus rizatriptan for the acute treatment of migraine. Journal of Headache and Pain. 2011;12(2):219–26.
- Savi L, Omboni S, Lisotto C, Sances G, Zanchin G, Ferrari MD, et al. Efficacy of frovatriptan in the acute treatment of menstrually-related migraine: analysis of a double-blind, randomized, multicenter, comparative study vs. Rizatriptan. Cephalalgia. 2011;77.
- The Menarini Group. Synopsis of Clinical Study Report: Lumi/06/Fro-Mig/001; EUCTR2006-002572-17. 2010 p. 1–87.
- WHO ITCRP. EUCTR2006-002572-17-IT [Internet]. 2007 [cited 2023 Jun 24]. Available from: https://trialsearch.who.int/Trial2.aspx?TrialID=EUCTR2006-002572-17-IT

152. Savi 2014 #

- Savi L, Mogavero S, Egan CG. Efficacy and pharmacokinetic activity of frovatriptan compared to rizatriptan in patients with moderate-to-severe migraine. Drug Design, Development and Therapy. 2014 Jul;8:983–92.

153. Seeburger 2012 #

- ClinicalTrials.gov. NCT00812006 [Internet]. 2008 [cited 2023 Jun 24]. Available from: https://clinicaltrials.gov/study/NCT00812006
- European Union Clinical Trials Registry. 2008-008283-26 [Internet]. 2009 [cited 2023 Jun 24]. Available from: https://www.clinicaltrialsregister.eu/ctr-search/trial/2008-008283-26/IT
- Seeburger JL, Cady RK, Winner P, MacGregor A, Valade D, Ge Y, et al. Rizatriptan for treatment of acute migraine in patients taking topiramate for migraine prophylaxis. Headache. 2012;52(1):57–67.
- WHO ITCRP. EUCTR2008-008283-26-ES [Internet]. 2009 [cited 2023 Jun 24]. Available from: https://trialsearch.who.int/Trial2.aspx?TrialID=EUCTR2008-008283-26-ES

154. Sheftell 2003 *

- ClinicalTrials.gov. NCT01978496 [Internet]. 2013 [cited 2023 Jun 24]. Available from: https://www.clinicaltrials.gov/study/NCT01978496
- Sheftell F, Ryan R, Pitman V. Efficacy, safety, and tolerability of oral eletriptan for treatment of acute migraine: A multicenter, double-blind, placebo-controlled study conducted in the United States. Headache. 2003;43(3):202–13.
- US Food and Drug Administration. Drug approval package: Eletriptan / Relpax (21-016) - Medical Review. 1999 p. 1–121.

155. Sheftell 2005a *

- Dahlöf C, Taylor FR, Mulleners W, Adelman J, Webster CJ, Few C. Sustained pain relief and sustained pain-free rates with sumatriptan fast disintegrating tablets: Results from two double-blind, placebo-controlled trials. European Journal of Neurology. 2005;12(Suppl. 2):70.
- GlaxoSmithKline. Clinical Study Report: SUM30047 (full report; document code RM2004/00176/00). 2004.
- GlaxoSmithKline. Clinical Study Report: SUM30047. 2005 p. 1–5.
- Sheftell FD, Dahlöf CGH, Brandes JL, Agosti R, Jones MW, Barrett PS. Two replicate randomized, double-blind, placebo-controlled trials of the time to onset of pain relief in the acute treatment of migraine with a fast-disintegrating/rapid-release formulation of sumatriptan tablets. Clinical therapeutics. 2005 Apr;27(4):407–17.

156. Sheftell 2005b *

- Dahlöf C, Taylor FR, Mulleners W, Adelman J, Webster CJ, Few C. Sustained pain relief and sustained pain-free rates with sumatriptan fast disintegrating tablets: Results from two double-blind, placebo-controlled trials. European Journal of Neurology. 2005;12(Suppl. 2):70.
- GlaxoSmithKline. Clinical Study Report: SUM30053. 2004 p. 1–4.
- GlaxoSmithKline. Clinical Study Report: SUM30053 (full report; document code: RM2004/00177/00). 2004.
- Sheftell FD, Dahlöf CGH, Brandes JL, Agosti R, Jones MW, Barrett PS. Two replicate randomized, double-blind, placebo-controlled trials of the time to onset of pain relief in the acute treatment of migraine with a fast-disintegrating/rapid-release formulation of sumatriptan tablets. Clinical therapeutics. 2005 Apr;27(4):407–17.

157. Smith 2005 *

- Smith TR, Sunshine A, Stark SR, Littlefield DE, Spruill SE, Alexander WJ. Sumatriptan and naproxen sodium for the acute treatment of migraine. Headache. 2005;45(8):983–91.

158. Solomon 1997 *

- Solomon GD, Cady RK, Klapper JA, Earl NL, Saper JR, Ramadan NM. Clinical efficacy and tolerability of 2.5 mg zolmitriptan for the acute treatment of migraine. Neurology. 1997;49(5):1219–25.
- US Food and Drug Administration. Drug approval package: Zolmitriptan (20-768) - Clinical Review. 1997 p. 1–85.
- US Food and Drug Administration. Drug approval package: Zolmitriptan (20-768) - Statistical Review. 1997 p. 1–39.

159. Spierings 2001 *

- Colman SS, Brod MI, Krishnamurthy A, Rowland CR, Jirgens KJ, Gomez-Mancilla B. Treatment satisfaction, functional status, and health-related quality of life of migraine patients treated with almotriptan or sumatriptan. Clin Ther. 2001;23(1):127–45.
- Spierings ELH, Gomez-Mancilla B, Grosz DE, Rowland CR, Whaley FS, Jirgens KJ. Oral almotriptan vs oral sumatriptan in the abortive treatment of migraine: A double-blind, randomized, parallel-group, optimum-dose comparison. Archives of Neurology. 2001;58(6):944–50.
- US Food and Drug Administration. Drug approval package: Almotriptan/Axert (21-001) - Medical Review. 2000 p. 1–97.

160. Spierings 2004 *

- Spierings ELH, Rapoport AM, Dodick DW, Charlesworth B. Acute treatment of migraine with zolmitriptan 5mg orally disintegrating tablet. CNS Drugs. 2004;18(15):1133–41.

161. Stark 2002 *

- Stark R, Dahlöf C, Haughie S, Hettiarachchi J. Efficacy, safety and tolerability of oral eletriptan in the acute treatment of migraine: Results of a phase III, multicentre, placebo-controlled study across three attacks. Cephalalgia. 2002;22(1):23–32.
- US Food and Drug Administration. Drug approval package: Eletriptan / Relpax (21-016) - Medical Review. 1999 p. 1–121.
- Wells NEJ, Steiner TJ. Effectiveness of eletriptan in reducing time loss caused by migraine attacks. PharmacoEconomics. 2000;18(6):557–66.

162. Steiner 2003 *

- Steiner TJ, Diener HC, MacGregor EA, Schoenen J, Muirhead N, Sikes CR. Comparative efficacy of eletriptan and zolmitriptan in the acute treatment of migraine. Cephalalgia. 2003;23:942–52.

163. Stronks 2003 #

- Stronks DL, Tulen JHM, Bussmann HBJ, Mulder LJMM, Passchier J. Effects of Naratriptan Versus Naproxen on Daily Functioning in the Acute Treatment of Migraine: A Randomized, Double-Blind, Double-Dummy, Crossover Study. Headache. 2003;43:845–52.

164. SUM20033 2003 *

- GlaxoSmithKline. Clinical Study Report: SUM20033 (full report; document code: RM2003/00372/00). 2004.
- GlaxoSmithKline. Clinical Study Report: SUM20033. 2005 p. 1–5.

165. SUMA4016 1998 #

- GlaxoSmithKline. Clinical Study Report: SUMA4016. 2005 p. 1–5.

166. SUMA4017 1998 #

- GlaxoSmithKline. Clinical Study Report: SUMA4017. 2005 p. 1–6.

167. Tazaki 1993a *

- Tazaki Y, Sakai F, Tashiro K, Hirai S, Gotoh F, Maruyama S, et al. Clinical Evaluation of SN-308 (Sumatriptan) Tablet on Migraine -Double-blind Parallel-Group Study-. Rinsho iyaku (journal of clinical therapeutics and medicines). 1993;9(9):2147–65.

168. Tazaki 1993b #

- Tazaki Y, Sakai F, Tashiro K, Hirai S, Goto F, Maruyama S, et al. Clinical Evaluation of SN-308 (Sumatriptan) Tablet on Migraine - Dose Finding Study by Double-Blind Cross-Over Method. Rinsho iyaku (journal of clinical therapeutics and medicines). 1997;13(21):5567–94.

169. Teall 1996 *

- ClinicalTrials.gov. NCT00897949 [Internet]. 2009 [cited 2023 Jun 24]. Available from: https://clinicaltrials.gov/study/NCT00897949
- Cutler NR, Jhee SS, Majumdar AK, McLoughlin D, Brucker MJ, Carides AD, et al. Pharmacokinetics of rizatriptan tablets during and between migraine attacks. Headache. 1999;39(4):264–9.

- Teall J, Tuchman M, Cutler N, Gross M, Willoughby E, Smith B, et al. Rizatriptan (MAXALT) for the acute treatment of migraine and migraine recurrence. A placebo-controlled, outpatient study. Headache. 1998;38(4):281–7.
- US Food and Drug Administration. Drug approval package: Rizatriptan / Maxalt (20-864) - Clinical Review. 1998 p. 1–149.
- US Food and Drug Administration. Drug approval package: Rizatriptan / Maxalt (20-864) - Letter and label. 1998.
- US Food and Drug Administration. Drug approval package: Rizatriptan / Maxalt (20-864) - Medical Review. 1998 p. 1–6 and 1–20.

170. The Diclofenac-K/Sumatriptan Migraine Study 1999 #

- The Diclofenac-K/Sumatriptan Migraine Study Group. Acute treatment of migraine attacks: efficacy and safety of a nonsteroidal anti-inflammatory drug, diclofenac-potassium, in comparison to oral sumatriptan and placebo. Cephalalgia. 1999;19(4):232–40.

171. Tfelt-Hansen 1995 *

- Tfelt-Hansen P, Henry P, Mulder LJ, Scheldewaert RG, Schoenen J, Chazot G. The effectiveness of combined oral lysine acetylsalicylate and metoclopramide compared with oral sumatriptan for migraine. The Lancet. 1995;346(8980):923–6.
- Tfelt-Hansen P, Henry P, Mulder LJ, Scheldewaert RG, Schoenen J, Chazot G. Kombination af oral lysin-acetylsalicylat og metoclopramid sammenlignet med oral sumatriptan i behandlingen af migræneanfald. Ugeskrift for Læger. 1996;158(45):6435–9.
- Tfelt-Hansen P. The effectiveness of combined oral lysine acetylsalicylate and metoclopramide (MigPriv®) in the treatment of migraine attacks. Comparison with placebo and oral sumatriptan. Funct neurol. 2000;15(Suppl):196–201.

172. Tfelt-Hansen 1998 *

- ClinicalTrials.gov. NCT00898677 [Internet]. 2009 [cited 2023 Jun 24]. Available from: https://clinicaltrials.gov/study/NCT00898677
- Tfelt-Hansen P, Teall J, Rodriguez F, Giacovazzo M, Paz J, Malbecq W, et al. Oral rizatriptan versus oral sumatriptan: A direct comparative study in the acute treatment of migraine. Headache. 1998;38(10):748–55.
- US Food and Drug Administration. Drug approval package: Rizatriptan / Maxalt (20-864) - Clinical Review. 1998. p. 1–149.
- US Food and Drug Administration. Drug approval package: Rizatriptan / Maxalt (20-864) - Letter and label. 1998.
- US Food and Drug Administration. Drug approval package: Rizatriptan / Maxalt (20-864) - Medical Review. 1998. p. 1–6 and 1–20.
- Visser WH, Jiang K. Effect of rizatriptan versus sumatriptan on migraine associated symptoms. Neurology. 1998;50(Supplement 4):A375.

173. Tfelt-Hansen 2006 *

- GlaxoSmithKline. Clinical Study Report: SUM40308. 2005 p. 1–3.
- Tfelt-Hansen P, Bach FW, Daugaard D, Tsiropoulos I, Riddersholm B. Treatment with sumatriptan 50 mg in the mildphase of migraine attacks in patients withinfrequent attacks: A randomised, double-blind,placebo-controlled study. Journal of Headache and Pain. 2006;7(6):389–94.

174. The Oral Sumatriptan Dose-defining Study 1991 *

- Patten J, The Oral Sumatriptan Dose-Defining Study Group. Clinical experience with oral sumatriptan: a placebo-controlled, dose-ranging study. J Neurol. 1991;238:S62–5.
- The Oral Sumatriptan Dose-Defining Study Group. Sumatriptan–an oral dose-defining study. Eur Neurol. 1991;31(5):300–5.

175. Toledano 2021 *

- ClinicalTrials.gov. NCT03061734 [Internet]. 2017 [cited 2023 Jun 24]. Available from: https://www.clinicaltrials.gov/study/NCT03061734
- Toledano AC. Low-Dose Naltrexone/Acetaminophen Combinations and Each Component in the Acute Treatment of Migraine: Findings of a Small, Randomized, Double-Blind, and Placebo-Controlled Clinical Trial. medRxiv. 2021;20:2021.03.22.21254145.

176. Tuchman 2006 #

- Tuchman M, Hee A, Emeribe U, Silberstein S. Efficacy and Tolerability of Zolmitriptan Oral Tablet in the Acute Treatment of Menstrual Migraine: CNS Drugs. 2006;20(12):1019–26.

177. Tullo 2010 #

- Allais G, Tullo V, Benedetto C, Zava D, Omboni S, Bussone G. Efficacy of frovatriptan in the acute treatment of menstrually related migraine: analysis of a double-blind, randomized, multicenter, Italian, comparative study versus zolmitriptan. Neurol Sci. 2011;32(Suppl 1):S99–104.
- European Union Clinical Trials Registry. 2006-005764-81 [Internet]. 2007 [cited 2023 Jun 24]. Available from: https://www.clinicaltrialsregister.eu/ctr-search/trial/2006-005764-81/IT
- The Menarini Group. Synopsis of Clinical Study Report: EUCTR2006-005764-81. 2010.
- Tullo V, Allais G, Curone M, Ferrari MD, Omboni S, Benedetto C, et al. Frovatriptan versus zolmitriptan for the acute treatment of migraine with aura: a subgroup analysis of a double-blind, randomized, multicenter, Italian study. Neurol Sci. 2012;33(Suppl 1):S61–4.
- Tullo V, Allais G, Ferrari MD, Curone M, Mea E, Omboni S, et al. Frovatriptan versus zolmitriptan for the acute treatment of migraine: A double-blind, randomized, multicenter, Italian study. Neurological Sciences. 2010;31(Suppl 1):S51–4.
- WHO ITCRP. EUCTR2006-005764-81-IT [Internet]. 2007 [cited 2023 Jun 24]. Available from: https://trialsearch.who.int/Trial2.aspx?TrialID=EUCTR2006-005764-81-IT

178. Visser 1996a *

- US Food and Drug Administration. Drug approval package: Rizatriptan / Maxalt (20-864) - Clinical Review. 1998. p. 1–149.
- US Food and Drug Administration. Drug approval package: Rizatriptan / Maxalt (20-864) - Letter and label. 1998.
- US Food and Drug Administration. Drug approval package: Rizatriptan / Maxalt (20-864) - Medical Review. 1998. p. 1–6 and 1–20.
- Visser WH, Terwindt GM, Reines SA, Jiang K, Lines CR, Ferrari MD. Rizatriptan vs Sumatriptan in the Acute Treatment of Migraine. Arch Neurol. 1996;53:1132–7.

179. Visser 1996b *

- US Food and Drug Administration. Drug approval package: Zolmitriptan (20-768) - Clinical Review. 1997. p. 1–85.
- US Food and Drug Administration. Drug approval package: Zolmitriptan (20-768) - Statistical Review. 1997. p. 1–39.

- Visser WH, Klein KB, Cox RC, Jones D, Ferrari MD. 311C90, A new central and peripherally acting 5-HT1D receptor agonist in the acute oral treatment of migraine: A double-blind, placebo-controlled, dose-range finding study. Neurology. 1996;46(2):522–6.

180. Voss 2016 *

- ClinicalTrials.gov. NCT01613248 [Internet]. 2016 [cited 2023 Jun 24]. Available from: https://www.clinicaltrials.gov/study/NCT01613248
- Merck Sharp & Dohme Corp. Protocol: 006-01: A Phase IIb, Multicenter, Randomized, Double-Blind, Placebo-Controlled, Dose-Finding Study of MK-1602 in the Treatment of Acute Migriane. 2012.
- US Food and Drug Administration. Drug approval package: Ubrogepant / AGN 241668; MK-1602 (211765Orig1s000) - Clinical Review. 2019 p. 1–119.
- US Food and Drug Administration. Drug approval package: Ubrogepant / AGN 241668; MK-1602 (211765Orig1s000) - Statistical Review. 2019 p. 1–5.
- US Food and Drug Administration. Drug approval package: Ubrogepant / AGN 241668; MK-1602 (211765Orig1s000) - Summary Review. 2019 p. 1–27.
- Voss T, Lipton RB, Dodick DW, Dupre N, Ge JY, Bachman R, et al. A phase IIb randomized, double-blind, placebo-controlled trial of ubrogepant for the acute treatment of migraine. Cephalalgia. 2016;36(9):887–98.
- Voss T, Lipton RB, Dodick DW, Dupre N, Ge JY, Bachman R, et al. Supplemental Table 1. Summary of adverse events within 14 days post dose for the all subjects as treated population. Cephalalgia. 2016;36(9).

181. Wentz 2008 *

- Wentz AL, Jimenez TB, Dixon RM, Aurora SK, Gold M. A double-blind, randomized, placebo-controlled, single-dose study of the cyclooxygenase-2 inhibitor, GW406381, as a treatment for acute migraine. European Journal of Neurology. 2008;15(4):420–7.

182. Winner 2003a *

- GlaxoSmithKline. Clinical Study Report: SUM40274. 2005 p. 1–4.
- Winner P, Mannix LK, Putnam DG, McNeal S, Kwong J, O’Quinn S, et al. Pain-free results with sumatriptan taken at the first sign of migraine pain: Randomized, double-blind, placebo-controlled studies. Mayo Clinic Proceedings. 2003;78(10):1214–22.

183. Winner 2003b *

- GlaxoSmithKline. Clinical Study Report: SUM40275. 2005 p. 1–3.
- Winner P, Mannix LK, Putnam DG, McNeal S, Kwong J, O’Quinn S, et al. Pain-free results with sumatriptan taken at the first sign of migraine pain: Randomized, double-blind, placebo-controlled studies. Mayo Clinic Proceedings. 2003;78(10):1214–22.

184. Yu 2023 *

- ClinicalTrials.gov. NCT04574362 [Internet]. 2020 [cited 2023 Jun 24]. Available from: https://clinicaltrials.gov/study/NCT04574362
- Yu S, Kim BK, Guo A, Kim MH, Zhang M, Wang Z, et al. Safety and efficacy of rimegepant orally disintegrating tablet for the acute treatment of migraine in China and South Korea: a phase 3, double-blind, randomised, placebo-controlled trial. The Lancet Neurology. 2023;22(6):476–84.
- Yu S, Kim BK, Guo A, Kim MH, Zhang M, Wang Z, et al. Supplementary Appendix 1. The Lancet Neurology. 2023;22:477–85.
- Yu S, Kim BK, Guo A, Kim MH, Zhang M, Wang Z, et al. Supplementary appendix 2. The Lancet Neurology. 2023;22:477–85.

- Yu S, Kim BK, Guo A, Kim MH, Zhang M, Wang Z, et al. Supplementary appendix 3. The Lancet Neurology. 2023;22:477–85.

Appendix 5. Network plots

Network plots for each outcome
The network plots on the following pages (GitHub links) present the network of eligible comparisons for each outcome (by individual drugs and drug classes). Each drug is represented with a circle (a node) and randomised comparisons between drugs are shown with lines between the nodes. The width of the line is proportional to the number of trials comparing each pair of interventions, and the size of each node is proportional to the number of randomly assigned participants.

5.01 Efficacy – Pain freedom at 2 hours

Number of studies = 115

Number of interventions (including placebo) = 18

Number of participants = 76 375

Number of events = 16 207 (21.2%)

Individual drugs: GitHub link

Drug classes: GitHub link

5.02 Efficacy – Sustained pain freedom from 2 to 24 hours

Number of studies = 56

Number of interventions (including placebo) = 15

Number of participants = 44 098

Number of events = 6 522 (14.8%)

Individual drugs: GitHub link

Drug classes: GitHub link

5.03 Efficacy – Pain relief at 2 hours

Number of studies = 109

Number of interventions (including placebo) = 18

Number of participants = 75 870

Number of events = 34 506 (45.5%)

Individual drugs: GitHub link

Drug classes: GitHub link

5.04 Efficacy – Pain relapse within to 2 to 48 hours

Number of studies = 8

Number of interventions (including placebo) = 4

Number of participants = 12 281

Number of events = 726 (5.9%)

Individual drugs: GitHub link

Drug classes: GitHub link

5.05 Efficacy – Use of rescue medication within 2 to 24 hours

Number of studies = 64

Number of interventions (including placebo) = 15

Number of participants = 49 090

Number of events = 14 569 (29.7%)

Individual drugs: GitHub link

Drug classes: GitHub link

5.06 Serious adverse events

Number of studies = 27

Number of interventions (including placebo) = 12

Number of participants = 26 252

Number of events = 56 (0.2%)

Individual drugs: GitHub link

Drug classes: GitHub link

5.07 Adverse events – Abdominal pain

Number of studies = 31

Number of interventions (including placebo) = 14

Number of participants = 22 650

Number of events = 3 172 (14.0%)

Individual drugs: GitHub link

Drug classes: GitHub link

5.08 Adverse events – Allergic reaction

Number of studies = 6

Number of interventions (including placebo) = 4

Number of participants = 9 168

Number of events = 1 284 (14.0%)

Individual drugs: GitHub link

Drug classes: GitHub link

5.09 Adverse events – Chest pain/discomfort

Number of studies = 57

Number of interventions (including placebo) = 14

Number of participants = 42 425

Number of events = 5 200 (12.3%)

Individual drugs: GitHub link

Drug classes: GitHub link

5.10 Adverse events – Constipation

Number of studies = 4

Number of interventions (including placebo) = 3

Number of participants = 6 408

Number of events = 919 (14.3%)

Individual drugs: GitHub link

Drug classes: GitHub link

5.11 Adverse events – Diarrhoea

Number of studies = 29

Number of interventions (including placebo) = 13

Number of participants = 22 598

Number of events = 2 868 (12.7%)

Individual drugs: GitHub link

Drug classes: GitHub link

5.12 Adverse events – Dizziness

Number of studies = 82

Number of interventions (including placebo) = 17

Number of participants = 56 928

Number of events = 8 544 (15.0 %)

Individual drugs: GitHub link

Drug classes: GitHub link

5.13 Adverse events – Dry mouth

Number of studies = 49

Number of interventions (including placebo) = 16

Number of participants = 37 415

Number of events = 4 875 (13.0%)

Individual drugs: GitHub link

Drug classes: GitHub link

5.14 Adverse events – Dyspepsia

Number of studies = 28

Number of interventions (including placebo) = 11

Number of participants = 21 480

Number of events = 3 181 (14.8%)

Individual drugs: GitHub link

Drug classes: GitHub link

5.15 Adverse events – Fatigue

Number of studies = 72

Number of interventions (including placebo) = 15

Number of participants = 47 598

Number of events = 6 582 (13.8%)

Individual drugs: GitHub link

Drug classes: GitHub link

5.16 Adverse events – Gastrointestinal bleeding

Sufficient outcome data not available for network meta-analysis.

5.17 Adverse events – Hepatic toxicity

Number of studies = 17

Number of interventions (including placebo) =9

Number of participants = 17 081

Number of events = 2 495 (14.6%)

Individual drugs: GitHub link

Drug classes: GitHub link

5.18 Adverse events – Major adverse cardiovascular events

Sufficient outcome data not available for network meta-analysis.

5.19 Adverse events – Nausea

Number of studies = 18

Number of interventions (including placebo) = 78

Number of participants = 57 246

Number of events = 8 312 (14.5%)

Individual drugs: GitHub link

Drug classes: GitHub link

5.20 Adverse events – Paraesthesia

Number of studies = 62

Number of interventions (including placebo) = 15

Number of participants = 43 595

Number of events = 6 160 (14.1%)

Individual drugs: GitHub link

Drug classes: GitHub link

5.21 Adverse events – Pruritus

Number of studies = 8

Number of interventions (including placebo) = 7

Number of participants = 7 433

Number of events = 1 025 (13.8%)

Individual drugs: GitHub link

Drug classes: GitHub link

5.22 Adverse events – Sedation

Number of studies = 73

Number of interventions (including placebo) = 15

Number of observations = 53 535

Number of events = 7 491 (14.0%)

Individual drugs: GitHub link

Drug classes: GitHub link

5.23 Adverse events – Serotonergic syndrome

Sufficient outcome data not available for network meta-analysis.

5.24 Adverse events – Vertigo

Number of studies = 19

Number of interventions (including placebo) = 9

Number of participants = 17 959

Number of events = 2 499 (13.2%)

Individual drugs: GitHub link

Drug classes: GitHub link

5.25 Adverse events – Vomiting

Number of studies = 43

Number of interventions (including placebo) = 17

Number of participants = 30 608

Number of events = 4 191 (13.7%)

Individual drugs: GitHub link

Drug classes: GitHub link

Appendix 6. Pairwise meta-analyses

Results of the pairwise meta-analysis are available for each outcome (GitHub links)

6.01 Efficacy – Pain freedom at 2 hours

- GitHub link – Individual drugs
- GitHub link – Drug classes

6.02 Efficacy – Sustained pain freedom from 2 to 24 hour

- GitHub link – Individual drugs
- GitHub link – Drug classes

6.03 Efficacy – Pain relief at 2 hours

- GitHub link – Individual drugs
- GitHub link – Drug classes

6.04 Efficacy – Pain relapse within to 2 to 48 hours

- GitHub link – Individual drugs
- GitHub link – Drug classes

6.05 Efficacy – Use of rescue medication within 2 to 24 hours

- GitHub link – Individual drugs
- GitHub link – Drug classes

6.06 Serious adverse events

- Individual drugs – Sufficient outcome data not available for meta-analysis.
- GitHub link – Drug classes

6.07 Adverse events – Abdominal pain

- GitHub link – Individual drugs
- GitHub link – Drug classes

6.08 Adverse events – Allergic reaction

- GitHub link – Individual drugs
- GitHub link – Drug classes

6.09 Adverse events – Chest pain/discomfort

- GitHub link – Individual drugs
- GitHub link – Drug classes

6.10 Adverse events – Constipation

- GitHub link – Individual drugs
- GitHub link – Drug classes

6.11 Adverse events – Diarrhoea

- GitHub link – Individual drugs
- GitHub link – Drug classes

6.12 Adverse events – Dizziness

- GitHub link – Individual drugs
- GitHub link – Drug classes

6.13 Adverse events – Dry mouth

- GitHub link – Individual drugs
- GitHub link – Drug classes

6.14 Adverse events – Dyspepsia

- GitHub link – Individual drugs
- GitHub link – Drug classes

6.15 Adverse events – Fatigue

- GitHub link – Individual drugs
- GitHub link – Drug classes

6.16 Adverse events – Gastrointestinal bleeding

Sufficient outcome data not available for meta-analysis.

6.17 Adverse events – Hepatic toxicity

- GitHub link – Individual drugs
- GitHub link – Drug classes

6.18 Adverse events – Major adverse cardiovascular events

Sufficient outcome data not available for meta-analysis.

6.19 Adverse events – Nausea

- GitHub link – Individual drugs
- GitHub link – Drug classes

6.20 Adverse events – Paraesthesia

- GitHub link – Individual drugs
- GitHub link – Drug classes

6.21 Adverse events – Pruritus

- GitHub link – Individual drugs
- GitHub link – Drug classes

6.22 Adverse events – Sedation

- GitHub link – Individual drugs
- GitHub link – Drug classes

6.23 Adverse events – Serotonergic syndrome

Sufficient outcome data not available for meta-analysis.

6.24 Adverse events – Vertigo

- GitHub link – Individual drugs
- GitHub link – Drug classes

6.25 Adverse events – Vomiting

- GitHub link – Individual drugs
- GitHub link – Drug classes

Appendix 7. Forest plots (by individual drugs and drug class)

Network meta-analysis: Inverse variance, random effects.

7.01 Efficacy – Pain freedom at 2 hours

Sumatriptan/triptan as reference:

- *Individual drugs:* GitHub link
- *Drug classes:* GitHub link

Ibuprofen/NSAID as reference:

- *Individual drugs:* GitHub link
- *Drug classes:* GitHub link

Placebo as reference:

- *Individual drugs:* GitHub link
- *Drug classes:* GitHub link

7.02 Efficacy – Sustained pain freedom from 2 to 24 hours

Sumatriptan/triptan as reference:

- *Individual drugs:* GitHub link
- *Drug classes:* GitHub link

Ibuprofen/NSAID as reference:

- *Individual drugs:* GitHub link
- *Drug classes:* GitHub link

Placebo as reference:

- *Individual drugs:* GitHub link
- *Drug classes:* GitHub link

7.03 Efficacy – Pain relief at 2 hours

Sumatriptan/triptan as reference:

- *Individual drugs:* GitHub link
- *Drug classes:* GitHub link

Ibuprofen/NSAID as reference:

- *Individual drugs:* GitHub link
- *Drug classes:* GitHub link

Placebo as reference:

- *Individual drugs:* GitHub link
- *Drug classes:* GitHub link

7.04 Efficacy – Pain relapse within 2 to 48 hours

Sumatriptan/triptan as reference:

- *Individual drugs:* GitHub link
- *Drug classes:* GitHub link

Ibuprofen/NSAID as reference:

- Sufficient outcome data not available for analysis.

Placebo as reference:

- *Individual drugs:* GitHub link
- *Drug classes:* GitHub link

7.05 Efficacy – Use of rescue medication within 2 to 24 hours

Sumatriptan/triptan as reference:

- *Individual drugs:* GitHub link
- *Drug classes:* GitHub link

Ibuprofen/NSAID as reference:

- *Individual drugs:* GitHub link
- *Drug classes:* GitHub link

Placebo as reference:

- *Individual drugs:* GitHub link
- *Drug classes:* GitHub link

7.06 Serious adverse events

- Sufficient outcome data not available for analysis.

7.07 Adverse events – Abdominal pain

Sumatriptan/triptan as reference:

- *Individual drugs:* GitHub link
- *Drug classes:* GitHub link

Ibuprofen/NSAID as reference:

- *Individual drugs:* GitHub link
- *Drug classes:* GitHub link

Placebo as reference:

- *Individual drugs:* GitHub link
- *Drug classes:* GitHub link

7.08 Adverse events – Allergic reaction

Sumatriptan/triptan as reference:

- *Individual drugs:* GitHub link
- *Drug classes:* GitHub link

Ibuprofen/NSAID as reference:

- Sufficient outcome data not available for analysis.

Placebo as reference:

- *Individual drugs:* GitHub link
- *Drug classes:* GitHub link

7.09 Adverse events – Chest pain/discomfort

Sumatriptan/triptan as reference:

- *Individual drugs:* GitHub link
- *Drug classes:* GitHub link

Ibuprofen/NSAID as reference:

- *Individual drugs:* Sufficient outcome data not available for analysis.
- *Drug classes:* GitHub link

Placebo as reference:

- *Individual drugs:* GitHub link
- *Drug classes:* GitHub link

7.10 Adverse events – Constipation

Sumatriptan/triptan as reference:

- *Individual drugs:* GitHub link
- *Drug classes:* GitHub link

Ibuprofen/NSAID as reference:

- Sufficient outcome data not available for analysis.

Placebo as reference:

- *Individual drugs:* GitHub link
- *Drug classes:* GitHub link

7.11 Adverse events – Diarrhoea

Sumatriptan/triptan as reference:

- *Individual drugs:* GitHub link
- *Drug classes:* GitHub link

Ibuprofen/NSAID as reference:

- *Individual drugs:* Sufficient outcome data not available for analysis.
- *Drug classes:* GitHub link

Placebo as reference:

- *Individual drugs:* GitHub link
- *Drug classes:* GitHub link

7.12 Adverse events – Dizziness

Sumatriptan/triptan as reference:

- *Individual drugs:* GitHub link
- *Drug classes:* GitHub link

Ibuprofen/NSAID as reference:

- *Individual drugs:* GitHub link
- *Drug classes:* GitHub link

Placebo as reference:

- *Individual drugs:* GitHub link
- *Drug classes:* GitHub link

7.13 Adverse events – Dry mouth

Sumatriptan/triptan as reference:

- *Individual drugs:* GitHub link
- *Drug classes:* GitHub link

Ibuprofen/NSAID as reference:

- *Individual drugs:* GitHub link
- *Drug classes:* GitHub link

Placebo as reference:

- *Individual drugs:* GitHub link
- *Drug classes:* GitHub link

7.14 Adverse events – Dyspepsia

Sumatriptan/triptan as reference:

- *Individual drugs:* GitHub link
- *Drug classes:* GitHub link

Ibuprofen/NSAID as reference:

- *Individual drugs:* GitHub link
- *Drug classes:* GitHub link

Placebo as reference:

- *Individual drugs:* GitHub link
- *Drug classes:* GitHub link

7.15 Adverse events – Fatigue

Sumatriptan/triptan as reference:

- *Individual drugs:* GitHub link
- *Drug classes:* GitHub link

Ibuprofen/NSAID as reference:

- *Individual drugs:* Sufficient outcome data not available for analysis.
- *Drug classes:* GitHub link

Placebo as reference:

- *Individual drugs:* GitHub link
- *Drug classes:* GitHub link

7.16 Adverse events – Gastrointestinal bleeding

- Sufficient outcome data not available for analysis.

7.17 Adverse events – Hepatic toxicity

Sumatriptan/triptan as reference:

- *Individual drugs:* GitHub link
- *Drug classes:* GitHub link

Ibuprofen/NSAID as reference:

- *Individual drugs:* Sufficient outcome data not available for analysis.
- *Drug classes:* GitHub link

Placebo as reference:

- *Individual drugs:* GitHub link
- *Drug classes:* GitHub link

7.18 Adverse events – Major adverse cardiovascular events

- Sufficient outcome data not available for analysis.

7.19 Adverse events – Nausea

Sumatriptan/triptan as reference:

- *Individual drugs:* GitHub link
- *Drug classes:* GitHub link

Ibuprofen/NSAID as reference:

- *Individual drugs:* GitHub link
- *Drug classes:* GitHub link

Placebo as reference:

- *Individual drugs:* GitHub link
- *Drug classes:* GitHub link

7.20 Adverse events – Paraesthesia

Sumatriptan/triptan as reference:

- *Individual drugs:* GitHub link
- *Drug classes:* GitHub link

Ibuprofen/NSAID as reference:

- *Individual drugs:* GitHub link
- *Drug classes:* GitHub link

Placebo as reference:

- *Individual drugs:* GitHub link
- *Drug classes:* GitHub link

7.21 Adverse events – Pruritus

Sumatriptan/triptan as reference:

- *Individual drugs:* GitHub link
- *Drug classes:* GitHub link

Ibuprofen/NSAID as reference:

- *Individual drugs:* Sufficient outcome data not available for analysis.
- *Drug classes:* GitHub link

Placebo as reference:

- *Individual drugs:* GitHub link
- *Drug classes:* GitHub link

7.22 Adverse events – Sedation

Sumatriptan/triptan as reference:

- *Individual drugs:* GitHub link
- *Drug classes:* GitHub link

Ibuprofen/NSAID as reference:

- *Individual drugs:* GitHub link
- *Drug classes:* GitHub link

Placebo as reference:

- *Individual drugs:* GitHub link
- *Drug classes:* GitHub link

7.23 Adverse events – Serotonergic syndrome

- Sufficient outcome data not available for analysis.

7.24 Adverse events – Vertigo

Sumatriptan/triptan as reference:

- *Individual drugs:* GitHub link
- *Drug classes:* GitHub link

Ibuprofen/NSAID as reference:

- *Individual drugs:* Sufficient outcome data not available for analysis.
- *Drug classes:* GitHub link

Placebo as reference:

- *Individual drugs:* GitHub link
- *Drug classes:* GitHub link

7.25 Adverse events – Vomiting

Sumatriptan/triptan as reference:

- *Individual drugs:* GitHub link
- *Drug classes:* GitHub link

Ibuprofen/NSAID as reference:

- *Individual drugs:* GitHub link
- *Drug classes:* GitHub link

Placebo as reference:

- *Individual drugs:* GitHub link
- *Drug classes:* GitHub link

Appendix 8. League tables

The following pages contain the league tables for each outcome (by individual drugs and drug classes). Mixed estimates (direct and/or indirect estimates from the network meta-analyses) are reported in the coloured triangles (bottom left), while estimates from the direct comparisons only are reported in the white triangles (top right). Network meta-analyses for all the presented league tables were carried out using a random effects model with inverse variance.

8.01 Efficacy – Pain freedom at 2 hours

Individual drugs:

**ASA**	.	.	.	.	.	.	.	.	.	.	.	2.34 (1.50-3.65)	.	.	1.04 (0.49-2.19)	.	.
1.00 (0.61-1.62)	**ALM**	.	.	.	.	.	.	.	.	.	.	2.62 (1.89-3.63)	.	.	0.71 (0.46-1.10)	.	0.82 (0.47-1.44)
1.43 (0.79-2.62)	1.44 (0.87-2.37)	**CEL**	.	.	.	.	.	.	.	.	.	1.83 (1.19-2.82)	.	.	.	.	.
0.86 (0.43-1.72)	0.87 (0.47-1.58)	0.60 (0.30-1.21)	**DIC**	.	.	.	.	.	.	.	.	3.05 (1.76-5.27)	.	.	.	.	.
0.51 (0.32-0.80)	0.51 (0.37-0.70)	0.35 (0.22-0.57)	0.59 (0.33-1.05)	**ELE**	.	.	.	.	2.04 (1.03-4.05)	.	.	5.32 (4.21-6.74)	.	.	1.59 (1.12-2.26)	.	1.54 (0.87-2.74)
0.80 (0.45-1.42)	0.80 (0.50-1.28)	0.56 (0.31-1.00)	0.92 (0.47-1.82)	1.57 (1.01-2.46)	**FRO**	.	.	.	.	.	.	4.51 (2.84-7.16)	.	.	0.45 (0.23-0.86)	.	.
1.22 (0.68-2.20)	1.23 (0.76-1.99)	0.85 (0.47-1.55)	1.42 (0.71-2.81)	2.41 (1.52-3.81)	1.53 (0.86-2.73)	**IBU**	.	.	.	.	.	2.15 (1.42-3.25)	.	.	.	.	.
1.13 (0.68-1.88)	1.14 (0.78-1.67)	0.79 (0.47-1.33)	1.31 (0.71-2.44)	2.24 (1.58-3.17)	1.42 (0.87-2.34)	0.93 (0.56-1.54)	**LAS**	.	.	.	.	2.32 (1.74-3.09)	.	.	.	.	.
1.09 (0.65-1.85)	1.10 (0.73-1.64)	0.76 (0.44-1.31)	1.26 (0.67-2.39)	2.15 (1.48-3.13)	1.37 (0.82-2.29)	0.89 (0.53-1.51)	0.96 (0.62-1.48)	**NAP**	.	.	.	2.30 (1.57-3.37)	.	.	0.66 (0.46-0.96)	.	.
1.52 (0.91-2.55)	1.53 (1.03-2.26)	1.06 (0.63-1.80)	1.77 (0.94-3.31)	3.01 (2.13-4.25)	1.91 (1.16-3.17)	1.25 (0.75-2.09)	1.34 (0.88-2.05)	1.40 (0.90-2.17)	**NAR**	.	.	1.92 (1.31-2.82)	.	0.32 (0.16-0.62)	0.37 (0.20-0.70)	.	.
1.29 (0.69-2.43)	1.30 (0.76-2.22)	0.90 (0.47-1.71)	1.50 (0.73-3.10)	2.55 (1.52-4.27)	1.62 (0.87-3.02)	1.06 (0.56-1.99)	1.14 (0.65-1.99)	1.19 (0.67-2.11)	0.85 (0.48-1.49)	**PAR**	.	2.00 (1.21-3.29)	.	0.54 (0.19-1.52)	.	.	.
1.09 (0.42-2.85)	1.09 (0.45-2.69)	0.76 (0.29-2.00)	1.26 (0.45-3.51)	2.15 (0.89-5.22)	1.37 (0.53-3.55)	0.89 (0.34-2.32)	0.96 (0.39-2.39)	1.00 (0.40-2.51)	0.72 (0.29-1.79)	0.84 (0.31-2.26)	**PHE**	2.41 (1.02-5.72)	.	.	.	.	.
2.63 (1.73-3.99)	2.64 (2.05-3.39)	1.83 (1.19-2.82)	3.05 (1.76-5.27)	5.19 (4.25-6.33)	3.30 (2.20-4.94)	2.15 (1.42-3.25)	2.32 (1.74-3.09)	2.41 (1.74-3.33)	1.73 (1.27-2.34)	2.03 (1.26-3.27)	2.41 (1.02-5.72)	**placebo**	0.52 (0.39-0.69)	0.22 (0.18-0.27)	0.29 (0.25-0.33)	0.50 (0.34-0.74)	0.27 (0.21-0.35)
1.34 (0.81-2.21)	1.34 (0.93-1.95)	0.93 (0.56-1.56)	1.55 (0.84-2.87)	1.46 (1.18-1.81)	1.68 (1.03-2.74)	1.10 (0.67-1.80)	1.18 (0.79-1.76)	1.23 (0.80-1.88)	0.88 (0.58-1.33)	1.04 (0.60-1.79)	1.23 (0.50-3.04)	0.51 (0.39-0.67)	**RIM**	.	0.89 (0.40-1.97)	.	.
0.62 (0.40-0.98)	0.63 (0.46-0.85)	0.43 (0.27-0.69)	0.72 (0.41-1.29)	1.23 (0.95-1.60)	0.78 (0.50-1.22)	0.51 (0.33-0.80)	0.55 (0.39-0.77)	0.57 (0.40-0.83)	0.41 (0.29-0.57)	0.48 (0.29-0.79)	0.57 (0.24-1.38)	0.24 (0.20-0.28)	0.47 (0.33-0.65)	**RIZ**	1.02 (0.70-1.48)	.	1.35 (0.74-2.48)
0.74 (0.48-1.14)	0.74 (0.57-0.97)	0.52 (0.33-0.81)	0.86 (0.49-1.51)	1.46 (1.18-1.81)	0.93 (0.62-1.40)	0.61 (0.40-0.93)	0.65 (0.48-0.89)	0.68 (0.49-0.94)	0.49 (0.35-0.67)	0.57 (0.35-0.93)	0.68 (0.28-1.63)	0.28 (0.25-0.32)	0.55 (0.41-0.74)	1.19 (0.97-1.45)	**SUM**	.	1.06 (0.71-1.58)
1.32 (0.75-2.34)	1.33 (0.84-2.10)	0.92 (0.52-1.65)	1.53 (0.79-3.00)	2.61 (1.69-4.03)	1.66 (0.95-2.90)	1.08 (0.62-1.91)	1.17 (0.72-1.89)	1.21 (0.73-2.01)	0.87 (0.53-1.42)	1.02 (0.56-1.89)	1.21 (0.47-3.13)	0.50 (0.34-0.74)	0.99 (0.62-1.59)	2.12 (1.39-3.25)	1.79 (1.20-2.67)	**UBR**	.
0.77 (0.49-1.22)	0.77 (0.58-1.03)	0.54 (0.33-0.86)	0.89 (0.50-1.60)	1.52 (1.17-1.97)	0.97 (0.62-1.51)	0.63 (0.40-0.99)	0.68 (0.48-0.96)	0.71 (0.49-1.02)	0.51 (0.35-0.72)	0.60 (0.36-0.99)	0.71 (0.29-1.71)	0.29 (0.24-0.36)	0.57 (0.41-0.80)	1.23 (0.96-1.59)	1.04 (0.84-1.28)	0.58 (0.38-0.89)	**ZOL**

The network meta-analysis results are in the blue cells (direct estimates in the white cells). Participants with pain freedom at 2 hours are reported for each comparison as odds ratio (OR), along with 95% confidence intervals. Coloured bottom left triangle: estimates above 1 favours the medication defined by the column. White top right triangle: estimates above 1 favours the medication defined by the row. ASA = Acetylsalicylic acid; ALM = Almotriptan; CEL = Celecoxib; DIC = Diclofenac; ELE = Eletriptan; FRO = Frovatriptan; IBU = Ibuprofen; LAS = Lasmiditan; NAP = Naproxen sodium; NAR = Naratriptan; PAR = Paracetamol; PHE = Phenazone; RIM = Rimegepant; RIZ = Rizatriptan; SUM = Sumatriptan; UBR = Ubrogepant; ZOL = Zolmitriptan.

Drug classes:

**antipyretic**	.	.	.	2.00 (1.19-3.36)	0.54 (0.19-1.57)
0.85 (0.47-1.53)	**ditan**	.	.	2.33 (1.70-3.18)	.
1.00 (0.57-1.73)	1.17 (0.79-1.74)	**gepant**	.	1.95 (1.53-2.50)	0.89 (0.39-2.06)
0.83 (0.49-1.41)	0.98 (0.68-1.41)	0.84 (0.61-1.14)	**NSAID**	2.28 (1.86-2.80)	0.73 (0.51-1.04)
1.98 (1.21-3.25)	2.33 (1.70-3.18)	1.99 (1.56-2.53)	2.38 (1.96-2.88)	**placebo**	0.28 (0.25-0.30)
0.55 (0.33-0.90)	0.64 (0.46-0.89)	0.55 (0.42-0.71)	0.66 (0.53-0.81)	0.28 (0.25-0.30)	**triptan**

The network meta-analysis results are in the blue cells (direct estimates in the white cells). Participants with pain freedom at 2 hours are reported for each comparison as odds ratio (OR), along with 95% confidence intervals. Coloured bottom left triangle: estimates above 1 favours the drug class defined by the column. White top right triangle: estimates above 1 favours the drug class defined by the row. NSAID = non-steroidal anti-inflammatory drug.

8.02 Efficacy – Sustained pain freedom from 2 to 24 hours

Individual drugs:

**ALM**	.	.	.	.	.	.	.	.	2.72 (1.92-3.83)	.	.	0.82 (0.42- 1.61)	.	0.86 (0.49- 1.50)
1.55 (0.89-2.69)	**CEL**	.	.	.	.	.	.	.	1.71 (1.07-2.74)	.	.	.	.	.
0.91 (0.43-1.94)	0.59 (0.26-1.36)	**DIC**	.	.	.	.	.	.	2.90 (1.45- 5.80)	.	.	.	.	.
0.62 (0.41-0.92)	0.40 (0.23-0.69)	0.68 (0.32-1.43)	**ELE**	.	.	.	2.12 (1.00-4.49)	.	3.73 (2.64- 5.28)	.	.	2.55 (1.35- 4.82)	.	1.55 (0.86- 2.79)
0.35 (0.11-1.07)	0.23 (0.07-0.73)	0.38 (0.11-1.38)	0.57 (0.19-1.73)	**IBU**	.	.	.	.	7.58 (2.58-22.27)	.	.	.	.	.
1.19 (0.78-1.83)	0.77 (0.44-1.36)	1.31 (0.61-2.80)	1.93 (1.26-2.97)	3.42 (1.11-10.50)	**LAS**	.	.	.	2.22 (1.62- 3.04)	.	.	.	.	.
1.20 (0.77-1.87)	0.77 (0.43-1.39)	1.31 (0.60-2.85)	1.94 (1.24-3.03)	3.43 (1.10-10.64)	1.00 (0.63-1.61)	**NAP**	.	.	1.95 (1.30- 2.91)	.	.	0.75 (0.50- 1.11)	.	.
1.68 (0.77-3.66)	1.09 (0.46-2.58)	1.84 (0.68-5.03)	2.73 (1.35-5.52)	4.82 (1.31-17.67)	1.41 (0.64-3.11)	1.41 (0.63-3.14)	**NAR**	.	0.92 (0.37- 2.29)	.	.	.	.	.
1.59 (0.63-4.05)	1.03 (0.38-2.81)	1.74 (0.57-5.38)	2.58 (1.02-6.57)	4.56 (1.13-18.44)	1.34 (0.52-3.43)	1.33 (0.51-3.46)	0.95 (0.30-2.98)	**PAR**	1.60 (0.56- 4.58)	.	0.65 (0.20- 2.09)	.	.	.
2.65 (1.98-3.54)	1.71 (1.07-2.74)	2.90 (1.45-5.80)	4.29 (3.21-5.74)	7.58 (2.58-22.27)	2.22 (1.62-3.04)	2.21 (1.56-3.14)	1.57 (0.76-3.25)	1.66 (0.68-4.04)	**placebo**	0.42 (0.31- 0.56)	0.32 (0.24- 0.44)	0.31 (0.26- 0.38)	0.51 (0.34- 0.76)	0.25 (0.15- 0.41)
1.10 (0.73-1.66)	0.71 (0.41-1.24)	1.21 (0.57-2.56)	1.79 (1.19-2.69)	3.16 (1.04-9.64)	0.92 (0.60-1.42)	0.92 (0.59-1.45)	0.66 (0.30-1.43)	0.69 (0.27-1.76)	0.42 (0.31-0.56)	**RIM**	.	1.14 (0.51- 2.57)	.	.
0.84 (0.56-1.26)	0.54 (0.31-0.94)	0.92 (0.43-1.95)	1.36 (0.91-2.04)	2.40 (0.78-7.34)	0.70 (0.46-1.08)	0.70 (0.44-1.11)	0.50 (0.23-1.09)	0.53 (0.22-1.28)	0.32 (0.24-0.43)	0.76 (0.50-1.15)	**RIZ**	.	.	1.52 (0.82- 2.82)
0.87 (0.63-1.20)	0.56 (0.34-0.93)	0.95 (0.46-1.94)	1.41 (1.02-1.93)	2.48 (0.83-7.41)	0.73 (0.51-1.05)	0.73 (0.51-1.03)	0.52 (0.25-1.08)	0.54 (0.22-1.35)	0.33 (0.27-0.39)	0.79 (0.56-1.10)	1.04 (0.74-1.45)	**SUM**	.	1.12 (0.65- 1.95)
1.35 (0.82-2.22)	0.87 (0.47-1.62)	1.48 (0.66-3.30)	2.19 (1.33-3.60)	3.86 (1.22-12.22)	1.13 (0.68-1.89)	1.13 (0.66-1.93)	0.80 (0.35-1.84)	0.85 (0.32-2.25)	0.51 (0.34-0.76)	1.22 (0.74-2.01)	1.61 (0.98-2.66)	1.56 (1.00-2.42)	**UBR**	.
0.91 (0.64-1.29)	0.59 (0.34-1.02)	0.99 (0.47-2.10)	1.47 (1.03-2.11)	2.60 (0.85-7.93)	0.76 (0.50-1.17)	0.76 (0.49-1.18)	0.54 (0.25-1.16)	0.57 (0.23-1.44)	0.34 (0.26-0.46)	0.82 (0.55-1.23)	1.08 (0.75-1.57)	1.05 (0.77-1.42)	0.67 (0.41-1.10)	**ZOL**

The network meta-analysis results are in the blue cells (direct estimates in the white cells). Participants with sustained pain freedom from 2 to 24 hours are reported for each comparison as odds ratio (OR), along with 95% confidence intervals. Coloured bottom left triangle: estimates above 1 favours the medication defined by the column. White top right triangle: estimates above 1 favours the medication defined by the row. ALM = Almotriptan; CEL = Celecoxib; DIC = Diclofenac; ELE = Eletriptan; IBU = Ibuprofen; LAS = Lasmiditan; NAP = Naproxen sodium; NAR = Naratriptan; PAR = Paracetamol; RIM = Rimegepant; RIZ = Rizatriptan; SUM = Sumatriptan; UBR = Ubrogepant; ZOL = Zolmitriptan.

Drug classes:

**antipyretic**	.	.	.	1.60 (0.56-4.59)	0.65 (0.20-2.10)
0.74 (0.29-1.90)	**ditan**	.	.	2.22 (1.62-3.05)	.
0.74 (0.30-1.83)	0.99 (0.67-1.47)	**gepant**	.	2.23 (1.76-2.84)	1.14 (0.51-2.58)
0.72 (0.29-1.80)	0.97 (0.65-1.45)	0.98 (0.69-1.38)	**NSAID**	2.16 (1.65-2.83)	0.75 (0.50-1.11)
1.65 (0.69-3.99)	2.22 (1.62-3.05)	2.25 (1.78-2.84)	2.29 (1.78-2.95)	**placebo**	0.32 (0.28-0.37)
0.53 (0.22-1.28)	0.71 (0.50-1.00)	0.72 (0.55-0.94)	0.73 (0.56-0.96)	0.32 (0.28-0.37)	**triptan**

The network meta-analysis results are in the blue cells (direct estimates in the white cells). Participants with sustained pain freedom from 2 to 24 hours are reported for each comparison as odds ratio (OR), along with 95% confidence intervals. Coloured bottom left triangle: estimates above 1 favours the drug class defined by the column. White top right triangle: estimates above 1 favours the drug class defined by the row. NSAID = non-steroidal anti-inflammatory drug.

8.03 Efficacy – Pain relief at 2 hours

Individual drugs:

**ASA**	.	.	.	.	.	.	.	.	.	.	.	1.78<br>(1.40-2.25)	.	.	1.00<br>(0.57-1.78)	.	.
0.73<br>(0.54-1.00)	**ALM**	.	.	.	.	.	.	.	.	.	.	2.44<br>(1.79-3.34)	.	.	0.92<br>(0.67-1.27)	.	0.80<br>(0.52-1.21)
1.06<br>(0.71-1.58)	1.45<br>(0.98-2.13)	**CEL**	.	.	.	.	.	.	.	.	.	1.75<br>(1.26-2.43)	.	.	.	.	.
0.85<br>(0.52-1.38)	1.16<br>(0.71-1.88)	0.80<br>(0.46-1.38)	**DIC**	.	.	.	.	.	.	.	.	2.19<br>(1.42-3.39)	.	.	.	.	.
0.46<br>(0.36-0.60)	0.63<br>(0.50-0.81)	0.44<br>(0.31-0.62)	0.55<br>(0.35-0.86)	**ELE**	.	.	.	.	1.55<br>(0.94-2.57)	.	.	4.03<br>(3.46-4.69)	.	.	1.45<br>(1.17-1.80)	.	1.24<br>(0.83-1.85)
0.91<br>(0.67-1.25)	1.25<br>(0.92-1.69)	0.86<br>(0.58-1.28)	1.08<br>(0.66-1.76)	1.97<br>(1.53-2.54)	**FRO**	.	.	.	.	.	.	2.06<br>(1.63-2.60)	.	.	0.66<br>(0.43-1.00)	.	.
0.94<br>(0.68-1.31)	1.29<br>(0.93-1.78)	0.89<br>(0.59-1.34)	1.11<br>(0.68-1.83)	2.04<br>(1.55-2.68)	1.03<br>(0.74-1.43)	**IBU**	.	.	.	.	.	1.97<br>(1.54-2.50)	.	.	.	.	.
0.98<br>(0.73-1.32)	1.34<br>(1.01-1.78)	0.93<br>(0.64-1.35)	1.16<br>(0.72-1.86)	2.12<br>(1.68-2.67)	1.07<br>(0.80-1.43)	1.04<br>(0.77-1.41)	**LAS**	.	.	.	.	1.89<br>(1.57-2.28)	.	.	.	.	.
0.89<br>(0.65-1.22)	1.22<br>(0.91-1.64)	0.85<br>(0.57-1.25)	1.06<br>(0.65-1.72)	1.93<br>(1.51-2.48)	0.98<br>(0.72-1.33)	0.95<br>(0.69-1.31)	0.91<br>(0.68-1.21)	**NAP**	.	.	.	1.96<br>(1.53-2.51)	.	1.00<br>(0.11-8.95)	0.78<br>(0.60-1.01)	.	.
0.89<br>(0.67-1.20)	1.22<br>(0.93-1.61)	0.84<br>(0.58-1.23)	1.06<br>(0.66-1.70)	1.93<br>(1.55-2.40)	0.98<br>(0.74-1.30)	0.95<br>(0.70-1.29)	0.91<br>(0.70-1.19)	1.00<br>(0.75-1.32)	**NAR**	.	.	2.34<br>(1.87-2.93)	.	0.42<br>(0.25-0.71)	0.60<br>(0.42-0.86)	.	0.85<br>(0.50-1.45)
0.82<br>(0.54-1.24)	1.12<br>(0.75-1.68)	0.77<br>(0.48-1.24)	0.97<br>(0.56-1.69)	1.77<br>(1.22-2.56)	0.90<br>(0.60-1.35)	0.87<br>(0.57-1.32)	0.83<br>(0.56-1.24)	0.91<br>(0.61-1.37)	0.92<br>(0.62-1.35)	**PAR**	.	2.28<br>(1.60-3.24)	.	0.76<br>(0.31-1.88)	.	.	.
0.76<br>(0.38-1.52)	1.04<br>(0.53-2.06)	0.72<br>(0.35-1.49)	0.90<br>(0.41-1.97)	1.65<br>(0.85-3.19)	0.83<br>(0.42-1.65)	0.81<br>(0.41-1.62)	0.78<br>(0.40-1.53)	0.85<br>(0.43-1.69)	0.85<br>(0.44-1.68)	0.93<br>(0.45-1.94)	**PHE**	2.43<br>(1.27-4.64)	.	.	.	.	.
1.85<br>(1.47-2.33)	2.53<br>(2.05-3.13)	1.75<br>(1.26-2.43)	2.19<br>(1.42-3.39)	4.00<br>(3.51-4.57)	2.03<br>(1.63-2.52)	1.97<br>(1.54-2.50)	1.89<br>(1.57-2.28)	2.07<br>(1.67-2.57)	2.07<br>(1.72-2.50)	2.26<br>(1.61-3.20)	2.43<br>(1.27-4.64)	**placebo**	0.56<br>(0.46-0.67)	0.31<br>(0.27-0.36)	0.37<br>(0.34-0.41)	0.66<br>(0.52-0.84)	0.31<br>(0.26-0.36)
1.02<br>(0.76-1.38)	1.40<br>(1.05-1.85)	0.97<br>(0.66-1.41)	1.21<br>(0.75-1.94)	2.21<br>(1.76-2.78)	1.12<br>(0.84-1.49)	1.08<br>(0.80-1.47)	1.04<br>(0.80-1.36)	1.14<br>(0.86-1.52)	1.14<br>(0.88-1.49)	1.25<br>(0.84-1.85)	1.34<br>(0.68-2.63)	0.55<br>(0.46-0.67)	**RIM**	.	1.10<br>(0.56-2.17)	.	.
0.58<br>(0.45-0.76)	0.80<br>(0.62-1.02)	0.55<br>(0.39-0.78)	0.69<br>(0.44-1.09)	1.26<br>(1.05-1.51)	0.64<br>(0.49-0.82)	0.62<br>(0.47-0.81)	0.59<br>(0.47-0.75)	0.65<br>(0.51-0.84)	0.65<br>(0.52-0.81)	0.71<br>(0.49-1.02)	0.76<br>(0.39-1.48)	0.31<br>(0.27-0.36)	0.57<br>(0.45-0.72)	**RIZ**	1.09<br>(0.83-1.43)	.	1.16<br>(0.73-1.86)
0.67<br>(0.52-0.85)	0.91<br>(0.74-1.13)	0.63<br>(0.45-0.88)	0.79<br>(0.51-1.23)	1.44<br>(1.25-1.66)	0.73<br>(0.58-0.92)	0.71<br>(0.55-0.92)	0.68<br>(0.55-0.84)	0.75<br>(0.60-0.93)	0.75<br>(0.62-0.91)	0.82<br>(0.57-1.16)	0.88<br>(0.46-1.68)	0.36<br>(0.33-0.39)	0.65<br>(0.53-0.80)	1.15<br>(0.99-1.33)	**SUM**	.	1.00<br>(0.80-1.26)
1.22<br>(0.87-1.70)	1.67<br>(1.21-2.30)	1.15<br>(0.77-1.73)	1.44<br>(0.88-2.37)	2.63<br>(2.00-3.46)	1.33<br>(0.96-1.85)	1.29<br>(0.92-1.82)	1.24<br>(0.92-1.69)	1.36<br>(0.99-1.88)	1.36<br>(1.01-1.85)	1.49<br>(0.98-2.27)	1.60<br>(0.80-3.19)	0.66<br>(0.52-0.84)	1.19<br>(0.88-1.62)	2.09<br>(1.59-2.76)	1.82<br>(1.41-2.36)	**UBR**	.
0.61<br>(0.47-0.79)	0.83<br>(0.66-1.05)	0.58<br>(0.41-0.82)	0.72<br>(0.46-1.14)	1.32<br>(1.11-1.57)	0.67<br>(0.52-0.86)	0.65<br>(0.49-0.85)	0.62<br>(0.49-0.78)	0.68<br>(0.53-0.87)	0.68<br>(0.55-0.85)	0.75<br>(0.52-1.08)	0.80<br>(0.41-1.55)	0.33<br>(0.29-0.38)	0.60<br>(0.47-0.75)	1.05<br>(0.88-1.25)	0.91<br>(0.80-1.05)	0.50<br>(0.38-0.66)	**ZOL**

The network meta-analysis results are in the purple cells (direct estimates in the white cells). Participants with pain relief at 2 hours are reported for each comparison as odds ratio (OR), along with 95% confidence intervals. Coloured bottom left triangle: estimates above 1 favours the medication defined by the column. White top right triangle: estimates above 1 favours the medication defined by the row. ASA = Acetylsalicylic acid; ALM = Almotriptan; CEL = Celecoxib; DIC = Diclofenac; ELE = Eletriptan; FRO = Frovatriptan; IBU = Ibuprofen; LAS = Lasmiditan; NAP = Naproxen sodium; NAR = Naratriptan; PAR = Paracetamol; PHE = Phenazone; RIM = Rimegepant; RIZ = Rizatriptan; SUM = Sumatriptan; UBR = Ubrogepant; ZOL = Zolmitriptan.

Drug classes:

**antipyretic**	.	.	.	2.29 (1.56-3.35)	0.76 (0.30-1.94)
1.18 (0.77-1.82)	**ditan**	.	.	1.90 (1.53-2.37)	.
1.32 (0.87-1.98)	1.11 (0.84-1.47)	**gepant**	.	1.69 (1.42-2.01)	1.10 (0.53-2.26)
1.13 (0.76-1.68)	0.96 (0.74-1.24)	0.86 (0.70-1.07)	**NSAID**	1.92 (1.67-2.19)	0.82 (0.63-1.06)
2.25 (1.55-3.27)	1.90 (1.53-2.37)	1.71 (1.44-2.03)	1.99 (1.74-2.26)	**placebo**	0.34 (0.32-0.37)
0.78 (0.53-1.13)	0.66 (0.52-0.83)	0.59 (0.49-0.71)	0.69 (0.59-0.79)	0.35 (0.32-0.37)	**triptan**

The network meta-analysis results are in the purple cells (direct estimates in the white cells). Participants with pain relief at 2 hours are reported for each comparison as odds ratio (OR), along with 95% confidence intervals. Coloured bottom left triangle: estimates above 1 favours the drug class defined by the column. White top right triangle: estimates above 1 favours the drug class defined by the row. NSAID = non-steroidal anti-inflammatory drug.

8.04 Efficacy – Pain relapse within to 2 to 48 hours

Individual drugs:

**LAS**	1.97 (1.51-2.58)	.	.
1.97 (1.51-2.58)	**placebo**	0.72 (0.57-0.91)	0.21 (0.06-0.73)
1.43 (1.00-2.03)	0.72 (0.57-0.91)	**RIM**	.
0.42 (0.12-1.48)	0.21 (0.06-0.73)	0.29 (0.08-1.03)	**SUM**

The network meta-analysis results are in the purple cells (direct estimates in the white cells). Participants with relapse within 2 to 48 hours are reported for each comparison as odds ratio (OR), along with 95% confidence intervals. Coloured bottom left triangle: estimates below 1 favours the medication defined by the column. White top right triangle: estimates below 1 favours the medication defined by the row. LAS = Lasmiditan; RIM = Rimegepant; SUM = Sumatriptan.

Drug classes:

**ditan**	.	1.97 (1.51-2.58)	.
1.43 (1.00-2.03)	**gepant**	1.38 (1.09-1.75)	.
1.97 (1.51-2.58)	1.38 (1.09-1.75)	**placebo**	0.21 (0.06-0.73)
0.42 (0.12-1.48)	0.29 (0.08-1.03)	0.21 (0.06-0.73)	**triptan**

The network meta-analysis results are in the purple cells (direct estimates in the white cells). Participants with relapse within 2 to 48 hours are reported for each comparison as odds ratio (OR), along with 95% confidence intervals. Coloured bottom left triangle: estimates below 1 favours the drug class defined by the column. White top right triangle: estimates below 1 favours the drug class defined by the row.

8.05 Efficacy – Use of rescue medication within 2 to 24 hours

Individual drugs:

**ASA**	.	.	.	.	.	.	.	.	.	0.47 (0.34-0.63)	.	.	1.13 (0.64-1.98)	.
1.01 (0.71-1.44)	**ALM**	.	.	.	.	.	.	.	.	0.48 (0.36-0.65)	.	.	1.18 (0.87-1.61)	0.98 (0.65-1.50)
1.06 (0.66-1.70)	1.05 (0.68-1.60)	**CEL**	.	.	.	.	.	.	.	0.44 (0.30-0.64)	.	.	.	.
1.84 (1.34-2.54)	1.82 (1.43-2.32)	1.74 (1.16-2.59)	**ELE**	.	.	.	.	0.48 (0.27-0.86)	.	0.25 (0.21-0.29)	.	.	0.68 (0.53-0.88)	0.59 (0.39-0.89)
1.01 (0.71-1.43)	1.00 (0.75-1.32)	0.95 (0.62-1.45)	0.55 (0.43-0.70)	**FRO**	.	.	.	.	.	0.45 (0.37-0.56)	.	.	1.18 (0.79-1.78)	.
0.79 (0.46-1.37)	0.78 (0.47-1.31)	0.75 (0.41-1.36)	0.43 (0.26-0.70)	0.78 (0.47-1.30)	**IBU**	.	.	.	.	0.59 (0.37-0.94)	.	.	.	.
1.01 (0.69-1.47)	1.00 (0.73-1.37)	0.95 (0.61-1.48)	0.55 (0.41-0.72)	1.00 (0.73-1.36)	1.27 (0.75-2.16)	**LAS**	.	.	.	0.46 (0.37-0.59)	.	.	.	.
0.97 (0.68-1.38)	0.96 (0.72-1.27)	0.91 (0.60-1.40)	0.53 (0.41-0.67)	0.96 (0.72-1.27)	1.22 (0.73-2.04)	0.96 (0.70-1.32)	**NAP**	.	.	0.52 (0.41-0.65)	.	.	1.12 (0.87-1.44)	.
1.02 (0.66-1.59)	1.01 (0.68-1.50)	0.97 (0.58-1.59)	0.56 (0.39-0.79)	1.01 (0.69-1.49)	1.29 (0.73-2.30)	1.01 (0.67-1.53)	1.06 (0.71-1.57)	**NAR**	.	0.33 (0.22-0.51)	.	1.28 (0.78-2.09)	.	.
1.39 (0.71-2.75)	1.38 (0.72-2.64)	1.32 (0.64-2.70)	0.76 (0.40-1.43)	1.38 (0.72-2.64)	1.76 (0.81-3.82)	1.38 (0.71-2.68)	1.44 (0.75-2.76)	1.36 (0.68-2.75)	**PAR**	0.34 (0.18-0.62)	.	.	.	.
0.47 (0.35-0.62)	0.46 (0.38-0.57)	0.44 (0.30-0.64)	0.25 (0.22-0.29)	0.46 (0.38-0.56)	0.59 (0.37-0.94)	0.46 (0.37-0.59)	0.48 (0.39-0.60)	0.46 (0.33-0.64)	0.34 (0.18-0.62)	**placebo**	2.30 (1.89-2.80)	2.20 (1.91-2.53)	2.50 (2.23-2.80)	2.70 (2.15-3.39)
1.08 (0.77-1.53)	1.07 (0.81-1.42)	1.02 (0.67-1.56)	0.59 (0.46-0.75)	1.07 (0.81-1.41)	1.37 (0.82-2.27)	1.07 (0.79-1.46)	1.12 (0.84-1.49)	1.06 (0.72-1.56)	0.78 (0.41-1.48)	2.31 (1.90-2.81)	**RIM**	.	0.75 (0.37-1.53)	.
1.02 (0.75-1.40)	1.01 (0.80-1.28)	0.96 (0.65-1.43)	0.55 (0.46-0.67)	1.01 (0.80-1.28)	1.29 (0.79-2.10)	1.01 (0.77-1.33)	1.06 (0.83-1.35)	1.00 (0.71-1.41)	0.73 (0.39-1.38)	2.18 (1.91-2.49)	0.94 (0.75-1.20)	**RIZ**	1.17 (0.85-1.62)	0.84 (0.54-1.31)
1.17 (0.87-1.58)	1.16 (0.94-1.43)	1.10 (0.75-1.62)	0.63 (0.54-0.75)	1.16 (0.93-1.43)	1.48 (0.91-2.39)	1.16 (0.89-1.50)	1.21 (0.98-1.49)	1.14 (0.81-1.62)	0.84 (0.45-1.57)	2.50 (2.25-2.77)	1.08 (0.87-1.34)	1.14 (0.97-1.34)	**SUM**	.
1.10 (0.78-1.55)	1.09 (0.85-1.39)	1.04 (0.68-1.58)	0.60 (0.48-0.75)	1.09 (0.83-1.43)	1.39 (0.84-2.30)	1.09 (0.80-1.48)	1.14 (0.86-1.50)	1.07 (0.73-1.57)	0.79 (0.41-1.50)	2.35 (1.95-2.84)	1.02 (0.77-1.33)	1.08 (0.86-1.34)	0.94 (0.76-1.16)	**ZOL**

The network meta-analysis results are in the purple cells (direct estimates in the white cells). Participants with use of rescue medications within 2 to 24 hours are reported for each comparison as odds ratio (OR), along with 95% confidence intervals. Coloured bottom left triangle: estimates below 1 favours the medication defined by the column. White top right triangle: estimates below 1 favours the medication defined by the row. ASA = Acetylsalicylic acid; ALM = Almotriptan; CEL = Celecoxib; ELE = Eletriptan; FRO = Frovatriptan;

IBU = Ibuprofen; LAS = Lasmiditan; NAP = Naproxen sodium; NAR = Naratriptan; PAR = Paracetamol; RIM = Rimegepant; RIZ = Rizatriptan; SUM = Sumatriptan; ZOL = Zolmitriptan.

Drug classes:

**antipyretic**	.	.	.	0.33 (0.16-0.65)	.
0.70 (0.33-1.48)	**ditan**	.	.	0.47 (0.34-0.64)	.
0.77 (0.37-1.59)	1.10 (0.74-1.63)	**gepant**	.	0.43 (0.34-0.55)	0.75 (0.35-1.65)
0.68 (0.34-1.39)	0.98 (0.69-1.40)	0.89 (0.66-1.21)	**NSAID**	0.49 (0.41-0.60)	1.12 (0.85-1.49)
0.33 (0.16-0.65)	0.47 (0.34-0.64)	0.43 (0.33-0.55)	0.48 (0.40-0.57)	**placebo**	2.53 (2.32-2.76)
0.83 (0.41-1.65)	1.19 (0.86-1.63)	1.08 (0.83-1.40)	1.21 (1.00-1.46)	2.53 (2.31-2.76)	**triptan**

The network meta-analysis results are in the purple cells (direct estimates in the white cells). Participants with use of rescue medications within 2 to 24 hours are reported for each comparison as odds ratio (OR), along with 95% confidence intervals. Coloured bottom left triangle: estimates below 1 favours the drug class defined by the column. White top right triangle: estimates below 1 favours the drug class defined by the row. NSAID = non-steroidal anti-inflammatory drug.

8.06 Serious adverse events

Sufficient outcome data not available for analysis.

8.07 Adverse events – Abdominal pain

Individual drugs:

**ASA**	.	.	.	.	.	.	.	.	.	0.97 (0.53-1.76)	.	.	.
1.11 (0.45-2.73)	**ALM**	.	.	.	.	.	.	.	.	0.88 (0.44-1.73)	.	.	.
0.83 (0.37-1.86)	0.75 (0.31-1.79)	**DIC**	.	.	.	.	.	.	.	1.17 (0.68-2.01)	.	.	.
1.11 (0.53-2.33)	1.01 (0.45-2.25)	1.34 (0.67-2.68)	**FRO**	.	.	.	.	.	.	0.62 (0.32-1.20)	.	1.06 (0.62-1.82)	.
0.95 (0.45-2.01)	0.86 (0.38-1.94)	1.14 (0.56-2.31)	0.85 (0.45-1.59)	**IBU**	.	.	.	.	.	1.02 (0.65-1.61)	.	.	.
0.89 (0.47-1.71)	0.81 (0.39-1.67)	1.08 (0.59-1.96)	0.80 (0.49-1.32)	0.94 (0.56-1.59)	**LAS**	.	.	.	.	1.08 (0.84-1.39)	.	.	.
0.83 (0.40-1.71)	0.75 (0.34-1.65)	1.00 (0.50-1.96)	0.74 (0.41-1.33)	0.87 (0.47-1.61)	0.92 (0.57-1.49)	**NAP**	.	.	.	1.10 (0.70-1.72)	.	1.27 (0.73-2.20)	.
2.90 (0.11-77.75)	2.63 (0.10-71.41)	3.50 (0.13-92.74)	2.61 (0.10-68.00)	3.07 (0.12-80.17)	3.25 (0.13-83.04)	3.51 (0.14-91.38)	**NAR**	.	.	0.33 (0.01-8.44)	.	.	.
1.00 (0.38-2.60)	0.90 (0.33-2.48)	1.20 (0.47-3.03)	0.89 (0.38-2.13)	1.05 (0.44-2.53)	1.11 (0.50-2.46)	1.21 (0.51-2.84)	0.34 (0.01-9.47)	**PAR**	.	0.97 (0.46-2.06)	.	.	.
1.18 (0.43-3.25)	1.07 (0.37-3.09)	1.43 (0.54-3.79)	1.06 (0.42-2.67)	1.25 (0.49-3.17)	1.32 (0.57-3.10)	1.43 (0.58-3.56)	0.41 (0.01-11.42)	1.19 (0.39-3.60)	**PHE**	0.82 (0.36-1.84)	.	.	.
0.97 (0.53-1.76)	0.88 (0.44-1.73)	1.17 (0.68-2.01)	0.87 (0.56-1.34)	1.02 (0.65-1.61)	1.08 (0.84-1.39)	1.17 (0.78-1.77)	0.33 (0.01-8.44)	0.97 (0.46-2.06)	0.82 (0.36-1.84)	**placebo**	0.94 (0.66-1.34)	0.90 (0.74-1.10)	1.19 (0.56-2.54)
1.07 (0.55-2.08)	0.96 (0.46-2.02)	1.28 (0.69-2.38)	0.96 (0.58-1.58)	1.13 (0.66-1.93)	1.19 (0.81-1.75)	1.29 (0.79-2.11)	0.37 (0.01-9.42)	1.07 (0.48-2.40)	0.90 (0.38-2.14)	1.10 (0.82-1.48)	**RIZ**	0.85 (0.59-1.21)	.
0.94 (0.50-1.76)	0.85 (0.42-1.72)	1.13 (0.64-2.01)	0.84 (0.55-1.29)	0.99 (0.60-1.62)	1.05 (0.76-1.44)	1.13 (0.74-1.73)	0.32 (0.01-8.22)	0.94 (0.43-2.04)	0.79 (0.34-1.82)	0.97 (0.80-1.17)	0.88 (0.66-1.18)	**SUM**	.
1.16 (0.44-3.03)	1.05 (0.38-2.89)	1.39 (0.55-3.53)	1.04 (0.44-2.48)	1.22 (0.51-2.94)	1.29 (0.58-2.86)	1.40 (0.59-3.30)	0.40 (0.01-11.00)	1.16 (0.40-3.37)	0.98 (0.32-2.96)	1.19 (0.56-2.54)	1.08 (0.48-2.44)	1.23 (0.57-2.69)	**ZOL**

The network meta-analysis results are in the purple cells (direct estimates in the white cells). Participants with abdominal pain during the trial are reported for each comparison as odds ratio (OR), along with 95% confidence intervals. Coloured bottom left triangle: estimates below 1 favours the medication defined by the column. White top right triangle: estimates below 1 favours the medication defined by the row. ASA = Acetylsalicylic acid; ALM = Almotriptan; DIC = Diclofenac; FRO = Frovatriptan; IBU = Ibuprofen; LAS = Lasmiditan; NAP = Naproxen sodium; NAR = Naratriptan; PAR = Paracetamol; PHE = Phenazone; RIZ = Rizatriptan; SUM = Sumatriptan; ZOL = Zolmitriptan.

Drug classes:

**antipyretic**	.	.	0.95 (0.46-1.97)	.
0.89 (0.42-1.89)	**ditan**	.	1.07 (0.86-1.34)	.
0.90 (0.42-1.91)	1.01 (0.74-1.38)	**NSAID**	1.04 (0.84-1.30)	1.27 (0.77-2.09)
0.95 (0.46-1.97)	1.07 (0.86-1.34)	1.06 (0.86-1.32)	**placebo**	1.03 (0.88-1.21)
0.99 (0.47-2.07)	1.11 (0.85-1.46)	1.10 (0.85-1.42)	1.04 (0.89-1.21)	**triptan**

The network meta-analysis results are in the purple cells (direct estimates in the white cells). Participants with abdominal pain during the trial are reported for each comparison as odds ratio (OR), along with 95% confidence intervals. Coloured bottom left triangle: estimates below 1 favours the drug class defined by the column. White top right triangle: estimates below 1 favours the drug class defined by the row. NSAID = non-steroidal anti-inflammatory drug.

8.08 Adverse events – Allergic reaction

Individual drugs:

**LAS**	1.05 (0.91-1.21)	.	.
1.05 (0.91-1.21)	**placebo**	1.06 (0.56-1.99)	0.92 (0.67-1.28)
1.11 (0.58-2.13)	1.06 (0.56-1.99)	**RIZ**	.
0.97 (0.68-1.38)	0.92 (0.67-1.28)	0.87 (0.43-1.78)	**SUM**

The network meta-analysis results are in the purple cells (direct estimates in the white cells). Participants with an allergic reaction during the trial are reported for each comparison as odds ratio (OR), along with 95% confidence intervals. Coloured bottom left triangle: estimates below 1 favours the medication defined by the column. White top right triangle: estimates below 1 favours the medication defined by the row. LAS = Lasmiditan; RIZ = Rizatriptan; SUM = Sumatriptan.

Drug classes:

**ditan**	1.05 (0.91-1.21)	.
1.05 (0.91-1.21)	**placebo**	0.95 (0.71-1.27)
1.00 (0.72-1.38)	0.95 (0.71-1.27)	**triptan**

The network meta-analysis results are in the purple cells (direct estimates in the white cells). Participants with an allergic reaction during the trial are reported for each comparison as odds ratio (OR), along with 95% confidence intervals. Coloured bottom left triangle: estimates below 1 favours the drug class defined by the column. White top right triangle: estimates below 1 favours the drug class defined by the row.

8.09 Adverse events – Chest pain/discomfort

Individual drugs:

**ALM**	.	.	.	.	.	.	.	0.78 (0.39-1.57)	.	.	0.77 (0.42-1.39)	.	2.00 (0.47-8.58)
0.78 (0.38-1.62)	**DIC**	.	.	.	.	.	.	1.12 (0.63-2.00)	.	.	.	.	.
0.62 (0.36-1.06)	0.79 (0.41-1.54)	**ELE**	.	.	.	.	.	1.64 (1.09-2.46)	.	.	0.96 (0.57-1.60)	.	1.28 (0.76-2.15)
1.11 (0.63-1.95)	1.41 (0.71-2.80)	1.78 (1.10-2.88)	**FRO**	.	.	.	.	0.64 (0.40-1.01)	.	.	1.06 (0.59-1.90)	.	.
0.77 (0.46-1.27)	0.98 (0.52-1.83)	1.23 (0.82-1.85)	0.69 (0.45-1.08)	**LAS**	.	.	.	1.15 (0.90-1.47)	.	.	.	.	.
0.78 (0.41-1.47)	0.99 (0.47-2.09)	1.25 (0.71-2.20)	0.70 (0.39-1.26)	1.01 (0.60-1.72)	**NAP**	.	.	1.07 (0.62-1.85)	.	1.00 (0.05-18.88)	1.13 (0.66-1.94)	.	.
0.79 (0.42-1.47)	1.01 (0.48-2.09)	1.27 (0.73-2.20)	0.71 (0.40-1.27)	1.03 (0.62-1.72)	1.01 (0.53-1.93)	**NAR**	.	0.88 (0.53-1.47)	.	0.67 (0.12-3.61)	1.92 (0.95-3.88)	.	.
1.78 (0.53-6.02)	2.28 (0.64-8.14)	2.87 (0.88-9.34)	1.61 (0.49-5.31)	2.33 (0.73-7.43)	2.29 (0.67-7.82)	2.26 (0.67-7.65)	**PAR**	0.36 (0.11-1.20)	.	1.00 (0.25-3.96)	.	.	.
0.88 (0.57-1.37)	1.12 (0.63-2.00)	1.42 (1.02-1.96)	0.80 (0.55-1.15)	1.15 (0.90-1.47)	1.13 (0.71-1.81)	1.12 (0.71-1.75)	0.49 (0.16-1.53)	**placebo**	1.65 (0.55-4.93)	0.90 (0.65-1.24)	0.94 (0.80-1.11)	0.67 (0.35-1.30)	0.83 (0.65-1.06)
1.50 (0.49-4.65)	1.92 (0.58-6.33)	2.42 (0.81-7.20)	1.36 (0.45-4.10)	1.96 (0.67-5.73)	1.93 (0.62-6.05)	1.91 (0.61-5.93)	0.84 (0.18-3.94)	1.71 (0.60-4.85)	**RIM**	.	0.52 (0.16-1.68)	.	.
0.99 (0.60-1.63)	1.27 (0.67-2.38)	1.59 (1.07-2.38)	0.90 (0.58-1.38)	1.29 (0.91-1.84)	1.28 (0.76-2.14)	1.26 (0.76-2.07)	0.56 (0.18-1.74)	1.13 (0.88-1.45)	0.66 (0.23-1.92)	**RIZ**	0.77 (0.56-1.05)	.	0.48 (0.16-1.42)
0.83 (0.53-1.28)	1.05 (0.58-1.91)	1.33 (0.95-1.86)	0.75 (0.51-1.08)	1.08 (0.81-1.43)	1.06 (0.67-1.70)	1.05 (0.66-1.66)	0.46 (0.15-1.45)	0.94 (0.81-1.09)	0.55 (0.19-1.57)	0.83 (0.65-1.07)	**SUM**	.	1.22 (0.74-2.02)
0.59 (0.27-1.31)	0.76 (0.32-1.82)	0.95 (0.46-1.99)	0.54 (0.25-1.14)	0.77 (0.38-1.56)	0.76 (0.34-1.71)	0.75 (0.34-1.67)	0.33 (0.09-1.24)	0.67 (0.35-1.30)	0.39 (0.11-1.36)	0.60 (0.30-1.21)	0.72 (0.37-1.41)	**UBR**	.
0.77 (0.48-1.24)	0.98 (0.53-1.82)	1.24 (0.87-1.75)	0.70 (0.46-1.06)	1.00 (0.72-1.39)	0.99 (0.59-1.65)	0.97 (0.59-1.60)	0.43 (0.14-1.37)	0.87 (0.70-1.09)	0.51 (0.18-1.48)	0.78 (0.56-1.07)	0.93 (0.73-1.19)	1.30 (0.65-2.60)	**ZOL**

The network meta-analysis results are in the purple cells (direct estimates in the white cells). Participants with chest pain or discomfort during the trial are reported for each comparison as odds ratio (OR), along with 95% confidence intervals. Coloured bottom left triangle: estimates below 1 favours the medication defined by the column. White top right triangle: estimates below 1 favours the medication defined by the row. ALM = Almotriptan; DIC = Diclofenac; ELE = Eletriptan; FRO = Frovatriptan; LAS = Lasmiditan; NAP = Naproxen sodium; NAR = Naratriptan; PAR = Paracetamol; RIM = Rimegepant; RIZ = Rizatriptan; SUM = Sumatriptan; UBR = Ubrogepant; ZOL = Zolmitriptan.

Drug classes:

**antipyretic**	.	.	.	0.36 (0.11-1.21)	1.00 (0.25-3.98)
0.46 (0.14-1.46)	**ditan**	.	.	1.15 (0.89-1.47)	.
0.46 (0.13-1.64)	1.01 (0.55-1.88)	**gepant**	.	1.17 (0.66-2.07)	0.52 (0.16-1.69)
0.47 (0.14-1.53)	1.02 (0.65-1.59)	1.01 (0.51-1.97)	**NSAID**	1.10 (0.73-1.64)	1.13 (0.65-1.94)
0.52 (0.17-1.63)	1.15 (0.89-1.47)	1.13 (0.64-1.99)	1.13 (0.78-1.63)	**placebo**	0.96 (0.85-1.08)
0.50 (0.16-1.56)	1.09 (0.83-1.44)	1.08 (0.61-1.91)	1.07 (0.74-1.56)	0.95 (0.84-1.07)	**triptan**

The network meta-analysis results are in the purple cells (direct estimates in the white cells). Participants with chest pain or discomfort during the trial are reported for each comparison as odds ratio (OR), along with 95% confidence intervals. Coloured bottom left triangle: estimates below 1 favours the drug class defined by the column. White top right triangle: estimates below 1 favours the drug class defined by the row. NSAID = non-steroidal anti-inflammatory drug.

8.10 Adverse events - Constipation

Individual drugs:

**LAS**	1.02<br>(0.84-1.25)	.
1.02<br>(0.84-1.25)	**placebo**	0.97<br>(0.77-1.21)
0.99<br>(0.73-1.34)	0.97<br>(0.77-1.21)	**SUM**

The network meta-analysis results are in the purple cells (direct estimates in the white cells). Participants with constipation during the trial are reported for each comparison as odds ratio (OR), along with 95% confidence intervals. Coloured bottom left triangle: estimates below 1 favours the medication defined by the column. White top right triangle: estimates below 1 favours the medication defined by the row. LAS = Lasmiditan; SUM = Sumatriptan.

Drug classes:

**ditan**	1.02<br>(0.84-1.25)	.
1.02<br>(0.84-1.25)	**placebo**	0.97<br>(0.77-1.21)
0.99<br>(0.73-1.34)	0.97<br>(0.77-1.21)	**triptan**

The network meta-analysis results are in the purple cells (direct estimates in the white cells). Participants with constipation during the trial are reported for each comparison as odds ratio (OR), along with 95% confidence intervals. Coloured bottom left triangle: estimates below 1 favours the drug class defined by the column. White top right triangle: estimates below 1 favours the drug class defined by the row.

8.11 Adverse events – Diarrhoea

Individual drugs:

**ASA**	.	.	.	.	.	.	.	0.96 (0.60-1.55)	.	.	.	.
0.97 (0.54-1.76)	**ALM**	.	.	.	.	.	.	0.82 (0.47-1.43)	.	.	1.06 (0.69-1.62)	.
0.89 (0.48-1.65)	0.92 (0.54-1.54)	**DIC**	.	.	.	.	.	1.08 (0.73-1.58)	.	.	.	.
0.89 (0.36-2.22)	0.91 (0.39-2.14)	1.00 (0.42-2.38)	**ELE**	.	.	.	.	1.08 (0.50-2.36)	.	.	.	.
0.91 (0.55-1.51)	0.94 (0.64-1.37)	1.02 (0.68-1.54)	1.03 (0.46-2.27)	**LAS**	.	.	.	1.05 (0.91-1.22)	.	.	.	.
0.83 (0.46-1.48)	0.85 (0.53-1.36)	0.93 (0.56-1.54)	0.93 (0.40-2.18)	0.91 (0.63-1.30)	**NAP**	.	.	1.07 (0.74-1.56)	.	.	1.21 (0.82-1.77)	.
1.07 (0.48-2.41)	1.10 (0.53-2.31)	1.20 (0.57-2.56)	1.21 (0.44-3.34)	1.18 (0.60-2.29)	1.30 (0.62-2.69)	**NAR**	.	0.89 (0.47-1.71)	.	.	.	.
1.03 (0.43-2.48)	1.06 (0.47-2.39)	1.16 (0.50-2.65)	1.16 (0.40-3.39)	1.13 (0.53-2.39)	1.24 (0.56-2.78)	0.96 (0.36-2.56)	**PHE**	0.93 (0.45-1.94)	.	.	.	.
0.96 (0.60-1.55)	0.99 (0.70-1.40)	1.08 (0.73-1.58)	1.08 (0.50-2.36)	1.05 (0.91-1.22)	1.16 (0.83-1.61)	0.89 (0.47-1.71)	0.93 (0.45-1.94)	**placebo**	1.65 (0.60-4.53)	1.12 (0.83-1.53)	0.93 (0.81-1.08)	1.21 (0.29-4.97)
1.53 (0.52-4.56)	1.57 (0.56-4.44)	1.72 (0.60-4.92)	1.73 (0.49-6.04)	1.68 (0.62-4.52)	1.85 (0.66-5.18)	1.43 (0.44-4.62)	1.49 (0.44-5.06)	1.60 (0.60-4.25)	**RIM**	.	0.65 (0.21-2.00)	.
1.13 (0.66-1.93)	1.16 (0.76-1.75)	1.26 (0.80-1.99)	1.27 (0.56-2.87)	1.23 (0.92-1.65)	1.36 (0.91-2.03)	1.05 (0.52-2.10)	1.09 (0.50-2.37)	1.17 (0.91-1.50)	0.73 (0.27-2.01)	**RIZ**	0.82 (0.60-1.12)	.
0.92 (0.56-1.52)	0.95 (0.67-1.34)	1.04 (0.69-1.56)	1.04 (0.47-2.30)	1.01 (0.83-1.24)	1.11 (0.80-1.55)	0.86 (0.44-1.67)	0.90 (0.42-1.89)	0.96 (0.84-1.10)	0.60 (0.23-1.61)	0.82 (0.64-1.05)	**SUM**	.
1.16 (0.26-5.17)	1.19 (0.28-5.12)	1.30 (0.30-5.64)	1.30 (0.26-6.58)	1.27 (0.31-5.28)	1.40 (0.33-5.99)	1.08 (0.23-5.13)	1.12 (0.23-5.54)	1.21 (0.29-4.97)	0.76 (0.13-4.23)	1.03 (0.24-4.34)	1.25 (0.30-5.21)	**UBR**

The network meta-analysis results are in the purple cells (direct estimates in the white cells). Participants with diarrhoea during the trial are reported for each comparison as odds ratio (OR), along with 95% confidence intervals. Coloured bottom left triangle: estimates below 1 favours the medication defined by the column. White top right triangle: estimates below 1 favours the medication defined by the row. ASA = Acetylsalicylic acid; ALM = Almotriptan; DIC = Diclofenac; ELE = Eletriptan; LAS = Lasmiditan; NAP = Naproxen sodium; NAR = Naratriptan; PAR = Paracetamol; PHE = Phenazone; RIM = Rimegepant; RIZ = Rizatriptan; SUM = Sumatriptan; UBR = Ubrogepant.

Drug classes:

**ditan**	.	.	1.05 (0.91-1.22)	.
1.55 (0.68-3.51)	**gepant**	.	0.67 (0.30-1.54)	0.65 (0.21-2.00)
0.99 (0.76-1.28)	0.64 (0.28-1.47)	**NSAID**	1.04 (0.83-1.29)	1.21 (0.82-1.77)
1.05 (0.91-1.22)	0.68 (0.30-1.52)	1.07 (0.86-1.32)	**placebo**	1.00 (0.88-1.13)
1.05 (0.87-1.28)	0.68 (0.30-1.53)	1.07 (0.84-1.35)	1.00 (0.88-1.13)	**triptan**

The network meta-analysis results are in the purple cells (direct estimates in the white cells). Participants with diarrhoea during the trial are reported for each comparison as odds ratio (OR), along with 95% confidence intervals. Coloured bottom left triangle: estimates below 1 favours the drug class defined by the column. White top right triangle: estimates below 1 favours the drug class defined by the row. NSAID = non-steroidal anti-inflammatory drug.

8.12 Adverse events – Dizziness

Individual drugs:

**ASA**	.	.	.	.	.	.	.	.	.	.	1.06 (0.58-1.92)	.	.	.	.	.
1.14 (0.56-2.33)	**ALM**	.	.	.	.	.	.	.	.	.	0.74 (0.39-1.42)	.	.	0.95 (0.56-1.64)	.	0.53 (0.20-1.44)
1.14 (0.54-2.42)	1.00 (0.55-1.83)	**CEL**	.	.	.	.	.	.	.	.	0.93 (0.59-1.47)	.	.	.	.	.
0.98 (0.44-2.19)	0.86 (0.45-1.67)	0.86 (0.43-1.74)	**DIC**	.	.	.	.	.	.	.	1.07 (0.63-1.83)	.	.	.	.	.
0.73 (0.39-1.39)	0.64 (0.41-1.00)	0.64 (0.38-1.07)	0.74 (0.42-1.32)	**ELE**	.	.	.	.	1.60 (0.84-3.05)	.	1.49 (1.14-1.96)	.	.	1.04 (0.68-1.60)	.	1.18 (0.73-1.89)
1.24 (0.63-2.46)	1.09 (0.65-1.81)	1.09 (0.62-1.92)	1.26 (0.67-2.36)	1.70 (1.14-2.53)	**FRO**	.	.	.	.	.	0.64 (0.42-0.98)	.	.	1.13 (0.68-1.89)	.	.
1.14 (0.58-2.26)	1.00 (0.60-1.67)	1.00 (0.57-1.76)	1.16 (0.62-2.17)	1.57 (1.05-2.33)	0.92 (0.58-1.47)	**IBU**	.	.	.	.	0.92 (0.67-1.28)	.	.	.	.	.
0.40 (0.21-0.75)	0.35 (0.22-0.54)	0.35 (0.21-0.58)	0.40 (0.23-0.71)	0.54 (0.40-0.74)	0.32 (0.21-0.47)	0.35 (0.23-0.51)	**LAS**	.	.	.	2.67 (2.15-3.32)	.	.	.	.	.
1.02 (0.52-1.99)	0.89 (0.55-1.45)	0.90 (0.52-1.54)	1.04 (0.56-1.90)	1.39 (0.97-2.01)	0.82 (0.53-1.27)	0.89 (0.57-1.38)	2.58 (1.79-3.72)	**NAP**	.	.	1.04 (0.75-1.45)	.	1.00 (0.05-18.75)	0.90 (0.63-1.28)	.	.
1.26 (0.61-2.63)	1.11 (0.62-1.97)	1.11 (0.59-2.07)	1.28 (0.65-2.53)	1.73 (1.11-2.70)	1.02 (0.60-1.74)	1.10 (0.65-1.88)	3.19 (1.99-5.14)	1.24 (0.74-2.07)	**NAR**	.	0.76 (0.47-1.23)	.	0.58 (0.17-1.91)	2.89 (0.13-62.19)	.	.
1.24 (0.53-2.88)	1.08 (0.53-2.22)	1.09 (0.51-2.31)	1.26 (0.56-2.80)	1.69 (0.89-3.21)	1.00 (0.50-1.98)	1.08 (0.55-2.14)	3.13 (1.65-5.92)	1.21 (0.62-2.36)	0.98 (0.47-2.04)	**PAR**	0.79 (0.42-1.46)	.	1.00 (0.30-3.30)	.	.	.
1.06 (0.58-1.92)	0.93 (0.63-1.37)	0.93 (0.59-1.47)	1.07 (0.63-1.83)	1.45 (1.15-1.81)	0.85 (0.61-1.19)	0.92 (0.67-1.28)	2.67 (2.15-3.32)	1.04 (0.77-1.39)	0.84 (0.55-1.28)	0.86 (0.47-1.56)	**placebo**	0.96 (0.60-1.56)	0.81 (0.67-0.98)	0.83 (0.73-0.96)	0.75 (0.51-1.11)	0.71 (0.57-0.89)
1.01 (0.47-2.18)	0.89 (0.48-1.64)	0.89 (0.46-1.72)	1.03 (0.50-2.10)	1.39 (0.82-2.35)	0.82 (0.46-1.46)	0.89 (0.50-1.58)	2.56 (1.52-4.32)	0.99 (0.57-1.74)	0.80 (0.42-1.52)	0.82 (0.38-1.76)	0.96 (0.59-1.54)	**RIM**	.	0.70 (0.23-2.14)	.	.
0.90 (0.49-1.68)	0.79 (0.52-1.21)	0.79 (0.49-1.29)	0.92 (0.53-1.61)	1.24 (0.94-1.63)	0.73 (0.51-1.05)	0.79 (0.55-1.14)	2.29 (1.74-3.01)	0.89 (0.64-1.23)	0.72 (0.46-1.12)	0.73 (0.40-1.35)	0.86 (0.72-1.01)	0.89 (0.54-1.48)	**RIZ**	0.97 (0.75-1.27)	.	0.81 (0.37-1.81)
0.93 (0.50-1.71)	0.81 (0.55-1.20)	0.81 (0.51-1.31)	0.94 (0.55-1.63)	1.27 (0.99-1.62)	0.75 (0.53-1.05)	0.81 (0.57-1.15)	2.35 (1.83-3.01)	0.91 (0.68-1.22)	0.73 (0.47-1.14)	0.75 (0.41-1.38)	0.88 (0.78-0.99)	0.92 (0.56-1.50)	1.03 (0.86-1.23)	**SUM**	.	.
0.79 (0.39-1.62)	0.69 (0.40-1.21)	0.70 (0.38-1.27)	0.80 (0.42-1.56)	1.08 (0.69-1.70)	0.64 (0.38-1.07)	0.69 (0.42-1.15)	2.00 (1.28-3.13)	0.78 (0.48-1.27)	0.63 (0.35-1.12)	0.64 (0.31-1.31)	0.75 (0.51-1.11)	0.78 (0.42-1.45)	0.88 (0.57-1.34)	0.85 (0.57-1.29)	**UBR**	.
0.76 (0.40-1.43)	0.67 (0.43-1.02)	0.67 (0.40-1.11)	0.77 (0.44-1.37)	1.04 (0.79-1.38)	0.61 (0.42-0.91)	0.67 (0.45-0.98)	1.92 (1.42-2.60)	0.75 (0.52-1.07)	0.60 (0.38-0.96)	0.62 (0.33-1.16)	0.72 (0.58-0.89)	0.75 (0.45-1.26)	0.84 (0.65-1.09)	0.82 (0.65-1.04)	0.96 (0.62-1.50)	**ZOL**

The network meta-analysis results are in the purple cells (direct estimates in the white cells). Participants with dizziness during the trial are reported for each comparison as odds ratio (OR), along with 95% confidence intervals. Coloured bottom left triangle: estimates below 1 favours the medication defined by the column. White top right triangle: estimates below 1 favours the medication defined by the row. ASA = Acetylsalicylic acid; ALM = Almotriptan; CEL = Celecoxib; DIC = Diclofenac; ELE = Eletriptan; FRO = Frovatriptan; IBU = Ibuprofen; LAS = Lasmiditan; NAP = Naproxen sodium; NAR = Naratriptan; PAR = Paracetamol; RIM = Rimegepant; RIZ = Rizatriptan; SUM = Sumatriptan; UBR = Ubrogepant; ZOL = Zolmitriptan.

Drug classes:

**antipyretic**	.	.	.	0.79 (0.42-1.47)	1.00 (0.30-3.31)
0.32 (0.17-0.61)	**ditan**	.	.	2.68 (2.15-3.35)	.
0.71 (0.36-1.39)	2.22 (1.52-3.24)	**gepant**	.	1.21 (0.89-1.64)	0.70 (0.23-2.15)
0.86 (0.46-1.61)	2.69 (2.02-3.58)	1.21 (0.85-1.73)	**NSAID**	0.99 (0.82-1.19)	0.90 (0.63-1.29)
0.86 (0.47-1.57)	2.68 (2.15-3.35)	1.21 (0.89-1.64)	1.00 (0.83-1.19)	**placebo**	0.86 (0.78-0.94)
0.73 (0.40-1.35)	2.29 (1.80-2.92)	1.03 (0.75-1.43)	0.85 (0.70-1.04)	0.86 (0.78-0.94)	**triptan**

The network meta-analysis results are in the purple cells (direct estimates in the white cells). Participants with dizziness during the trial are reported for each comparison as odds ratio (OR), along with 95% confidence intervals. Coloured bottom left triangle: estimates below 1 favours the drug class defined by the column. White top right triangle: estimates below 1 favours the drug class defined by the row. NSAID = non-steroidal anti-inflammatory drug.

8.13 Adverse events – Dry mouth

Individual drugs:

**ASA**	.	.	.	.	.	.	.	.	.	.	1.03<br>(0.61-1.72)	.	.	.	.
1.28<br>(0.60-2.73)	**ALM**	.	.	.	.	.	.	.	.	.	0.92<br>(0.51-1.66)	.	.	.	0.25<br>(0.05-1.18)
0.97<br>(0.50-1.90)	0.76<br>(0.38-1.53)	**DIC**	.	.	.	.	.	.	.	.	1.06<br>(0.69-1.61)	.	.	.	.
0.21<br>(0.05-0.83)	0.16<br>(0.04-0.65)	0.21<br>(0.05-0.82)	**ELE**	.	.	.	.	.	.	.	5.00<br>(1.37-18.24)	.	.	.	.
0.80<br>(0.40-1.59)	0.62<br>(0.30-1.28)	0.82<br>(0.44-1.53)	3.89<br>(0.99-15.32)	**FRO**	.	.	.	.	.	.	.	.	1.16<br>(0.75-1.79)	.	.
1.01<br>(0.54-1.89)	0.79<br>(0.41-1.52)	1.04<br>(0.60-1.80)	4.92<br>(1.29-18.81)	1.27<br>(0.71-2.25)	**IBU**	.	.	.	.	.	1.02<br>(0.71-1.45)	.	.	.	.
0.93<br>(0.54-1.60)	0.73<br>(0.41-1.30)	0.96<br>(0.61-1.51)	4.54<br>(1.23-16.71)	1.17<br>(0.72-1.90)	0.92<br>(0.62-1.36)	**LAS**	.	.	.	.	1.10<br>(0.93-1.30)	.	.	.	.
0.99<br>(0.56-1.76)	0.77<br>(0.42-1.42)	1.02<br>(0.62-1.66)	4.82<br>(1.29-17.98)	1.24<br>(0.75-2.05)	0.98<br>(0.64-1.51)	1.06<br>(0.79-1.43)	**NAP**	.	.	.	1.08<br>(0.82-1.43)	.	0.92<br>(0.69-1.24)	.	.
0.95<br>(0.42-2.17)	0.75<br>(0.32-1.73)	0.98<br>(0.46-2.11)	4.65<br>(1.10-19.65)	1.20<br>(0.55-2.61)	0.94<br>(0.46-1.96)	1.02<br>(0.53-1.98)	0.96<br>(0.49-1.91)	**NAR**	.	.	1.09<br>(0.56-2.12)	.	0.95<br>(0.12-7.38)	.	.
0.75<br>(0.28-2.02)	0.59<br>(0.22-1.61)	0.78<br>(0.30-1.98)	3.68<br>(0.79-17.14)	0.95<br>(0.37-2.44)	0.75<br>(0.30-1.85)	0.81<br>(0.35-1.90)	0.76<br>(0.32-1.82)	0.79<br>(0.28-2.26)	**PAR**	.	1.19<br>(0.48-2.92)	1.40<br>(0.44-4.45)	.	.	.
1.10<br>(0.44-2.76)	0.86<br>(0.34-2.20)	1.13<br>(0.48-2.70)	5.37<br>(1.20-24.02)	1.38<br>(0.57-3.34)	1.09<br>(0.47-2.51)	1.18<br>(0.55-2.57)	1.11<br>(0.50-2.47)	1.16<br>(0.43-3.11)	1.46<br>(0.47-4.51)	**PHE**	0.93<br>(0.44-1.99)	.	.	.	.
1.03<br>(0.61-1.72)	0.80<br>(0.46-1.40)	1.06<br>(0.69-1.61)	5.00<br>(1.37-18.24)	1.29<br>(0.82-2.03)	1.02<br>(0.71-1.45)	1.10<br>(0.93-1.30)	1.04<br>(0.81-1.33)	1.08<br>(0.57-2.04)	1.36<br>(0.59-3.14)	0.93<br>(0.44-1.99)	**placebo**	0.95<br>(0.79-1.14)	0.87<br>(0.75-1.00)	0.80<br>(0.61-1.07)	0.84<br>(0.64-1.10)
0.98<br>(0.57-1.69)	0.77<br>(0.43-1.37)	1.01<br>(0.64-1.59)	4.78<br>(1.30-17.61)	1.23<br>(0.77-1.97)	0.97<br>(0.66-1.43)	1.05<br>(0.84-1.33)	0.99<br>(0.75-1.32)	1.03<br>(0.53-1.98)	1.30<br>(0.56-3.02)	0.89<br>(0.41-1.93)	0.96<br>(0.81-1.12)	**RIZ**	0.97<br>(0.77-1.20)	.	.
0.92<br>(0.54-1.57)	0.72<br>(0.41-1.27)	0.95<br>(0.61-1.48)	4.49<br>(1.22-16.48)	1.16<br>(0.75-1.79)	0.91<br>(0.63-1.33)	0.99<br>(0.80-1.22)	0.93<br>(0.73-1.20)	0.97<br>(0.51-1.85)	1.22<br>(0.53-2.84)	0.84<br>(0.39-1.80)	0.90<br>(0.79-1.02)	0.94<br>(0.79-1.11)	**SUM**	.	.
0.83<br>(0.46-1.49)	0.65<br>(0.35-1.20)	0.85<br>(0.51-1.41)	4.02<br>(1.07-15.12)	1.03<br>(0.60-1.77)	0.82<br>(0.52-1.29)	0.89<br>(0.64-1.23)	0.83<br>(0.57-1.22)	0.87<br>(0.43-1.74)	1.09<br>(0.45-2.64)	0.75<br>(0.33-1.68)	0.80<br>(0.61-1.07)	0.84<br>(0.61-1.16)	0.90<br>(0.66-1.22)	**UBR**	.
0.84<br>(0.47-1.50)	0.65<br>(0.36-1.19)	0.86<br>(0.52-1.42)	4.08<br>(1.09-15.27)	1.05<br>(0.62-1.78)	0.83<br>(0.53-1.29)	0.90<br>(0.66-1.23)	0.85<br>(0.59-1.22)	0.88<br>(0.44-1.75)	1.11<br>(0.46-2.66)	0.76<br>(0.34-1.69)	0.82<br>(0.63-1.06)	0.85<br>(0.63-1.16)	0.91<br>(0.68-1.22)	1.01<br>(0.69-1.49)	**ZOL**

The network meta-analysis results are in the purple cells (direct estimates in the white cells). Participants with dry mouth during the trial are reported for each comparison as odds ratio (OR), along with 95% confidence intervals. Coloured bottom left triangle: estimates below 1 favours the medication defined by the column. White top right triangle: estimates below 1 favours the medication defined by the row. ASA = Acetylsalicylic acid; ALM = Almotriptan; DIC = Diclofenac; ELE = Eletriptan; FRO = Frovatriptan; IBU = Ibuprofen; LAS = Lasmiditan; NAP = Naproxen sodium; NAR = Naratriptan; PAR = Paracetamol; PHE = Phenazone; RIZ = Rizatriptan; SUM = Sumatriptan; UBR = Ubrogepant; ZOL = Zolmitriptan.

Drug classes:

**antipyretic**	.	.	.	1.19 (0.48-2.93)	1.40 (0.44-4.46)
1.25 (0.54-2.94)	**ditan**	.	.	1.10 (0.93-1.31)	.
1.11 (0.46-2.69)	0.89 (0.64-1.24)	**gepant**	.	1.24 (0.93-1.66)	.
1.35 (0.58-3.16)	1.07 (0.85-1.37)	1.21 (0.87-1.69)	**NSAID**	1.04 (0.87-1.25)	0.92 (0.69-1.24)
1.39 (0.60-3.20)	1.10 (0.93-1.31)	1.24 (0.93-1.66)	1.03 (0.87-1.22)	**placebo**	0.90 (0.81-1.00)
1.25 (0.54-2.89)	1.00 (0.82-1.22)	1.12 (0.83-1.52)	0.93 (0.77-1.12)	0.90 (0.81-1.00)	**triptan**

The network meta-analysis results are in the purple cells (direct estimates in the white cells). Participants with dry mouth during the trial are reported for each comparison as odds ratio (OR), along with 95% confidence intervals. Coloured bottom left triangle: estimates below 1 favours the drug class defined by the column. White top right triangle: estimates below 1 favours the drug class defined by the row. NSAID = non-steroidal anti-inflammatory drug.

8.14 Adverse events – Dyspepsia

Individual drugs:

**ASA**	.	.	.	.	.	.	.	0.96 (0.56-1.66)	.	.
1.19 (0.52-2.70)	**ALM**	.	.	.	.	.	.	0.81 (0.44-1.51)	.	.
0.86 (0.43-1.76)	0.73 (0.34-1.57)	**DIC**	.	.	.	.	.	1.12 (0.71-1.76)	.	.
1.01 (0.55-1.87)	0.85 (0.43-1.69)	1.17 (0.69-2.00)	**IBU**	.	.	.	.	0.95 (0.72-1.27)	.	.
0.90 (0.50-1.61)	0.76 (0.40-1.45)	1.04 (0.63-1.71)	0.89 (0.63-1.26)	**LAS**	.	.	.	1.07 (0.88-1.31)	.	.
0.83 (0.45-1.52)	0.70 (0.36-1.37)	0.96 (0.56-1.63)	0.82 (0.55-1.21)	0.92 (0.66-1.29)	**NAP**	.	.	1.19 (0.88-1.61)	.	0.99 (0.72-1.36)
0.96 (0.39-2.33)	0.81 (0.32-2.06)	1.11 (0.48-2.55)	0.94 (0.44-2.01)	1.06 (0.51-2.21)	1.16 (0.54-2.45)	**NAR**	.	1.01 (0.50-2.03)	.	.
1.17 (0.48-2.83)	0.98 (0.39-2.50)	1.35 (0.59-3.10)	1.15 (0.54-2.44)	1.30 (0.63-2.68)	1.41 (0.67-2.98)	1.22 (0.45-3.28)	**PAR**	0.83 (0.41-1.66)	.	.
0.96 (0.56-1.66)	0.81 (0.44-1.51)	1.12 (0.71-1.76)	0.95 (0.72-1.27)	1.07 (0.88-1.31)	1.16 (0.89-1.53)	1.01 (0.50-2.03)	0.83 (0.41-1.66)	**placebo**	1.01 (0.73-1.39)	0.83 (0.70-1.00)
1.01 (0.55-1.85)	0.85 (0.44-1.67)	1.17 (0.69-1.97)	1.00 (0.68-1.47)	1.12 (0.81-1.56)	1.22 (0.86-1.74)	1.06 (0.50-2.23)	0.87 (0.41-1.82)	1.05 (0.81-1.36)	**RIZ**	0.85 (0.63-1.16)
0.83 (0.47-1.47)	0.70 (0.37-1.33)	0.96 (0.59-1.56)	0.82 (0.59-1.14)	0.92 (0.71-1.20)	1.00 (0.76-1.32)	0.87 (0.42-1.79)	0.71 (0.35-1.46)	0.86 (0.73-1.02)	0.82 (0.64-1.06)	**SUM**

The network meta-analysis results are in the purple cells (direct estimates in the white cells). Participants with dyspepsia during the trial are reported for each comparison as odds ratio (OR), along with 95% confidence intervals. Coloured bottom left triangle: estimates below 1 favours the medication defined by the column. White top right triangle: estimates below 1 favours the medication defined by the row. ASA = Acetylsalicylic acid; ALM = Almotriptan; DIC = Diclofenac; IBU = Ibuprofen; LAS = Lasmiditan; NAP = Naproxen sodium; NAR = Naratriptan; PAR = Paracetamol; RIZ = Rizatriptan; SUM = Sumatriptan.

Drug classes:

**antipyretic**	.	.	0.82 (0.41-1.64)	.
0.77 (0.38-1.58)	**ditan**	.	1.07 (0.88-1.30)	.
0.79 (0.39-1.60)	1.02 (0.79-1.32)	**NSAID**	1.06 (0.89-1.26)	0.99 (0.73-1.35)
0.82 (0.41-1.64)	1.07 (0.88-1.30)	1.04 (0.88-1.23)	**placebo**	0.92 (0.79-1.07)
0.77 (0.38-1.56)	1.00 (0.78-1.27)	0.97 (0.80-1.19)	0.93 (0.81-1.08)	**triptan**

The network meta-analysis results are in the purple cells (direct estimates in the white cells). Participants with dyspepsia during the trial are reported for each comparison as odds ratio (OR), along with 95% confidence intervals. Coloured bottom left triangle: estimates below 1 favours the drug class defined by the column. White top right triangle: estimates below 1 favours the drug class defined by the row. NSAID = non-steroidal anti-inflammatory drug.

8.15 Adverse events – Fatigue

Individual drugs:

**ASA**	.	.	.	.	.	.	.	.	.	1.02 (0.55-1.88)	.	.	.	.
1.65 (0.74-3.67)	**ALM**	.	.	.	.	.	.	.	.	0.77 (0.40-1.50)	.	0.13 (0.02-0.76)	.	0.51 (0.22-1.19)
0.90 (0.39-2.08)	0.55 (0.26-1.17)	**DIC**	.	.	.	.	.	.	.	1.12 (0.64-1.97)	.	.	.	.
0.62 (0.32-1.21)	0.38 (0.22-0.66)	0.69 (0.38-1.27)	**ELE**	.	.	.	1.55 (0.78-3.08)	.	.	1.84 (1.39-2.44)	.	1.01 (0.65-1.57)	.	1.31 (0.80-2.14)
1.06 (0.51-2.22)	0.64 (0.34-1.23)	1.17 (0.59-2.34)	1.70 (1.07-2.70)	**FRO**	.	.	.	.	.	0.76 (0.42-1.38)	.	0.96 (0.56-1.65)	.	.
0.65 (0.34-1.26)	0.40 (0.23-0.69)	0.72 (0.39-1.32)	1.05 (0.75-1.45)	0.62 (0.39-0.98)	**LAS**	.	.	.	.	1.55 (1.23-1.96)	.	.	.	.
0.86 (0.39-1.89)	0.52 (0.26-1.05)	0.95 (0.45-2.00)	1.37 (0.80-2.35)	0.81 (0.43-1.52)	1.31 (0.77-2.26)	**NAP**	.	.	.	1.03 (0.59-1.79)	.	1.14 (0.65-2.01)	.	.
1.15 (0.54-2.44)	0.70 (0.36-1.36)	1.28 (0.63-2.58)	1.85 (1.17-2.91)	1.09 (0.60-1.96)	1.77 (1.09-2.87)	1.34 (0.70-2.57)	**NAR**	.	.	0.82 (0.50-1.35)	0.68 (0.21-2.23)	0.42 (0.09-2.00)	.	.
1.38 (0.56-3.37)	0.84 (0.37-1.91)	1.52 (0.65-3.59)	2.21 (1.11-4.39)	1.30 (0.61-2.79)	2.11 (1.06-4.19)	1.61 (0.71-3.61)	1.19 (0.55-2.59)	**PAR**	.	0.69 (0.35-1.34)	0.84 (0.24-2.89)	.	.	.
1.02 (0.37-2.82)	0.62 (0.24-1.61)	1.12 (0.42-3.02)	1.63 (0.70-3.79)	0.96 (0.39-2.38)	1.55 (0.67-3.62)	1.18 (0.46-3.06)	0.88 (0.35-2.21)	0.74 (0.26-2.08)	**PHE**	1.00 (0.44-2.26)	.	.	.	.
1.02 (0.55-1.88)	0.62 (0.37-1.03)	1.12 (0.64-1.97)	1.63 (1.29-2.05)	0.96 (0.64-1.44)	1.55 (1.23-1.96)	1.18 (0.73-1.93)	0.88 (0.57-1.35)	0.74 (0.39-1.41)	1.00 (0.44-2.26)	**placebo**	0.84 (0.67-1.05)	0.83 (0.71-0.96)	0.73 (0.39-1.37)	0.82 (0.66-1.01)
0.92 (0.48-1.75)	0.56 (0.32-0.96)	1.02 (0.56-1.84)	1.47 (1.10-1.97)	0.87 (0.56-1.35)	1.41 (1.04-1.90)	1.07 (0.64-1.80)	0.80 (0.50-1.26)	0.67 (0.34-1.30)	0.90 (0.39-2.09)	0.90 (0.75-1.10)	**RIZ**	0.88 (0.66-1.17)	.	1.27 (0.57-2.83)
0.84 (0.45-1.59)	0.51 (0.30-0.86)	0.93 (0.53-1.66)	1.35 (1.05-1.74)	0.80 (0.53-1.19)	1.29 (0.99-1.69)	0.98 (0.60-1.60)	0.73 (0.47-1.14)	0.61 (0.32-1.18)	0.83 (0.36-1.90)	0.83 (0.73-0.95)	0.92 (0.75-1.13)	**SUM**	.	1.00 (0.63-1.61)
0.74 (0.31-1.79)	0.45 (0.20-1.01)	0.82 (0.35-1.91)	1.19 (0.61-2.33)	0.70 (0.33-1.48)	1.13 (0.58-2.22)	0.86 (0.39-1.92)	0.64 (0.30-1.38)	0.54 (0.22-1.33)	0.73 (0.26-2.05)	0.73 (0.39-1.37)	0.81 (0.42-1.56)	0.88 (0.46-1.68)	**UBR**	.
0.85 (0.44-1.62)	0.51 (0.31-0.87)	0.94 (0.52-1.69)	1.36 (1.03-1.79)	0.80 (0.51-1.25)	1.30 (0.96-1.75)	0.99 (0.59-1.66)	0.74 (0.46-1.17)	0.62 (0.31-1.21)	0.84 (0.36-1.93)	0.84 (0.69-1.01)	0.92 (0.71-1.20)	1.01 (0.81-1.25)	1.14 (0.59-2.21)	**ZOL**

The network meta-analysis results are in the purple cells (direct estimates in the white cells). The proportion of participants with fatigue during the trial are reported for each comparison as odds ratio (OR), along with 95% confidence intervals. Coloured bottom left triangle: estimates below 1 favours the medication defined by the column. White top right triangle: estimates below 1 favours the medication defined by the row. ASA = Acetylsalicylic acid; ALM = Almotriptan; DIC = Diclofenac; ELE = Eletriptan; FRO = Frovatriptan;

LAS = Lasmiditan; NAP = Naproxen sodium; NAR = Naratriptan; PAR = Paracetamol; PHE = Phenazone; RIZ = Rizatriptan; SUM = Sumatriptan; UBR = Ubrogepant; ZOL = Zolmitriptan.

Drug classes:

**antipyretic**	.	.	.	0.69 (0.34-1.38)	0.84 (0.24-2.97)
0.47 (0.23-0.98)	**ditan**	.	.	1.57 (1.21-2.04)	.
0.54 (0.21-1.42)	1.15 (0.55-2.39)	**gepant**	.	1.37 (0.69-2.71)	.
0.68 (0.32-1.43)	1.44 (0.95-2.17)	1.25 (0.59-2.66)	**NSAID**	1.04 (0.75-1.46)	1.14 (0.61-2.13)
0.75 (0.38-1.46)	1.57 (1.21-2.04)	1.37 (0.69-2.71)	1.10 (0.79-1.51)	**placebo**	0.84 (0.75-0.94)
0.63 (0.32-1.24)	1.33 (1.00-1.76)	1.15 (0.58-2.31)	0.92 (0.66-1.29)	0.84 (0.75-0.94)	**triptan**

The network meta-analysis results are in the purple cells (direct estimates in the white cells). The proportion of participants with fatigue during the trial are reported for each comparison as odds ratio (OR), along with 95% confidence intervals. Coloured bottom left triangle: estimates below 1 favours the drug class defined by the column. White top right triangle: estimates below 1 favours the drug class defined by the row. NSAID = non-steroidal anti-inflammatory drug.

8.16 Adverse events – Gastrointestinal bleeding

Sufficient outcome data not available for analysis.

8.17 Adverse events – Hepatic toxicity

Individual drugs:

**CEL**	.	.	0.86 (0.58-1.27)	.	.	.	.	.
0.75 (0.49-1.16)	**LAS**	.	1.14 (0.95-1.37)	.	.	.	.	.
0.13 (0.02-0.80)	0.18 (0.03-1.03)	**PAR**	6.40 (1.12-36.57)	.	.	.	.	.
0.86 (0.58-1.27)	1.14 (0.95-1.37)	6.40 (1.12-36.57)	**placebo**	0.90 (0.71-1.14)	1.08 (0.74-1.59)	0.96 (0.67-1.37)	0.82 (0.68-0.99)	1.35 (0.76-2.41)
0.77 (0.49-1.22)	1.03 (0.76-1.38)	5.77 (0.99-33.50)	0.90 (0.71-1.14)	**RIM**	.	0.78 (0.27-2.31)	.	.
1.01 (0.60-1.69)	1.35 (0.92-1.97)	7.55 (1.28-44.57)	1.18 (0.84-1.65)	1.31 (0.87-1.97)	**RIZ**	0.88 (0.62-1.27)	.	.
0.89 (0.53-1.48)	1.18 (0.81-1.72)	6.65 (1.13-39.17)	1.04 (0.75-1.44)	1.15 (0.77-1.71)	0.88 (0.64-1.21)	**SUM**	.	1.00 (0.74-1.36)
0.70 (0.46-1.09)	0.94 (0.72-1.22)	5.26 (0.91-30.38)	0.82 (0.68-0.99)	0.91 (0.68-1.23)	0.70 (0.47-1.02)	0.79 (0.54-1.15)	**UBR**	.
0.92 (0.52-1.63)	1.23 (0.79-1.92)	6.91 (1.15-41.39)	1.08 (0.72-1.62)	1.20 (0.75-1.91)	0.92 (0.60-1.39)	1.04 (0.77-1.40)	1.31 (0.84-2.05)	**ZOL**

The network meta-analysis results are in the purple cells (direct estimates in the white cells). Participants with hepatic toxicity during the trial are reported for each comparison as odds ratio (OR), along with 95% confidence intervals. Coloured bottom left triangle: estimates below 1 favours the medication defined by the column. White top right triangle: estimates below 1 favours the medication defined by the row. CEL = Celecoxib; LAS = Lasmiditan; PAR = Paracetamol; RIM = Rimegepant; RIZ = Rizatriptan; SUM = Sumatriptan; UBR = Ubrogepant; ZOL = Zolmitriptan.

Drug classes:

**antipyretic**	.	.	.	6.40 (1.12-36.49)	.
5.65 (0.98-32.47)	**ditan**	.	.	1.13 (0.95-1.35)	.
5.46 (0.95-31.31)	0.97 (0.77-1.21)	**gepant**	.	1.17 (1.02-1.35)	0.78 (0.27-2.30)
7.48 (1.26-44.54)	1.33 (0.87-2.03)	1.37 (0.91-2.07)	**NSAID**	0.86 (0.58-1.26)	.
6.40 (1.12-36.49)	1.13 (0.95-1.35)	1.17 (1.02-1.35)	0.86 (0.58-1.26)	**placebo**	1.12 (0.84-1.50)
7.09 (1.21-41.36)	1.26 (0.90-1.76)	1.30 (0.95-1.78)	0.95 (0.58-1.53)	1.11 (0.83-1.47)	**triptan**

The network meta-analysis results are in the purple cells (direct estimates in the white cells). Participants with hepatic toxicity during the trial are reported for each comparison as odds ratio (OR), along with 95% confidence intervals. Coloured bottom left triangle: estimates below 1 favours the drug class defined by the column. White top right triangle: estimates below 1 favours the drug class defined by the row. NSAID = non-steroidal anti-inflammatory drug.

8.18 Adverse events – Major adverse cardiovascular events

Sufficient outcome data not available for analysis.

8.19 Adverse events – Nausea

Individual drugs:

**ASA**	.	.	.	.	.	.	.	.	.	.	.	1.12 (0.70-1.78)	.	.	.	.	.
1.08 (0.62-1.87)	**ALM**	.	.	.	.	.	.	.	.	.	.	0.90 (0.52-1.56)	.	.	0.86 (0.59-1.26)	.	1.28 (0.57-2.84)
1.21 (0.68-2.17)	1.13 (0.71-1.78)	**CEL**	.	.	.	.	.	.	.	.	.	0.92 (0.65-1.31)	.	.	.	.	.
0.88 (0.49-1.58)	0.82 (0.52-1.30)	0.73 (0.44-1.19)	**DIC**	.	.	.	.	.	.	.	.	1.27 (0.89-1.80)	.	.	.	.	.
0.99 (0.60-1.64)	0.92 (0.64-1.31)	0.82 (0.54-1.22)	1.12 (0.75-1.68)	**ELE**	.	.	.	.	1.10 (0.69-1.75)	.	.	1.17 (0.91-1.49)	.	.	0.92 (0.66-1.28)	.	.
1.26 (0.74-2.13)	1.17 (0.80-1.71)	1.04 (0.68-1.59)	1.42 (0.93-2.19)	1.27 (0.93-1.75)	**FRO**	.	.	.	.	.	.	0.78 (0.56-1.10)	.	.	0.86 (0.61-1.22)	.	.
1.28 (0.77-2.11)	1.19 (0.83-1.69)	1.05 (0.71-1.57)	1.45 (0.97-2.16)	1.29 (0.97-1.71)	1.02 (0.74-1.39)	**IBU**	.	.	.	.	.	0.88 (0.72-1.06)	.	.	.	.	.
0.90 (0.55-1.45)	0.83 (0.60-1.15)	0.74 (0.51-1.07)	1.02 (0.70-1.48)	0.91 (0.71-1.16)	0.71 (0.54-0.94)	0.70 (0.56-0.89)	**LAS**	.	.	.	.	1.25 (1.10-1.42)	.	.	.	.	.
1.01 (0.61-1.69)	0.94 (0.65-1.35)	0.83 (0.55-1.26)	1.15 (0.76-1.74)	1.02 (0.76-1.38)	0.81 (0.58-1.11)	0.79 (0.59-1.06)	1.13 (0.87-1.46)	**NAP**	.	.	.	1.14 (0.89-1.46)	.	0.45 (0.03-5.84)	0.90 (0.70-1.17)	.	.
1.24 (0.70-2.20)	1.15 (0.74-1.80)	1.02 (0.63-1.66)	1.41 (0.87-2.28)	1.26 (0.89-1.78)	0.99 (0.66-1.49)	0.97 (0.66-1.43)	1.38 (0.97-1.97)	1.23 (0.83-1.82)	**NAR**	.	.	0.80 (0.55-1.16)	.	0.46 (0.11-1.87)	1.72 (0.18-16.78)	.	.
2.00 (1.07-3.73)	1.85 (1.11-3.10)	1.64 (0.95-2.84)	2.26 (1.31-3.90)	2.02 (1.27-3.22)	1.59 (0.98-2.58)	1.56 (0.99-2.48)	2.22 (1.44-3.44)	1.97 (1.23-3.16)	1.61 (0.94-2.73)	**PAR**	.	0.54 (0.35-0.82)	.	0.81 (0.23-2.87)	.	.	.
1.20 (0.50-2.86)	1.11 (0.50-2.47)	0.99 (0.44-2.23)	1.36 (0.60-3.07)	1.21 (0.57-2.61)	0.95 (0.44-2.07)	0.94 (0.44-2.01)	1.34 (0.63-2.82)	1.19 (0.55-2.55)	0.97 (0.43-2.16)	0.60 (0.26-1.40)	**PHE**	0.93 (0.45-1.94)	.	.	.	.	.
1.12 (0.70-1.78)	1.04 (0.77-1.40)	0.92 (0.65-1.31)	1.27 (0.89-1.80)	1.13 (0.92-1.39)	0.89 (0.70-1.14)	0.88 (0.72-1.06)	1.25 (1.10-1.42)	1.11 (0.89-1.38)	0.90 (0.65-1.25)	0.56 (0.37-0.85)	0.93 (0.45-1.94)	**placebo**	0.91 (0.73-1.13)	0.86 (0.70-1.05)	0.84 (0.75-0.93)	0.81 (0.67-0.98)	0.81 (0.68-0.97)
1.01 (0.61-1.69)	0.94 (0.65-1.36)	0.83 (0.55-1.26)	1.15 (0.76-1.73)	1.02 (0.76-1.38)	0.80 (0.58-1.12)	0.79 (0.59-1.06)	1.13 (0.88-1.45)	1.00 (0.73-1.36)	0.81 (0.55-1.21)	0.51 (0.32-0.81)	0.84 (0.39-1.81)	0.90 (0.73-1.12)	**RIM**	.	0.86 (0.33-2.23)	.	.
1.09 (0.67-1.78)	1.01 (0.73-1.41)	0.90 (0.61-1.32)	1.24 (0.84-1.82)	1.10 (0.86-1.42)	0.87 (0.65-1.15)	0.85 (0.67-1.10)	1.22 (0.99-1.49)	1.08 (0.83-1.40)	0.88 (0.61-1.26)	0.55 (0.35-0.85)	0.91 (0.43-1.93)	0.98 (0.83-1.14)	1.08 (0.83-1.41)	**RIZ**	0.81 (0.67-0.98)	.	.
0.94 (0.59-1.51)	0.88 (0.65-1.18)	0.78 (0.54-1.12)	1.07 (0.74-1.54)	0.95 (0.77-1.18)	0.75 (0.59-0.96)	0.74 (0.60-0.92)	1.05 (0.90-1.23)	0.93 (0.75-1.16)	0.76 (0.54-1.06)	0.47 (0.31-0.72)	0.79 (0.37-1.65)	0.84 (0.77-0.93)	0.93 (0.74-1.18)	0.86 (0.74-1.01)	**SUM**	.	1.05 (0.81-1.36)
0.91 (0.55-1.50)	0.84 (0.59-1.20)	0.75 (0.50-1.11)	1.03 (0.69-1.53)	0.92 (0.69-1.22)	0.72 (0.53-0.99)	0.71 (0.54-0.93)	1.01 (0.80-1.28)	0.89 (0.67-1.20)	0.73 (0.50-1.07)	0.45 (0.29-0.72)	0.75 (0.35-1.61)	0.81 (0.67-0.98)	0.90 (0.67-1.20)	0.83 (0.65-1.07)	0.96 (0.77-1.19)	**UBR**	.
0.94 (0.58-1.53)	0.87 (0.63-1.20)	0.77 (0.53-1.13)	1.06 (0.72-1.56)	0.95 (0.74-1.22)	0.75 (0.56-0.99)	0.73 (0.57-0.94)	1.04 (0.86-1.27)	0.93 (0.71-1.20)	0.75 (0.53-1.08)	0.47 (0.30-0.73)	0.78 (0.37-1.65)	0.84 (0.72-0.97)	0.93 (0.71-1.21)	0.86 (0.70-1.06)	0.99 (0.85-1.17)	1.03 (0.81-1.32)	**ZOL**

The network meta-analysis results are in the purple cells (direct estimates in the white cells). Participants with nausea during the trial are reported for each comparison as odds ratio (OR), along with 95% confidence intervals. Coloured bottom left triangle: estimates below 1 favours the medication defined by the column. White top right triangle: estimates below 1 favours the medication defined by the row. ASA = Acetylsalicylic acid; ALM = Almotriptan; CEL = Celecoxib; DIC = Diclofenac; ELE = Eletriptan; FRO = Frovatriptan; IBU = Ibuprofen; LAS = Lasmiditan; NAP = Naproxen sodium; NAR = Naratriptan; PAR = Paracetamol; PHE = Phenazone; RIM = Rimegepant; RIZ = Rizatriptan; SUM = Sumatriptan; UBR = Ubrogepant; ZOL = Zolmitriptan.

Drug classes:

**antipyretic**	.	.	.	0.54 (0.35-0.82)	0.81 (0.23-2.87)
0.45 (0.29-0.70)	**ditan**	.	.	1.25 (1.10-1.42)	.
0.48 (0.31-0.75)	1.06 (0.87-1.29)	**gepant**	.	1.17 (1.02-1.36)	0.86 (0.33-2.23)
0.57 (0.37-0.87)	1.25 (1.05-1.49)	1.18 (0.98-1.42)	**NSAID**	1.00 (0.89-1.14)	0.90 (0.70-1.17)
0.56 (0.37-0.86)	1.25 (1.10-1.42)	1.18 (1.02-1.36)	1.00 (0.88-1.12)	**placebo**	0.89 (0.82-0.96)
0.50 (0.33-0.77)	1.11 (0.95-1.29)	1.05 (0.89-1.23)	0.89 (0.77-1.01)	0.89 (0.82-0.96)	**triptan**

The network meta-analysis results are in the purple cells (direct estimates in the white cells). Participants with nausea during the trial are reported for each comparison as odds ratio (OR), along with 95% confidence intervals. Coloured bottom left triangle: estimates below 1 favours the drug class defined by the column. White top right triangle: estimates below 1 favours the drug class defined by the row. NSAID = non-steroidal anti-inflammatory drug.

8.20 Adverse events – Paraesthesia

Individual drugs:

**ASA**	.	.	.	.	.	.	.	.	1.03 (0.58-1.83)	.	.	.	.	.
1.04 (0.52-2.10)	**ALM**	.	.	.	.	.	.	.	0.86 (0.46-1.60)	.	.	0.92 (0.56-1.52)	.	.
0.88 (0.41-1.88)	0.84 (0.44-1.60)	**DIC**	.	.	.	.	.	.	1.17 (0.71-1.93)	.	.	.	.	.
1.18 (0.60-2.32)	1.13 (0.67-1.92)	1.34 (0.73-2.48)	**ELE**	.	.	.	.	.	0.92 (0.61-1.40)	.	.	0.76 (0.46-1.26)	.	.
1.09 (0.55-2.15)	1.04 (0.61-1.78)	1.24 (0.67-2.30)	0.92 (0.55-1.53)	**FRO**	.	.	.	.	0.75 (0.44-1.30)	.	.	0.96 (0.59-1.56)	.	.
1.10 (0.48-2.52)	1.06 (0.52-2.16)	1.26 (0.58-2.72)	0.94 (0.47-1.86)	1.02 (0.51-2.03)	**IBU**	.	.	.	0.93 (0.52-1.67)	.	.	.	.	.
0.69 (0.37-1.26)	0.66 (0.42-1.03)	0.78 (0.46-1.34)	0.58 (0.39-0.88)	0.63 (0.42-0.96)	0.62 (0.33-1.16)	**LAS**	.	.	1.50 (1.23-1.83)	.	.	.	.	.
0.94 (0.49-1.80)	0.90 (0.55-1.47)	1.07 (0.60-1.92)	0.80 (0.50-1.27)	0.86 (0.54-1.38)	0.85 (0.44-1.65)	1.37 (0.95-1.97)	**NAP**	.	1.18 (0.83-1.67)	.	0.45 (0.03-5.95)	0.90 (0.64-1.26)	.	.
1.17 (0.49-2.78)	1.12 (0.52-2.40)	1.33 (0.59-3.02)	0.99 (0.47-2.08)	1.08 (0.51-2.27)	1.06 (0.44-2.54)	1.70 (0.86-3.36)	1.24 (0.61-2.55)	**NAR**	1.01 (0.51-1.99)	.	.	0.24 (0.03-1.68)	.	.
1.03 (0.58-1.83)	0.98 (0.66-1.47)	1.17 (0.71-1.93)	0.87 (0.61-1.25)	0.95 (0.65-1.37)	0.93 (0.52-1.67)	1.50 (1.23-1.83)	1.09 (0.81-1.48)	0.88 (0.46-1.68)	**placebo**	1.83 (0.64-5.22)	0.88 (0.71-1.09)	0.83 (0.72-0.94)	0.75 (0.42-1.35)	0.77 (0.62-0.96)
1.81 (0.57-5.78)	1.73 (0.59-5.12)	2.06 (0.67-6.35)	1.53 (0.53-4.46)	1.67 (0.57-4.86)	1.64 (0.51-5.26)	2.63 (0.94-7.37)	1.93 (0.67-5.51)	1.55 (0.47-5.14)	1.76 (0.64-4.83)	**RIM**	.	0.52 (0.17-1.62)	.	.
0.95 (0.52-1.74)	0.91 (0.59-1.40)	1.08 (0.63-1.84)	0.80 (0.54-1.20)	0.87 (0.58-1.31)	0.86 (0.46-1.59)	1.38 (1.05-1.81)	1.01 (0.71-1.43)	0.81 (0.41-1.59)	0.92 (0.77-1.11)	0.52 (0.19-1.46)	**RIZ**	0.91 (0.70-1.19)	.	.
0.88 (0.49-1.58)	0.84 (0.56-1.25)	1.00 (0.60-1.67)	0.74 (0.52-1.07)	0.81 (0.56-1.16)	0.79 (0.44-1.44)	1.28 (1.01-1.61)	0.93 (0.69-1.26)	0.75 (0.39-1.45)	0.85 (0.76-0.96)	0.48 (0.18-1.33)	0.92 (0.76-1.12)	**SUM**	.	1.04 (0.70-1.56)
0.77 (0.34-1.76)	0.74 (0.37-1.51)	0.88 (0.41-1.90)	0.66 (0.33-1.30)	0.71 (0.36-1.42)	0.70 (0.31-1.60)	1.13 (0.61-2.09)	0.82 (0.43-1.59)	0.66 (0.28-1.59)	0.75 (0.42-1.35)	0.43 (0.13-1.37)	0.82 (0.44-1.50)	0.88 (0.49-1.60)	**UBR**	.
0.81 (0.44-1.48)	0.77 (0.50-1.20)	0.92 (0.54-1.57)	0.68 (0.46-1.03)	0.74 (0.49-1.12)	0.73 (0.39-1.36)	1.18 (0.89-1.56)	0.86 (0.60-1.23)	0.69 (0.35-1.36)	0.79 (0.65-0.96)	0.45 (0.16-1.25)	0.85 (0.65-1.11)	0.92 (0.74-1.15)	1.04 (0.56-1.93)	**ZOL**

The network meta-analysis results are in the purple cells (direct estimates in the white cells). Participants with paraesthesia during the trial are reported for each comparison as odds ratio (OR), along with 95% confidence intervals. Coloured bottom left triangle: estimates below 1 favours the medication defined by the column. White top right triangle: estimates below 1 favours the medication defined by the row. ASA = Acetylsalicylic acid; ALM = Almotriptan; DIC = Diclofenac; ELE = Eletriptan; FRO = Frovatriptan; IBU = Ibuprofen; LAS = Lasmiditan; NAP = Naproxen sodium; NAR = Naratriptan; RIM = Rimegepant; RIZ = Rizatriptan; SUM = Sumatriptan; UBR = Ubrogepant; ZOL = Zolmitriptan.

Drug classes:

**ditan**	.	.	1.50 (1.22-1.85)	.
1.41 (0.81-2.45)	**gepant**	.	1.07 (0.64-1.80)	0.52 (0.16-1.63)
1.41 (1.04-1.91)	1.00 (0.57-1.75)	**NSAID**	1.10 (0.87-1.41)	0.89 (0.63-1.26)
1.50 (1.22-1.85)	1.06 (0.64-1.78)	1.06 (0.85-1.33)	**placebo**	0.88 (0.80-0.97)
1.32 (1.05-1.66)	0.94 (0.56-1.58)	0.94 (0.74-1.18)	0.88 (0.80-0.97)	**triptan**

The network meta-analysis results are in the purple cells (direct estimates in the white cells). Participants with paraesthesia during the trial are reported for each comparison as odds ratio (OR), along with 95% confidence intervals. Coloured bottom left triangle: estimates below 1 favours the drug class defined by the column. White top right triangle: estimates below 1 favours the drug class defined by the row. NSAID = non-steroidal anti-inflammatory drug.

8.21 Adverse events – Pruritus

Individual drugs:

**ASA**	.	.	.	.	1.03 (0.46-2.31)	.
0.93 (0.31-2.82)	**DIC**	.	.	.	1.10 (0.52-2.35)	.
0.94 (0.25-3.50)	1.01 (0.28-3.64)	**ELE**	.	.	1.09 (0.39-3.04)	.
0.88 (0.33-2.32)	0.94 (0.37-2.38)	0.93 (0.29-2.97)	**LAS**	.	1.17 (0.68-2.01)	.
1.18 (0.35-4.03)	1.27 (0.39-4.18)	1.25 (0.32-4.98)	1.35 (0.46-3.91)	**NAR**	0.87 (0.35-2.18)	.
1.03 (0.46-2.31)	1.10 (0.52-2.35)	1.09 (0.39-3.04)	1.17 (0.68-2.01)	0.87 (0.35-2.18)	**placebo**	0.75 (0.42-1.34)
0.77 (0.28-2.08)	0.82 (0.32-2.14)	0.81 (0.25-2.65)	0.88 (0.40-1.93)	0.65 (0.22-1.92)	0.75 (0.42-1.34)	**SUM**

The network meta-analysis results are in the purple cells (direct estimates in the white cells). Participants with pruritus during the trial are reported for each comparison as odds ratio (OR), along with 95% confidence intervals. Coloured bottom left triangle: estimates below 1 favours the medication defined by the column. White top right triangle: estimates below 1 favours the medication defined by the row. ASA = Acetylsalicylic acid; DIC = Diclofenac; ELE = Eletriptan; LAS = Lasmiditan; NAR = Naratriptan; SUM = Sumatriptan.

Drug classes:

**ditan**	.	1.09 (0.81-1.48)	.
1.02 (0.64-1.63)	**NSAID**	1.07 (0.75-1.53)	.
1.09 (0.81-1.48)	1.07 (0.75-1.53)	**placebo**	0.89 (0.66-1.20)
0.98 (0.64-1.49)	0.95 (0.60-1.52)	0.89 (0.66-1.20)	**triptan**

The network meta-analysis results are in the purple cells (direct estimates in the white cells). Participants with pruritus during the trial are reported for each comparison as odds ratio (OR), along with 95% confidence intervals. Coloured bottom left triangle: estimates below 1 favours the drug class defined by the column. White top right triangle: estimates below 1 favours the drug class defined by the row. NSAID = non-steroidal anti-inflammatory drug.

8.22 Adverse events – Serotonergic syndrome

Sufficient outcome data not available for analysis.

8.23 Adverse events – Sedation

Individual drugs:

**ASA**	.	.	.	.	.	.	.	.	.	1.03<br>(0.58-1.82)	.	.	.	.
1.16<br>(0.58-2.31)	**ALM**	.	.	.	.	.	.	.	.	0.81<br>(0.43-1.50)	.	0.86<br>(0.53-1.42)	.	0.85<br>(0.27-2.67)
0.95<br>(0.45-2.03)	0.82<br>(0.44-1.53)	**DIC**	.	.	.	.	.	.	.	1.08<br>(0.66-1.76)	.	.	.	.
0.77<br>(0.41-1.42)	0.66<br>(0.43-1.02)	0.80<br>(0.47-1.39)	**ELE**	.	.	.	.	1.28<br>(0.70-2.34)	.	1.52<br>(1.15-2.01)	.	0.93<br>(0.60-1.44)	.	1.20<br>(0.78-1.84)
1.31<br>(0.68-2.54)	1.13<br>(0.69-1.84)	1.38<br>(0.76-2.49)	1.72<br>(1.16-2.54)	**FRO**	.	.	.	.	.	0.61<br>(0.41-0.92)	.	1.06<br>(0.64-1.76)	.	.
1.04<br>(0.54-2.00)	0.90<br>(0.55-1.46)	1.09<br>(0.61-1.95)	1.36<br>(0.93-2.00)	0.79<br>(0.51-1.24)	**IBU**	.	.	.	.	0.98<br>(0.73-1.34)	.	.	.	.
0.67<br>(0.37-1.23)	0.58<br>(0.38-0.88)	0.70<br>(0.41-1.20)	0.88<br>(0.65-1.19)	0.51<br>(0.35-0.75)	0.64<br>(0.45-0.93)	**LAS**	.	.	.	1.53<br>(1.25-1.86)	.	.	.	.
0.96<br>(0.50-1.83)	0.82<br>(0.51-1.32)	1.00<br>(0.56-1.79)	1.25<br>(0.86-1.81)	0.73<br>(0.47-1.12)	0.92<br>(0.60-1.41)	1.42<br>(0.99-2.04)	**NAP**	.	.	1.17<br>(0.83-1.65)	.	0.96<br>(0.69-1.35)	.	.
1.24<br>(0.57-2.69)	1.06<br>(0.56-2.02)	1.30<br>(0.63-2.67)	1.61<br>(0.96-2.71)	0.94<br>(0.51-1.74)	1.19<br>(0.65-2.18)	1.84<br>(1.05-3.23)	1.29<br>(0.71-2.36)	**NAR**	.	0.69<br>(0.37-1.31)	0.09<br>(0.01-0.72)	2.89<br>(0.14-61.76)	.	.
1.20<br>(0.54-2.71)	1.04<br>(0.52-2.05)	1.26<br>(0.59-2.69)	1.57<br>(0.85-2.91)	0.92<br>(0.48-1.77)	1.16<br>(0.61-2.21)	1.79<br>(0.98-3.28)	1.26<br>(0.66-2.40)	0.98<br>(0.45-2.12)	**PAR**	0.80<br>(0.44-1.44)	1.00<br>(0.31-3.24)	.	.	.
1.03<br>(0.58-1.82)	0.88<br>(0.61-1.29)	1.08<br>(0.66-1.76)	1.34<br>(1.06-1.69)	0.78<br>(0.57-1.08)	0.98<br>(0.73-1.34)	1.53<br>(1.25-1.86)	1.07<br>(0.80-1.45)	0.83<br>(0.49-1.40)	0.85<br>(0.48-1.51)	**placebo**	0.89<br>(0.74-1.07)	0.91<br>(0.80-1.05)	0.83<br>(0.59-1.16)	0.84<br>(0.68-1.02)
0.93<br>(0.51-1.68)	0.80<br>(0.53-1.19)	0.97<br>(0.58-1.63)	1.21<br>(0.92-1.59)	0.70<br>(0.49-1.01)	0.89<br>(0.63-1.26)	1.38<br>(1.07-1.78)	0.97<br>(0.69-1.35)	0.75<br>(0.43-1.29)	0.77<br>(0.43-1.38)	0.90<br>(0.77-1.06)	**RIZ**	1.01<br>(0.79-1.31)	.	1.60<br>(0.72-3.57)
0.96<br>(0.53-1.72)	0.83<br>(0.57-1.20)	1.01<br>(0.60-1.67)	1.25<br>(0.98-1.60)	0.73<br>(0.53-1.01)	0.92<br>(0.66-1.28)	1.43<br>(1.13-1.80)	1.00<br>(0.74-1.35)	0.78<br>(0.46-1.32)	0.80<br>(0.44-1.43)	0.93<br>(0.83-1.05)	1.04<br>(0.87-1.23)	**SUM**	.	0.94<br>(0.63-1.41)
0.85<br>(0.43-1.65)	0.73<br>(0.44-1.21)	0.89<br>(0.49-1.62)	1.11<br>(0.73-1.67)	0.65<br>(0.40-1.03)	0.81<br>(0.52-1.29)	1.26<br>(0.85-1.87)	0.89<br>(0.56-1.40)	0.69<br>(0.37-1.28)	0.70<br>(0.36-1.37)	0.83<br>(0.59-1.16)	0.92<br>(0.63-1.34)	0.88<br>(0.62-1.27)	**UBR**	.
0.89<br>(0.49-1.63)	0.77<br>(0.51-1.15)	0.94<br>(0.55-1.59)	1.17<br>(0.90-1.52)	0.68<br>(0.47-0.98)	0.86<br>(0.60-1.22)	1.33<br>(1.02-1.74)	0.94<br>(0.66-1.32)	0.72<br>(0.42-1.25)	0.74<br>(0.41-1.35)	0.87<br>(0.73-1.04)	0.97<br>(0.77-1.22)	0.93<br>(0.76-1.14)	1.05<br>(0.72-1.55)	**ZOL**

The network meta-analysis results are in the purple cells (direct estimates in the white cells). Participants with sedation during the trial are reported for each comparison as odds ratio (OR), along with 95% confidence intervals. Coloured bottom left triangle: estimates below 1 favours the medication defined by the column. White top right triangle: estimates below

1 favours the medication defined by the row. ASA = Acetylsalicylic acid; ALM = Almotriptan; DIC = Diclofenac; ELE = Eletriptan; FRO = Frovatriptan; IBU = Ibuprofen; LAS = Lasmiditan; NAP = Naproxen sodium; NAR = Naratriptan; PAR = Paracetamol; RIZ = Rizatriptan; SUM = Sumatriptan; UBR = Ubrogepant; ZOL = Zolmitriptan.

Drug classes:

**antipyretic**	.	.	.	0.80 (0.44-1.45)	1.00 (0.31-3.26)
0.55 (0.30-1.02)	**ditan**	.	.	1.53 (1.25-1.88)	.
0.70 (0.36-1.38)	1.27 (0.84-1.90)	**gepant**	.	1.21 (0.85-1.72)	.
0.82 (0.45-1.50)	1.48 (1.12-1.96)	1.17 (0.78-1.74)	**NSAID**	1.06 (0.87-1.29)	0.96 (0.68-1.35)
0.85 (0.48-1.51)	1.53 (1.25-1.88)	1.21 (0.85-1.72)	1.04 (0.86-1.25)	**placebo**	0.93 (0.84-1.02)
0.79 (0.44-1.41)	1.42 (1.13-1.78)	1.12 (0.78-1.61)	0.96 (0.79-1.18)	0.93 (0.84-1.02)	**triptan**

The network meta-analysis results are in the purple cells (direct estimates in the white cells). Participants with sedation during the trial are reported for each comparison as odds ratio (OR), along with 95% confidence intervals. Coloured bottom left triangle: estimates below 1 favours the drug class defined by the column. White top right triangle: estimates below 1 favours the drug class defined by the row. NSAID = non-steroidal anti-inflammatory drug.

8.24 Adverse events – Vertigo

Individual drugs:

**ALM**	.	.	.	.	0.81 (0.38-1.71)	.	.	2.00 (0.46-8.81)
0.67 (0.32-1.41)	**LAS**	.	.	.	1.40 (1.06-1.84)	.	.	.
0.79 (0.32-1.92)	1.17 (0.62-2.20)	**NAP**	.	.	1.09 (0.57-2.06)	.	1.22 (0.64-2.32)	.
0.51 (0.09-3.03)	0.76 (0.15-4.01)	0.65 (0.12-3.68)	**NAR**	.	1.54 (0.27-8.77)	.	2.89 (0.13-63.39)	.
1.16 (0.38-3.59)	1.73 (0.68-4.41)	1.48 (0.51-4.28)	2.26 (0.35-14.61)	**PHE**	0.81 (0.33-1.98)	.	.	.
0.94 (0.47-1.87)	1.40 (1.06-1.84)	1.20 (0.68-2.12)	1.83 (0.36-9.40)	0.81 (0.33-1.98)	**placebo**	0.96 (0.54-1.70)	0.89 (0.68-1.16)	1.02 (0.49-2.10)
0.97 (0.42-2.23)	1.44 (0.84-2.49)	1.24 (0.60-2.54)	1.89 (0.35-10.34)	0.84 (0.30-2.30)	1.03 (0.64-1.66)	**RIZ**	0.95 (0.51-1.76)	.
0.86 (0.42-1.80)	1.28 (0.88-1.87)	1.10 (0.62-1.95)	1.68 (0.32-8.75)	0.74 (0.29-1.89)	0.92 (0.71-1.19)	0.89 (0.55-1.43)	**SUM**	.
1.11 (0.47-2.60)	1.64 (0.80-3.37)	1.41 (0.59-3.38)	2.15 (0.37-12.57)	0.95 (0.31-2.90)	1.17 (0.60-2.28)	1.14 (0.50-2.57)	1.28 (0.63-2.61)	**ZOL**

The network meta-analysis results are in the purple cells (direct estimates in the white cells). Participants with vertigo during the trial are reported for each comparison as odds ratio (OR), along with 95% confidence intervals. Coloured bottom left triangle: estimates below 1 favours the medication defined by the column. White top right triangle: estimates below 1 favours the medication defined by the row. ALM = Almotriptan; LAS = Lasmiditan; NAP = Naproxen sodium; NAR = Naratriptan; PHE = Phenazone; RIZ = Rizatriptan; SUM = Sumatriptan; ZOL = Zolmitriptan.

Drug classes:

**ditan**	.	1.39 (1.07-1.80)	.
1.31 (0.78-2.22)	**NSAID**	0.99 (0.60-1.63)	1.22 (0.66-2.25)
1.39 (1.07-1.80)	1.06 (0.67-1.67)	**placebo**	0.96 (0.78-1.19)
1.35 (0.97-1.89)	1.03 (0.64-1.65)	0.97 (0.79-1.20)	**triptan**

The network meta-analysis results are in the purple cells (direct estimates in the white cells). Participants with vertigo during the trial are reported for each comparison as odds ratio (OR), along with 95% confidence intervals. Coloured bottom left triangle: estimates below 1 favours the drug class defined by the column. White top right triangle: estimates below 1 favours the drug class defined by the row. NSAID = non-steroidal anti-inflammatory drug.

8.25 Adverse events – Vomiting

Individual drugs:

**ALM**	.	.	.	.	.	.	.	.	.	.	0.79 (0.45- 1.39)	.	.	.	.	13.10 (0.74-233.26)
1.00 (0.51-1.96)	**CEL**	.	.	.	.	.	.	.	.	.	0.88 (0.60- 1.28)	.	.	.	.	.
0.77 (0.39-1.52)	0.77 (0.45-1.33)	**DIC**	.	.	.	.	.	.	.	.	1.14 (0.77- 1.68)	.	.	.	.	.
1.00 (0.52-1.91)	1.00 (0.60-1.66)	1.29 (0.77-2.17)	**ELE**	.	.	.	.	1.16 (0.68- 1.99)	.	.	0.86 (0.60- 1.23)	.	.	.	.	.
1.08 (0.57-2.04)	1.08 (0.66-1.77)	1.40 (0.85-2.30)	1.08 (0.68-1.72)	**FRO**	.	.	.	.	.	.	0.63 (0.40- 1.01)	.	.	1.04 (0.69- 1.57)	.	.
1.06 (0.56-2.02)	1.06 (0.64-1.75)	1.37 (0.83-2.29)	1.06 (0.66-1.71)	0.98 (0.62-1.55)	**IBU**	.	.	.	.	.	0.83 (0.59- 1.15)	.	.	.	.	.
0.82 (0.46-1.44)	0.81 (0.54-1.22)	1.06 (0.70-1.60)	0.82 (0.56-1.19)	0.75 (0.53-1.07)	0.77 (0.53-1.10)	**LAS**	.	.	.	.	1.08 (0.93- 1.25)	.	.	.	.	.
0.84 (0.44-1.61)	0.84 (0.50-1.40)	1.08 (0.64-1.82)	0.84 (0.51-1.36)	0.78 (0.49-1.23)	0.79 (0.49-1.27)	1.03 (0.70-1.50)	**NAP**	.	.	.	1.01 (0.68- 1.49)	.	.	1.17 (0.78- 1.75)	.	.
1.24 (0.64-2.39)	1.24 (0.74-2.08)	1.60 (0.95-2.71)	1.24 (0.82-1.88)	1.15 (0.71-1.84)	1.17 (0.72-1.89)	1.52 (1.04-2.23)	1.48 (0.90-2.43)	**NAR**	.	.	0.65 (0.45- 0.94)	.	.	6.47 (0.35-118.32)	.	.
1.57 (0.67-3.65)	1.56 (0.74-3.30)	2.03 (0.96-4.29)	1.57 (0.76-3.25)	1.45 (0.71-2.97)	1.48 (0.72-3.04)	1.92 (0.99-3.72)	1.87 (0.90-3.89)	1.26 (0.61-2.63)	**PAR**	.	0.56 (0.30- 1.07)	.	.	.	.	.
1.08 (0.43-2.67)	1.07 (0.48-2.43)	1.39 (0.61-3.16)	1.08 (0.48-2.39)	1.00 (0.45-2.19)	1.01 (0.46-2.24)	1.32 (0.63-2.76)	1.28 (0.58-2.86)	0.87 (0.39-1.94)	0.69 (0.26-1.81)	**PHE**	0.82 (0.40- 1.68)	.	.	.	.	.
0.88 (0.51-1.53)	0.88 (0.60-1.28)	1.14 (0.77-1.68)	0.88 (0.63-1.24)	0.81 (0.59-1.12)	0.83 (0.59-1.15)	1.08 (0.93-1.25)	1.05 (0.74-1.49)	0.71 (0.50-1.01)	0.56 (0.30-1.07)	0.82 (0.40-1.68)	**placebo**	1.00 (0.68- 1.46)	0.97 (0.75- 1.26)	1.03 (0.89- 1.18)	0.63 (0.38- 1.04)	0.91 (0.69- 1.20)
0.88 (0.45-1.72)	0.88 (0.51-1.50)	1.14 (0.66-1.96)	0.88 (0.53-1.47)	0.81 (0.50-1.34)	0.83 (0.50-1.37)	1.08 (0.72-1.63)	1.05 (0.63-1.76)	0.71 (0.42-1.19)	0.56 (0.27-1.19)	0.82 (0.36-1.85)	1.00 (0.68-1.46)	**RIM**	.	.	.	.
0.98 (0.54-1.76)	0.97 (0.63-1.50)	1.26 (0.82-1.96)	0.98 (0.66-1.46)	0.90 (0.63-1.30)	0.92 (0.62-1.36)	1.20 (0.93-1.55)	1.17 (0.79-1.73)	0.79 (0.52-1.19)	0.62 (0.32-1.22)	0.91 (0.43-1.92)	1.11 (0.90-1.36)	1.11 (0.72-1.71)	**RIZ**	0.89 (0.70- 1.14)	.	.
0.93 (0.53-1.64)	0.93 (0.62-1.39)	1.20 (0.80-1.81)	0.93 (0.65-1.34)	0.86 (0.63-1.18)	0.88 (0.61-1.25)	1.14 (0.93-1.39)	1.11 (0.78-1.58)	0.75 (0.51-1.09)	0.59 (0.31-1.14)	0.86 (0.41-1.80)	1.06 (0.93-1.21)	1.06 (0.71-1.58)	0.95 (0.78-1.16)	**SUM**	.	.
0.55 (0.26-1.16)	0.55 (0.29-1.03)	0.71 (0.38-1.35)	0.55 (0.30-1.01)	0.51 (0.28-0.93)	0.52 (0.28-0.95)	0.68 (0.40-1.14)	0.66 (0.36-1.21)	0.44 (0.24-0.82)	0.35 (0.16-0.80)	0.51 (0.21-1.24)	0.63 (0.38-1.04)	0.63 (0.33-1.18)	0.56 (0.33-0.97)	0.59 (0.35-1.00)	**UBR**	.
0.82 (0.44-1.51)	0.82 (0.51-1.30)	1.06 (0.66-1.70)	0.82 (0.53-1.27)	0.76 (0.50-1.15)	0.77 (0.50-1.19)	1.00 (0.73-1.37)	0.98 (0.63-1.52)	0.66 (0.42-1.03)	0.52 (0.26-1.05)	0.76 (0.35-1.65)	0.93 (0.70-1.23)	0.93 (0.58-1.49)	0.84 (0.59-1.18)	0.88 (0.65-1.20)	1.49 (0.83-2.64)	**ZOL**

The network meta-analysis results are in the purple cells (direct estimates in the white cells). Participants with vomiting during the trial are reported for each comparison as odds ratio (OR), along with 95% confidence intervals. Coloured bottom left triangle: estimates below 1 favours the medication defined by the column. White top right triangle: estimates below 1 favours the medication defined by the row. ALM = Almotriptan; CEL = Celecoxib; DIC = Diclofenac; ELE = Eletriptan; FRO = Frovatriptan; IBU = Ibuprofen; LAS = Lasmiditan; NAP = Naproxen sodium; NAR = Naratriptan; PAR = Paracetamol; PHE = Phenazone; RIM = Rimegepant; RIZ = Rizatriptan; SUM = Sumatriptan; UBR = Ubrogepant; ZOL = Zolmitriptan.

Drug classes:

**antipyretic**	.	.	.	0.56 (0.30-1.06)	.
0.52 (0.27-1.01)	**ditan**	.	.	1.08 (0.93-1.25)	.
0.47 (0.23-0.96)	0.91 (0.65-1.27)	**gepant**	.	1.19 (0.88-1.60)	.
0.59 (0.31-1.15)	1.14 (0.91-1.42)	1.25 (0.88-1.77)	**NSAID**	0.94 (0.78-1.12)	1.17 (0.78-1.74)
0.56 (0.30-1.06)	1.08 (0.93-1.25)	1.19 (0.88-1.60)	0.95 (0.80-1.12)	**placebo**	1.08 (0.98-1.20)
0.61 (0.32-1.16)	1.17 (0.98-1.40)	1.29 (0.94-1.77)	1.03 (0.85-1.25)	1.08 (0.98-1.20)	**triptan**

The network meta-analysis results are in the purple cells (direct estimates in the white cells). Participants with vomiting during the trial are reported for each comparison as odds ratio (OR), along with 95% confidence intervals. Coloured bottom left triangle: estimates below 1 favours the drug class defined by the column. White top right triangle: estimates below 1 favours the drug class defined by the row. NSAID = non-steroidal anti-inflammatory drug.

Appendix 9. Cumulative P-score ranking

P-scores are metrics to rank interventions, which are analogous to surface under the cumulative ranking curve (SUCRA) but based on frequentist instead of Bayesian methods. P-score ranking produces a hierarchy of all treatments that mostly follows that of the point estimates but takes precision into account. P-score values closer to 1.00 indicate better intervention.[24]

9.01 Efficacy – Pain freedom at 2 hours

Individual drugs:

	P-score (common)	P-score (random)
eletriptan	0.9984	0.9900
rizatriptan	0.9286	0.9150
sumatriptan	0.8516	0.8053
zolmitriptan	0.7859	0.7719
frovatriptan	0.6358	0.7340
diclofenac potassium	0.7116	0.6600
almotriptan	0.6077	0.5537
acetylsalicylic acid	0.6049	0.5442
phenazone	0.5143	0.4734
naproxen sodium	0.4756	0.4659
lasmiditan	0.4213	0.4298
ibuprofen	0.2525	0.3636
paracetamol	0.3522	0.3201
ubrogepant	0.2555	0.2917
rimegepant	0.2706	0.2712
celecoxib	0.2030	0.2349
naratriptan	0.1301	0.1737
placebo	0.0004	0.0017

Drug classes:

	P-score (common)	P-score (random)
triptan	0.9994	0.9973
NSAID	0.6249	0.6344
ditan	0.6102	0.5883
antipyretic	0.4564	0.4093
gepant	0.3090	0.3699
placebo	0.0001	0.0007

9.02 Efficacy – Sustained pain freedom from 2 to 24 hours

Individual drugs:

	**P-score (common)**	**P-score (random)**
ibuprofen	0.9784	0.9629
eletriptan	0.9319	0.9186
rizatriptan	0.7851	0.7419
sumatriptan	0.7451	0.7242
zolmitriptan	0.6653	0.6682
diclofenac potassium	0.6850	0.6286
almotriptan	0.5638	0.5674
rimegepant	0.4744	0.4704
lasmiditan	0.3325	0.3976
naproxen sodium	0.3645	0.3959
ubrogepant	0.2585	0.3052
paracetamol	0.2607	0.2633
celecoxib	0.2043	0.2244
naratriptan	0.2401	0.2131
placebo	0.0104	0.0183

Drug classes:

	**P-score (common)**	**P-score (random)**
triptan	0.9859	0.9751
NSAID	0.5875	0.5757
gepant	0.5879	0.5461
ditan	0.4607	0.5344
antipyretic	0.3544	0.3423
placebo	0.0236	0.0263

9.03 Efficacy – Pain relief at 2 hours

Individual drugs:

	**P-score (common)**	**P-score (random)**
eletriptan	0.9974	0.9951
rizatriptan	0.9134	0.9023
zolmitriptan	0.8722	0.8676
sumatriptan	0.7948	0.7816
almotriptan	0.7289	0.6989
phenazone	0.6325	0.6085
paracetamol	0.5836	0.5653
diclofenac potassium	0.5557	0.5215
naratriptan	0.4547	0.4707
naproxen sodium	0.4782	0.4663
frovatriptan	0.4491	0.4359
ibuprofen	0.4066	0.3930
lasmiditan	0.2787	0.3338
acetylsalicylic acid	0.3069	0.3107
rimegepant	0.2299	0.2755
celecoxib	0.2420	0.2588
ubrogepant	0.0755	0.1143
placebo	0.0001	0.0003

Drug classes:

	**P-score (common)**	**P-score (random)**
triptan	0.9919	0.9809
antipyretic	0.7450	0.7018
NSAID	0.6137	0.5617
ditan	0.4253	0.4742
gepant	0.2241	0.2814
placebo	0.0000	0.0000

9.04 Efficacy – Pain relapse within to 2 to 48 hours

Individual drugs:

	P-score (common)	P-score (random)
placebo	0.9966	0.9966
rimegepant	0.6503	0.6503
lasmiditan	0.3121	0.3121
sumatriptan	0.0410	0.0410

Drug classes:

	P-score (common)	P-score (random)
placebo	0.9966	0.9966
gepant	0.6503	0.6503
ditan	0.3121	0.3121
triptan	0.0410	0.0410

9.05 Efficacy – Use of rescue medication within 2 to 24 hours

Individual drugs:

	P-score (common)	P-score (random)
eletriptan	0.9896	0.9858
paracetamol	0.7948	0.7841
sumatriptan	0.7872	0.7575
zolmitriptan	0.6373	0.6162
rimegepant	0.5688	0.5815
celecoxib	0.5427	0.5294
naratriptan	0.4355	0.4747
rizatriptan	0.4615	0.4571
lasmiditan	0.4928	0.4428
almotriptan	0.4319	0.4404
frovatriptan	0.4803	0.4401
acetylsalicylic acid	0.4154	0.4318
naproxen sodium	0.3058	0.3571
ibuprofen	0.1561	0.2004
placebo	0.0002	0.0010

Drug classes:

	P-score (common)	P-score (random)
antipyretic	0.8411	0.8301
triptan	0.8431	0.7668
gepant	0.5436	0.5955
ditan	0.4684	0.4373
NSAID	0.3037	0.3701
placebo	0.0000	0.0001

9.06 Serious adverse events

Sufficient outcome data not available for analysis.

9.07 Adverse events – Abdominal pain

Individual drugs:

	P-score (common)	P-score (random)
naratriptan	0.7393	0.7388
frovatriptan	0.6181	0.6265
phenazone	0.6363	0.6245
zolmitriptan	0.6254	0.6130
rizatriptan	0.6614	0.5993
almotriptan	0.5949	0.5838
acetylsalicylic acid	0.4900	0.4944
paracetamol	0.5347	0.4880
placebo	0.4512	0.4674
ibuprofen	0.4179	0.4364
sumatriptan	0.4093	0.4053
lasmiditan	0.3529	0.3452
diclofenac potassium	0.2483	0.3039
naproxen sodium	0.2203	0.2734

Drug classes:

	P-score (common)	P-score (random)
triptan	0.6950	0.6813
antipyretic	0.6156	0.5734
placebo	0.5490	0.5519
NSAID	0.2889	0.3607
ditan	0.3514	0.3327

9.08 Adverse events – Allergic reaction

Individual drugs:

	P-score (common)	P-score (random)
placebo	0.6213	0.6213
rizatriptan	0.6121	0.6121
lasmiditan	0.3988	0.3988
sumatriptan	0.3678	0.3678

Drug classes:

	P-score (common)	P-score (random)
placebo	0.6921	0.6921
triptan	0.4281	0.4281
ditan	0.3798	0.3798

9.09 Adverse events – Chest pain/discomfort

Individual drugs:

	P-score (common)	P-score (random)
paracetamol	0.8906	0.8693
rimegepant	0.8282	0.8170
frovatriptan	0.7842	0.7766
rizatriptan	0.7451	0.7004
almotriptan	0.7286	0.6673
placebo	0.5743	0.5606
sumatriptan	0.3943	0.4385
diclofenac potassium	0.3687	0.4004
naratriptan	0.3293	0.3982
naproxen sodium	0.3623	0.3830
lasmiditan	0.3906	0.3398
zolmitriptan	0.3656	0.3389
ubrogepant	0.1190	0.1784
eletriptan	0.1191	0.1315

Drug classes:

	P-score (common)	P-score (random)
antipyretic	0.9115	0.8877
placebo	0.6969	0.6366
triptan	0.4472	0.4625
gepant	0.2429	0.3719
NSAID	0.3357	0.3533
ditan	0.3657	0.2880

9.10 Adverse events – Constipation

Individual drugs:

	P-score (common)	P-score (random)
placebo	0.6033	0.6033
lasmiditan	0.4719	0.4719
sumatriptan	0.4248	0.4248

Drug classes:

	P-score (common)	P-score (random)
placebo	0.6033	0.6033
ditan	0.4719	0.4719
triptan	0.4248	0.4248

9.11 Adverse events – Diarrhoea

Individual drugs:

	P-score (common)	P-score (random)
rimegepant	0.7864	0.7864
rizatriptan	0.7132	0.7132
naratriptan	0.5803	0.5803
ubrogepant	0.5786	0.5786
phenazone	0.5354	0.5354
acetylsalicylic acid	0.5181	0.5181
almotriptan	0.4917	0.4917
placebo	0.4905	0.4905
eletriptan	0.4029	0.4029
sumatriptan	0.3956	0.3956
lasmiditan	0.3736	0.3736
diclofenac potassium	0.3709	0.3709
naproxen sodium	0.2628	0.2628

Drug classes:

	P-score (common)	P-score (random)
gepant	0.8388	0.8388
placebo	0.5353	0.5353
triptan	0.5248	0.5248
ditan	0.3049	0.3049
NSAID	0.2963	0.2963

9.12 Adverse events – Dizziness

Individual drugs:

	P-score (common)	P-score (random)
frovatriptan	0.7938	0.7872
naratriptan	0.8111	0.7851
paracetamol	0.7842	0.7290
ibuprofen	0.7363	0.6992
almotriptan	0.6701	0.6859
celecoxib	0.6993	0.6733
placebo	0.6328	0.6210
naproxen sodium	0.5143	0.5553
rimegepant	0.4872	0.5410
acetylsalicylic acid	0.5251	0.5266
diclofenac potassium	0.5035	0.5093
sumatriptan	0.4116	0.4095
rizatriptan	0.3916	0.3762
ubrogepant	0.2352	0.2607
zolmitriptan	0.1749	0.1860
eletriptan	0.1289	0.1545
lasmiditan	0.0001	0.0003

Drug classes:

	P-score (common)	P-score (random)
antipyretic	0.8595	0.8108
placebo	0.7511	0.7353
NSAID	0.7169	0.7282
gepant	0.3074	0.3667
triptan	0.3650	0.3589
ditan	0.0000	0.0001

9.13 Adverse events – Dry mouth

Individual drugs:

	**P-score (common)**	**P-score (random)**
almotriptan	0.8244	0.8237
placebo	0.7091	0.7033
phenazone	0.6622	0.6598
ibuprofen	0.6227	0.6204
naproxen sodium	0.6044	0.6023
rizatriptan	0.6049	0.5984
acetylsalicylic acid	0.5930	0.5918
diclofenac potassium	0.5627	0.5630
naratriptan	0.5360	0.5359
lasmiditan	0.5173	0.4906
sumatriptan	0.4620	0.4639
paracetamol	0.3487	0.3487
zolmitriptan	0.3119	0.3358
frovatriptan	0.3115	0.3242
ubrogepant	0.3145	0.3225
eletriptan	0.0147	0.0157

Drug classes:

	**P-score (common)**	**P-score (random)**
placebo	0.8463	0.8376
NSAID	0.6966	0.7008
ditan	0.5138	0.4741
triptan	0.4187	0.4404
antipyretic	0.2920	0.2946
gepant	0.2326	0.2525

9.14 Adverse events – Dyspepsia

Individual drugs:

	P-score (common)	P-score (random)
almotriptan	0.7344	0.7237
paracetamol	0.7190	0.6927
rizatriptan	0.6335	0.6160
ibuprofen	0.6150	0.6076
acetylsalicylic acid	0.5502	0.5528
placebo	0.5411	0.5495
naratriptan	0.4886	0.4965
lasmiditan	0.4125	0.3954
diclofenac potassium	0.3348	0.3635
naproxen sodium	0.2432	0.2627
sumatriptan	0.2278	0.2397

Drug classes:

	P-score (common)	P-score (random)
antipyretic	0.7681	0.7462
placebo	0.6347	0.6383
NSAID	0.4144	0.4327
ditan	0.3782	0.3556
triptan	0.3044	0.3272

9.15 Adverse events – Fatigue

Individual drugs:

	P-score (common)	P-score (random)
almotriptan	0.9403	0.9265
paracetamol	0.8381	0.8126
naratriptan	0.7448	0.7240
frovatriptan	0.6703	0.6473
placebo	0.6490	0.6414
acetylsalicylic acid	0.5526	0.5654
phenazone	0.5650	0.5646
rizatriptan	0.5288	0.4916
diclofenac potassium	0.4522	0.4701
naproxen sodium	0.4075	0.4152
zolmitriptan	0.3421	0.3793
sumatriptan	0.3792	0.3648
ubrogepant	0.2381	0.2917
lasmiditan	0.1265	0.1188
eletriptan	0.0655	0.0866

Drug classes:

	P-score (common)	P-score (random)
antipyretic	0.9194	0.8860
placebo	0.7742	0.7441
NSAID	0.5388	0.5598
triptan	0.4348	0.4090
gepant	0.2381	0.3119
ditan	0.0947	0.0892

9.16 Adverse events – Gastrointestinal bleeding

Sufficient outcome data not available for analysis.

9.17 Adverse events – Hepatic toxicity

Individual drugs:

	P-score (common)	P-score (random)
rizatriptan	0.8278	0.8227
celecoxib	0.7836	0.7812
zolmitriptan	0.6779	0.6799
sumatriptan	0.6226	0.6215
placebo	0.6044	0.6050
rimegepant	0.3865	0.3956
lasmiditan	0.3448	0.3370
ubrogepant	0.2324	0.2366
paracetamol	0.0201	0.0205

Drug classes:

	P-score (common)	P-score (random)
NSAID	0.8393	0.8386
triptan	0.8024	0.8021
placebo	0.6686	0.6692
ditan	0.3754	0.3717
gepant	0.2943	0.2982
antipyretic	0.0201	0.0202

9.18 Adverse events – Major cardiovascular adverse events

Sufficient outcome data not available for analysis.

9.19 Adverse events – Nausea

Individual drugs:

	P-score (common)	P-score (random)
paracetamol	0.9825	0.9825
ibuprofen	0.7958	0.7958
frovatriptan	0.7649	0.7649
naratriptan	0.7263	0.7263
celecoxib	0.6879	0.6879
placebo	0.6227	0.6227
phenazone	0.6016	0.6016
rizatriptan	0.5649	0.5649
almotriptan	0.5227	0.5227
naproxen sodium	0.4152	0.4152
rimegepant	0.4117	0.4117
acetylsalicylic acid	0.4087	0.4087
eletriptan	0.3677	0.3677
sumatriptan	0.2617	0.2617
zolmitriptan	0.2588	0.2588
diclofenac potassium	0.2203	0.2203
ubrogepant	0.2098	0.2098
lasmiditan	0.1768	0.1768

Drug classes:

	P-score (common)	P-score (random)
antipyretic	0.9980	0.9980
placebo	0.6923	0.6923
NSAID	0.6886	0.6886
triptan	0.3309	0.3309
gepant	0.2159	0.2159
ditan	0.0744	0.0744

9.20 Adverse events – Paraesthesia

Individual drugs:

	**P-score (common)**	**P-score (random)**
rimegepant	0.8821	0.8670
eletriptan	0.7544	0.7409
naratriptan	0.6644	0.6697
frovatriptan	0.6593	0.6469
ibuprofen	0.6509	0.6283
placebo	0.6246	0.6118
almotriptan	0.5724	0.5926
acetylsalicylic acid	0.5361	0.5280
rizatriptan	0.5067	0.4708
naproxen sodium	0.4437	0.4567
diclofenac potassium	0.3640	0.3832
sumatriptan	0.3216	0.3318
ubrogepant	0.2350	0.2660
zolmitriptan	0.2187	0.2298
lasmiditan	0.0661	0.0765

Drug classes:

	**P-score (common)**	**P-score (random)**
placebo	0.8658	0.8237
NSAID	0.6260	0.6232
gepant	0.5417	0.5981
triptan	0.4315	0.4210
ditan	0.0349	0.0340

9.21 Adverse events – Pruritus

Individual drugs:

	P-score (common)	P-score (random)
naratriptan	0.7015	0.6657
placebo	0.6081	0.6036
acetylsalicylic acid	0.5217	0.5427
eletriptan	0.4569	0.4949
diclofenac potassium	0.4125	0.4824
lasmiditan	0.4819	0.4165
sumatriptan	0.3173	0.2943

Drug classes:

	P-score (common)	P-score (random)
placebo	0.7039	0.7116
NSAID	0.4462	0.4898
ditan	0.4903	0.4305
triptan	0.3596	0.3682

9.22 Adverse events – Sedation

Individual drugs:

	**P-score (common)**	**P-score (random)**
frovatriptan	0.8795	0.8651
naratriptan	0.7603	0.7591
almotriptan	0.7407	0.7322
paracetamol	0.7625	0.7232
placebo	0.6331	0.6212
ibuprofen	0.6271	0.6032
acetylsalicylic acid	0.5273	0.5268
sumatriptan	0.4633	0.4719
diclofenac potassium	0.4620	0.4716
naproxen sodium	0.4459	0.4716
rizatriptan	0.4283	0.4064
zolmitriptan	0.3243	0.3480
ubrogepant	0.2708	0.3036
eletriptan	0.1357	0.1508
lasmiditan	0.0392	0.0452

Drug classes:

	**P-score (common)**	**P-score (random)**
antipyretic	0.8476	0.8112
placebo	0.7692	0.7475
NSAID	0.6020	0.6066
triptan	0.4662	0.4703
gepant	0.2897	0.3324
ditan	0.0254	0.0319

9.23 Adverse events – Serotonergic syndrome

Sufficient outcome data not available for analysis.

9.24 Adverse events – Vertigo

Individual drugs:

	P-score (common)	P-score (random)
phenazone	0.7215	0.7038
zolmitriptan	0.6739	0.7000
almotriptan	0.6588	0.6098
rizatriptan	0.6564	0.6055
placebo	0.5726	0.5937
sumatriptan	0.4723	0.4629
naproxen sodium	0.3132	0.3804
naratriptan	0.2432	0.2560
lasmiditan	0.1881	0.1879

Drug classes:

	P-score (common)	P-score (random)
placebo	0.7486	0.7301
triptan	0.6980	0.6341
NSAID	0.4749	0.5689
ditan	0.0785	0.0668

9.25 Adverse events – Vomiting

Individual drugs:

	P-score (common)	P-score (random)
paracetamol	0.9031	0.9021
naratriptan	0.8297	0.8282
frovatriptan	0.6963	0.6961
ibuprofen	0.6710	0.6695
phenazone	0.6202	0.6197
celecoxib	0.5862	0.5855
eletriptan	0.5855	0.5845
rizatriptan	0.5768	0.5748
almotriptan	0.5665	0.5648
sumatriptan	0.4941	0.4947
rimegepant	0.4077	0.4098
placebo	0.3827	0.3827
naproxen sodium	0.3366	0.3380
zolmitriptan	0.2881	0.2927
lasmiditan	0.2592	0.2576
diclofenac potassium	0.2486	0.2504
ubrogepant	0.0476	0.0488

Drug classes:

	P-score (common)	P-score (random)
antipyretic	0.9577	0.9577
triptan	0.7025	0.7025
NSAID	0.5902	0.5902
placebo	0.4138	0.4138
ditan	0.2154	0.2154
gepant	0.1205	0.1205

Appendix 10. Vitruvian Plots

Vitruvian plots were developed in R as radial bar plots, i.e. bar charts plotted in a polar coordinate system (where coordinates are defined as the distance from a polar point and an angle from a polar axis) instead of on a cartesian plane (where coordinates are defined as distances from two fixed perpendicular axes).[27,28] Each *Vitruvian plot* corresponds to a specific intervention and shows the network meta-analysis results for multiple outcomes using either sumatriptan, ibuprofen, or placebo as the reference intervention. The impact of the intervention on the outcomes is measured on an absolute event rate scale, which is easier to understand for patients and clinicians.[29] For dichotomous outcomes, we used the odds ratios (ORs) from the outcome-specific network meta-analyses and a fixed event rate for placebo (control event rate, CER) to estimate the experimental event rate of each intervention (EER):[30]

$$EER = \frac{OR}{(1 - CER + (CER * OR))}$$

Each plot shows the overall profile of each active intervention depicted as wedges and expressed as absolute estimates across four efficacy outcomes (pain freedom at 2 hours, sustained pain freedom from 2 to 24 hours, pain relief at 2 hours, and use of rescue medications from 2 to 24 hours) as well as specific adverse events (chest pain/discomfort, dizziness, fatigue, nausea, paraesthesia, and sedation). Data on all six specific adverse events were available using sumatriptan and placebo as the reference, while no outcome data for chest pain/discomfort or fatigue was available for ibuprofen. Absolute estimates for the intervention of interest are reported in white circles to facilitate the contextualisation of the absolute effects.

10.01 Vitruvian plots – Acetylsalicylic acid

- Sumatriptan as reference: GitHub link
- Ibuprofen as reference: GitHub link
- Placebo as reference: GitHub link

10.02 Vitruvian plots – Almotriptan

- Sumatriptan as reference: GitHub link
- Ibuprofen as reference: GitHub link
- Placebo as reference: GitHub link

10.03 Vitruvian plots – Celecoxib

- Sumatriptan as reference: GitHub link
- Ibuprofen as reference: GitHub link
- Placebo as reference: GitHub link

10.04 Vitruvian plots – Diclofenac potassium

- Sumatriptan as reference: GitHub link
- Ibuprofen as reference: GitHub link
- Placebo as reference: GitHub link

10.05 Vitruvian plots – Eletriptan

- Sumatriptan as reference: GitHub link
- Ibuprofen as reference: GitHub link
- Placebo as reference: GitHub link

10.06 Vitruvian plots – Frovatriptan

- Sumatriptan as reference: GitHub link
- Ibuprofen as reference: GitHub link
- Placebo as reference: GitHub link

10.07 Vitruvian plots – Ibuprofen

- Sumatriptan as reference: GitHub link
- Ibuprofen as reference: GitHub link
- Placebo as reference: GitHub link

10.08 Vitruvian plots – Lasmiditan

- Sumatriptan as reference: GitHub link
- Ibuprofen as reference: GitHub link
- Placebo as reference: GitHub link

10.09 Vitruvian plots – Naproxen sodium

- Sumatriptan as reference: GitHub link
- Ibuprofen as reference: GitHub link
- Placebo as reference: GitHub link

10.10 Vitruvian plots – Naratriptan

- Sumatriptan as reference: GitHub link
- Ibuprofen as reference: GitHub link
- Placebo as reference: GitHub link

10.11 Vitruvian plots – Paracetamol

- Sumatriptan as reference: GitHub link
- Ibuprofen as reference: GitHub link
- Placebo as reference: GitHub link

10.12 Vitruvian plots – Phenazone

- Sumatriptan as reference: GitHub link
- Ibuprofen as reference: GitHub link
- Placebo as reference: GitHub link

10.13 Vitruvian plots – Placebo

- Sumatriptan as reference: GitHub link
- Ibuprofen as reference: GitHub link
- Placebo as reference: GitHub link

10.14 Vitruvian plots – Rimegepant

- Sumatriptan as reference: GitHub link
- Ibuprofen as reference: GitHub link
- Placebo as reference: GitHub link

10.15 Vitruvian plots – Rizatriptan

- Sumatriptan as reference: GitHub link
- Ibuprofen as reference: GitHub link
- Placebo as reference: GitHub link

10.16 Vitruvian plots – Sumatriptan

- Sumatriptan as reference: GitHub link
- Ibuprofen as reference: GitHub link
- Placebo as reference: GitHub link

10.17 Vitruvian plots – Ubrogepant

- Sumatriptan as reference: GitHub link
- Ibuprofen as reference: GitHub link
- Placebo as reference: GitHub link

10.18 Vitruvian plots – Zolmitriptan

- Sumatriptan as reference: GitHub link
- Ibuprofen as reference: GitHub link
- Placebo as reference: GitHub link

Appendix 11. CINeMA (certainty of evidence)

CINeMA quality assessment of the comparisons in the network

We evaluated the certainty of evidence using the Confidence in Network Meta-Analysis (CINeMA) framework.[25] CINeMA is a software, freely available as an open-source web application (https://cinema.ispm.unibe.ch/), which uses the R-package *'meta'* and *'netmeta'* to perform network meta-analysis of the data.[25,31] We assessed each network estimate for the primary outcomes pain freedom at 2 hours and sustained pain freedom from 2 to 24 hours according to the following criteria:[25]

1. **Within-study bias:** We graded the risk of bias for the individual studies using the Cochrane Risk of Bias Version 2 (RoB2) assessment tool.[32] RoB2 is structured into five domains that focuses on different aspects of trial design, conduct, and reporting. Based on the risk of bias for each domain, each study can be judged to have an overall 'Low risk' or 'High' risk of bias, or 'Some concerns' can be expressed.[32]
   CINeMA combines the percentual per-study contribution for each judgement of risk of bias (according to RoB2) to evaluate within-study bias for each estimate from a network meta-analysis.[25] Using CINeMA, we summarized RoB2 assessments for each pairwise comparison using the "Average RoB" rule, which applies a weighted average score for each relative effect estimate according to the percentage contribution of studies at each bias level.[33]
   Based on these scores, estimates from each pairwise comparison was assigned a within-study bias of "No concerns", "Some concerns", or "Major concerns".[33]
2. **Reporting bias:** We used the Risk Of Bias due to Missing Evidence in Network meta-analysis (ROB-MEN) tool to assess reporting bias.[34] ROB-MEN is a freely available open-source web application (https://cinema.ispm.unibe.ch/rob-men/). We assessed possible bias due to (i) the presence of identified studies with unavailable results (within-study assessment of bias), and (ii) the potential for unpublished studies (across-study assessment of bias).[34]
   We assessed within-study assessment of bias by answering the following signaling question: "Was there any eligible study for which results for the outcome of interest were unavailable, likely because of the *p*-value, magnitude or direction of the result generated?". If 'yes' could be answered to this question, we also addressed the following question: "Was the amount of information omitted from the synthesis sufficient to have a notable effect on the magnitude of the synthesized result?". If both questions could be answered with a 'yes', within-study bias (selective outcome reporting) was suspected for a given study.[34]
   Across-study assessment of bias for each pairwise comparison combines the contribution of direct comparisons to the network meta-analysis estimates with qualitative judgements of the risk of publication bias, and quantitative methods, including assessment of contour-enhanced funnel plots (where comparisons included at least 10 studies), meta-regression (using the variance as covariate), and statistical testing for small-study effects.[34]
   Based on the within-study and across-study assessments of bias, we assigned for all network meta-analysis estimates an overall 'Low risk of bias', 'Some concerns', or 'High risk of bias'.[34]
3. **Indirectness:** We sought to ensure transitivity in our network by limiting the included studies to adults with migraine diagnosed according to any iteration of the International Classification of Headache Disorders (ICHD).[8–11] Also, we excluded studies on migraine in which participants had a primary diagnosis of a concomitant somatic or psychiatric disorder, and studies that were based in an emergency department-setting. Full details regarding inclusion and exclusion criteria are available in the pre-registered and publicly available protocol (https://osf.io/kq3ys/). To assess possible heterogeneity of treatment effects, we assessed the primary outcomes using Bayesian network meta-regression for sex assigned at birth (proportion of female respondents) and presence of aura (proportion of participants presenting with aura at baseline). We furthermore conducted the following sensitivity analyses on the primary outcomes: (i) splitting nodes that included high and low doses, (ii) included only studies with low risk of bias or (iii) diagnosis of menstrual migraine, (iv) excluding studies with concomitant medical/psychiatric comorbidity or (v) participants using preventive medication, (vi) including only studies where participants were instructed to treat moderate or severe headache intensity exclusively, and (vii) the impact of placebo response.
   We considered potentially effect-modifying variables in relation to participant-, intervention-, and study characteristics for each study included in the network according to the relevance to the research question.[25]

Assessment of transitivity is challenging or impossible for interventions that are poorly connected to the network.[25] Therefore, we downgraded the network estimates for indirectness for comparisons that involved single-connected nodes.

4. **Imprecision:** We considered a clinically meaningful threshold for OR to be 0.952 or 1.05 for efficacy and adverse event outcomes. We rated treatment estimates as having 'major concerns', if the 95% confidence intervals (CIs) extended beyond the area of equivalence on the opposite side of the no effect line as the point estimate, so that the estimated treatment effect was compatible with clinically meaningful effects in both directions.[25] We rated 'no concerns' if the CIs were entirely on one side of the no effect line, or if it was entirely within the area of equivalence.[25] We rated 'some concerns', if the CIs extended into but not beyond the area of equivalence on the opposite side of the no effect line. Finally, we rated 'major concerns' if CIs extended beyond the area of equivalence on the opposite side of the no effect line.[25]
5. **Heterogeneity:** Using CINeMA, we assessed the CIs and prediction intervals for each pairwise network estimate and checked whether the prediction intervals included values that would change the conclusions based on the CIs. When CIs and prediction intervals led to the same conclusions, we rated 'no concerns' in terms of heterogeneity.[25] When CIs and prediction intervals led to conclusions that were somewhat different but of lesser impact for decision-making, we rated 'some concerns' in terms of heterogeneity.[25] When CIs and prediction intervals led to conclusions that were different, i.e. when the prediction interval crossed two boundaries (OR 1.0 and either 0.952 or 1.05), we rated "major concerns" in terms of heterogeneity.
6. **Incoherence:** When the assumption of transitivity holds, direct and indirect evidence should be in agreement and coherent.[25] For estimates where both direct and indirect evidence were available, we used CINeMA to compare direct and indirect evidence, and downgraded comparisons that were significantly different at local node splitting test. If only direct or indirect evidence was available, CINeMA used a global design-by-treatment interaction test, and rates 'no concerns' where the p-value was >0.10, 'some concerns' if it was between 0.05 and 0.10, and 'major concerns' if it was <0.05.[25]

Finally, we assigned each comparison an overall qualitative judgment of high, moderate, low, or very low certainty of evidence based on the level of concerns for the six CINeMA domains.[25] We downgraded the overall certainty of evidence by one level for each domain with "some concerns" and two levels for each domain with "major concerns", keeping in mind the fact that the six CINeMA domains are interconnected.[25] We considered the domains jointly to avoid downgrading the overall level of confidence more than necessary for related concerns. An example to illustrate the interdependent nature of the six domains could be that indirectness relates to intransitivity, which can present as incoherence. Incoherence could be concealed by heterogeneity, which in turn could increase imprecision, thereby lowering the confidence for some comparisons. Heterogeneity might also be related to variability in within-study bias and/or the presence of reporting bias.[25]

Appendix 12. Risk of Bias charts

The Risk of Bias chart shows the contribution of studies with low, moderate, or high risk of bias to each comparison (network estimate).

12.1 Pain freedom at 2 hours

GitHub link

12.2 Sustained pain freedom from 2 to 24 hours

GitHub link

Appendix 13. Evaluation of inconsistency; main analysis

13.01 Efficacy – Pain freedom at 2 hours

Individual drugs:
- GitHub link – Local
- GitHub link – Global

Drug class:
- GitHub link – Local
- GitHub link – Global

13.02 Efficacy – Sustained pain freedom from 2 to 24 hours

Individual drugs:
- GitHub link – Local
- GitHub link – Global

Drug class:
- GitHub link – Local
- GitHub link – Global

13.03 Efficacy – Pain relief at 2 hours

Individual drugs:
- GitHub link – Local
- GitHub link – Global

Drug class:
- GitHub link – Local
- GitHub link – Global

13.04 Efficacy – Pain relapse within to 2 to 48 hours

Individual drugs:
- GitHub link – Local
- GitHub link – Global

Drug class:
- GitHub link – Local
- GitHub link – Global

13.05 Efficacy – Use of rescue medication within 2 to 24 hours

Individual drugs:
- GitHub link – Local
- GitHub link – Global

Drug class:
- GitHub link – Local
- GitHub link – Global

13.06 Serious adverse events

Sufficient outcome data not available for analysis.

13.07 Adverse events – Abdominal pain

Individual drugs:
- GitHub link – Local

- GitHub link – Global

Drug class:
- GitHub link – Local
- GitHub link – Global

13.08 Adverse events – Allergic reaction

Individual drugs:
- GitHub link – Local
- GitHub link – Global

Drug class:
- GitHub link – Local
- GitHub link – Global

13.09 Adverse events – Chest pain/discomfort

Individual drugs:
- GitHub link – Local
- GitHub link – Global

Drug class:
- GitHub link – Local
- GitHub link – Global

13.10 Adverse events – Constipation

Individual drugs:
- GitHub link – Local
- GitHub link – Global

Drug class:
- GitHub link – Local
- GitHub link – Global

13.11 Adverse events – Diarrhoea

Individual drugs:
- GitHub link – Local
- GitHub link – Global

Drug class:
- GitHub link – Local
- GitHub link – Global

13.12 Adverse events – Dizziness

Individual drugs:
- GitHub link – Local
- GitHub link – Global

Drug class:
- GitHub link – Local
- GitHub link – Global

13.13 Adverse events – Dry mouth

Individual drugs:
- GitHub link – Local

- GitHub link – Global

Drug class:
- GitHub link – Local
- GitHub link – Global

13.14 Adverse events – Dyspepsia

Individual drugs:
- GitHub link – Local
- GitHub link – Global

Drug class:
- GitHub link – Local
- GitHub link – Global

13.15 Adverse events – Fatigue

Individual drugs:
- GitHub link – Local
- GitHub link – Global

Drug class:
- GitHub link – Local
- GitHub link – Global

13.16 Adverse events – Gastrointestinal bleeding

Sufficient outcome data not available for analysis.

13.17 Adverse events – Hepatic toxicity

Individual drugs:
- GitHub link – Local
- GitHub link – Global

Drug class:
- GitHub link – Local
- GitHub link – Global

13.18 Adverse events – Major adverse cardiovascular events

Sufficient outcome data not available for analysis.

13.19 Adverse events – Nausea

Individual drugs:
- GitHub link – Local
- GitHub link – Global

Drug class:
- GitHub link – Local
- GitHub link – Global

13.20 Adverse events – Paraesthesia

Individual drugs:
- GitHub link – Local
- GitHub link – Global

Drug class:

- GitHub link – Local
- GitHub link – Global

13.21 Adverse events – Pruritus

Individual drugs:
- GitHub link – Local
- GitHub link – Global

Drug class:
- GitHub link – Local
- GitHub link – Global

13.22 Adverse events – Sedation

Individual drugs:
- GitHub link – Local
- GitHub link – Global

Drug class:
- GitHub link – Local
- GitHub link – Global

13.23 Adverse events – Serotonergic syndrome

Sufficient outcome data not available for analysis.

13.24 Adverse events – Vertigo

Individual drugs:
- GitHub link – Local
- GitHub link – Global

Drug class:
- GitHub link – Local
- GitHub link – Global

13.25 Adverse events – Vomiting

Individual drugs:
- GitHub link – Local
- GitHub link – Global

Drug class:
- GitHub link – Local
- GitHub link – Global

Appendix 14. Subgroup analyses

14.1 Bayesian network meta-regression for the proportion of female participants

14.1.1 Pain freedom at 2 hours

- GitHub link

14.1.2 Sustained pain freedom from 2 to 24 hours

- GitHub link

14.2 Bayesian network meta-regression for the proportion of participants with aura at baseline

14.2.1. Pain freedom at 2 hours

- GitHub link

14.2.2 Sustained pain freedom from 2 to 24 hours

- GitHub link

Appendix 15. Sensitivity analyses

15.1 Splitting of nodes that include low and high doses

For the sensitivity analysis on low and high doses, we divided each intervention into low dose (from minimum to, but not including, midpoint of the dose range in included studies) and high dose (from midpoint to maximum of the dose range in included studies). For acetylsalicylic acid, celecoxib, diclofenac potassium, frovatriptan, phenazone, and rimegepant, only one dose was examined in the network meta-analysis.

Drug	Doses in included studies [mg]
Acetylsalicylic acid	1,000 (only one dose)
Almotriptan	6.25 (low); 12.5 (high)
Celecoxib	120 (only one dose)
Diclofenac potassium	50 (only one dose)
Eletriptan	20–40 (low); 80 (high)
Frovatriptan	2.5 (only one dose)
Ibuprofen	200 (low); 400–600 (high)
Lasmiditan	50-100 (low); 200 (high)
Naproxen sodium	275–500 (low); 800 (high)
Naratriptan	1 (low); 2.5 (high)
Paracetamol	325 (low); 1,000 (high)
Phenazone	1,000 (only one dose)
Rimegepant	75 (only one dose)
Rizatriptan	5 (low); 10 (high)
Sumatriptan	25–50 (low); 85–100 (high)
Ubrogepant	50 (low); 100 (high)
Zolmitriptan	2.5 (low); 5 (high)

15.1.1 Pain freedom at 2 hours

- GitHub link – Individual drugs
- GitHub link – Drug classes

15.1.2 Sustained pain freedom from 2-24 hours

- GitHub link – Individual drugs
- GitHub link – Drug classes

15.2 Inclusion of doses licensed by the FDA only

For the sensitivity analysis on US Food and Drug Administration (FDA)-licensed doses, we included only therapeutic doses of oral medications licensed by the FDA for the acute abortive treatment of migraine or headache attacks in adults.

Drug	Dose range licensed by the FDA [mg]
Acetylsalicylic acid	324–975
Almotriptan	6.25–12.5
Celecoxib	120
Diclofenac potassium	50
Eletriptan	20–40
Frovatriptan	2.5
Ibuprofen	200–400
Lasmiditan	50–200
Naproxen sodium	220–440
Naratriptan	1–2.5
Paracetamol	1,000–1,300
Rimegepant	75
Rizatriptan	5–10
Sumatriptan	25–100
Ubrogepant	50–100
Zolmitriptan	1.25–5

15.2.1 Pain freedom at 2 hours

- GitHub link – Individual drugs
- GitHub link – Drug classes

15.2.2 Sustained pain freedom from 2-24 hours

- GitHub link – Individual drugs
- GitHub link – Drug classes

15.3 Inclusion of studies with low risk of bias only

For the sensitivity analysis on risk of bias, we included only studies with low risk of bias according to our Risk of Bias 2 (RoB2) assessments.

15.3.1 Pain freedom at 2 hours

- GitHub link – Individual drugs
- GitHub link – Drug classes

15.3.2 Sustained pain freedom from 2-24 hours

- GitHub link – Individual drugs
- GitHub link – Drug classes

15.4 Inclusion of studies where participants had menstrual migraine only

For the sensitivity analysis on menstrual migraine, we included only studies in which ≥50% of participants were diagnosed with menstrual migraine.

15.4.1 Pain freedom at 2 hours

- GitHub link – Individual drugs
- GitHub link – Drug classes

15.4.2 Sustained pain freedom from 2-24 hours

- GitHub link – Individual drugs
- GitHub link – Drug classes

15.5 Exclusion of studies where participants had comorbidity

For the sensitivity analysis on concomitant comorbidity, we excluded studies where participants had any concomitant medical comorbidity (somatic or psychiatric).

15.5.1 Pain freedom at 2 hours

Sufficient outcome data not available for analysis.

15.5.2 Sustained pain freedom from 2-24 hours

Sufficient outcome data not available for analysis.

15.6 Exclusion of studies allowing use of preventive migraine medications

For the sensitivity analysis on preventive medications, we excluded studies that allowed the ongoing use of concomitant preventive medications for migraine.

15.6.1 Pain freedom at 2 hours

- GitHub link – Individual drugs
- GitHub link – Drug classes

15.6.2 Sustained pain freedom from 2-24 hours

- GitHub link – Individual
- GitHub link – Drug classes

15.7 Inclusion of studies where participants treated moderate or severe headache only

For the sensitivity analysis on headache intensity, we included only studies where participants were instructed to treat at moderate or severe headache intensity at baseline.

15.7.1 Pain freedom at 2 hours

- GitHub link – Individual drugs
- GitHub link – Drug classes

15.7.2 Sustained pain freedom from 2-24 hours

- GitHub link – Individual drugs
- GitHub link – Drug classes

15.8 Placebo response; only inclusion of studies after 1997

For the sensitivity analysis on placebo response, we included only studies after 1997, as this year was identified as a structural break in the meta-regression of the log-proportion of placebo responders for pain freedom at 2 hours. No structural break was identified for sustained pain freedom from 2 to 24 hours.

15.8.1 Pain freedom at 2 hours

- GitHub link – Individual drugs
- GitHub link – Drug classes

Meta-regression model

Mixed-Effects Model (k = 74; tau^2 estimator: REML)

tau^2 (estimated amount of residual heterogeneity): 0.1293 (SE = 0.0338)
tau (square root of estimated tau^2 value): 0.3596
I^2 (residual heterogeneity / unaccounted variability): 76.70%
H^2 (unaccounted variability / sampling variability): 4.29
R^2 (amount of heterogeneity accounted for): 44.43%

Test for Residual Heterogeneity:
QE(df = 65) = 283.0096, p-val < .0001

Test of Moderators (coefficients 2:5):
F(df1 = 4, df2 = 65) = 9.0727, p-val < .0001

Model Results:

	estimate	se	tval	df	pval	ci.lb	ci.ub	
intrcpt	-43.9890	14.5343	-3.0266	65	0.0035	-73.0160	-14.9620	**
data$study_year	0.0219	0.0074	2.9518	65	0.0044	0.0071	0.0366	**
data$centers_n	0.0002	0.0015	0.1557	65	0.8767	-0.0028	0.0033	
data$modsev_percentage	-0.0106	0.0020	-5.2760	65	<.0001	-0.0146	-0.0066	***
data$female_percentage	-0.0047	0.0112	-0.4162	65	0.6786	-0.0270	0.0177	
data$mean_age	-0.0158	0.0285	-0.5552	65	0.5807	-0.0728	0.0411	
factor(data$regions_simplified)Asia	-0.3785	0.2291	-1.6522	65	0.1033	-0.8361	0.0790	
factor(data$regions_simplified)Europe	-0.1305	0.1438	-0.9077	65	0.3674	-0.4176	0.1566	
factor(data$regions_simplified)Mixed	-0.3984	0.1454	-2.7401	65	0.0079	-0.6888	-0.1080	**

Signif. codes: 0 '***' 0.001 '**' 0.01 '*' 0.05 '.' 0.1 ' ' 1

Relative risk [RR]: 1.12, 95% CI 1.04–1.2, p=0, for every 5-year increase.

15.8.2 Sustained pain freedom from 2-24 hours

Meta-regression model

Mixed-Effects Model (k = 42; tau^2 estimator: REML)

tau^2 (estimated amount of residual heterogeneity): 0.1257 (SE = 0.0460)
tau (square root of estimated tau^2 value): 0.3546
I^2 (residual heterogeneity / unaccounted variability): 72.73%
H^2 (unaccounted variability / sampling variability): 3.67
R^2 (amount of heterogeneity accounted for): 13.55%

Test for Residual Heterogeneity:
QE(df = 33) = 124.2275, p-val < .0001

Test of Moderators (coefficients 2:5):
F(df1 = 4, df2 = 33) = 2.3890, p-val = 0.0708

Model Results:

	estimate	se	tval	df	pval	ci.lb	ci.ub	
intrcpt	-28.1118	21.3282	-1.3181	33	0.1966	-71.5043	15.2807	
data$study_year	0.0138	0.0108	1.2783	33	0.2101	-0.0082	0.0359	
data$centers_n	-0.0022	0.0020	-1.0790	33	0.2884	-0.0063	0.0019	
data$modsev_percentage	-0.0066	0.0027	-2.4236	33	0.0210	-0.0121	-0.0011	*
data$female_percentage	-0.0136	0.0174	-0.7815	33	0.4401	-0.0489	0.0218	
data$mean_age	-0.0090	0.0406	-0.2225	33	0.8253	-0.0917	0.0736	
factor(data$regions_simplified)Asia	-0.0758	0.2815	-0.2694	33	0.7893	-0.6485	0.4968	
factor(data$regions_simplified)Europe	-0.0682	0.2077	-0.3283	33	0.7447	-0.4908	0.3544	
factor(data$regions_simplified)Mixed	-0.2943	0.2019	-1.4576	33	0.1544	-0.7052	0.1165	

Signif. codes: 0 ‘***’ 0.001 ‘**’ 0.01 ‘*’ 0.05 ‘.’ 0.1 ‘ ’ 1

Relative risk [RR]: 1.07, 95% CI 0.96–1.2, p=0.21, for every 5-year increase.

Table S1. Drugs and doses licensed by international regulatory agencies

Drug	BNF	Europe*	FDA	PMDA	TGA	Therapeutic range (min-max)
Non-steroidal anti-inflammatory drugs (NSAIDs)						
Acetylsalicylic acid	300-900 mg (M)	BfArM: 500-1000 mg (H)	324-975 mg (H)	500-1500 mg (H)	300-1000 mg (M)	300-1500 mg
Celecoxib	-	-	120 mg (M)	-	-	120 mg
Diclofenac potassium	50 mg (M)	BfArM: 50 mg (M)	50 mg (M)	-	50 mg (M)	50 mg
Flurbiprofen	37.5-100 mg (M)	-	-	-	-	37.5-100 mg
Ibuprofen	400-600 mg (M)	ANSM: 200-400 mg (M)<br>BfArM: 200-400 mg (H)	200-400 mg (M)	200 mg (H)	200-400 mg (M)	200-600 mg
Ibuprofen sodium	256-512 mg (M)	ANSM: 256-512 mg (M)	256-512 mg (H)	-	256-512 mg (M)	256-512 mg
Ibuprofen lysine	342-684 mg (M)	ANSM, BfArM: 342-684 mg (M)	-	-	342-684 mg (M)	342-684 mg
Ketoprofen	-	ANSM: 75-150 mg (M)	-	-	-	75-150 mg
Naproxen	-	-	-	-	500 mg (M)	500 mg
Naproxen sodium	-	BfArM: 220-440 mg (H)	220-440 mg (H)		825 mg (M)	220-825 mg
Phenazone	-	BfArM: 1000 mg (M)	-	-	-	1000 mg
Tolfenamic acid	200 mg (M)	-	-	-	-	200 mg
Antipyretics						
Paracetamol	500-1000 mg (M)	ANSM, BfArM: 500-1000 mg (M)	1000-1300 mg (H)	300-1000 mg (H)	480-1330 mg (M)	300-1330 mg
Triptans						
Sumatriptan	50-100 mg (M)	ANSM, BfArM: 50-100 mg (M)	25-100 mg (M)	50-100 mg (M)	50-100 mg (M)	25-100 mg
Eletriptan	40-80 mg (M)	ANSM, BfArM: 40-80 mg (M)	20-40 mg (M)	20-40 mg (M)	40-80 mg (M)	20-80 mg
Rizatriptan	5-10 mg (M)	ANSM, BfArM: 5-10 mg (M)	5-10 mg (M)	10 mg (M)	10 mg (M)	5-10 mg
Zolmitriptan	2.5-5 mg (M)	ANSM, BfArM: 2.5-5 mg (M)	1.25-5 mg (M)	2.5-5 mg (M)	2.5-5 mg (M)	1.25-5 mg
Naratriptan	2.5 mg (M)	ANSM, BfArM: 2.5 mg (M)	1-2.5 mg (M)	2.5 mg (M)	2.5 mg (M)	1-2.5 mg
Almotriptan	12.5 mg (M)	ANSM, BfArM: 12.5 mg (M)	6.25-12.5 mg (M)	-	-	6.25-12.5 mg
Frovatriptan	2.5 mg (M)	ANSM, BfArM: 2.5 mg (M)	2.5 mg (M)	-	-	2.5 mg
Ditans						
Lasmiditan	-	EMA: 50-200 mg (M)	50-200 mg (M)	50-200 mg (M)	-	50-200 mg
Gepants						
Rimegepant	75 mg (M)	EMA: 75 mg (M)	75 mg (M)	-	-	75 mg
Ubrogepant	-	-	50-100 mg (M)	-	-	50-100 mg
Other						
Ergotamine tartrate	-	BfArM: 2 mg (M)	2 mg (M)	-	-	2 mg

Table 1 legend: Therapeutic doses for oral forms of medications licensed for the abortive treatment of acute migraine attacks in adults, according to international regulatory agencies. Abbreviations: ANSM, Agence Nationale de Sécurité du Médicament et des Produits de Santé (http://agence-prd.ansm.sante.fr/php/ecodex/index.php – France); BfArM, Bundesinstitut für Arzneimittel und Medizinprodukte (https://portal.dimdi.de/amguifree/am/search.xhtml – Germany); BNF, British National Formulary (https://bnf.nice.org.uk/ – United Kingdom); EMA, European Medicines Agency (https://www.ema.europa.eu – Europe); FDA, Food and Drugs Administration (https://www.fda.gov – United States of America); PMDA, Pharmaceuticals and Medical Devices Agency (https://www.pmda.go.jp – Japan); TGA, Therapeutic Goods Administration (https://www.tga.gov.au, https://www.pbs.gov.au/browse/medicine-listing – Australia). *: ANSM, BfArM or EMA; M: licensed for migraine; H: licensed for headache.

Table S2. Unpublished records identified through other sources

No.	Source	Record
001	AstraZeneca	AstraZeneca. Clinical Study Report: A Multicentre, Randomised, Double-Blind Trial to Compare the Efficacy and Safety of ZOMIG 2.5 mg, NARAMIG 2.5 mg and Placebo in the Acute Treatment of Adult Patients with Migraine (311CIL/0099). 2000 p. 1–5.
002	AstraZeneca	AstraZeneca. Clinical Study Report: An International, Randomized, Placebo-controlled, Double-blind Trial to Evaluate the Efficacy and Tolerability of ZOMIGTM 2.5 mg (Orally Dispersible Tablet) in the Acute Treatment of Adult Patients with Migraine (311CIL/0107). 2000.
003	AstraZeneca	AstraZeneca. Clinical Study Report: A Multicenter, 2-phase, Double-blind, Randomized, Placebo-controlled, Parallel Trial to Evaluate the Efficacy of a Single Dose of Zolmitriptan (ZOMIG®) as Acute Treatment in Phase I and Repeated Doses as Preemptive Treatment in Phase II for Menstrual Migraine. 2005 p. 1–10.
004	AstraZeneca	AstraZeneca. Clinical Study Report: A Multicentre, Randomised, Double-Blind, Placebo-Controlled, Parallel-Group Trial to Assess the Efficacy of Oral Zolmitriptan 2.5 mg in the Acute Treatment of Migraine During the Mild Intensity Phase of an Attack in Patients Highly. 2002 p. 1–6.
005	AstraZeneca	AstraZeneca. Study synopsis: A Multicenter, Randomized, Open-label Comparison of the Effects of ZOMIG-ZMT® (zolmitriptan) and Usual Non-triptan Migraine Care on Work Loss, Productivity, and Patient Preference. 2006.
006	AstraZeneca	Zeneca Pharmaceticals. Clinical Study Report: A Randomised, Double-Blind, Parallel Group Multicentre Trial to Compare the Efficacy and Safety of Zolmitriptan 2.5 mg, Zolmitriptan 5.0 mg and Sumatriptan 50 mg in the Treatment of Migraine Headache (311CIL/0070 [ZEUS]) (6-Attack Analysis). 1999 p. 1–5.
007	Eli Lilly	Eli Lilly, Company. Synopsis: COL MIG-302. 2017 p. 2–8.
008	Eli Lilly	Eli Lilly, Company. Clinical Study Report Synopsis: H8H-JE-LAIH. 2020 p. 1–6.
009	Endo pharmaceuticals	Endo pharmaceuticals. Clinical Trial Results Summary: Study EN3266-401. 2006 p. 1–2.
010	Endo pharmaceuticals	Endo pharmaceuticals. Clinical Trial Results Summary: Study VML 251-3MRM02. 2004 p. 1–6
011	European Medicines Agency (EMA)	European Medicines Agency. Assessment Report: Lasmiditan / Raywow. 2022 p. 1–141.
012	European Medicines Agency (EMA)	European Medicines Agency. Assessment Report: Rimegepant / Vydura. 2022 p. 1–135.
013	Food and Drug Administration (FDA)	US Food and Drug Administration. Drug approval package: Almotriptan/Axert (21-001) - Medical Review. 2000 p. 1–97
014	Food and Drug Administration (FDA)	US Food and Drug Administration. Drug approval package: Almotriptan/Axert (21-001) - Statistical Review. 2000.
015	Food and Drug Administration (FDA)	US Food and Drug Administration. Drug approval package: Aspirin / Extra Strength Bayer (21-317) - Medical Review. 2001 p. 1–12.
016	Food and Drug Administration (FDA)	US Food and Drug Administration. Drug approval package: Aspirin / Extra Strength Bayer (21-317) - Statistical Review. 2000 p. 1–29.
017	Food and Drug Administration (FDA)	US Food and Drug Administration. Drug approval package: Celecoxib / Elyxyb (212157Orig1s000) - Clinical Review. 2020 p. 1–92.
018	Food and Drug Administration (FDA)	US Food and Drug Administration. Drug approval package: Celecoxib / Elyxyb (212157Orig1s000) - Statistical Review. 2020 p. 1–22.
019	Food and Drug Administration (FDA)	US Food and Drug Administration. Drug approval package: Diclofenac potassium / Cambia (22-165) - Medical Review. 2008 p. 1–84.
020	Food and Drug Administration (FDA)	US Food and Drug Administration. Drug approval package: Diclofenac potassium / Cambia (22-165) - Statistical Review. 2008 p. 1–33.
021	Food and Drug Administration (FDA)	US Food and Drug Administration. Approved Labeling: Eletriptan / Relpax (21-016). 1999 p. 1–18.
022	Food and Drug Administration (FDA)	US Food and Drug Administration. Drug approval package: Eletriptan / Relpax (21-016) - Medical Review. 1999 p. 1–121.
023	Food and Drug Administration (FDA)	US Food and Drug Administration. Administrative Documents: Eletriptan / Relpax (21-016). 2002.

024	Food and Drug Administration (FDA)	US Food and Drug Administration. Drug approval package: Frovatriptan / Miguard (21-006) - Medical Review. 1999 p. 1–110.
025	Food and Drug Administration (FDA)	US Food and Drug Administration. Drug approval package: Ibuprofen / Motrin Migraine (19-012) - Medical Review. 1999 p. 1–248.
026	Food and Drug Administration (FDA)	US Food and Drug Administration. Drug approval package: Ibuprofen / Motrin Migraine (19-012) - Statistical Review. 1999 p. 1–20.
027	Food and Drug Administration (FDA)	US Food and Drug Administration. Drug approval package: Lasmiditan / Reywow (211280Orig1s000) - Clinical Review. 2019.
028	Food and Drug Administration (FDA)	US Food and Drug Administration. Drug approval package: Lasmiditan / Reywow (211280Orig1s000) - Statistical Review. 2019 p. 1–33.
029	Food and Drug Administration (FDA)	US Food and Drug Administration. Drug approval package: Naratriptan / Amerge (20-763) - Medical Review. 1997.
030	Food and Drug Administration (FDA)	US Food and Drug Administration. Drug approval package: Naratriptan / Amerge (20-763) - Statistical Review. 1997.
031	Food and Drug Administration (FDA)	US Food and Drug Administration. Drug approval package: Rimegepant / Nurtec-ODT (212728Orig1s000) - Medical Review. 2020 p. 1–118.
032	Food and Drug Administration (FDA)	US Food and Drug Administration. Drug approval package: Rimegepant / Nurtec-ODT (212728Orig1s000) - Statistical Review. 2020 p. 1–42 and 1–21.
033	Food and Drug Administration (FDA)	US Food and Drug Administration. Drug approval package: Rimegepant / Nurtec-ODT (212728Orig1s000) - Summary Review. 2020 p. 1–28.
034	Food and Drug Administration (FDA)	US Food and Drug Administration. Drug approval package: Rizatriptan / Maxalt (20-864) - Clinical Review. 1998. p. 1–149.
035	Food and Drug Administration (FDA)	US Food and Drug Administration. Drug approval package: Rizatriptan / Maxalt (20-864) - Letter and label. 1998.
036	Food and Drug Administration (FDA)	US Food and Drug Administration. Drug approval package: Rizatriptan / Maxalt (20-864) - Medical Review. 1998. p. 1–6 and 1–20.
037	Food and Drug Administration (FDA)	US Food and Drug Administration. Drug approval package: Ubrogepant / AGN 241668; MK-1602 (211765Orig1s000) - Clinical Review. 2019 p. 1–119.
038	Food and Drug Administration (FDA)	US Food and Drug Administration. Drug approval package: Ubrogepant / AGN 241668; MK-1602 (211765Orig1s000) - Statistical Review. 2019 p. 1–5.
039	Food and Drug Administration (FDA)	US Food and Drug Administration. Drug approval package: Ubrogepant / AGN 241668; MK-1602 (211765Orig1s000) - Summary Review. 2019 p. 1–27.
040	Food and Drug Administration (FDA)	US Food and Drug Administration. Drug approval package: Zolmitriptan (20-768) - Clinical Review. 1997 p. 1–85.
041	Food and Drug Administration (FDA)	US Food and Drug Administration. Drug approval package: Zolmitriptan (20-768) - Statistical Review. 1997 p. 1–39.
042	Food and Drug Administration (FDA)	US Food and Drug Administration. Drug approval package: Zolmitriptan orally disintegrating tablets / Zomig - ZMT (21-231) - Clinical Review. 2001 p. 1–17.
043	Food and Drug Administration (FDA)	US Food and Drug Administration. Drug approval package: Zolmitriptan orally disintegrating tablets / Zomig - ZMT (21-231) - Statistical Review. 2001 p. 1–12.
044	GlaxoSmithKline	GlaxoSmithKline. Clinical Study Report: S2B216. 2005 p. 1–6.
045	GlaxoSmithKline	GlaxoSmithKline. Clinical Study Report: S2BT16. 2005 p. 1–5.
046	GlaxoSmithKline	GlaxoSmithKline. Clinical Study Report: S2CM07. 2005 p. 1–8.
047	GlaxoSmithKline	GlaxoSmithKline. Clinical Study Report: S2CM11. 2005 p. 1–5.
048	GlaxoSmithKline	GlaxoSmithKline. Clinical Study Report: S2CT34. 2006 p. 1–5.
049	GlaxoSmithKline	GlaxoSmithKline. Clinical Study Report: S2W40010. 2005 p. 1–4.
050	GlaxoSmithKline	GlaxoSmithKline. Clinical Study Report: S2W40031. 2005 p. 1–4.
051	GlaxoSmithKline	GlaxoSmithKline. Clinical Study Report: S2WA1007. 2005 p. 1–4.
052	GlaxoSmithKline	GlaxoSmithKline. Clinical Study Report: S2WA3001. 2005 p. 1–7.

053	GlaxoSmithKline	GlaxoSmithKline. Clinical Study Report: S2WA3003. 2005 p. 1–7.
054	GlaxoSmithKline	GlaxoSmithKline. Clinical Study Report: S2WA4002. 2005 p. 1–4.
055	GlaxoSmithKline	GlaxoSmithKline. Clinical Study Report: S2WA4003. 2005 p. 1–6.
056	GlaxoSmithKline	GlaxoSmithKline. Clinical Study Report: S2WA4004. 2005 p. 1–6.
057	GlaxoSmithKline	GlaxoSmithKline. Clinical Study Report: S2WB2003 (S2WT50). 2005 p. 1–4.
058	GlaxoSmithKline	GlaxoSmithKline. Clinical Study Report: S2WB2004. 2005 p. 1–8.
059	GlaxoSmithKline	GlaxoSmithKline. Clinical Study Report: S2WB3002. 2005 p. 1–72.
060	GlaxoSmithKline	GlaxoSmithKline. Clinical Study Report: S2WB3011. 2005 p. 1–4.
061	GlaxoSmithKline	GlaxoSmithKline. Clinical Study Report: S2WB4001. 2005 p. 1–5.
062	GlaxoSmithKline	GlaxoSmithKline. Clinical Study Report: SUM20033. 2005 p. 1–5.
063	GlaxoSmithKline	GlaxoSmithKline. Clinical Study Report: SUM20033 (full report; document code: RM2003/00372/00). 2004.
064	GlaxoSmithKline	GlaxoSmithKline. Clinical Study Report: SUM30018. 2005 p. 1–8.
065	GlaxoSmithKline	GlaxoSmithKline. Clinical Study Report: SUM30047. 2005 p. 1–5.
066	GlaxoSmithKline	GlaxoSmithKline. Clinical Study Report: SUM30047 (full report; document code RM2004/00176/00). 2004.
067	GlaxoSmithKline	GlaxoSmithKline. Clinical Study Report: SUM30053. 2004 p. 1–4.
068	GlaxoSmithKline	GlaxoSmithKline. Clinical Study Report: SUM30053 (full report; document code: RM2004/00177/00). 2004.
069	GlaxoSmithKline	GlaxoSmithKline. Clinical Study Report: SUM40274. 2005 p. 1–4.
070	GlaxoSmithKline	GlaxoSmithKline. Clinical Study Report: SUM40275. 2005 p. 1–3.
071	GlaxoSmithKline	GlaxoSmithKline. Clinical Study Report: SUM40282. 2005 p. 1–4.
072	GlaxoSmithKline	GlaxoSmithKline. Clinical Study Report: SUM40282 (full report; document code: GM2002/00022/00). 2002.
073	GlaxoSmithKline	GlaxoSmithKline. Clinical Study Report: SUM40285. 2005 p. 1–4.
074	GlaxoSmithKline	GlaxoSmithKline. Clinical Study Report: SUM40285 (full report; document code: RM2001/00174/00). 2002.
075	GlaxoSmithKline	GlaxoSmithKline. Clinical Study Report: SUM40291. 2005 p. 1–4.
076	GlaxoSmithKline	GlaxoSmithKline. Clinical Study Report: SUM40298 (full report; document code: RM2003/00227/00). 2004.
077	GlaxoSmithKline	GlaxoSmithKline. Clinical Study Report: SUM40298. 2005 p. 1–6.
078	GlaxoSmithKline	GlaxoSmithKline. Clinical Study Report: SUM40299 (full report; document code: RM2003/00100/00). 2004.
079	GlaxoSmithKline	GlaxoSmithKline. Clinical Study Report: SUM40299. 2005 p. 1–5.
080	GlaxoSmithKline	GlaxoSmithKline. Clinical Study Report: SUM40308. 2005 p. 1–3.
081	GlaxoSmithKline	GlaxoSmithKline. Clinical Study Report: SUM40312. 2005 p. 1–6.
082	GlaxoSmithKline	GlaxoSmithKline. Clinical Study Report: SUM40312 (full report; document code: RM2003/00456/00). 2004.
083	GlaxoSmithKline	GlaxoSmithKline. Clinical Study Report: SUM40311. 2005 p. 1–7.
084	GlaxoSmithKline	GlaxoSmithKline. Clinical Study Report: SUMA2002. 2005 p. 1–9.
085	GlaxoSmithKline	GlaxoSmithKline. Clinical Study Report: SUMA30018. 2005 p. 1–8.
086	GlaxoSmithKline	GlaxoSmithKline. Clinical Study Report: SUMA4014. 2005 p. 1–6.
087	GlaxoSmithKline	GlaxoSmithKline. Clinical Study Report: SUMA4016. 2005 p. 1–5.
088	GlaxoSmithKline	GlaxoSmithKline. Clinical Study Report: SUMA4017. 2005 p. 1–6.

089	GlaxoSmithKline	GlaxoSmithKline. Clinical Study Report: SUMB4007. 2005 p. 1–6.
090	GlaxoSmithKline	GlaxoSmithKline. Clinical Study Report: SUMT37. 2006 p. 1–3.
091	GlaxoSmithKline	GlaxoSmithKline. Clinical Study Report: SMT-06-91. 2005 p. 1–3.
092	GlaxoSmithKline	GlaxoSmithKline. Clinical Study Report: TXA112496 (document code: RM2007/00907/00). 2008 p. 1–5.
093	Novartis	Novartis. Clinical Study Report: CCAT458C2301; A double-blind, double-dummy, randomized, multi-center, cross-over study to assess the efficacy and tolerability of single doses of CAT458 sachets (50 mg diclofenac-K powder for oral solution) as an acute treatment for. 2004 p. 1–17.
094	Novartis	Novartis. Clinical Study Report: BGG492. 2012 p. 1–6.
095	Via link from Trial Registry website	Almirall Prodesfarma SA. Clinical Study Report: Treatment of Acute Migraine when Pain is Mild versus when Pain is Moderate to Severe: an Almotriptan Parallel, Placebo Controlled Clinical Trial. “Act when mild?” 2007.
096	Via link from Trial Registry website	Almirall Prodesfarma SA. Study synopsis: A randomized, prospective, cross-over, double blind, placebo-controlled multicentre study to assess the efficacy and tolerability of Almotriptan 12.5 mg in the mild pain phase of Menstrual Migraine (MM) followed by an open follow-up evaluation. 2009.
097	Via link from Trial Registry website	Boehringer-Ingelheim. Synopsis: BI Trial No.: 1246.4. 2009 p. 1–7.
098	Via link from Trial Registry website	CoLucid Pharmaceuticals Inc. Synopsis version 1.0: COL-144, COL MIG-202. 2010 p. 3–13.
099	Via link from Trial Registry website	CoLucid Pharmaceuticals Inc. Synopsis: COL MIG-301. 2017 p. 2–8.
100	Via link from Trial Registry website	O’Gorman C, Jones A, Lipton RB, Tepper SJ, Tabuteau H. Presentation: Efficacy and safety of AXS-07 (MoSEICTM meloxicam/rizatriptan) in the acute treatment of migraine: results from the momentum phase 3, randomized, double-blind, active- and placebo- controlled trial. American Academy of Neurology Science Highlights: Emerging Science Presentation [Internet]. 2020.
101	Via link from Trial Registry website	O’Gorman C, Jones A, Lipton RB, Tepper SJ, Tabuteau H. Comparative efficacy of AXS-07 (MoSEIC Meloxicam/Rizatriptan) versus rizatriptan in the acute treatment of migraine. Headache. 2021;61(SUPPL 1):115-116.
102	Via link from Trial Registry website	The Menarini Group. Synopsis of Clinical Study Report: EUCTR2006-005764-81. 2010.
103	Via link from Trial Registry website	The Menarini Group. Synopsis of Clinical Study Report: Lumi/06/Fro-Mig/001; EUCTR2006-002572-17. 2010 p. 1–87.
104	Via link from Trial Registry website	The Menarini Group. Synopsis of Clinical Study Report: Gui/06/Fro-pp/002; EUCTR2006-006574-21. 2011.
105	Via link from Trial Registry website	The Menarini Group. Synopsis of Clinical Study Report: EUCTR2006-000805-42. 2014 p. 1–14.
106	Via link from Trial Registry website	The Menarini Group. Synopsis of Clinical Study Report: EUCTR2006-000785-36. 2010.
107	Via link from Trial Registry website	The Menarini Group. Synopsis of Clinical Study Report: Lumi/09/Fro+Dex-Mig/001
108	Hand search	Adwan Z. Zolmitriptan 5 mg orally disintegrating tablets deliver a 30-min onset of action and high sustained headache response in a placebo-controlled trial. Cephalalgia. 2004;24:792–3.
109	Hand search	Aube M, Chouha F, Vaillancourt J, Sampalis J. Effectiveness and Safety of Rizatriptan Benzoate 10 mg in the Treatment of Migraine Headaches. Clinical Medicine Insights: Therapeutics.2010:2 567–76.
110	Hand search	Cabarrocas X, Group AOS. Efficacy data on almotriptan, a novel 5-HT1B/1D agonist. Cephalalgia 1997; 17:421.
111	Hand search	Ferrari MD, Goadsby PJ, Roon KI, Lipton RB. Triptans (serotonin, 5-HT1B/1D agonists) in migraine: detailed results and methods of a meta-analysis of 53 trials. Cephalalgia. 2002;22(8):633–58.
112	Hand search	Fuseau E. Acute treatment of migraine with oral naratriptan: The concentration-efficacy relationship: Clin Pharmacol Ther. 1998;63(2):187.

113	Hand search	Toledano AC. Low-Dose Naltrexone/Acetaminophen Combinations and Each Component in the Acute Treatment of Migraine: Findings of a Small, Randomized, Double-Blind, and Placebo-Controlled Clinical Trial. medRxiv. 2021;20:2021.03.22.21254145.
114	Hand search	Ryan R, Géraud G, Goldstein J, Cady R, Keywood C. Clinical efficacy of frovatriptan: Placebo-controlled studies. Headache. 2002;42(SUPPL. 2):84–92.
115	Hand search	Sanis Health Inc. Product Monograph: ^{Pr}Eletriptan: 20 mg and 40 mg eletriptan (as eletriptan hydrobromide) tablets. 2021 p. 1–34.
116	Hand search	Stark S, O'Quinn S, McNeal S, Putnam G, Watson C. Naratriptan is effective for the treatment of migraine headache in sumatriptan non responders. 41st Annual Scientific Meeting: The American Association for the Study of Headache June 1l-13, 1999, Boston, Massachusetts. *Headache*. 1999;39(5):344-87.
117	Hand search	Stark S, O'Quinn, S McNeal, S, Pait G, Watson C. Naratriptan is effective for the treatment of migraine headache in sumatriptan non responders. American Academy of Neurology 51st annual meeting. Toronto, Ontario, Canada. April 17-24, 1999. Abstracts. *Neurology*. 1999;52(6 Suppl):A1-A654.
118	Hand search	Winner P, Prensky A, Linder S, et al. Adolescent migraine: Efficacy and safety of sumatriptan tablets. J. Neurol. Sci. 1997;150 SUPPL.:S172.

Table S3. Characteristics of studies included the systematic review

Study	Year	Countries/ regions	Setting	ICHD-version	Intervention	Dose (mg)	Oral formulation	No. randomised	Age, mean	Age, SD	Female, %	Sponsor
0462-039 1996	1996	USA	Outpatient	1 (1988)	rizatriptan	10	oral disintegrating tablet	127	39.0	10.7	89.5	Merck & Co.
					rizatriptan	5	oral disintegrating tablet	126	39.4	9.9	89.0	
					placebo	0	oral disintegrating tablet	128	41.3	10.7	84.7	
103 UN	*	*	Outpatient	1 (1988)	eletriptan	40	tablet	507	40.4	*	86.2	Pfizer
					placebo	0	tablet	124	40.8	*	91.9	
311CIL0099 1999	1999	*	Outpatient	1 (1988)	naratriptan	2.5	*	174	*	*	*	AstraZeneca
					zolmitriptan	2.5	*	174	*	*	*	
					placebo	0	*	92	*	*	*	
311CUS0003 2002	2002	USA	Outpatient	*	zolmitriptan	2.5	tablet	175	*	*	100.0	AstraZeneca
					placebo	0	tablet	161	*	*	100.0	
97-030 UN	*	*	Outpatient	1 (1988)	ibuprofen	400	tablet	239	38.5	*	85.4	McNeil Consumer Healthcare
					ibuprofen	200	tablet	240	38.9	*	82.5	

					placebo	0	tablet	234	38.2	*	85.5	
Adwan 2004	2004	Syria	*	*	zolmitriptan	5	oral disintegrating tablet	*	*	*	*	*
					placebo	0	oral disintegrating tablet	*	*	*	*	
Ahrens 1999	1999	Australasia, Europe, North America	Outpatient	1 (1988)	rizatriptan	10	wafer	188	43.1	*	90.4	Merck & Co.
					rizatriptan	5	wafer	182	42.7	*	84.1	
					placebo	0	wafer	185	41.6	*	90.8	
Allais 2010	2010	Italy	Outpatient	1 (1988)	almotriptan	12.5	tablet	74	35.2	8.1	100.0	Almirall Prodesfarma
					placebo	0	tablet	73	34.7	7.9	100.0	
ANODYNE-2 2018	2018	USA	Outpatient	3 (2018)	sumatriptan	100	capsule	*	*	*	*	Allodynic Therapeutics
					placebo	0	capsule	*	*	*	*	
Ashina 2021	2021	China, Europe, India, Mexico, USA	Outpatient	3 (2018)	lasmiditan	200	tablet	536	41.7	12.0	86.0	Eli Lilly
					lasmiditan	100	tablet	539	41.9	12.0	83.1	
					placebo	0	tablet	538	40.6	12.1	83.2	
Barbanti 2012	2012	Italy	Outpatient	2 (2004)	rizatriptan	10	wafer	45	44.0	12.2	80.5	Merck & Co.
					placebo	0	wafer	44	41.4	11.7	87.2	
Bartolini 2011	2011	Italy	Outpatient	2 (2004)	almotriptan	12.5	encapsulated tablet	68	*	*	*	Laboratori Guidotti

					frovatriptan	2.5	encapsulated tablet	65	*	*	*	
Bomhof 1999	1999	Europe,<br>Latin America,<br>North America	Outpatient	1 (1988)	rizatriptan	10	tablet	201	38.9	*	87.0	Merck & Co.
					naratriptan	2.5	tablet	214	39.5	*	82.0	
					placebo	0	tablet	107	39.1	*	81.0	
Boureau 1994	1994	France	Outpatient	1 (1988)	acetylsalicylic acid	1,000	tablet	*	*	*	*	*
					placebo	0	tablet	*	*	*	*	
Brandes 2005	2005	Canada,<br>USA	Outpatient	1 (1988)	eletriptan	40	*	213	38.7	11.5	83.0	Pfizer
					eletriptan	20	*	192	39.1	10.8	79.0	
					placebo	0	*	208	39.1	11.7	85.0	
Brandes 2007a	2007	USA	Outpatient	1 (1988) and 2 (2004)	naproxen sodium	500	tablet	419	39.4	11.3	86.1	Pharmacia
					sumatriptan	85	tablet	415	40.1	10.9	85.8	
					placebo	0	tablet	421	40.0	11.1	84.4	
Brandes 2007b	2007	USA	Outpatient	1 (1998) and 2 (2004)	naproxen sodium	500	tablet	434	40.4	11.6	88.7	GlaxoSmithKline in partnership with POZEN
					sumatriptan	85	tablet	434	40.3	11.4	87.3	

					placebo	0	tablet	435	40.6	10.7	89.1	
Brauneis 1994	1994	Italy	*	1 (1988)	sumatriptan	100	*	8	*	*	*	*
					placebo	0	*	7	*	*	*	
Bussone 2000	2000	Italy	Outpatient	1 (1988)	sumatriptan	50	tablet	156	*	*	*	Glaxo Wellcome
					placebo	0	tablet	56	*	*	*	
Cady 2004	2004	USA	Outpatient	*	frovatriptan	2.5	tablet	*	*	*	*	Vernalis
					placebo	0	tablet	*	*	*	*	
Cady 2006a	2006	USA	Outpatient	2 (2004)	rizatriptan	10	tablet	388	*	*	88.1	Merck & Co.
					placebo	0	tablet	195	*	*	89.3	
Cady 2006b	2006	USA	Outpatient	2 (2004)	rizatriptan	10	tablet	376	*	*	86.4	Merck & Co.
					placebo	0	tablet	188	*	*	91.1	
Cady 2009	2009	Germany, USA	Outpatient	2 (2004)	rizatriptan	10	oral disintegrating tablet	46	*	*	80.0	Merck & Co.
					placebo	0	oral disintegrating tablet	46	*	*	93.0	
Carpay 2004	2004	Belgium, Denmark, Finland, France, Germany, Greece, Italy, Netherlands,	Outpatient	1 (1988)	sumatriptan	100	tablet	159	39.7	10.3	85.9	GlaxoSmithKline
					sumatriptan	50	tablet	160	41.5	11.9	83.2	

		Norway, Spain, Switzerland, UK			placebo	0	tablet	162	40.6	10.3	79.7	
Chung 2006	2006	South Korea	Outpatient	*	almotriptan	12.5	tablet	58	*	*	*	Yuhan Corporation
					placebo	0	tablet	56	*	*	*	
Codispoti 2001	2001	USA	Outpatient	1 (1988)	ibuprofen	400	tablet	241	38.0	10.8	86.5	McNeil Consumer Healthcare
					ibuprofen	200	tablet	240	38.9	11.3	78.7	
					placebo	0	tablet	240	39.1	11.1	87.3	
Croop 2019	2019	USA	Outpatient	3beta (2013)	rimegepant	75	oral disintegrating tablet	732	40.3	12.1	84.9	Biohaven Pharmaceuticals
					placebo	0	oral disintegrating tablet	734	40.0	11.9	84.9	
CTRI/2010/091/00 1157 2010	2010	India	*	*	sumatriptan	50	tablet	*	*	*	*	MSN Laboratories Ltd.
					almotriptan	12.5	tablet	*	*	*	*	
CTRI/2023/001/04 8905 2023	2023	India	*	3 (2018)	lasmiditan	100	tablet	*	*	*	*	Pure and Cure Healthcare Private Limited
					lasmiditan	50	tablet	*	*	*	*	
					placebo	0	tablet	*	*	*	*	
Cutler 1995	1995	USA	In-clinic	1 (1988)	sumatriptan	100	tablet	66	38.1	*	92.4	Glaxo
					sumatriptan	50	tablet	62	39.5	*	93.5	

					sumatriptan	25	tablet	66	37.5	*	90.9	
					placebo	0	tablet	65	39.8	*	89.2	
Dahlöf 1993	1993	Finland,<br>Sweden	Outpatient	1 (1988)	diclofenac potassium	50	tablet	*	*	*	*	Ciba-Geigy AB
					placebo	0	tablet	*	*	*	*	
Dahlöf 1998	1998	Australia, Austria, Belgium,<br>Denmark, Finland, France, Germany, Holland,<br>Ireland,<br>Norway,<br>Spain,<br>Sweden,<br>Switzerland,<br>UK	Outpatient	1 (1988)	zolmitriptan	5	tablet	265	39.7	10.5	81.6	Glaxo Wellcome
					placebo	0	tablet	126	39.5	10.7	76.1	
Dahlöf 2001	2001	Denmark, Estonia, France, Germany, Hungary,<br>Netherlands,<br>Poland,<br>Portugal, Sweden, UK	Outpatient	1 (1988)	almotriptan	12.5	tablet	194	41.2	10.9	83.5	Almirall Prodesfarma
					almotriptan	6.25	tablet	201	40.9	9.4	86.2	
					placebo	0	tablet	99	39.4	12.2	86.3	
Dahlöf 2009	2009	Australia,<br>Belgium,<br>Denmark, France, Germany,<br>Netherlands,<br>Spain,<br>South Africa,<br>Switzerland,<br>Sweden,	Outpatient	*	sumatriptan	50	*	167	40.0	9.9	88.2	SmithKline Beecham
					placebo	0	*	166	40.4	9.4	79.1	

		UK,<br>USA										
Dib 2002	2002	France	Outpatient	1 (1988)	ketoprofen	150	tablet	*	*	*	*	Laboratoire Aventis
					ketoprofen	75	tablet	*	*	*	*	
					zolmitriptan	2.5	tablet	*	*	*	*	
					placebo	0	tablet	*	*	*	*	
Diener 2002	2002	Australia, Europe,<br>Israel,<br>South Africa	Outpatient	1 (1988)	eletriptan	80	tablet	214	40.1	11.0	89.7	Pfizer
					eletriptan	40	tablet	210	39.8	11.0	85.7	
					placebo	0	tablet	106	41.5	11.0	86.7	
Diener 2004a	2004	Germany	Outpatient	1 (1988)	acetylsalicylic acid	1,000	effervescent (active) + encapsulated tablet (dummy)	147	41.8	11.8	88.4	Bayer Corporation
					sumatriptan	50	encapsulated tablet (active) + effervescent (dummy)	135	43.7	12.1	82.2	
					placebo	0	effervescent + encapsulated tablet	153	41.9	11.7	83.6	
Diener 2004b	2004	Germany,<br>Italy,<br>Spain	Outpatient	1 (1988)	acetylsalicylic acid	1,000	effervescent tablet (active) + capsule (dummy) + encapsulated tablet (dummy)	*	*	*	*	Bayer
					ibuprofen	400	Capsule (active) + effervescent tablet	*	*	*	*	

							(dummy) + encapsulated tablet (dummy)					
					sumatriptan	50	encapsulated tablet (active) + effervescent tablet (dummy) + capsule (dummy)	*	*	*	*	
					placebo	0	effervescent tablet + capsule + encapsulated tablet	*	*	*	*	
Diener 2005	2005	Germany, Hungary, Italy, Netherlands, Poland	Outpatient	1 (1988)	diclofenac potassium	50	oral solution (active) + tablet (dummy)	107	*	*	*	Novartis
					diclofenac potassium	50	tablet (active) + oral solution (dummy)	107	*	*	*	
					placebo	0	tablet + oral solution	103	*	*	*	
Diener 2011	2011	Belgium, France, Germany, Spain, Sweden, Netherlands, UK	Outpatient	2 (2004)	eletriptan	40	encapsulated tablet (active) + tablet (dummy)	84	37.9	10.1	88.4	Boehringer Ingelheim
					placebo	0	encapsulated tablet + tablet	84	38.2	10.3	87.1	
Dodick 2019	2019	USA	Outpatient	3beta (2013)	ubrogepant	100	tablet	557	40.7	12.4	86.0	Allergan
					ubrogepant	50	tablet	556	40.2	12.0	88.7	
					placebo	0	tablet	559	41.1	11.9	87.8	
Dowson 2002a	2002	France, Germany, Netherlands,	Outpatient	1 (1988)	sumatriptan	100	capsule	194	42.1	10.5	83.5	Almirall Prodesfarma

		Spain,<br>UK			almotriptan	12.5	capsule	184	42.8	10.7	85.9	
					placebo	0	capsule	99	40.2	10.1	88.9	
Dowson 2002b	2002	Canada,<br>South<br>Africa,<br>UK	Outpatient	1 (1988)	zolmitriptan	2.5	oral disintegrating tablet	291	41.0	9.9	88.3	AstraZeneca
					placebo	0	oral disintegrating tablet	282	42.0	10.2	86.2	
Dowson 2005	2005	UK	Outpatient	1 (1988)	sumatriptan	100	tablet	*	*	*	*	Glaxo Laboratories
					placebo	0	tablet	*	*	*	*	
Eletriptan Steering Committee in Japan 2002	2002	Japan	Outpatient	1 (1988)	eletriptan	80	tablet	77	35.0	*	79.0	Pfizer
					eletriptan	40	tablet	80	36.0	*	74.0	
					eletriptan	20	tablet	80	35.0	*	68.0	
					placebo	0	tablet	84	36.0	*	76.0	
Ensink 1991	1991	Denmark, Federal Republic of Germany, France, Finland,<br>Sweden,<br>UK	Outpatient	1 (1988)	sumatriptan	100	oral disintegrating tablet	149	42.0	10.0	86.0	Glaxo
					placebo	0	oral disintegrating tablet	84	40.0	10.0	83.0	
EUCTR2006-000785-36 2008	2008	Austria,<br>Finland, Germany,<br>Greece,<br>UK	Outpatient	*	rizatriptan	10	capsule	64	*	*	*	Menarini Group
					frovatriptan	2.5	capsule	62	*	*	*	
EUCTR2006-000805-42 2008	2008	Denmark, France, Ireland, Spain,	Outpatient	*	frovatriptan	2.5	capsule	65	*	*	*	Menarini Group

		Turkey			zolmitriptan	2.5	capsule	63	*	*	*	
Freitag 2007	2007	USA	Outpatient	*	rizatriptan	10	oral disintegrating tablet	232	40.0	*	89.2	Merck & Co.
					placebo	0	oral disintegrating tablet	114	41.0	*	90.4	
Freitag 2008	2008	USA	Outpatient	2 (2004)	paracetamol	1,000	tablet	48	42.0	11.7	88.4	Merck & Co.
					rizatriptan	10	tablet	48	44.3	10.6	83.3	
					placebo	0	tablet	49	45.2	10.9	94.9	
Färkkila 2003	2003	Denmark, Finland, Norway, Sweden, Netherlands	Outpatient	1 (1988)	eletriptan	80	tablet	171	40.9	10.2	84.8	Pfizer
					eletriptan	40	tablet	188	41.4	10.6	85.6	
					placebo	0	tablet	87	40.9	12.0	90.8	
Färkkila 2012	2012	Belgium, Finland, France, Germany, Spain	Outpatient	2 (2004)	lasmiditan	200	tablet	100	39.5	10.3	92.0	CoLucid Pharmaceuticals
					lasmiditan	100	tablet	104	42.0	10.6	83.0	
					lasmiditan	50	tablet	106	40.4	12.5	84.0	
					placebo	0	tablet	103	40.5	10.3	87.0	
Gallagher 2001	2001	USA	Outpatient	1 (1988)	sumatriptan	50	tablet	338	40.6	10.2	87.3	Zeneca Pharmaceuticals
					sumatriptan	25	tablet	336	39.6	10.2	88.9	
					zolmitriptan	5	tablet	337	40.2	10.5	89.8	

					zolmitriptan	2.5	tablet	327	39.9	10.0	84.4	
Garcia-Ramos 2003	2003	Argentina, Brazil, Chile, Costa Rica, Columbia, Ecuador, Guatemala, Mexico, Panama, UK, Venezuela	Outpatient	1 (1988)	eletriptan	40	tablet (active) + capsule (dummy)	220	36.3	11.1	79.0	Pfizer
					naratriptan	2.5	capsule (active) + tablet (dummy)	221	37.5	11.0	82.0	
					placebo	0	tablet + capsule	107	36.4	11.1	82.0	
Geraud 2000	2000	Australia, Canada, Europe	Outpatient	1 (1988)	sumatriptan	100	tablet	624	38.0	10.6	84.0	Glaxo Wellcome
					zolmitriptan	5	tablet	615	38.3	10.4	83.0	
					placebo	0	tablet	74	37.9	9.7	86.0	
Gijsmant 1997	1997	Netherlands, USA	Outpatient	1 (1988)	rizatriptan	10	tablet	173	*	*	*	Merck & Co.
					rizatriptan	5	tablet	172	*	*	*	
					placebo	0	tablet	85	*	*	*	
Goadsby 1991	1991	Australia	Outpatient	1 (1988)	sumatriptan	100	tablet	*	*	*	*	Glaxo Group Research Ltd
					placebo	0	tablet	*	*	*	*	
Goadsby 2000	2000	Australia, Europe	Outpatient	1 (1988)	sumatriptan	100	capsule (active) + tablet (dummy)	167	39.9	10.0	83.7	Pfizer
					eletriptan	80	tablet (active) + capsule (dummy)	173	40.2	11.0	80.9	

					eletriptan	40	tablet (active) + capsule (dummy)	169	41.4	11.0	84.6	
					eletriptan	20	tablet (active) + capsule (dummy)	171	40.1	11.0	81.9	
					placebo	0	tablet + capsule	169	41.3	10.0	79.6	
Goadsby 2007	2007	Belgium, Finland, France, Germany, Italy, Portugal, Spain, Sweden, UK	Outpatient	2 (2004)	almotriptan	12.5	encapsulated tablet	532	39.0	11.0	86.8	Almirall Prodesfarma
					zolmitriptan	2.5	encapsulated tablet	530	40.0	11.0	83.0	
Goadsby 2008	2008	Belgium, France, Germany, Italy, Portugal	Outpatient	2 (2004)	almotriptan	12.5	tablet	116	37.8	10.3	84.2	Almirall Prodesfarma
					almotriptan	12.5	tablet	123	36.1	10.7	82.5	
					placebo	0	tablet	123	39.8	9.9	82.8	
					placebo	0	tablet	129	39.3	11.6	86.9	
Goadsby 2019	2019	Germany, UK, USA	Outpatient	3beta (2013)	lasmiditan	200	tablet	750	41.8	12.4	82.6	Eli Lilly
					lasmiditan	100	tablet	754	43.4	12.6	84.9	
					lasmiditan	50	tablet	750	42.8	13.2	84.7	
					placebo	0	tablet	751	42.6	12.9	84.5	
Goldstein 1998	1998	USA	Outpatient	1 (1988)	sumatriptan	50	*	291	40.8	*	86.0	Merck & Co.
					sumatriptan	25	*	297	40.5	*	86.0	

					rizatriptan	10	*	305	39.9	*	91.0	
					rizatriptan	5	*	294	39.9	*	88.0	
					placebo	0	*	142	39.9	*	88.0	
Goldstein 2005	2005	USA	Outpatient	1 (1988)	sumatriptan	50	caplet	67	*	*	*	Bristol-Myers Squibb
					placebo	0	caplet	35	*	*	*	
Goldstein 2006	2006	USA	Outpatient	1 (1988)	ibuprofen	400	tablet	734	38.4	*	81.5	*
					placebo	0	tablet	243	38.3	*	81.4	
Gomez-Mancilla 2014	2014	Germany, Spain, USA	In-clinic	2 (2004)	sumatriptan	100	capsule	25	36.6	10.2	88.0	Novartis
					placebo	0	capsule	25	41.4	11.0	88.0	
Gruffyd-Jones 2001	2001	Argentina, Australia, Austria, Belgium, Canada, Czech Republic, Denmark, Finland, Germany, Hungary, Mexico, Netherlands, New Zealand, Norway, Portugal, Poland, Sweden, Spain, South Africa, Switzerland, UK	Outpatient	1 (1988)	sumatriptan	50	tablet	597	41.9	10.7	84.6	AstraZeneca
					zolmitriptan	5	tablet	593	41.7	10.6	86.0	
					zolmitriptan	2.5	tablet	597	42.1	10.7	85.4	

Göbel 2000	2000	Canada, Denmark, France, Germany, Netherlands, Norway	Outpatient	1 (1988)	sumatriptan	100	tablet	130	44.0	8.7	91.1	Glaxo Wellcome
					naratriptan	2.5	tablet	134	45.0	9.9	90.7	
Göbel 2004	2004	Germany	Outpatient	1 (1988)	phenazone	1,000	tablet	120	45.4	12.3	85.7	Krewel-Meuselbach Pharmaceuticals
					placebo	0	tablet	120	45.1	11.9	83.5	
Havanka 2000	2000	Austria, Belgium, Denmark, Finland, France, Germany, Israel, Netherlands, New Zealand, Poland, Portugal, Sweden	In-clinic	1 (1988)	sumatriptan	100	tablet	98	38.0	9.0	88.8	Glaxo Wellcome
					naratriptan	2.5	tablet	87	40.0	10.0	87.4	
					naratriptan	1	tablet	85	40.0	10.0	87.1	
					placebo	0	tablet	91	39.0	10.0	89.0	
Ho 2008a	2008	USA	Outpatient	2 (2004)	rizatriptan	10	tablet (active) + liquid-filled capsule (dummy)	45	40.2	10.8	82.4	Merck & Co.
					placebo	0	liquid-filled capsule + tablet	147	42.2	10.6	90.4	
Ho 2008b	2008	Denmark, Italy, Netherlands, Poland, Spain, UK, USA	Outpatient	2 (2004)	zolmitriptan	5	tablet (active) + liquid-filled capsule (dummy)	469	41.7	12.0	86.4	Merck & Co.
					placebo	0	liquid-filled capsule + tablet	461	42.3	12.0	84.5	
Jelinski 2006	2006	Canada	Outpatient	1 (1988)	sumatriptan	100	tablet	127	39.8	11.4	85.7	GlaxoSmithKline
					sumatriptan	50	tablet	126	39.8	9.7	87.3	

					placebo	0	tablet	111	40.7	9.8	83.5	
Kaniecki 2006	2006	Canada, USA	Outpatient	1 (1988)	sumatriptan	100	tablet	168	37.3	11.0	72.3	GlaxoSmithKline
					placebo	0	tablet	164	37.4	11.2	73.2	
Kellstein 2000	2000	USA	Outpatient	1 (1988)	ibuprofen	600	gelatine liquigels	260	36.0	*	74.2	*
					ibuprofen	400	gelatine liquigels	260	36.7	*	75.9	
					ibuprofen	200	gelatine liquigels	257	37.3	*	75.3	
					placebo	0	gelatine liquigels	195	37.4	*	76.8	
Klapper 2000	2000	France, Norway, USA	Outpatient	1 (1988)	rizatriptan	10	wafer	16	*	*	*	*
					placebo	0	wafer	14	*	*	*	
Klapper 2004	2004	France, Norway, USA	Outpatient	1 (1988)	zolmitriptan	2.5	tablet	150	41.4	11.3	82.6	AstraZeneca
					placebo	0	*	152	42.0	10.3	89.4	
Klassen 1997	1997	USA	Outpatient	1 (1988)	naratriptan	2.5	tablet	140	40.5	10.2	89.8	Glaxo Wellcome
					naratriptan	1	tablet	132	38.2	10.6	83.8	
					placebo	0	tablet	137	39.9	8.6	87.7	
Kolodny 2004	2004	USA	Outpatient	1 (1988)	sumatriptan	50	tablet	287	*	*	*	Merck & Co.
					sumatriptan	25	tablet	290	*	*	*	

					rizatriptan	10	tablet	294	*	*	*	
					rizatriptan	5	tablet	288	*	*	*	
					placebo	0	tablet	288	*	*	*	
Kramer 1998	1998	USA	Outpatient	1 (1988)	rizatriptan	10	tablet	378	40.6	10.4	84.2	Merck & Co.
					placebo	0	tablet	95	41.0	9.6	81.9	
Kuca 2018	2018	USA	Outpatient	2 (2004)	lasmiditan	200	tablet	745	41.4	12.0	84.6	Initially CoLucid Pharmaceuticals, later Eli Lilly & Co.
					lasmiditan	100	tablet	744	42.2	11.7	81.3	
					placebo	0	tablet	742	42.4	12.3	85.1	
Kudrow 2005	2005	USA	Outpatient	*	sumatriptan	50	encapsulated tablet (active) + capsule (dummy)	214	41.1	9.9	90.3	Pfizer
					placebo	0	capsule + encapsulated tablet	209	39.0	9.8	87.9	
Landy 2004	2004	Canada, Europe, New Zealand	Outpatient	1 (1988)	sumatriptan	100	tablet	167	37.9	8.4	100.0	GlaxoSmithKline
					sumatriptan	50	tablet	171	37.8	8.5	100.0	
					placebo	0	tablet	175	37.5	7.6	100.0	
Lange 2000	2000	Germany	Outpatient	1 (1988)	acetylsalicylic acid	1,000	tablet	169	42.2	11.7	*	Bayer Corporation
					placebo	0	tablet	174	41.2	11.1	*	

Lee 2001	2001	South Korea	Outpatient	1 (1988)	zolmitriptan	2.5	tablet	87	37.3	9.1	82.0	AstraZeneca
					placebo	0	tablet	80	39.6	10.1	95.0	
Lines 2001	2001	Norway, Switzerland, Sweden, UK	Outpatient	1 (1988)	sumatriptan	50	tablet	428	41.8	10.1	81.5	Merck & Co.
					rizatriptan	5	tablet	418	39.9	10.3	82.0	
					placebo	0	tablet	87	44.3	10.5	87.5	
Lipton 2000a	2000	USA	Outpatient	1 (1988)	paracetamol	1,000	tablet	176	37.3	10.4	76.0	McNeil Consumer Healthcare
					placebo	0	tablet	175	36.0	9.3	83.1	
Lipton 2000b	2000	USA	Outpatient	1 (1988)	sumatriptan	50	tablet	*	*	*	*	Glaxo Wellcome
					placebo	0	tablet	*	*	*	*	
Lipton 2005	2005	USA	Outpatient	1 (1988)	acetylsalicylic acid	1,000	caplet	243	37.3	8.7	78.6	Bayer Corporation
					placebo	0	caplet	242	37.9	9.4	79.0	
Lipton 2010	2010	USA	Outpatient	2 (2004)	diclofenac potassium	50	oral solution	404	40.5	11.4	85.4	ProEthic Pharmaceuticals
					placebo	0	oral solution	403	39.9	11.2	84.1	
Lipton 2019a	2019	USA	Outpatient	3beta (2013)	celecoxib	120	oral solution	311	40.5	11.7	88.4	Dr. Reddy's Laboratories
					placebo	0	oral solution	311	40.0	12.6	85.8	

Lipton 2019b	2019	USA	Outpatient	3beta (2013)	ubrogepant	50	tablet	562	41.0	12.4	88.4	Allergan
					placebo	0	tablet	563	41.5	12.2	87.7	
Lipton 2019c	2019	USA	Outpatient	3beta (2013)	rimegepant	75	tablet	594	40.2	11.9	88.2	Biohaven Pharmaceuticals
					placebo	0	tablet	592	40.9	12.1	89.2	
Lipton 2021	2021	USA	Outpatient	3 (2018)	celecoxib	120	oral solution	316	41.4	14.0	82.0	Dr. Reddy's Laboratories
					placebo	0	oral solution	315	40.4	13.0	86.6	
Loder 2005	2005	USA	Outpatient	1 (1988)	zolmitriptan	2.5	oral disintegrating tablet	304	40.0	10.6	84.0	AstraZeneca
					placebo	0	oral disintegrating tablet	304	42.7	10.5	86.6	
MacGregor 2002	2002	UK	Outpatient	1 (1988)	acetylsalicylic acid	900	mouth-dispersible tablet	*	*	*	*	Reckitt Benckiser
					placebo	0	mouth-dispersible tablet	*	*	*	*	
Mannix 2007a	2007	USA	Outpatient	2 (2004)	rizatriptan	10	tablet	268	*	*	100.0	Merck & Co.
					placebo	0	tablet	135	*	*	100.0	
Mannix 2007b	2007	USA	Outpatient	2 (2004)	rizatriptan	10	tablet	269	*	*	100.0	Merck & Co.
					placebo	0	tablet	130	*	*	100.0	
Marcus 2014	2014	USA	Outpatient	*	sumatriptan	100	capsule	109	40.6	10.5	83.5	Bristol-Myers Squibb (original

					rimegepant	75	capsule	91	38.5	11.9	89.0	sponsor); Biohaven Pharmeuticals (final)
					placebo	0	capsule	229	37.9	11.4	85.6	
Massiou 2005	2005	France	Outpatient	1 (1988)	naratriptan	2.5	tablet	128	34.9	8.6	100.0	GlaxoSmithKline
					placebo	0	tablet	129	35.7	8.4	100.0	
Mathew 1997	1997	USA	Outpatient	1 (1988)	naratriptan	2.5	tablet	169	*	*	*	Glaxo Wellcome
					naratriptan	1	tablet	167	*	*	*	
					placebo	0	tablet	172	*	*	*	
Mathew 2003	2003	Worldwide	Outpatient	1 (1988)	sumatriptan	100	tablet	974	41.8	10.4	86.0	Pfizer
					eletriptan	40	tablet	965	41.1	10.8	87.0	
					placebo	0	tablet	482	41.6	10.6	87.0	
Mathew 2004	2004	USA	Outpatient	1 (1988)	rizatriptan	10	tablet	74	39.0	9.5	91.9	Merck & Co.
					placebo	0	tablet	38	42.0	7.2	92.1	
Mathew 2007	2007	USA	Outpatient	*	almotriptan	12.5	tablet	189	40.7	11.7	88.3	Janssen-Ortho LLC
					placebo	0	tablet	189	40.2	11.1	85.2	
Misra 2010	2010	India	Outpatient	2 (2004)	naproxen	500	*	11	*	*	*	*
					sumatriptan	50	*	11	*	*	*	

					rizatriptan	10	*	11	*	*	*	
Mitsikostas 2010	2010	Greece	Outpatient	2 (2004)	sumatriptan	50	tablet	*	*	*	*	GlaxoSmithKline
					placebo	0	tablet	*	*	*	*	
MOMENTUM 2019	2019	USA	Outpatient	3 (2018)	rizatriptan	10	*	455	41.4	10.7	84.2	Axsome Therapeutics, Inc.
					placebo	0	*	227	40.8	11.5	84.7	
Moon 2010	2010	South Korea	Outpatient	2 (2004)	frovatriptan	2.5	*	149	36.8	10.2	92.6	*
					placebo	0	*	149	38.1	9.2	87.9	
Munjal 2017	2017	USA	Outpatient	2 (2004)	celecoxib	120	oral solution	21	*	*	*	Dr. Reddy's Laboratories
					placebo	0	oral solution	21	*	*	*	
Myllylä 1998	1998	Finland	Outpatient	1 (1988)	sumatriptan	100	tablet	46	40.0	10.0	85.0	GEA Farmaceutisk Fabrik
					placebo	0	tablet	48	39.0	9.5	94.0	
Nappi 1994	1994	Italy	Outpatient	1 (1988)	sumatriptan	100	tablet	181	39.0	9.0	76.0	Glaxo
					placebo	0	tablet	94	39.0	11.0	79.0	
NCT00471952 2008	2008	USA	Outpatient	*	rizatriptan	10	capsule	*	*	*	*	Merck Sharp and Dohme
					placebo	0	capsule	*	*	*	*	
NCT00821483 2008	2008	*	*	*	frovatriptan	2.5	*	*	*	*	*	SK Chemicals Co., Ltd.

					placebo	0	*	*	*	*	*	
NCT00920686 2010	2010	USA	Outpatient	*	sumatriptan	100	capsule	75	41.2	12.0	85.5	NeurAxon Inc.
					placebo	0	capsule	82	37.2	10.9	68.3	
NCT01248468 2011	2011	USA	Outpatient	*	sumatriptan	100	tablet	304	37.8	11.4	80.6	Novartis
					placebo	0	tablet	149	37.6	11.5	83.9	
NCT01657370 2012	2012	USA	Outpatient	2 (2004)	ubrogepant	100	tablet	31	*	*	66.7	Merck & Co.
					ubrogepant	50	tablet	34	*	*	92.9	
					placebo	0	tablet	33	*	*	89.3	
NCT01986270 1998	1998	*	Outpatient	1 (1988)	eletriptan	80	tablet	180	34.1	*	88.3	Pfizer
					eletriptan	40	tablet	184	35.3	*	81.5	
					sumatriptan	50	tablet	181	34.7	*	76.2	
					sumatriptan	25	tablet	180	35.3	*	79.4	
					placebo	0	tablet	93	35.2	*	83.9	
NCT03235479 2018	2018	USA	Outpatient	3beta (2013)	rimegepant	75	tablet	582	41.9	12.3	85.5	Biohaven Pharmaceuticals
					placebo	0	tablet	580	41.3	12.1	85.6	
NCT04218162 2020	2020	South Korea	*	3 (2018)	lasmiditan	100	tablet	*	*	*	*	IlDong Pharmaceutical

					lasmiditan	50	tablet	*	*	*	*	Co Ltd
					placebo	0	*	*	*	*	*	
NCT04384367 2022	2022	Brazil	*	*	rizatriptan	10	tablet	*	*	*	*	Europharma Laboratorios S.A.
					naproxen sodium	550	tablet	*	*	*	*	
					placebo	0	tablet	*	*	*	*	
NCT05399459 2022	2022	Japan	*	3 (2018)	rimegepant	75	tablet	*	*	*	*	Biohaven Pharmaceuticals
					placebo	0	tablet	*	*	*	*	
NCT05509400 2022	2022	Australia, Austria, Belgium, Canada, Colombia, Denmark, Finland, Germany, Italy, Mexico, Poland, Spain, Sweden, UK, USA	Outpatient	3 (2018)	rimegepant	75	tablet	*	*	*	*	Biohaven Pharmaceuticals
					placebo	0	tablet	*	*	*	*	
NCT05685225 2023	2023	USA	*	3 (2018)	paracetamol	*	*	*	*	*	*	Allodynic Therapeutics
					placebo	0	*	*	*	*	*	
Nett 2003	2003	Canada, Puerto Rico,	Outpatient	1 (1988)	sumatriptan	100	tablet	137	40.5	10.0	100.0	GlaxoSmithKline

		USA			sumatriptan	50	tablet	141	39.8	10.5	100.0	
					placebo	0	tablet	139	40.7	10.5	100.0	
NTR33 2006	2006	Netherlands	Outpatient	*	rizatriptan	10	*	*	*	*	*	Merck Sharp and Dohme
					ibuprofen	400	*	*	*	*	*	
NTR34 2005	2005	Netherlands	Outpatient	*	naratriptan	2.5	*	*	*	*	*	GlaxoSmithKline
					paracetamol	1,000	*	*	*	*	*	
Padma 1998	1998	India	Outpatient	1 (1988)	sumatriptan	100	tablet	*	*	*	*	Dabur Research Foundation
					placebo	0	tablet	*	*	*	*	
Pascual 2000a	2000	Belgium, Czech Republic, Estonia, Germany, Hungary, Netherlands, Poland, Spain, UK	Outpatient	1 (1988)	almotriptan	12.5	tablet	408	40.9	*	86.3	Almirall Prodesfarma
					almotriptan	6.25	tablet	404	40.6	*	89.4	
					placebo	0	tablet	201	40.3	*	81.8	
Pascual 2000b	2000	International	Outpatient	1 (1988)	rizatriptan	10	tablet	308	38.5	*	84.0	Merck & Co.
					zolmitriptan	2.5	tablet	304	39.4	*	84.0	
					placebo	0	tablet	154	38.2	*	81.0	
Pfaffenrath 1998	1998	Czech Republic, Germany, France,	Outpatient	1 (1988)	sumatriptan	100	tablet	298	40.0	10.5	82.9	Glaxo Wellcome

		Netherlands, Norway, Sweden, Italy			sumatriptan	50	tablet	303	40.4	10.9	87.8	
					sumatriptan	25	tablet	303	39.3	10.5	83.5	
					placebo	0	tablet	99	40.4	10.7	80.8	
Pini 1995	1995	Italy	Outpatient	1 (1988)	sumatriptan	100	*	151	*	*	*	Glaxo
					placebo	0	*	87	*	*	*	
Prior 2010	2010	USA	Outpatient	1 (1988)	paracetamol	1,000	tablet	190	38.1	11.0	80.8	McNeil Consumer Healthcare
					placebo	0	tablet	188	39.8	11.8	85.8	
Rapoport 1997	1997	USA	Outpatient	1 (1988)	zolmitriptan	5	tablet	313	41.4	9.3	89.0	Glaxo Wellcome
					zolmitriptan	2.5	tablet	317	41.5	9.2	88.0	
					placebo	0	tablet	154	41.9	9.2	88.0	
Rapoport 2002	2002	USA	Outpatient	1 (1988)	frovatriptan	2.5	tablet	253	41.6	*	87.0	Vernalis
					placebo	0	tablet	249	39.8	*	84.0	
Rederich 1995	1995	USA	Outpatient	1 (1988)	sumatriptan	100	tablet	*	*	*	*	Glaxo Research Institute
					placebo	0	tablet	*	*	*	*	
Ryan 2002a	2002	USA	Outpatient	1 (1988)	frovatriptan	2.5	tablet	251	42.3	9.9	87.4	Vernalis
					placebo	0	tablet	123	40.2	10.3	86.1	

Ryan 2002b	2002	North America	Outpatient	1 (1988)	frovatriptan	2.5	tablet	850	41.2	10.0	89.7	Vernalis
					placebo	0	tablet	424	41.4	10.3	86.3	
Ryan 2002c	2002	Australia, Europe, South Africa	Outpatient	1 (1988)	sumatriptan	100	tablet	521	40.4	*	86.0	Vernalis
					frovatriptan	2.5	tablet	531	41.1	10.4	85.4	
					placebo	0	tablet	264	40.3	10.8	85.2	
S2WA4003 1998	1998	USA	Outpatient	1 (1988)	naproxen sodium	275	capsule (active) + tablet (dummy)	81	38.8	10.4	86.4	Glaxo Wellcome
					naratriptan	2.5	tablet (active) + capsule (dummy)	87	38.0	10.2	82.8	
S2WA4004 1998	1998	USA	Outpatient	1 (1988)	naproxen sodium	275	capsule (active) + tablet (dummy)	85	36.2	9.1	85.9	Glaxo Wellcome
					naratriptan	2.5	tablet (active) + capsule (dummy)	86	37.3	10.2	84.9	
S2WB3002 1996	1996	Austria, Belgium, Canada, Denmark, Finland, Iceland, Italy, Norway, Portugal, South Africa, Spain, Sweden, Switzerland, UK	Outpatient	1 (1988)	sumatriptan	100	tablet	260	40.0	10.1	84.2	Glaxo Wellcome
					naratriptan	2.5	tablet	246	41.1	10.3	82.3	
					naratriptan	1	tablet	259	40.4	9.8	81.3	
					placebo	0	tablet	130	39.6	9.4	89.8	
S2WB4001 1998	1998	Canada, Denmark, Finland, Greece,	Outpatient	1 (1988)	naratriptan	2.5	tablet	285	42.1	9.4	86.5	GlaxoSmithKline

		Hungary,<br>Israel,<br>Italy,<br>Mexico,<br>Netherlands,<br>Slovakia,<br>UK			sumatriptan	50	tablet	290	41.5	9.5	88.4	
S2WB4003 UN	*	*	*	*	zolmitriptan	2.5	*	75	*	*	*	GlaxoSmithKline
					naratriptan	2.5	*	79	*	*	*	
					placebo	0	*	27	*	*	*	
S98-073 UN	*	USA	Outpatient	1 (1988)	acetylsalicylic acid	1,000	caplet	224	31.7	10.2	80.0	Bayer Corporation
					placebo	0	caplet	222	29.5	9.5	76.0	
S98-074 UN	*	Canada,<br>Finland, Germany,<br>Italy, Netherlands,<br>Spain,<br>USA	Outpatient	1 (1988)	acetylsalicylic acid	1,000	caplet	240	41.8	10.3	78.7	Bayer Corporation
					placebo	0	caplet	242	40.8	11.7	82.4	
Sakai 2002	2002	Japan	Outpatient	1 (1988)	zolmitriptan	5	*	69	39.6	12.1	80.8	*
					zolmitriptan	2.5	*	75	37.6	12.4	86.5	
					placebo	0	*	77	37.5	11.6	71.4	
Sakai 2021	2021	Japan	Outpatient	2 (2004)	lasmiditan	200	tablet	218	44.7	10.4	79.7	Eli Lilly
					lasmiditan	100	tablet	261	45.7	9.7	84.6	

					lasmiditan	50	tablet	109	44.9	10.2	86.2	
					placebo	0	tablet	258	45.2	9.0	83.2	
Sandrini 2002	2002	Canada,<br>Europe,<br>South Africa	Outpatient	1 (1988)	eletriptan	80	encapsulated tablet	164	39.9	10.7	86.6	Pfizer
					eletriptan	40	encapsulated tablet	175	38.0	10.1	88.0	
					sumatriptan	100	encapsulated tablet	170	38.2	10.2	87.1	
					sumatriptan	50	encapsulated tablet	181	37.4	10.2	89.5	
					placebo	0	encapsulated tablet	84	37.5	10.9	89.3	
Saper 2006	2006	Asia,<br>Europe,<br>Latin America,<br>USA	Outpatient	1 (1988)	ibuprofen	400	tablet	243	41.3	12.0	86.9	Merck & Co.
					placebo	0	tablet	238	40.4	11.5	87.1	
Sargent 1995	1995	USA	Outpatient	1 (1988)	sumatriptan	100	tablet	46	38.9	9.6	95.6	Glaxo
					sumatriptan	50	tablet	46	37.4	7.7	89.1	
					sumatriptan	25	tablet	48	41.6	8.3	87.5	
					placebo	0	tablet	47	41.3	10.5	93.6	
Savani 1999	1999	Belgium, Finland,<br>Hungary,<br>New Zealand,<br>Norway,<br>UK	Outpatient	1 (1988)	sumatriptan	50	tablet	379	38.8	10.9	85.19	Glaxo Wellcome
					placebo	0	tablet	181	39.0	10.8	87.01	
Savi 2011	2011	Italy	Outpatient	2 (2004)	rizatriptan	10	encapsulated tablet	73	*	*	*	Istituto Lusofarmaco

					frovatriptan	2.5	encapsulated tablet	75	*	*	*	d'Italia
Savi 2014	2014	Switzerland	In-clinic	2 (2004)	frovatriptan	2.5	tablet	*	*	*	*	Menarini Group
					rizatriptan	10	tablet	*	*	*	*	
Seeburger 2012	2012	Canada,<br>France,<br>Italy,<br>Spain,<br>USA	Outpatient	2 (2004)	rizatriptan	10	oral disintegrating tablet	72	46.1	11.5	90.3	Merck & Co.
					placebo	0	oral disintegrating tablet	36	40.0	11.6	94.4	
Sheftell 2003	2003	USA	Outpatient	1 (1988)	eletriptan	80	tablet	312	41.9	*	89.7	Pfizer
					eletriptan	40	tablet	296	41.7	*	85.1	
					eletriptan	20	tablet	290	41.5	*	84.8	
					placebo	0	tablet	292	41.8	*	88.0	
Sheftell 2005a	2005	Canada,<br>USA	Outpatient	1 (1988)	sumatriptan	100	oral disintegrating tablet	551	41.5	11.2	84.2	GlaxoSmithKline
					sumatriptan	50	oral disintegrating tablet	556	41.6	10.8	84.8	
					placebo	0	oral disintegrating tablet	558	41.2	10.8	87.9	
Sheftell 2005b	2005	Europe	Outpatient	1 (1988)	sumatriptan	100	oral disintegrating tablet	550	40.2	10.8	82.0	GlaxoSmithKline
					sumatriptan	50	oral disintegrating tablet	561	39.9	10.8	85.2	
					placebo	0	oral disintegrating tablet	555	39.2	10.5	86.7	

Smith 2005	2005	USA	Outpatient	1 (1988) and 2 (2004)	naproxen sodium	500	tablet (active) + encapsulated tablet (dummy)	250	42.1	10.7	89.2	Pozen, Inc.
					sumatriptan	50	encapsulated tablet (active) + tablet (dummy)	229	41.2	11.3	90.8	
					placebo	0	encapsulated tablet + tablet	242	41.2	10.2	88.4	
Solomon 1997	1997	USA	Outpatient	1 (1988)	zolmitriptan	2.5	*	219	40.7	11.3	85.0	Glaxo Wellcome
					placebo	0	*	108	40.2	11.8	85.1	
Spierings 2001	2001	USA	Outpatient	1 (1988)	sumatriptan	50	tablet	623	40.3	10.1	89.0	Pharmacia
					almotriptan	12.5	tablet	632	41.2	10.1	89.0	
Spierings 2004	2004	USA	Outpatient	1 (1988)	zolmitriptan	5	oral disintegrating tablet	358	42.2	10.4	85.4	AstraZeneca
					placebo	0	oral disintegrating tablet	363	41.9	10.3	87.7	
Stark 2002	2002	Australia, Europe, South Africa	Outpatient	1 (1988)	eletriptan	80	tablet	462	42.0	*	84.8	Pfizer
					eletriptan	40	tablet	453	41.3	*	83.4	
					placebo	0	tablet	238	41.8	*	80.7	
Steiner 2003	2003	Austria, Belgium, Croatia, Czech Republic, Denmark, France, Germany, Greece,	Outpatient	1 (1988)	eletriptan	80	tablet (active) + capsule (dummy)	473	40.4	10.5	83.0	Pfizer
					eletriptan	40	tablet (active) + capsule (dummy)	473	40.3	10.4	88.0	

		Hungary,<br>Italy, Netherlands,<br>Norway,<br>Poland,<br>Portugal, Slovakia,<br>Slovenia,<br>Spain,<br>Switzerland,<br>UK			zolmitriptan	2.5	capsule (active) + tablet (dummy)	478	40.1	10.5	83.0	
					placebo	0	tablet + capsule	163	39.9	10.6	86.0	
Stronks 2003	2003	Netherlands	Outpatient	1 (1988)	naratriptan	2.5	capsule (active) + tablet (dummy)	*	*	*	*	GlaxoSmithKline
					naproxen	500	tablet (active) + capsule (dummy)	*	*	*	*	
SUM20033 2003	2003	Canada,<br>USA	In-clinic	1 (1988)	sumatriptan	100	oral disintegrating tablet	75	42.2	10.6	85.3	GlaxoSmithKline
					placebo	0	oral disintegrating tablet	77	42.5	10.9	81.8	
SUMA4016 1998	1998	USA	Outpatient	1 (1988)	sumatriptan	50	tablet	94	38.3	10.0	79.8	GlaxoSmithKline
					naproxen sodium	275	tablet	91	37.6	10.9	89.7	
SUMA4017 1998	1998	USA	Outpatient	1 (1988)	naproxen sodium	275	capsule (active) + tablet (dummy)	93	37.1	9.9	79.5	Glaxo Wellcome
					sumatriptan	50	tablet (active) + capsule (dummy)	88	38.5	10.4	92.3	
Tazaki 1993a	1993	Japan	Outpatient	1 (1988)	sumatriptan	100	tablet	76	*	*	78.3	*
					placebo	0	tablet	85	*	*	75.0	
Tazaki 1993b	1993	Japan	Outpatient	1 (1988)	sumatriptan	100	tablet	35	*	*	*	*

					sumatriptan	50	tablet	31	*	*	*	
					placebo	0	tablet	30	*	*	*	
Teall 1996	1998	USA,<br>9 other countries (not specified)	Outpatient	1 (1988)	rizatriptan	10	tablet	549	40.7	9.6	88.2	Merck & Co.
					rizatriptan	5	tablet	554	40.5	9.6	86.0	
					placebo	0	tablet	370	40.6	10.5	85.2	
Tfelt-Hansen 1995	1995	Belgium, Denmark, France, Netherlands	Outpatient	1 (1988)	sumatriptan	100	*	139	39.0	*	77.7	Synthelabo Research
					placebo	0	*	137	39.0	*	77.4	
Tfelt-Hansen 1998	1998	Australia, Canada, Europe,<br>Middle East,<br>South Africa,<br>South America	Outpatient	1 (1988)	sumatriptan	100	tablet	455	39.2	10.1	79.6	Merck & Co.
					rizatriptan	10	tablet	455	37.0	10.0	82.4	
					rizatriptan	5	tablet	180	38.3	10.3	84.1	
					placebo	0	tablet	178	38.3	10.3	82.5	
Tfelt-Hansen 2006	2006	Denmark	Outpatient	1 (1988)	sumatriptan	50	*	76	36.7	9.9	73.6	GlaxoSmithKline
					placebo	0	*	74	38.4	12.0	83.3	
The Diclofenac-K/Sumatriptan Migraine Study 1999	1999	Italy	Outpatient	1 (1988)	diclofenac potassium	50	tablet	*	*	*	*	Ciba-Geigy AB
					sumatriptan	100	tablet	*	*	*	*	

					placebo	0	tablet	*	*	*	*	
The Oral Sumatriptan Dose-defining Study 1991	1991	Austria, Belgium, Federal Republic of Germany, Finland, France, Netherlands, Sweden, UK	Outpatient	1 (1988)	sumatriptan	100	oral disintegrating tablet	313	40.0	*	84.0	Glaxo
					placebo	0	oral disintegrating tablet	212	41.0	*	83.0	
Toledano 2021	2021	USA	Outpatient	3beta (2013)	paracetamol	325	capsule	18	46.0	8.0	58.3	Allodynic Therapeutics
					placebo	0	capsule	18	40.0	10.0	82.4	
Tuchman 2006	2006	USA	Outpatient	1 (1988)	zolmitriptan	2.5	tablet	186	38.3	*	100.0	AstraZeneca
					placebo	0	tablet	180	38.7	*	100.0	
Tullo 2010	2010	Italy	Outpatient	2 (2004)	frovatriptan	2.5	encapsulated tablet	68	*	*	*	Istituto Lusofarmaco d'Italia
					zolmitriptan	2.5	encapsulated tablet	65	*	*	*	
Visser 1996a	1996	Netherlands, USA	Outpatient	1 (1988)	sumatriptan	100	tablet	72	41.0	10.0	90.3	The Wellcome Foundation
					rizatriptan	10	tablet	89	40.0	9.0	86.5	
					placebo	0	tablet	85	39.0	9.0	92.9	
Visser 1996b	1996	Netherlands	In-clinic	1 (1988)	zolmitriptan	5	tablet	21	44.0	7.0	76.0	The Wellcome Foundation
					placebo	0	tablet	20	44.0	8.0	85.0	

Voss 2016	2016	USA	Outpatient	2 (2004)	ubrogepant	100	tablet	140	41.9	11.0	88.2	Merck & Co.
					ubrogepant	50	tablet	139	40.7	12.3	86.8	
					placebo	0	tablet	139	40.5	11.7	87.6	
Wentz 2008	2008	Canada, Finland, Germany, Italy, Netherlands, Spain, USA	Outpatient	1 (1988)	naproxen sodium	825	tablet	109	41.1	10.1	82.8	GlaxoSmithKline
					placebo	0	tablet	117	40.7	10.5	77.6	
Winner 2003a	2003	USA	Outpatient	1 (1988)	sumatriptan	100	tablet	138	40.5	10.0	88.7	GlaxoSmithKline
					sumatriptan	50	tablet	138	39.8	10.5	87.7	
					placebo	0	tablet	141	40.7	10.5	87.2	
Winner 2003b	2003	USA	Outpatient	1 (1988)	sumatriptan	100	tablet	127	41.7	11.0	89.7	GlaxoSmithKline
					sumatriptan	50	tablet	122	43.5	10.4	84.7	
					placebo	0	tablet	133	42.7	9.8	90.8	
Yu 2023	2023	China, South Korea	Outpatient	3beta (2013)	rimegepant	75	oral disintegrating tablet	716	37.0	*	78.8	Biohaven Pharmaceuticals
					placebo	0	oral disintegrating tablet	715	36.0	*	83.5	

Table S4. Inclusion and exclusion criteria of studies included in the systematic review

Study	Inclusion criteria	Exclusion criteria
**0462-039 1996**	Male or non-pregnant female 18 to 65 years old with at least a 6-month history of migraine according to the International Headache Society (IHS) criteria with 1 to 8 migraines per month	Basilar or hemiplegic migraine, significant medical illnesses (including heart, kidney, liver, neurological, endocrine, gastrointestinal, hypertension), pregnancy or nursing, concomitant medication: not allowed: within 2 weeks monoamine oxidase (MAO) inhibitors, methysergide, or lithium. within 48 hours: any ergot derivative, sumatriptan or midrin, within 24 hours: any opiate, within 6 hours: analgesics, antiemetics.
**103 UN**	Adult (age ≥18), outpatient, generally in good health and capable of taking medication on an outpatient basis. At least one migraine attack every six weeks. Diagnosis of migraine according to International Headache (IHS). Migraine with or without aura. Clinic-based recruitment.	Coronary artery disease, significant arrhythmias, heart failure, uncontrolled hypertension, clinically significant active renal, hepatic, gastrointestinal, neurological including epilepsy, endocrine, metabolic, or psychiatric disease were excluded from phase 2/3 studies.
**311CIL0099 1999**	Patients were to: have had an established diagnosis of migraine as defined by the International Headache Society Criteria with an age at onset of less than 50; have experienced at least 1 migraine headache per month before the start of the trial; be between 18 and 65 years old; be able to differentiate between migraine and non-migraine headaches.	History of basilar, ophthalmoplegic or hemiplegic migraine headache; had non-migraine headaches on more than 6 days per month over the preceding 6 months; had history or symptoms suggestive of ischaemic heart disease or other vascular disease; systolic blood pressure 150 mmHg or diastolic blood pressure 95 mmHg; had current or anticipated use of methysergide or methylergotamine in the 2 weeks before randomisation; had recent history of abuse of alcohol or other drugs; were pregnant or breast-feeding; had a previous unacceptable adverse experience following use of ZOMIG, NARAMIG or other 5-hydroxytryptamine (5-HT) agonist drug, or known hypersensitivity; had hepatic or renal failure or impairment.
**311CUS0003 2002**	Women aged ≥18 years who had a history of regular menstrual periods and predictable menstrual migraine headaches.	Women aged ≥18 years who had a history of regular menstrual periods and predictable menstrual migraine headaches.
**97-030 UN**	18 years of age or older, experiencing at least moderate pain associated with migraine headache	Not reported.

**Adwan 2004**	Adult patients with a history of International Headache Society (IHS)-diagnosed migraine with or without aura were eligible for study participation	Not reported.
**Ahrens 1999**	Men and women, ≥18 years of age, were eligible for entry into the study. Patients must have had at least a 6-month history of migraine with or without aura as defined by International Headache Society (IHS) criteria, a frequency of one to eight migraine attacks per month, and generally been in good health.	History, clinical or electrocardiographic evidence of significant cardiovascular disease; uncontrolled hypertension; pregnant women or nursing mothers; history of alcohol or other drug abuse, participation in a prior rizatriptan trial, or use of an investigational compound within a month prior to the study
**Allais 2010**	1. Women 18 to 50 years of age who meet the International Headache Society (IHS)-diagnostic criteria for menstrual migraine. 2. Subject with regular menstrual periods and an history of predictable migraine attack occurring from 2 days before to 3 days after onset of menses in at least two of the three preceding months. 3. Subjects must be almotriptan-naive patients. 4. Subjects may take a single medication effective for migraine prophylaxis, for any reason. If taking a medication effective for migraine prophylaxis, subjects must have been taking a maintenance dose for at least 1 month prior to the Screening Visit (Visit “S”), and must remain on a stable dose for the duration of the study. 5. Subjects must be in generally good health as confirmed by medical and medication history, and baseline physical examination including vital signs. 6. Childbearing potential women must use an acceptable means of contraception or hormonal contraceptives for at least 30 days prior to study entry and throughout the study; or be practicing abstinence and agree to continue abstinence or to use an acceptable method of contraception should sexual activity commence	1. Subjects who routinely experience any other type of headache that would confound discrimination from a menstrual migraine headache or subjects who typically have headache which start without a mild phase. 2. Subjects who have had 15 or more headache days per month in the previous 6 months (chronic daily headache), or subjects having an average migraine headache frequency of more than 6 per month for the past 3 months 3. Subjects taking more than 1 medication for any reason which is effective for the prophylaxis of migraine headache. 4. Subjects taking any of the prohibited concomitant medications listed in the protocol or starting non-pharmacologic approaches for migraine treatment within 14 days of Visit “S”. 5. Subjects who typically experience vomiting with their headaches or with hemiplegic or basilar migraines. 6. Subjects known to have any significant and unstable medical disease that would compromise the subject’s welfare or confound the study results, or any disease or condition that compromises the function of those body systems that could result in altered absorption, excess accumulation or impaired metabolism or excretion of the test medication.
**ANODYNE-2 2018**	Male or female 18 to 65 years of age. History of migraine with or without aura according to the International Classification of Headache Disorders (ICHD)-3rd edition (beta version) for at least one-year with first migraine prior to age 50. Migraine-associated nausea with ≥half the migraine attacks. 2 to 8 migraines per month in each of the previous 3 months. The patient is able to complete study questionnaires, comply with the study requirements and restrictions, and willing to provide written informed consent and authorize Health Insurance Portability and Accountability Act (HIPAA). The female patient who is premenopausal or postmenopausal less than 1 year, or have not had surgical sterilization (i.e., tubal ligation, partial or complete hysterectomy) must have a negative urine pregnancy test, be non-lactating, and	The patient in the opinion of the investigator may have medication-overuse headaches (as defined by ICHD - 3 beta criteria for medication-overuse headache) during the preceding 3 months. The patient in the opinion of the investigator has chronic migraine (as defined by ICHD - 3 beta criteria for chronic migraine) during the preceding 3 months. History of cluster headaches or neurologically complicated migraine (hemiplegic, basilar, retinal, ophthalmoplegic). Initiation or change in medications with possible migraine prophylactic effects during 3 months before inclusion into the trial (E.g., calcium channel blockers, tricyclic antidepressants, beta-blockers, or Botox). Use of opiates or barbiturates more than 3 days per month. Any concurrent medical or psychiatric condition, this includes, but is not limited to chronic unstable debilitating diseases, significant renal or hepatic impairment. The patient has a history within the previous 3 years of abuse of any drug, prescription, illicit, or alcohol. The Female patient is pregnant or breast-feeding. The Male patient is not practicing 2 different methods of birth control with their partner during the study, and for 28 days after the

	commit to using 2 methods of adequate and reliable contraception throughout the study and for 28 days after taking the last dose of the study drug (e.g., barrier with additional spermicidal, intra-uterine device, hormonal contraception). Male patients must be surgically sterile or commit to the use of 2 different methods of birth control during the study and for 28 days after the study.ice, hormonal contraception).	investigational drug last dose or will not remain abstinent during the study, and for 28 days after the last dose. The patient has known-hypersensitivity reaction to any of the components of the investigational drug. Consumption of analgesic medication or muscle relaxants (including all benzodiazepines) for other conditions on a regular basis. The patient has used emergency care treatment more than 3 times in the previous 6 months. The patient has participated in another study with an investigational drug within 30 days prior to randomization and/or plan to participate during the study. The patient has a history of congenital heart disease, cardiac arrhythmias, or cardiovascular disease, e.g., ischemic heart disease (e.g., stable angina pectoris, unstable angina, vasospastic angina, myocardial infarction or silent myocardial ischemia), cerebrovascular syndromes (e.g., strokes of any type or transient ischemic attacks), peripheral vascular disease, ischemic bowel disease, or Raynaud syndrome. Uncontrolled hypertension (sitting >160 mmHg systolic pressure or >95mmHg diastolic pressure). The patient, in the investigator's opinion, is likely to have unrecognized cardiovascular or cerebrovascular disease. History of epilepsy. Allergy to sulfonamides. Consumption of monoamine oxidase inhibitor (MAOI) drug, tricyclic antidepressant, selective serotonin reuptake inhibitor (SSRI) or Serotonin Noradrenaline Reuptake Inhibitor (SNRI).
**Ashina 2021**	Migraine with or without aura fulfilling the International Headache Society (IHS) diagnostic criteria 1.1 and 1.2.1. History of disabling migraine for at least 1 year. Migraine onset before the age of 50 years, History of 3 to 8 migraine attacks per month (<15 headache days per month) during the past 3 months. Migraine Disability Assessment (MIDAS) score ≥11. Able and willing to complete an eDiary to record the details of each migraine attack treated with study drug. Women of child-bearing potential must be using or willing to use a highly effective form of contraception. Agree not to post any personal medical data or information related to the study on any website or social media site until the entire trial has completed.	Known hypersensitivity to lasmiditan, or to any excipient of lasmiditan oral tablets. History or evidence of hemorrhagic stroke, epilepsy, or any other condition placing the participant at increased risk of seizures. History of recurrent dizziness and/or vertigo including benign paroxysmal positional vertigo, Ménière's disease, vestibular migraine, and other vestibular disorders. History of diabetes mellitus with complications (diabetic retinopathy, nephropathy, or neuropathy). History of orthostatic hypotension with syncope. Significant renal or hepatic impairment in the opinion of the investigator or if they meet hepatic monitoring criteria. Participants who, in the investigator's judgment, are actively suicidal and therefore deemed to be at significant risk for suicide. History, within past 12 months, of chronic migraine or other forms of primary or secondary chronic headache disorder (eg, hemicranias continua, medication overuse headache where headache frequency is ≥15 headache days per month). Use of more than 3 doses per month of either opioids or barbiturates. Initiation of or a change in concomitant medication to reduce the frequency of migraine episodes within 3 months prior to screening. Pregnant or breast-feeding women. History of drug or alcohol abuse/dependence within 1 year prior to screening. Any medical condition or clinical laboratory test which in the judgment of the investigator makes the participant unsuitable for the study. Currently enrolled in any other clinical study involving an investigational product. Relatives of, or staff directly reporting to, the Investigator. Participants who are employees of the sponsor.
**Barbanti 2012**	Male or female ≥18 years of age at screening. History of migraine with or without aura > 1 year with ≥1 and ≤8 moderate or Severe migraine attacks per month in the 2 months prior to screening that typically last longer than 2 hours. During the migraine attack (if untreated) patient has every time at least 1 of the following symptoms due to the activation of the trigeminal-autonomic reflex (UAs): unilateral conjunctival injection and/or lacrimation and/or nasal congestion/rhinorrhea and/or ptosis and/or eyelid oedema and/or forehead/facial sweating. A patient who is of reproductive	Patient is pregnant or breast-feeding, or expecting to conceive within the projected duration of the study. Patient has difficulty distinguishing his/her migraine attacks from tension or interval headaches. History of predominantly mild migraine attacks or migraines usually resolved spontaneously in less than 2 hours. Basilar or hemiplegic migraine headache. Patient has more than 15 headache-days per month or has taken medication for acute headache on more than 10 days per month in any of the 3 months prior to screening. Patient is taking migraine Propranolol or has discontinued it from less than 14 days. Patient is taking migraine prophylactic medication where the prescribed daily dose has changed during the 3 months prior to screening. Patient was >50 years old at age of migraine onset. Recent history (within

	potential agrees to remain abstinent or use (or have their partner use) 2 acceptable methods of birth control within the projected duration of the study (intrauterine device (IUD), diaphragm with spermicide, contraceptive sponge, condoms, vasectomy. Health condition in the opinion of the investigator based on screening assessment including medical history, physical examination, and laboratory testing carried out within ~2 months prior to study treatment. Patient agrees to participate by giving written informed consent and able to complete the study questionnaire(s) and paper diary.	the past 5 years) or current evidence of drug or alcohol abuse or is a "recreational user" of illicit drugs. Concomitant use of propranolol, ergot derivatives, methysergide or monoamine oxidase (MAO)-inhibitors. Hypersensitivity to any marketed 5-hydroxytryptamine (5-HT)$_{1B/1D}$ receptor agonist. History or clinical evidence of ischemic heart disease (e.g., angina pectoris of any type, history of myocardial infarction or documented silent ischemia) or symptoms or findings consistent with ischemic heart disease, coronary artery vasospasm (including Prinzmetal's variant angina, or other significant underlying cardiovascular disease. Patient has clinical, laboratory, or electrocardiogram (ECG) evidence of uncontrolled hypertension, uncontrolled diabetes, or significant pulmonary, renal, hepatic, endocrine, or other systemic disease in the opinion of the investigator. Patient has, in the opinion of the investigator, other confounding pain syndromes, psychiatric conditions such as uncontrolled major depression based on criteria such as DSM-IV, dementia or significant neurological disorders other than migraine. History of neoplastic disease ≤5 years prior to signing informed consent. Patient has a history of gastric or small intestinal surgery (including gastric bypass surgery or banding), or has a disease that causes malabsorption. History or current evidence of any clinically significant disease that according to the investigator might confound the results of the study, complicate the interpretation of the study results, interfere with the patient's participation for the full duration of the study, or pose an additional undue risk to the patient.
**Bartolini 2011**	The study included subjects of male or female gender, 65 years old, with a current history of migraine with or without aura, according to International Headache Society (IHS) 2004 criteria, and with at least one, but no more than 6 migraine attacks per month for 6 months prior to entering the study.	Patient could not be enrolled in the study in case of: (a) uncontrolled hypertension; (b) ischemic heart disease; (c) cardiac arrhythmias or symptomatic Wolff-Parkinson-White syndrome; (d) previous stroke or transient ischemic attack; (e) severe liver or renal impairment; (f) any other severe or disabling medical condition; (g) history of alcohol or analgesic or psychotropic drug abuse; (h) known hypersensitivity to study drugs; (i) previously demonstrated inadequate response to at least two triptans; (j) current use of propranolol or ergotamine (and its derivatives) as a prophylactic agent; (k) current use or use in the previous 2 weeks of monoamine oxidase (MAO)-inhibitors; (l) use of either test medication to treat any one of the last three episodes of migraine;(m) other headaches that have been lasting for more than 6 days. Pregnant women and breast-feeding mothers were excluded as well, while women with childbearing potential but not practicing an effective method of birth control were to be submitted to a pregnancy test, if clinically indicated.
**Bomhof 1999**	Women and men (18 to 65 years), who met International Headache Society (IHS) criteria for migraine with or without aura, were enrolled. Patients had to have a 6-month history of migraine and usually experienced 1–8 attacks/month.	Patients were excluded if they had clinical evidence of cerebrovascular or cardiovascular disease including significant electrocardiogram (ECG) abnormality, or if they had a history within 1 year or current evidence of drug or alcohol abuse. Patients with any contraindication or sensitivity to 5-hydroxytryptamine (5-HT)$_{1B/1D}$ agonists, or those who had received treatment with any other investigational compound or device within the past 30 days, were also excluded, as were pregnant women or nursing mothers.
**Boureau 1994**	Men and women, 18 to 65 years old, suffering from migraine without aura who met the diagnostic criteria of the International Headache Society (IHS) beginning before the age of 50 years. The disorder had been present for more than a year, and they had two to six acute attacks per month.	Subjects with other types of headaches, pregnant women, patients with contra-indications to aspirin, acetaminophen and codeine, or sufferers of diseases likely to modify the course of the trial. Were not included in the trial. Inability to complete the self-evaluation report form assessed by an evaluation of an ordinary acute attack during the consultation on admission was also an exclusionary criterion.

**Brandes 2005**	Eligible patients consisted of women or men, aged 18 to 65 years, who met the International Headache Society criteria for migraine with or without aura, with a duration of illness of at least 1 year, and who reported an average frequency of one to four attacks per month over the past 3 months.	Patients were excluded for the following reasons: (i) presence of frequent non-migrainous headache, chronic daily headache, or atypical or migraine variant presentations (e.g. familial hemiplegic, basilar migraine, migraine with prolonged aura, etc.); (ii) any history of cerebrovascular or coronary artery disease, heart failure, uncontrolled hypertension or abnormal electrocardiogram (ECG); (iii) any clinically significant medical illness or laboratory abnormalities; (iv) severe reduction in gastrointestinal absorption; (v) current use of prophylactic medication for the treatment of migraine; (vi) hypersensitivity or known contraindication to treatment with eletriptan; (vii) concomitant use of potent cytochrome 3A4 (CYP3A4) inhibitors; (viii) misuse or abuse of alcohol or other substances, including analgesics or ergotamine; (ix) use of any experimental drug within the past month; and (x) women who were pregnant, breast-feeding, or sexually active without use of medically acceptable contraception.
**Brandes 2007a**	Men and nonpregnant, nonlactating women were eligible for the studies if they were between 18 and 65 years of age, had at least a 6-month history of migraine with or without aura as defined by the International Headache Society criteria, had an average of 2 to 6 moderate or severe migraine episodes monthly during the 3 months preceding the screening visit, and could distinguish migraine episodes from other types of headache. Women had to be physiologically incapable of becoming pregnant or, if they could become pregnant, had to agree to practice adequate contraception during the study.	Patients were excluded if they had more than 6 migraine attacks monthly during either of the 2 months before screening; chronic daily headache (≥15 days per month of nonmigraine headaches during each of the 3 months before screening); uncontrolled hypertension (diastolic blood pressure > 95mm Hg or systolic blood pressure >160mm Hg); confirmed or suspected cardiovascular or cerebrovascular disease; a history of cardiac arrhythmias requiring medication or clinically significant electrocardiogram abnormalities that, in the investigator's opinion, contraindicated study participation; or basilar or hemiplegic migraine. Other exclusion criteria included current use or use within 3 months before screening of migraine prophylactic medication containing ergotamine, an ergot derivative, or methysergide; use of a monoamine oxidase inhibitor within 2 weeks or preparations containing St John's wort within 4 weeks before screening; and regular use of any anticoagulant or NSAID (except aspirin, ≤325 mg/d, for cardiovascular prophylaxis)
**Brandes 2007b**	Men and nonpregnant, nonlactating women were eligible for the studies if they were between 18 and 65 years of age, had at least a 6-month history of migraine with or without aura as defined by the International Headache Society criteria, had an average of 2 to 6 moderate or severe migraine episodes monthly during the 3 months preceding the screening visit, and could distinguish migraine episodes from other types of headache. Women had to be physiologically incapable of becoming pregnant or, if they could become pregnant, had to agree to practice adequate contraception during the study.	Patients were excluded if they had more than 6 migraine attacks monthly during either of the 2 months before screening; chronic daily headache (≥15 days per month of nonmigraine headaches during each of the 3 months before screening); uncontrolled hypertension (diastolic blood pressure >95mm Hg or systolic blood pressure >160mm Hg); confirmed or suspected cardiovascular or cerebrovascular disease; a history of cardiac arrhythmias requiring medication or clinically significant electrocardiogram abnormalities that, in the investigator's opinion, contraindicated study participation; or basilar or hemiplegic migraine. Other exclusion criteria included current use or use within 3 months before screening of migraine prophylactic medication containing ergotamine, an ergot derivative, or methysergide; use of a monoamine oxidase inhibitor within 2 weeks or preparations containing St John's wort within 4 weeks before screening; and regular use of any anticoagulant or NSAID (except aspirin, ≤325 mg/d, for cardiovascular prophylaxis)
**Brauneis 1994**	International Classification of Headache Disorders (ICHD)-verified diagnosis of migraine.	Severe arterial hypertension. History of angina pectoris. Myocardial ischemia. Vasculopathy. Patients with severe liver and/or renal insufficiency. Epilepsy.

**Bussone 2000**	Patients, aged 18 to 65 years, were eligible for the study if they met International Headache Society (IHS) criteria for migraine with or without aura and if they had experienced migraine attacks of moderate to severe intensity for at least one year with a frequency of 1 to 6 per month.	Ergotamine, migraine prophylaxis, drug abuse, cardiovascular disorders, severe systemic diseases, pregnancy and lactation.
**Cady 2004**	Adults were eligible to enter the study if they had a history of migraine of at least 1 year, according to IHS criteria, with relatively frequent migraines (between 2 and 8 per month over the previous 2 months), and gave written informed consent.	Patients were excluded from the study if they had more than 15 headache days per month; if they had coronary artery disease, significant cerebrovascular disease, uncontrolled hypertension, severe hepatic or renal insufficiency, or any other condition that could interfere with study participation. Pregnant and breast-feeding women were also excluded. Existing migraine prophylaxis was permitted but was to remain unchanged from 2 months prior to study entry until the end of the study treatment period.
**Cady 2006a**	Patients 18 years of age or older, with at least a 6-month history of migraine with or without aura by International Headache Society criteria, were eligible. Patients had a history of 1 to 4 migraine attacks per month, with attacks that were typically mild at onset. Female patients of childbearing potential had negative urine pregnancy tests at screening and agreed to use adequate contraception during the study. Patients with a history of coexisting migraine and episodic or chronic tension-type headache had to be able to clearly distinguish migraine attacks from tension-type headache to be eligible.	Patients with ischemic heart disease, uncontrolled hypertension, coronary artery vasospasm (including Prinzmetal's variant angina), or other significant underlying cardiovascular disease were excluded. Patients agreed to discontinue using monoamine oxidase inhibitors and propranolol 2 weeks before receiving study medication; any 5-hydroxytryptamine (5-HT)$_{1B/D}$-agonist, ergot-type medication (eg, methysergide, dihydroergotamine), opiates, or barbiturates 24 hours before receiving study medication; and non-opiate analgesics and anti-emetics 6 hours before receiving study medication. In addition, daily analgesics taken for any reason were not permitted (except for aspirin <325 mg/day). Patients who participated in TAME1 were not eligible to participate in TAME2.
**Cady 2006b**	Patients 18 years of age or older, with at least a 6-month history of migraine with or without aura by International Headache Society criteria, were eligible. Patients had a history of 1 to 4 migraine attacks per month, with attacks that were typically mild at onset. Female patients of childbearing potential had negative urine pregnancy tests at screening and agreed to use adequate contraception during the study. Patients with a history of coexisting migraine and episodic or chronic tension-type headache had to be able to clearly distinguish migraine attacks from tension-type headache to be eligible.	Patients with ischemic heart disease, uncontrolled hypertension, coronary artery vasospasm (including Prinzmetal's variant angina), or other significant underlying cardiovascular disease were excluded. Patients agreed to discontinue using monoamine oxidase inhibitors and propranolol 2 weeks before receiving study medication; any 5-hydroxytryptamine (5-HT)$_{1B/D}$-agonist, ergot-type medication (eg, methysergide, dihydroergotamine), opiates, or barbiturates 24 hours before receiving study medication; and non-opiate analgesics and anti-emetics 6 hours before receiving study medication. In addition, daily analgesics taken for any reason were not permitted (except for aspirin <325 mg/day). Patients who participated in TAME1 were not eligible to participate in TAME2.
**Cady 2009**	Participants were at least 18 years of age with at least a 1-year history of migraine with or without aura by International Headache Society criteria. Participants had a history of 1 to 4 migraine attacks per month with attacks that were typically mild at onset and recognizable as migraine. Women of childbearing potential agreed to use adequate contraception during the study. Participants with a history of coexisting migraine and episodic tension-type headache had to be able to clearly distinguish migraine attacks from tension-type headache to be eligible	Participants with chronic tension-type headache or > with >15 headache days per month or who had taken medication for >10 days per month in any of the previous 3 months were excluded. Participants with a history of cerebrovascular accident, transient ischemic attack, ischemic heart disease, uncontrolled hypertension, coronary artery vasospasm (including Prinzmetal's variant angina), or other significant underlying cardiovascular or peripheral vascular disease were excluded. Participants agreed to discontinue use of the following: monoamine oxidase inhibitors and propranolol 1 month before randomization; any 5-hydroxytryptamine (5-HT)$_{1B/1D}$ agonist, ergot-type medication, opiates, or barbiturates 24 hours before treatment with study medication; and non-opiate analgesics and antiemetics 6 hours before treatment with study medication. In addition, daily analgesics taken for any reason were not permitted (except for aspirin 325 mg/day).

**Carpay 2004**	Between 18 and 65 years of age, had ≥1-year history of migraine with or without as defined by the 1988 International Headache Society (IHS)-criteria, had 1 to 6 migraines monthly during the two months preceding the screening visit, and had a history of moderate to severe migraines typically preceded by a mild-pain phase.	Patients were excluded if they had >6 migraines monthly during either of the 2 months before screening; uncontrolled hypertension (diastolic blood pressure ≥95 mm Hg or systolic blood pressure ≥160 mm Hg); suspected or confirmed cardiovascular or cerebrovascular disease; or ophthalmic, basilar, or hemiplegic migraine. Other exclusion criteria included use of migraine prophylactic medication containing ergotamine. an ergot derivative, or methysergide; use of a monoamine oxidase inhibitor within 2 weeks before the study; and. in countries where the combination of a selective serotonin reuptake inhibitor and a triptan is not allowed, need for a selective serotonin receptor inhibitor during the study.
**Chung 2006**	Outpatients with migraine, not further specified	Not reported.
**Codispoti 2001**	(1) Men or women aged 18 years or older; (2) history of migraine with at least moderate pain intensity on a scale of none. mild. moderate. or severe that met the International Headache Society (IHS) diagnostic criteria for migraine with or without aura; (3) fulfillment of the criteria for migraine after administration of a semi-structured diagnostic headache interview (Clinical Assessment Form for Migraine Headache. Innovative Medical Research. Baltimore; Md (4) migraine attack frequency of at least one episode every 2 months but not more than six episodes per month in the year before study entry; (5) history of treatment of previous migraines with over-the-counter medications; (6) ability to differentiate a migraine headache from an interval (tension-type) headache; and (7) if a woman. postmenopausal or using an effective form of birth control for at least 3 months before study entry.	(1) History of severely incapacitating migraines with more than 50% of episodes requiring bed rest or prohibiting performance of daily activities; (2) more than 20% of migraine episodes included vomiting; (3) routine experience of any other type of headache that would confound discrimination from migraine headache; (4) history of headaches due to other underlying pathology or related to head or neck trauma; (5) history of alcohol abuse, drug dependency or history of significant psychiatric illness in the 12 months before study entry; (6) history of clinically significant renal or hepatic disease; uncontrolled hypertension; clinically significant coronary vascular disease not stable for the past 6 months; history of seizures. cerebral ischemia. infarct. hemorrhage or other central nervous system disease; unstable metabolic disease. hypoglycemia. or diabetes; malignancy within the past 5 years; active tuberculosis; or prior gastrointestinal surgery which could influence the absorption. metabolism. or excretion of study medication; (7) history of allergy or sensitivity to ibuprofen. aspirin. or other NSAIDs; (8) current use of NSAIDs. aspirin. or analgesics on a regular basis (more than 15 days per month). except low-dose (325 mg daily or less) aspirin for cardiovascular prophylaxis; or (9) previous enrollment in this study. a recent (within 1 year) McNeil migraine headache study. or any other investigational drug study (within 30 days), conditions: (10) history of severely incapacitating migraines with more than 50% of episodes requiring bed rest or prohibiting performance of daily.
**Croop 2019**	Eligible participants included men and women aged 18 years and older with at least a 1-year history of migraine with or without aura according to the criteria of the third edition of the International Classification of Headache Disorders (beta version); migraine onset before age 50 years; at least two and not more than eight migraine attacks of moderate or severe intensity per month, and fewer than 15 days per month with migraine or non-migraine headache within the past 3 months. Participants had to be able to distinguish migraine attacks from attacks of tension-type and cluster headache, and those taking preventive migraine medication had to be on a stable dose for at least 3 months before study entry. If all other	Participants were excluded if they had any medical condition that might interfere with study assessments of efficacy and safety or expose participants to undue risk of a significant adverse event, as decided by the investigator (case by case). Participants were also excluded if they had been treated for or showed evidence of alcohol or drug abuse within the past 12 months; had a history of drug or other allergy that made them unsuitable for participation; or had electrocardiogram (ECG) or laboratory test findings that raised safety or tolerability concerns. Complete criteria for exclusion from the study are available in the study protocol

	criteria for inclusion were met, participants with contraindications to triptans could be included	
**CTRI/2010/091/001157 2010**	All subjects with duly filled and signed in ICFs (Informed Consent Forms). Male and female outpatients ≥18 years of age. Diagnosis of migraine, with or without aura. At least moderate pain with migraines. Average of 2 to 6 migraines per month for past 3 months. Duration of headache pain at least 4 hours. Able to tell the difference between a migraine and a tension headache If taking a medication to prevent migraines, patients must have been taking a maintenance dose for at least 30 days before screening. In generally good health. If female, using birth control. Patients who are able and are willing to comply with the protocol and have signed IEC or IRB approved Informed Consent Form	Patients unwilling to sign on ICF. Routinely experience other type of headache that might seem like a migraine headache. An average of 15 or more headache days per month in the past 6 months. Migraines began after age 50. Taking medicine for preventing migraines. Use of any drugs on list of prohibited drugs, of opioid drugs in past 7 days, of corticosteroids in the past 30 days, of an investigational drug within 30 days. Use of non-drug treatment for migraine in past 14 days unless have used this treatment for 14 days and plan to continue throughout study. Overuse of medications that treat pain or nausea. Migraine aura without headache. Hemiplegic or basilar migraines. Usually have vomiting with headache. Headaches that usually occur upon waking. Significant unstable medical disease. Abusing drugs or alcohol. History of a significant mental disorder. Pregnant or breast-feeding. Patients with clinically significant or unstable hepatic, respiratory, or hematologic illnesses, unstable cardiovascular disease, or symptomatic peripheral vascular disease. Patients with abnormal clinical chemistry, hematology, urinalysis, or ECG test results that are considered clinically significant by the investigator or the sponsor. Patients with an estimated creatinine clearance of 60 mL/minute based on serum creatinine levels will be excluded. Patients known to be positive for Hepatitis B, Hepatitis C, human immunodeficiency virus (HIV)-1 or HIV-2. Patients being treated with any of the excluded medications mentioned in the protocol. Patients with concomitant diseases such as malignancy, HIV. Participation in other trials or previous participation in this or any other almotriptan clinical trial.
**CTRI/2023/001/048905 2023**	1. Patients who are able and willing to give written informed consent and are at least 18 years of age at time of screening visit with migraine with or without aura fulfilling the IHS diagnostic criteria 1.1 (migraine without aura) or 1.2.1 (migraine with aura). 2. History of disabling migraine for at least 1 year. 3. Migraine onset before the age of 50 years. 4. History of 3 to 8 migraine attacks per month (<15 headache days per month) during the past 3 months. 5. Migraine Disability Assessment (MIDAS) score ≥11. 6. Patients who are able and willing to complete subject dairy to record the details of each migraine attack treated with study drug. 7. Females of child-bearing potential must agree to use a highly effective method of contraception (that is, one with less than 1% failure rate) such as combined oral contraceptives, implanted/injected contraceptives, intrauterine devices (IUD) or sterile partner until 30 days after the last dose of study medication.	1. Known hypersensitivity to lasmiditan, or to any excipient of Lasmiditan oral tablets. 2. History or evidence of hemorrhagic stroke, epilepsy or any other condition placing the patient at increased risk of seizures. 3. History of recurrent dizziness and/or vertigo including benign paroxysmal positional vertigo (BPPV), Ménière's disease, vestibular migraine, and other vestibular disorders. 4. History of diabetes mellitus HbA1C >8% with complications (diabetic retinopathy, nephropathy, or neuropathy). 5. History of orthostatic hypotension with syncope. 6. Significant renal or hepatic impairment in the opinion of investigator. 7. History, within past 12 months of chronic migraine or other forms of primary or secondary chronic headache disorders (eg, hemicranias continua, medication overuse headache where headache frequency is ≥ 15 headache days per month). 8. Patients with use of more than 3 doses per month either opioids or barbiturates. 9. Initiation of or a change in concomitant medication to reduce the frequency of migraine episodes within 3 months prior to screening visit. 10. Female patients who are pregnant or breast-feeding. 11. Women of childbearing potential who test positive for pregnancy based on serum/urine pregnancy test collected at screening visit. 12. History of drug or alcohol abuse/dependence within 1 year prior to screening visit (excessive or compulsive use as judged by the investigator), or currently using drugs of potential abuse or any prescribed or over-the-counter medication in a manner that the investigator considers indicative of abuse/dependence. 13. Have an acute, serious, or unstable medical condition, or a history or presence of any other medical illness including but not limited to any autoimmune

		disease, cardiovascular (CV), hepatic, respiratory, hematological, endocrine, psychiatric, or neurological disease, or any clinically significant laboratory abnormality, that, in the judgement of the investigator, indicates a medical problem that would preclude study participation.
**Cutler 1995**	Men and women 18 to 65 years old with greater that 1 year history of migraine with or without aura, diagnosed according to International Headache Society (IHS)-criteria, were eligible for the study. Patients must have had 1 to 6 migraine attacks per month during the 2 months before screening.	Patients with a history of ischemic heart disease or with diastolic blood pressure greater than 95 mmHg or systolic blood pressure greater than 160 mmHg were excluded. Pregnant or breast-feeding women of childbearing potential who were not using adequate contraception were excluded.
**Dahlöf 1993**	Patients 18 to 65 years old, who experienced 2 to 8 migraine attacks with or without aura. per month and have had a migraine history for at least one year. Only migraineurs fulfilling the International Headache Society (IHS)-diagnostic criteria 1.1, 1.2 and 1.2.1 were included.	Patients with gastric or duodenal ulcer, history of intolerance to diclofenac or history of asthmatic attacks triggered by NSAIDs, ergotamine and/or analgesic addiction (more than 15 tablets/month or 10 suppositories/month, on concomitant NSAID therapy, pregnant or nursing women, those with frequent migraine with aura while on oral contraceptives, and childbearing women on insufficient contraception were excluded. Prophylactic medication for migraine was stopped at least two weeks prior to participation in the study.
**Dahlöf 1998**	Patients aged 18 to 65 years, with migraine (with or without aura) according to the International Headache Society Definitions (Headache Classification Committee of the International Headache Society, 1988), were eligible for inclusion. Patients were required to have suffered from migraine for at least 1 year, with an age of onset less than 40 years, an attack frequency of between 1 and 6 per month and no more than 6 days of non-migraine headaches per month.	A history of coronary artery disease or other vascular disease; Prinzmetal's angina; renal or hepatic disease; neurological or psychiatric disease; hypertension (usual systolic blood pressure ≥160 mmHg or diastolic blood pressure ≥95 mmHg)
**Dahlöf 2001**	History (1 year) of migraine attacks with or without aura, as defined by the International Headache Society. Adults aged 18 to 65 years, whose migraine attacks began before age 50, were eligible for enrollment. Additional inclusion criteria were migraine attacks occurring 1 to 6 times per month; a 24-hour headache-free period between attacks; and normal body mass index (17 to 30 kg/m2), systolic blood pressure (105 to 140 mm Hg), diastolic blood pressure (50 to 90 mm Hg), heart rate (50 to 90 bpm), and electrocardiogram (ECG) at initial screening.	Migraine with prolonged aura, familial hemiplegic migraine, and migrainous infarction or vertebrobasilar migraine; migraine patients with Raynaud's phenomenon; the occurrence of more than six headaches per month; and time between screening and migraine attack longer than 8 weeks. Other exclusion criteria included pregnancy, medical anomalies of major organ systems upon clinical or laboratory screening, history of drug abuse (including migraine medication), alcoholism, or mental retardation. Patients were not allowed to have taken any migraine medications, e.g., analgesics, nonsteroidal anti-inflammatory drugs (NSAID), 5-hydroxytryptamine (5-HT)$_{1B/1D}$ receptor agonists, dopamine antagonists, for 2 days before intake of study medication. The use of any anti-psychotic or antidepressant medication 3 months before study enrollment, or any investigational drug 1 month before study enrollment was prohibited. Patients with a known intolerance or hypersensitivity to sumatriptan or other 5-HT$_{1B/1D}$ receptor agonists were excluded from the study.

**Dahlöf 2009**	Written informed consent required. Either sex between 18 and 65 years. Women of childbearing potential must not have been at risk of pregnancy and, if taking oral contraception, must have done so for at least 3 months prior to screening. Migraine attacks for at least 1 year before screening. Diagnosis of migraine by a neurologist within 1 month of screening visit (International Headache Society criteria). One to 6 attacks each month, with at least 48 h between attacks, in the 6 months before screening Age <50 years when migraine attacks began.	Serious adverse reactions or contraindications to sumatriptan. Treated non-migrainous headaches with analgesia more than 10 days/month over the 6 months before screening. Could not reliably distinguish between migraines and headaches of different etiology. Pregnant or breastfeeding women. Historic/current evidence of alcohol or drug abuse. Prior misuse/overuse of analgesic, opioid and/or antimigraine treatments within the past 3 months. Clinically significant cerebrovascular disease. Major psychiatric disorder. Consistent failure to respond to conventional acute-migraine therapies Diagnosis of vertebrobasilar or hemiplegic migraine. Clinically significant cardiovascular disease. Clinically significant abnormalities in screening laboratory sample. Previous inclusion in a tonabersat study. Previous inclusion in more than one clinical migraine study within the past year. Use of an investigational drug (for any indication) within 30 days, or 5 half-lives. Any other clinically significant condition. Concurrent use of valproic acid, calcium antagonists or flunarizine (any indication). Vomiting regularly (>20% of the time) within 2 h after taking oral medication during a migraine attack.
**Dib 2002**	Diagnosis of typical migraine with or without aura according to the criteria of the International Headache Society age between 18 and 65, presence of migraine for at least 1 year with onset observed before age 50, and frequency of migraine attacks of 1 to 6 days per month in the 3 months preceding the study. Patients needed to be able to recognize the signs of onset of a migraine attack, and to distinguish these attacks from tension headaches.	Atypical forms of migraine; the occurrence of tension headaches more often than six times a month; regular vomiting or absolute gastric intolerance within 30 minutes of migraine onset; identified contra-indications to ketoprofen or zolmitriptan; psychiatric comorbidity; alcohol or substance abuse; consumption of antimigraine drugs more frequently than 10 days a month; and use of NSAID, 5-hydroxytryptamine 1D (5-HT_{1D}) receptor agonists, or prophylactic ergot antimigraine drugs. Pregnant women and those breast-feeding or not using an effective contraceptive method were excluded.
**Diener 2002**	Otherwise healthy male or female patients were eligible for the study if they were aged 18 to 65 years and had experienced migraine with or without aura (as defined by the International Headache Society) for at least 1 year and with onset before the age of 40 years. For patients to be included in the study, their frequency of migraine attacks had to be at least 1 every 6 weeks but not more than 6 per month	Frequent non-migrainous headaches (more than 6 per month on average); atypical migraine that had consistently failed to respond to medical therapy; migraine with prolonged aura; familial hemiplegic migraine; basilar migraine; migrainous infarction; known coronary artery disease; clinically significant arrhythmias; heart failure; uncontrolled hypertension (presence of any hypertension in patients enrolled in Germany); peripheral vascular disease or Raynaud’s syndrome; clinically significant active systemic, renal, hepatic, gastrointestinal, neurological, endocrine, metabolic or psychiatric disease; severe limitation of gastrointestinal absorption; serious documented drug allergy; alcohol or substance misuse; and regular excessive use of analgesics or ergotamine (intake on more than 2 days in 7). Female patients who were pregnant, breast-feeding or at risk of pregnancy because of ineffective contraceptive precautions were not considered for entry. Patients who were intolerant of Cafergot or its constituents or who were taking medication contraindicated with Cafergot were also ineligible. Otherwise, patients who were taking migraine prophylactic drugs were acceptable. Use of analgesics or antiemetics in the 6 h before treatment, or sumatriptan or ergot derivatives in the 48 h prior to therapy, was not permitted.
**Diener 2004a**	Male and female patients between the ages of 18 and 65 years were recruited at general practitioners and neurology clinics throughout Germany. All patients had a migraine with or without aura as defined by the International Headache Society 1988 criteria present for more than 1 year and a minimum average of 1 attack per month, but not more than 6 attacks per month. Patients were recruited to comply with all study procedures, including the completion of the diary cards, and to be able to distinguish nonmigraine	Not reported.

	headache from typical migraine. All patients provided written informed consent. At the time of treatment of the migraine attack, each of the following associated symptoms must be present: nausea, photophobia and phonophobia. Migraine headache must be of moderate or severe intensity and no aura present.	
**Diener 2004b**	Patients (18 to 65 years) were eligible for the study if they gave informed consent in writing and if they met the International Headache Society (IHS) diagnostic criteria for migraine with or without aura. Patients were required to have a history of migraine of at least 1 years and between 1 and 6 attacks per month.	Patients having at least one of the following criteria were excluded: participation in a study during 4 weeks prior to the start of the study; all other types of headache, including tension-type headache; hypersensitivity to acetylsalicylic acid, salicylates, ibuprofen, nonsteroidal anti-inflammatory drugs or sumatriptan; peptic ulceration or gastric bleeding; haemorrhagic diathesis; disorders of kidney, liver, lung, heart, or brain function; neurological disorders; hypertension; coronary heart disease and/or history of myocardial infarction; pregnant or lactating women or women of childbearing age not using contraception; drug or alcohol abuse and prohibited concomitant medication.
**Diener 2005**	Male or female, aged 18 to 65 years, who met the IHS diagnostic criteria for migraine with or without aura, who had disease duration of at least 1 year and had 2 to 6 migraine attacks per month over the previous 3 months. Who were females and for whom current or future pregnancy (at least 2 months after completing the trial) could be excluded, who were not lactating. Had to have completed a written voluntary informed consent form.	Suffering from interval headaches, other types of migraine. Receiving prohibited medication or with a known hypersensitivity to the active substance or its excipients or other chemically closely related substances, particularly acetylsalicylic acid and in general other analgesics, antipyretics and NSAIDs. In whom attacks of asthma, urticaria, or acute rhinitis were precipitated by acetylsalicylic acid or other drugs with prostaglandin-synthetase inhibiting activity. With severe cardiac, liver or acute renal insufficiency, with active peptic ulcer disease or a history of significant gastrointestinal disease or gastrointestinal bleeding over the past year. With phenylketonuria, porphyria, active blood dyscrasia, bone marrow depression or clinically significant findings on an electrocardiogram (ECG).
**Diener 2011**	Male and female subjects were eligible for the study if they were 18 and 65 years of age, had a history of migraine with or without aura (International Classification of Headache Disorders, 2nd edition), for at least one year and were in general good health. In the three months prior to the screening visit, subjects had to have two to eight migraine attacks of moderate to severe intensity per month, each lasting at least six hours	Subjects were excluded if they fulfilled contraindications listed in the eletriptan summary of product characteristics, suffered from other pain syndromes, were taking migraine prevention medication or other pain medication on more than 10 days per month, were classified as treatment resistant by the investigator or had signs of liver injury (history of severe hepatic disease or elevated liver function test >2x upper limit of normal [ULN]). Subjects on benzodiazepines, antidepressants or potent CYP3A4 inhibitors (cytochrome P450) were not allowed to participate in the trial.
**Dodick 2019**	1. Written informed consent and patient privacy information (eg, Written Authorization for Use and Release of Health and Research Study Information) obtained from the patient prior to initiation of any study-specific procedures. 2. Male or female patients ages 18 to 75 years, inclusive, at Visit 1. 3. At least a 1-year history of migraine with or without aura consistent with a diagnosis according to the International Classification of Headache Disorders, 3rd edition, beta version (ICHD-3 beta, 2013; Section 12.1.1). 4. Migraine onset before age 50. 5. By history, the patient's migraines typically last between 4 and 72 hours if untreated or treated unsuccessfully and migraine episodes are separated by at least 48 hours of headache pain freedom. 6. History of 2 to 8 migraine attacks per month with moderate to severe headache pain in each of	1. Woman is pregnant, planning to become pregnant during the course of the study, or currently lactating. Women of childbearing potential must have a negative urine pregnancy test at Visit 1 and Visit 2. 2. Difficulty distinguishing migraine headache from tension-type or other headaches. 3. History of 15 or more headache days per month on average in the 6 months prior to Visit 1 in the investigator's judgment, or a current diagnosis of chronic migraine as defined by International Classification of Headache Disorders (ICHD)-3 beta (Section 12.1.1). A headache day is defined as a day in which there was any occurrence of a headache of a minimum duration of 2 hours or a headache of any duration for which acute medication was taken. 4. Has taken medication for acute treatment of headache (including acetaminophen, nonsteroidal anti-inflammatory drugs [NSAIDs], triptans, ergotamine, opioids, or combination analgesics) on 10 or more days per month in any of the 3 months prior to Visit 1. 5. Has a history of migraine aura with diplopia or impairment of level of consciousness, hemiplegic migraine, or retinal migraine as defined by ICHD-3 beta (Section 12.1.1). 6. Has

	the 3 months prior to Screening (Visit 1). 7. Patients must be using a medically acceptable and highly effective method of birth control defined in Section 4.5.1.2. 8. Be able to read, understand and complete the study questionnaires and eDiary.	a current diagnosis of new persistent daily headache, trigeminal autonomic cephalgia (eg, cluster headache), or painful cranial neuropathy as defined by ICHD-3 beta (Section 12.1.1). 7. Required hospital treatment of a migraine attack 3 or more times in the 6 months prior to Visit 1. 8. Requirement for any medication or diet (ie, grapefruit juice) that is on the list of prohibited concomitant medications (see Sections 4.5.2 and 12.2) that cannot be discontinued or switched to an allowable, alternative medication, including certain medications that require stable dosing before Visit 1. 9. Has a chronic non-headache pain condition requiring daily pain medication (with the exception of pregabalin). 10. History of hypersensitivity or clinically significant adverse reaction to a CGRP receptor antagonist. 11. An ECG with clinically significant abnormalities as determined by the investigator at Visit 1 12. A QTcF > 450 msec for males or QTcF > 470 msec for females at Visit 1. 13. Clinically significant cardiovascular or cerebrovascular disease per the investigator's opinion including, but not limited to: Clinically significant ischemic heart disease (eg, unstable angina pectoris). Clinically significant cardiac rhythm or conduction abnormalities (eg, atrial fibrillation, second- or third-degree heart block) or risk factors for Torsade de Pointes (eg, heart failure, hypokalemia, bradycardia). Myocardial infarction, transient ischemic attack, or stroke within 6 months prior to Visit 1. Heart failure defined as New York Heart Association functional classification system, Class III or IV. Hypertension as defined by sitting systolic blood pressure > 160 mm Hg or sitting diastolic blood pressure > 100 mm Hg at Visits 1 or 2. Vital sign measurements that exceed these limits may be repeated only once. 15. Clinically significant abnormalities (as determined by the investigator) in physical examination or laboratory safety test at Visit 1 as per guidelines below: ALT or AST greater than 1.5 times the ULN OR Total bilirubin greater than 1.5 mg/dL (except for patients with a diagnosis of Gilbert's disease) OR Serum albumin lower than 2.8 g/dL. Positive result on the urine drug screen at Visit 1 unless explained by concomitant medication use (eg, opioids prescribed for migraine pain).17. Any clinically significant hematologic, endocrine, pulmonary, renal, hepatic, gastrointestinal, or neurologic disease. If there is a history of such disease but the condition has been stable for more than 1 year prior to Visit 1, and is judged by the investigator as not likely to interfere with the patient's participation in the study, the patient may be included. Patients on dialysis for renal failure are excluded. 18. In the opinion of the investigator, other confounding pain syndromes, confounding psychiatric conditions, dementia, epilepsy or other significant neurological disorders other than migraine 19. Significant risk of self-harm, based on clinical interview and responses on the C-SSRS, or of harm to others in the opinion of the investigator; patients must be excluded if they report suicidal ideation with intent, with or without a plan, (ie, Type 4 or 5 on the C-SSRS) in the past 6 months or report suicidal behavior in the last 6 months prior to Visit 1 or Visit 2 assessments (see Section 6.5.7 for details). 20. History of malignancy in the 5 years prior to Visit 1, except for adequately treated basal cell or squamous cell skin cancer, or in situ cervical cancer. 21. History of gastric or small intestinal surgery (including gastric bypass surgery or banding), or has a disease that causes malabsorption (eg, Crohn's disease). 22. History of acute hepatitis within 6 months of Screening (Visit 1) or chronic hepatitis (including nonalcoholic steatohepatitis) or a positive result on anti-hepatitis A immunoglobulin M (IgM) antibody, hepatitis B surface antigen, or anti–hepatitis C antibody testing at Screening (Visit 1). 23. At Visit 1, a user of recreational or illicit drugs or has had a history within the past year

		of drug or alcohol abuse or dependence. 24. Currently participating or has participated in a study with an investigational compound or device within 30 days prior to Visit 1 (this includes studies using marketed compounds or devices). If patient is currently participating in or has participated in a study with injectable monoclonal antibodies blocking the CGRP pathway, the patient may participate in this study if at least 3 months have passed since the last injection. 25. Previously exposed to MK-8031 (investigational CGRP receptor antagonist) 26. Employed by or is an immediate family member (parents, spouses, siblings or children) of one of the investigators, study staff, or Allergan. 27. Any medical or other reasons (eg, unlikely to adhere to the study procedures, keep appointments, or is planning to relocate during the study) that, in the investigator's opinion, might indicate that the patient is unsuitable for the study.
**Dowson 2002a**	Male and female migraine patients aged between 18 and 65 years who met the International Headache Society criteria for migraine with or without aura were eligible to participate in the study. Patients had at least a 1-year history of one to six migraine attacks per month, with an age of onset of migraine of less than 50 years and at least 24 h free from headache between their attacks. Female patients of childbearing age were not pregnant on testing and practiced contraception. Patients were tested with an electrocardiogram (ECG) and were enrolled if there were no abnormalities of clinical relevance.	Patients were excluded from the study if they had migraine with prolonged aura, familial hemiplegic migraine, migrainous infarction, vertebrobasilar migraine or Raynaud's phenomenon associated with migraine, or any other significant medical condition. In addition, patients suffering from cardio vascular disease (cardiac ischaemia, atherosclerosis, cardiac arrhythmia or hypertension (blood pressure; > systolic 160 mmHg, diastolic >95 mmHg)), alcoholism, drug abuse or mental retardation were also excluded from the study. Patients taking certain drugs were not permitted to enter the study (any investigational drug 1 month before study medication; monoamine oxidase inhibitors, lithium, selective serotonin reuptake inhibitors, ergots or derivatives or methysergide in the 2 weeks prior to study medication.
**Dowson 2002b**	Female and male patients aged 18 to 65 years with an established diagnosis of migraine (as defined by International Headache Society criteria) were recruited. All patients were required to have an age at migraine onset of <50 years, to have experienced ≥1 migraine headache per month during the 3 months prior to the study, and to be able to distinguish between migraine and non-migraine headaches.	Patients were excluded if they had a history of basilar, ophthalmoplegic or hemiplegic migraine, or if they reported non-migraine headaches on ≥6 days per month in the preceding 6 months. Other exclusion criteria were: pregnancy, lactation or inadequate contraception in female patients; history or symptoms of ischaemic heart disease (or other vascular disease, including Prinzmetal's angina), dysrhythmias or cardiac accessory pathway disorders (e.g. Wolff-Parkinson-White syndrome); uncontrolled hypertension; use of monoamine oxidase A inhibitors, methysergide or methylergonovine within 2 weeks before randomization; a recent history of alcohol or drug abuse; known hypersensitivity or adverse reaction to study medication or other 5-hydroxytryptamine (5-HT)$_{1B/1D}$-receptor agonists; phenylketonuria; severe hepatic impairment; or participation in another clinical trial during or within 30 days prior to this study.
**Dowson 2005**	All patients were women aged between 18 and 50 years who self-reported to the investigator with menstrually-related migraine (based on patient recall of having experienced a menstrually-related migraine attack in two of their last three menstrual cycles and >80% of their attacks falling within the menstrual window in the previous 6 months). Patients presented with a history of one to four moderate or severe migraine attacks with or without aura per month (in accordance with the International Headache Society [IHS] diagnostic criteria pertaining at the time). The majority of attacks (at least 80%) were reported to occur during the menstrual window (the 8 days starting 3 days before the onset of menstruation). Prophylactic migraine therapy and hormonal therapy were kept	Patients were excluded from the study if they were pregnant or likely to become pregnant during the study, were lactating, or had hypertension (supine diastolic pressure >95 mmHg), ischaemic heart disease, atherosclerotic disease or other concurrent medical conditions that could affect the study data or for which there was a medical contraindication. Patients were also excluded if they were regularly taking analgesics, anti-emetics or antihistamines, abused ergotamine or alcohol, or had a hypersensitivity to, intolerance of, or contraindication to the use of sumatriptan.

	constant for 3 and 6 months, respectively, prior to the study and throughout the study.	
**Eletriptan Steering Committee in Japan 2002**	Randomized patients were males or females who were neither pregnant nor breast-feeding and ranged in age from 18 to 64 years. Based on their previous medical history, patients were entered if they reported at least one acute migraine attack with or without aura every 6 weeks. Patients had to have a headache of severe or moderate intensity prior to dosing.	Patients had to have a headache of severe or moderate intensity prior to dosing and were not permitted to use any analgesics (including dimetotiazine mesylate) or antiemetics within 6 h before or within 2 h post-dosing. Furthermore, they could not have received ergotamine or an ergotamine-like agent within 24 h before or after dosing and/or if dosing occurred less than 6 h after the onset of their headache. Patients were excluded if they had severely limited gastrointestinal absorption (e.g. total gastrectomy). To ensure reliable 'bridging', inclusion and exclusion criteria were identical to those used in previous clinical studies.
**Ensink 1991**	Migraine with or without aura diagnosed according to International Classification of Headache Disorders (ICHD) criteria. Age between 18 to 65 years. At least a 1-year history of migraine.	Prophylactic meds within 2 weeks of study start. pregnant or lactating. Hypertensive (resting diastolic >95 mmHg. ischemic heart disease or any medical condition (renal or hepatic impairment) that might interfere with interpretation of the study results or if they had a current or recent history of drug abuse including alcohol).
**EUCTR2006-000785-36 2008**	A subject is eligible for inclusion in this study only if all of the following criteria apply: 1. ambulant male and non-pregnant female subjects; 2. ≥18 and ≤65 years of age at the randomisation visit; 3. with a current history of migraine with or without aura according to the International Headache Society (IHS)-criteria; 4. having experienced an average of at least one but not more than six migraine attacks per month for 6 months prior to entry into the study; 5. willing and able to understand and complete the anticipated study questionnaires; 6. willing and able to sign the informed consent prior to entry into the study.	History suggestive of ischemic heart disease (IHD; e.g. myocardial infarction, angina pectoris, coronary vasospasm, vasospastic [Prinzmetal's variant] angina) or any atherosclerotic disease (e.g. peripheral vascular disease) indicating an increased risk of coronary ischemia; for patients with risk factors for CHD (especially for smokers, patients with diabetes mellitus, males > 40 years of age, postmenopausal females, patients with bundle branch heart block and patients with CHD in their family anamnesis) the investigator had to consider carefully study participatìon with special attention to ECG results and anamnesis data [as per locaI Amendment in Germany dated 21 May 2007] symptomatic Wolff-Parkinson-White syndrome or cardiac arrhythmias associated with other cardiac accessory conduction pathway disorders history of stroke or transient ischemic attack (TIA) uncontrolled hypertension; moderate severe or severe hypertension and uncontrolled slight hypertension (systolic blood pressure >160 mmHg/diastolic blood pressure >100 mmHg) [as per Local Amendment in Germany dated 21 May 2007] of history of basilar, hemiplegic or ophthalmoplegic migraine, severe liver impairment (i.e., Child-Pugh score ≥ 7), severe renal impairment (i.e., Creatinine Clearance [CrC1] <26 mL/min), renal disease, or renal failure, known or suspected intolerance of, or hypersensitivity or contraindications to any component of the trial medications, including inert substances (e.g. intolerance to galactose, Lapp's lactase deficiency, malabsorption of glucose-galactose, phenylketonuria) use of either test medication to treat any one of the last three episodes of migraine history of intolerance or inefficacy of at least two triptans for the treatment of migraine attacks current use of propanolol or ergotamine or its derivatives current use or use within the last 2 weeks of monoaminooxidase (MAO)-inhibitors abuse of alcohol, analgesics or psychotropic drugs severe concurrent medical condition that may affect the interpretation of clinicial trial results pregnancy or breastfeeding participation in a clinical trial, currently or within the previous month, inability or refusal to issue the

		informed consent, more than six days of tension-type headache signs of CHD in baseline electrocardiogram (ECG).
**EUCTR2006-000805-42 2008**	1. Ambulant male and non-pregnant female subjects; 2. ≥18 and ≤65 years of age at the randomisation visit; 3. with a current history of migraine with or without aura according to the IHS criteria; 4. having experienced an average of at least one but not more than six migraine attacks per month for 6 months prior to entry into the study; 5. willing and able to understand and complete the anticipated study questionnaires; 6. willing and able to sign the informed consent prior to entry into the study.	1. History suggestive of ischaemic heart disease (IHD; e.g. myocardial infarction, angina pectoris, coronary vasospasm, vasospastic [Prinzmetal's variant] angina) or any atherosclerotic disease (e.g. peripheral vascular disease) indicating an increased risk of coronary ischaemia; 2. symptomatic Wolff-Parkinson-White syndrome or cardiac arrhythmias associated with other cardiac accessory conduction pathway disorders; 3. history of stroke or transient ischaemic attack (TIA); 4. uncontrolled hypertension; 5. history of basilar, hemiplegic or ophthalmoplegic migraine; 6. severe liver impairment (i.e., Child-Pugh C); 7. severe renal impairment (i.e., CrCl <26 ml/min), renal disease, or renal failure; 8. known or suspected intolerance of, or hypersensitivity or contraindications to any component of the trial medications, including inert substances (e.g. intolerance to galactose, Lapp's lactase deficiency, malabsorption of glucose-galactose, phenylketonuria); 9. use of either test medication to treat any one of the last three episodes of migraine; 10. history of intolerance or inefficacy of at least two triptans for the treatment of migraine attacks; 11. current use of ergotamine (or its derivatives) as a prophylactic agent; 12. current use or use within the last 2 weeks of monoamine oxidase (MAO)-inhibitors; 13. abuse of alcohol, analgesics or psychotropic drugs; 14. any severe concurrent medical condition that, according to the site Investigator, may affect the interpretation of clinical trial results; 15. pregnancy or breastfeeding; 16. participation in a clinical trial within the previous month or current participation in any other clinical research study or clinical trial; 17. inability or unwillingness to issue the informed consent; 18. more than six days of tension-type headache.
**Freitag 2007**	The study enrolled adults with at least a 6-month history of migraine with or without aura who typically experienced migraine-associated nausea by the time their headache became moderate or severe. Patients had to be able to distinguish between migraine and other types of headache. Women were required to use adequate contraception. Antimigraine prophylactic medications other than propranolol were permitted. No prohibited medications were allowed, including non-opiate analgesics and antiemetics 6 hours before treatment. Patients had to be in generally good health, with no cardiovascular disease and no other confounding health conditions	Heart disease, high blood pressure.
**Freitag 2008**	The study enrolled patients with at least a 6-month history of migraine with or without aura as defined according to the International Headache Society (IHS) criteria. Patients included men and women (not pregnant or nursing), at least 18 years of age, who had the ability to distinguish between migraine attacks and other types of headache.	Patients were excluded from participation in the study if they had more than 6 migraine attacks per month or typically had greater than 10 headache days per month or if they had a history of hemiplegic or basilar migraine. Those who had daily or almost daily (typically >3 days of 7 days) use of NSAIDs, cyclooxygenase (COX)-2 inhibitors, or other analgesics; monoamine oxidase inhibitors; or propranolol were excluded. Patients also were excluded if they had a history or clinical evidence of ischemic heart disease, coronary artery vasospasm (including Prinzmetal's variant angina), or other significant underlying cardiovascular disease or

		uncontrolled hypertension or clinical evidence of significant pulmonary, renal, hepatic, endocrine, neurologic (other than migraine), psychiatric, or any other condition that would pose an additional risk or interfere with optimal participation in the study, or if they had demonstrated hypersensitivity to or experienced a serious adverse event in response to rizatriptan, acetaminophen, or any of their inactive components.
**Färkkila 2003**	Male and female subjects age ≥18 years were invited to participate if they met the International Headache Society (IHS) diagnostic criteria for migraine, with or without aura, and could reasonably expect to suffer at least one acute attack of migraine every 6 weeks. Patients were required to have discontinued therapy with oral sumatriptan at least 2 weeks, but not longer than 2 years, prior to the screening visit. In general, subjects had been in the practices of the investigators for a significant period of time and their lack of sufficient response to sumatriptan was documented in the patient notes, along with the use of other therapies, including other triptans, during the last 6 months before screening. Patients were asked to give one of the following reasons for stopping the treatment with sumatriptan: slow onset of action, inconsistent response, poor overall efficacy, recurrence or tolerability. Female subjects were required to be adequately protected against pregnancy.	Pregnancy or breastfeeding, known coronary artery disease, significant arrhythmias, heart failure, significant electrocardiogram (ECG) abnormalities, and uncontrolled hypertension. Any significant systemic, organ, neurological, endocrine, metabolic, and psychological disorders reported by the patient or discovered during the physical examination also resulted in exclusion. Patients considered to have atypical migraine such as frequent attacks, prolonged aura or any migraine that was considered atypical were not included in the study. Patients who, during the course of the trial, required treatment with sumatriptan or any other 5-hydroxytryptamine (5-HT)$_{1B/1D}$ agonist in addition to study medication were also excluded.
**Färkkila 2012**	Men or women (18 to 65 years) who had at least a 1-year history of migraine with or without aura (according to International Headache Society criteria 1.1 and 2.1) with onset before the age of 50 years and one to eight migraine attacks per month were eligible for enrolment	Patients taking prescription or herbal migraine prophylaxis, vasoactive drugs, serotonin reuptake inhibitors, or known cytochrome P450 inhibitors. Prescription preventative migraine drugs were dis continued at least 15 days (flunarizine 30 days) before screening.
**Gallagher 2001**	Patients enrolled into the study had established diagnoses of migraine according to the criteria of the International Headache Society (IHS) with a history of migraine attacks for at least 1 year. Women were asked to use a reliable method of contraception.	Patients were excluded from the study if they had evidence or history of ischemic heart disease, arrhythmia, or accessory conduction pathway disorders (eg, Wolff-Parkinson-White syndrome); hypertension (systolic BP>160 mm Hg or diastolic BP>95mm Hg); or any condition that may have put them at increased risk on exposure to study medication or that may have interfered with efficacy or safety assessments. Patients with a history of basilar, ophthalmoplegic, or hemiplegic migraine; with nonmigraine headache for 10 or more days per month in the previous 6 months; or who were using monoamine oxidase inhibitors, methysergide, methylergonovine, fenfluramine, or dexfenfluramine were also excluded. Other exclusion criteria included drug or alcohol abuse, clinically abnormal laboratory results at screening, lactation, unacceptable adverse events (in the opinion of the investigator) following previous use of any 5-hydroxytryptamine (5-HT)$_{1B/1D}$ receptor agonist, simultaneous participation in another clinical trial, or treatment with another investigational drug within 30 days before screening.

**Garcia-Ramos 2003**	Male or female adults, aged 18 to 80 years, were eligible for study entry if they met International Headache Society (IHS) criteria for migraine with or without aura and reported a minimum of 1 acute migraine attack every 6 weeks.	Patients were excluded from the study if they reported (1) coronary artery disease, heart failure, uncontrolled hypertension or abnormal ECG; (2) frequent migraine or concomitant non-migrainous headache (>6 per month), migraine variants (e.g. familial hemiplegic or basilar migraine), and/or migraines which, in the clinical judgement of the investigator, had consistently failed to respond to adequate medical therapy; (3) hypersensitivity or known contra-indication to treatment with eletriptan or naratriptan; (4) concomitant use of potent CYP3A4 inhibitors or use of monoamine oxidase (MAO) inhibitors in the 2 weeks prior to study entry; (5) any clinically significant medical illness or laboratory abnormalities; (6) severe reduction in gastrointestinal absorption; (7) misuse or abuse of alcohol or other substances, including analgesics or ergotamine; (8) use of any experimental drug within the past month; (9) (if female) current pregnancy, breast-feeding, or not using a medically accepted form of contraception.
**Geraud 2000**	Male or female outpatients (18 to 65 years) with an established diagnosis of migraine with or without aura were recruited from 106 centres in 20 countries. Patients were eligible for inclusion if they had a history of migraine of at least 1 year's duration, an age at migraine onset of <50 years and had experienced an average of 1 to 6 attacks per month for the 6 months preceding the study. In addition, patients were required to be able to distinguish migraine from other types of headache and to comply with study procedures including completion of a patient diary. Only patients who had never taken sumatriptan or zolmitriptan were eligible for inclusion. The rationale for this was to alleviate the potential for bias by patients having preconceived ideas/experience regarding the efficacy of the active treatments.	Patients were excluded if they had received regular treatment during the month preceding the study with psychoactive drugs (e.g. hypnotics, benzodiazepines, neuroleptics, antidepressants) or drugs with a clinically important action at a 5-hydroxytryptamine (5-HT) receptor. However, patients were permitted to use medications such as b-blockers, calcium channel blockers (excluding flunarizine), clonidine and valproic acid for migraine prophylaxis. Patients were also excluded from the study for the following reasons: history of drug or alcohol abuse, diagnosis of basilar, ophthalmoplegic or hemiplegic migraine, history or symptoms of ischaemic heart disease or other vascular disease, angina pectoris, Wolff-Parkinson-White syndrome or other cardiac accessory conduction pathways or dysrhythmias, uncontrolled hypertension, any medical or psychiatric condition which may have put the patient at risk or interfered with efficacy assessments, experience of non-migraine headaches on more than 6 days per month in the past 6 months, and participation in a clinical trial within 30 days of the study. In addition, women who were pregnant or lactating or who were not using adequate contraception were excluded from the study.
**Gijsmant 1997**	Migraine by International Headache Society (IHS)-criteria. Male and female aged 18 to 55 years, suffering from 1 to 8 migraines per month, with at least a 6-month history of migraine, and otherwise good health.	Not permitted to receive any investigational compound within 30 days, fluoxetine within 6 weeks, prophylactic anti-migraine treatment within 2 weeks, ergot derivative, isometheptene, or sumatriptan within 48 hours, opiates within 24 h, or any other analgesics within 6 h prior to treatment with any dose of study medication.
**Goadsby 1991**	Males and nonpregnant females using adequate contraception were eligible if they were between 18 and 60 years of age (inclusive), were able to give informed consent, had at least a 12-month history of migraine with or without aura (as defined by the 1988 International Headache Society criteria), with 1 to 6 migraines monthly, and had a history of moderate to severe pain during migraine attacks. Subjects could receive prophylactic anti-migraine medication provided this was continued throughout the trial.	Subjects were excluded if they had uncontrolled hypertension (diastolic blood pressure >95 mmHg) at screening; a history suggestive of ischaemic heart disease; other systemic disease (eg, epilepsy, hepatic, renal or heart disease). Other exclusion criteria included psychiatric illness, ergotamine abuse (=1mg per day for =three months) or other drug abuse (eg, regularly taking more than the recommended doses), and subjects regularly taking narcotic analgesics or subjects known for narcotic abuse.

**Goadsby 2000**	We included male and female subjects, 18 years of age and older, who met the International Headache Society (IHS) criteria for migraine with or without aura, who experienced at least one acute attack every 6 weeks.	Patients were excluded for the following reasons: >6 migraine attacks per month, frequent tension-type headaches, recent history of alcohol or other substance misuse (including analgesics or ergotamine), serious allergic reactions to drugs, use of any experimental drug within the past month, pregnant or breastfeeding women, severely limited gastrointestinal absorption, any medical condition that might interfere with the interpretation of the study results, coronary artery disease, heart failure, uncontrolled hypertension, and receiving medication specifically contraindicated with sumatriptan.
**Goadsby 2007**	Male and female patients aged 18 to 65 years with at least a 12-month history, with onset before age 50 years, of migraine with or without aura according to International Headache Society criteria and two to six migraine attacks per month in the 2 months preceding the trial were eligible to enroll. Patients were not excluded on the basis of previous triptan usage, including almotriptan and zolmitriptan, making encapsulation necessary for blinding purposes.	Hemiplegic or basilar migraine, tension-type headache >4 days/month, inability to distinguish between tension-type and migraine headache, a history of ischaemic heart disease, severe hypertension or uncontrolled mild-to-moderate hypertension, cerebrovascular disease, peripheral artery disease, moderate-to-severe renal or hepatic disease, pregnancy, lactation, a history of abuse of analgesics, ergot derivatives or triptans, and allergy or sensitivity to sulfonamides or triptans.
**Goadsby 2008**	Male or female. 18 to 65 years old with a minimum one year of migraine history (IHS criteria) of moderate or severe intensity and with a frequency of two to six attacks per month. for the past 3 months.	Contraindications to almotriptan or history of almotriptan discontinuation. Chronic daily headache or migraine frequency of more than 6 episodes per month. Onset of migraine after age of 50. or headache predominantly occurring on waking in the morning also excluded patients. Pregnant or breastfeeding patients were excluded. as were patients who planned to begin non-pharmacological approaches to treatment during the period of study.
**Goadsby 2019**	Males or females (≥18 years) who had at least a 1-year history of disabling migraine with or without aura (International Headache Society diagnostic criteria 1.1 and 1.2.1) (Headache Classification Committee of the International Headache Society, 2013), a Migraine Disability Assessment (MIDAS) score ≥11, onset before the age of 50 years, and three to eight migraine attacks per month were eligible for enrolment.	History of chronic migraine or other forms of primary or secondary headache disorder such as hemicrania continua, or medication overuse headache, where headache frequency is ≥ 15 headache days per month within the past 12 months; haemorrhagic stroke, epilepsy, or any other condition placing the patient at increased risk of seizures; recurrent dizziness and/or vertigo including benign paroxysmal positional vertigo, Ménière's disease, vestibular migraine, and other vestibular disorders; diabetes mellitus with complications (diabetic retinopathy, nephropathy or neuropathy); orthostatic hypotension with syncope; significant renal or hepatic impairment; current evidence of abuse of any drug, prescription or illicit, or alcohol within the previous 3 years; and patients who were at imminent risk of suicide by the Columbia Suicide Severity Rating Scale (C-SSRS) or had a suicide attempt within 6 months prior to the screening visit. In addition, patients who used more than three doses per month of either opioids or barbiturates or had initiation of or a change in concomitant medication to reduce the frequency of migraine attacks within 3 months prior to the screening visit were considered ineligible for study entry. Patients with cardiovascular risk factors were identified using the American College of Cardiology/American Heart Association guidelines, which identified factors with greatest predictive potential for a first cardiovascular event. They include age, total and high-density lipoprotein cholesterol, systolic blood pressure (including treated or untreated), diabetes, and current smoking status. In contrast to the first phase 3 trial, this trial did not exclude individuals with known coronary artery disease, clinically significant arrhythmia, or uncontrolled hypertension.

**Goldstein 1998**	A total of 1538 men and women (18 to 91 years), who met the International Headache Society (IHS) criteria for migraine with or without aura, were enrolled. All patients had at least a 6-month history of migraine and typically experienced one to eight attacks per month.	Patients were specifically excluded if there was clinical evidence of cardiovascular disease, hypertension, significant ECG abnormality, or if they had a known history (within 1 year) or current evidence of drug or alcohol abuse. Patients with any contraindication or sensitivity to sumatriptan or those who had received treatment with any other investigational compound or device within the past 30 days were excluded. Women who were pregnant or breast-feeding were also excluded.
**Goldstein 2005**	To be eligible for enrollment, subjects had to report an average of 1 to 8 migraine episodes per month that satisfied International Headache Society (IHS) diagnostic criteria for migraine with or without aura and were of at least moderate intensity if left untreated. In addition, subjects had to be able to distinguish migraine headaches from other headache types at the onset of an attack.	Subjects who reported vomiting during more than 20% of migraine episodes or who required bedrest during more than 50% of migraine episodes were excluded.
**Goldstein 2006**	A detailed clinical assessment, conducted by the investigator, ensured that each subject's headaches met International Headache Society (IHS) diagnostic criteria for migraine without aura (IHS 1.1) or migraine 1 with aura (IHS 1.2).1 In addition, each subject was at least 18 years old, was in good general health, and had experienced a migraine attack at least once every 2 months—but no more than 6 times monthly—during the prior 12 months. Untreated attacks were of at least moderate pain intensity.	Patients whose headache symptoms may have been caused or aggravated by recent head or neck trauma and patients with cluster headache, specific migraine variants, or other serious nonmigraine causes of headache were excluded. Patients who reported using analgesic drug products for headache on more than 12 days per month were also excluded. Unlike earlier studies of over-the-counter (OTC) medications for migraine, no patients were excluded based on the requirement for bed rest or the presence of frequent vomiting.
**Gomez-Mancilla 2014**	Diagnosis of moderate to severe migraine for at least 1 year. At least 1 migraine episode, but not more 15 migraine days per month. Past use of triptans. Migraine onset before 50 years of age	Diagnosis of basilar, ophthalmoplegic or hemiplegic migraine. More than 6 non-migraine headaches per month. Patients receiving migraine prophylaxis treatment. Patients receiving regular treatment with psychoactive drugs. Smokers. Patients with a very high or low body weight.
**Gruffyd-Jones 2001**	Male and female patients aged 18 to 65 years with an established diagnosis of migraine (Headache Classification Committee of the International Headache Society, 1988) were recruited. Patients were required to have: a history of migraine symptoms of at least 1 year duration, an age of migraine onset <50 years; a history of 1 to 6 attacks per month in the 2 months prior to the study and the ability to distinguish non-migraine headaches from typical migraine without aura. Eligible patients were able to comply with all trial procedures, including the completion of diary cards, and all provided written, informed consent.	Patients with basilar, ophthalmoplegic or hemiplegic migraine headache or non-migraine headache on more than 10 days per month over the preceding 6 months were excluded. Other exclusion criteria included: pregnancy, lactation or inadequate contraception in female patients; ischaemic heart disease (or other vascular disease, including Prinzmetal's angina), dysrhythmias or cardiac accessory pathway disorders (e.g. Wolf-Parkinson-White syndrome); uncontrolled hypertension (systolic blood pressure 160 mmHg or diastolic blood pressure 95 mmHg); use of monoamine oxidase inhibitors, methysergide, or methylergonovine within 2 weeks of randomization; an abnormal clinical laboratory result, recent history of alcohol or drug abuse, hypersensitivity to study treatments, previous unacceptable adverse event following use of 5-hydroxytryptamine (5-HT)$_{1B/1D}$ receptor agonists, participation in another clinical trial during or within 30 days prior to this study and risk of transmitting human immunodeficiency (HIV), any other sexually transmitted disease or hepatitis B. Patients could only be admitted to the trial once.

**Göbel 2000**	Males and nonpregnant females using adequate contraception of any race were eligible if they were between 18 and 65 years of age (inclusive), had a history of migraine with or without aura, for at least 12 months prior to the study as defined by the 1988 International Headache Society criteria, had an average of 2 to 6 attacks per month of moderate/severe migraine over the last 6 months, had experienced on average an incidence of migraine headache recurrence of ≥50% of successfully treated attacks following treatment with any acute migraine therapy over the last 6 months, and were able to distinguish migraine headaches from other headache types (e.g. tension-type headache).	Subjects were excluded if they had uncontrolled hypertension (sitting diastolic blood pressure ≥95 mmHg or systolic blood pressure ≥160 mmHg) at screening; a history of epilepsy or structural brain lesions which lowered the convulsive threshold; confirmed or suspected cardiovascular, peripheral vascular disease or impaired hepatic or renal function; basilar or hemiplegic migraine. Other exclusion criteria included known hypersensitivity to the study drug, use of migraine prophylactic medication containing ergotamine, an ergot derivative, or methysergide; use of a monoamine oxidase inhibitor within 2 weeks before screening; and, in countries where the combination of a selective serotonin reuptake inhibitor and a triptan is not allowed, the need for a selective serotonin receptor inhibitor. All subjects provided written informed consent.
**Göbel 2004**	Men or women aged 18 to 65 years were invited to take part in the study if they satisfied the criteria for migraine with or without aura for at least 12 months in accordance with the first edition of the headache classification of the International Headache Society. Attack frequency had to be between one and six attacks per month, and the patients had to be able to identify a migraine attack.	The exclusion criteria included other headache syndromes requiring medication therapy, migraine aura without headache, migraine with prolonged aura, familial hemiplegic migraine, basilar migraine, known hypersensitivity to the study medication, known hypersensitivity to pyrazolone and phenylbutazone, known genetic glucose-6-phosphate-dehydrogenase deficiency, acute intermittent porphyria, and other significant neurological or other disorders. Other exclusion criteria were pregnancy, breast feeding, psychological disorders, medication or alcohol abuse, and participation in another study in the 4 weeks preceding the screening appointment or during the course of the study.
**Havanka 2000**	Males and nonpregnant females using adequate contraception were eligible if they were of any race, between 18 and 55 years of age (inclusive), had at least a 12-month history of migraine with or without aura as defined by the 1988 International Headache Society criteria, had 1 to 6 migraines monthly during the 2 months preceding the screening visit, and had a history of moderate to severe pain during migraine attacks, and were able to distinguish migraine headaches from other headaches.	Subjects were excluded if they: had a history of ischaemic heart disease (IHD) (i.e. angina pectoris, history of myocardial infarction (MI) or documented silent ischaemia), Prinzmetal's angina, symptoms or signs consistent with IHD or evidence of IHD on the screening electrocardiogram; had a history of coronary vasospasm or atherosclerotic disease (cardiovascular disease, peripheral vascular disease or Raynaud's disease) which placed them at increased risk of coronary ischaemia; had supine systolic blood pressure >165mmHg (reduced to >140mmHg in protocol Amendment 5) and/or supine diastolic blood pressure >95mmHg (treated or untreated) after 15 minutes rest at the time of entry into the study; had evidence of severe hypertension (i.e. eye fundus changes at stage 3 or 4) at the time of entry to the study; had any severe concurrent medical condition which could affect the interpretation of efficacy and safety data or otherwise contraindicate participation in a clinical trial with a new chemical entity; had current abuse of opiate analgesics or other psychotropic drugs or history of abuse during the previous year; had current history within the previous year of ergotamine abuse (>10mg/week); had current or history within the previous year of alcohol abuse (>315g/week) or other drugs; had participated or planned to participate in a clinical trial within 1 month prior to entry or during the period of the study; were receiving monoamine oxidase inhibitors, 5-hydroxytryptamine (5HT) reuptake inhibitors, lithium or flunarizine dihydrochloride; had known hypersensitivity to sumatriptan or naratriptan; had used sumatriptan, dihydroergotamine or ergotamine-containing drugs during the 24 hours before or after study drug administration or analgesics and/or anti-emetics 6 hours before study drug administration; had used prophylactic migraine medication during the 2 weeks prior to study medication.

**Ho 2008a**	Patient has at least 1 year history of migraine (with or without aura). Females of childbearing years must use acceptable contraception throughout trial. Patient is in general good health based on screening assessment.	Patient is pregnant/breast-feeding (or is a female expecting to conceive during the study period). Patient has heart disease, uncontrolled hypertension (high blood pressure), uncontrolled diabetes or other significant disease. Patient has major depression, other pain syndromes that might interfere with study assessments, psychiatric conditions, dementia, or significant neurological disorders (other than migraine). Patient has a history of gastric or small intestinal surgery or has a disease that causes malabsorption. Patient has a history of cancer within the last 5 years.
**Ho 2008b**	Patients were eligible for the study if they were ≥18 years of age, had a history of migraine for at least 1 year, and in the 2 months prior to the screening visit had had one to eight moderate or severe migraine attacks per month with or without aura (International Headache Society criteria) that typically lasted 4 to 72 h untreated.	Pregnant/breast-feeding (or is a female expecting to conceive during study period). Has history or evidence of stroke/transient ischemic attacks, heart disease, coronary artery vasospasm, other significant underlying cardiovascular diseases, uncontrolled hypertension (high blood pressure), uncontrolled diabetes, or human immunodeficiency virus (HIV) disease. Has major depression, other pain syndromes that might interfere with study assessments, psychiatric conditions, dementia, or significant neurological disorders (other than migraine). Has a history of gastric, or small intestinal surgery, or has a disease that causes malabsorption Has a history of cancer within the last 5 years.
**Jelinski 2006**	Male and female migraine patients between the ages of 18 and 65 years were invited to participate in the clinical trial. International Headache Society (IHS) criteria, 1 to 6 migraine attacks per month in the two months prior to screening, and typically to experience moderate to severe migraine pain preceded by a mild pain phase. Capable of reading, comprehending, and completing subject questionnaires and willing to provide informed written consent to participate.	Confirmed or suspected ischaemic heart disease (IHD), cardiac arrhythmias, hypertension, impaired hepatic or renal function, and a history of cerebrovascular disease, congenital heart disease, ischemic abdominal syndromes, peripheral vascular disease, Raynaud syndrome, or epilepsy. Patients diagnosed with basilar migraine, hemiplegic migraine, cluster headache or who showed evidence of a rebound headache pattern caused by ergotamine or analgesic overuse in the three months prior to enrollment were excluded. Patients taking monoamine oxidase (MAO) inhibitor drugs were not enrolled. Pregnant or breast-feeding women and those not using adequate contraception were also excluded from the trial.
**Kaniecki 2006**	Male and female patients aged 18 to 65 years were eligible if they answered affirmatively to the question ‘Do you suffer from tension or stress headaches?‘. Patients meeting 1988 IHS diagnostic criteria for migraine with or without aura 14 in the diagnosis phase were eligible for the treatment phase if they (1) had had migraine for at least 1 year; (2) only had migraine headaches or, if they had multiple headache types, could differentiate migraine from the others; (3) experienced 1–6migraine headaches during each of the 2 months before screening; and (4) typically had migraine headaches of moderate to severe intensity.	Prior diagnosis of migraine or probable migraine and the presence of headache for at least 15 days monthly during either of the 2 months before screening. Patients were excluded if they (1) had ever used a triptan, ergotamine, or an ergot-derivative; (2) had persistent head or neck pain outside of migraine attacks on more than 15 days monthly during the 2 months before screening; (3) had headaches suspected or determined to be of organic cause or exacerbated by an organic cause; (4) had uncontrolled hypertension (diastolic blood pressure ≥95 mmHg or systolic blood pressure ≥160 mmHg); (5) had confirmed or suspected cardiovascular or cerebrovascular disease; or (6) had basilar or hemiplegic migraine. Additional exclusion criteria were (7) pregnancy or not practicing adequate contraception (in females and (8) the use of a monoamine oxidase inhibitor within 2 weeks of study entry.
**Kellstein 2000**	Males and females (≥12 years old) must have had at least a 1-year history of migraine with or without aura as defined by International Headache Society (IHS) criteria. Subjects estimated that their average migraine headaches, if left untreated, were at least moderate in pain intensity, and occurred at an average frequency ranging from one every 2 months to eight per month during the previous year. Onset of these headaches had to be before age 50, and subjects must have previously experienced some relief from over-the-counter (OTC) analgesics.	Subjects were ineligible if they had: a head or neck injury or a change in headache pattern during the previous 6 months; a history during the last year of chronic daily use (>30 days) of analgesics, NSAIDs, sedatives/hypnotics, tranquilizers, or a history of drug or alcohol abuse; hypersensitivity or other contraindications to aspirin, ibuprofen or any other NSAID; migraine attacks which were accompanied by vomiting 20% of the time or more; migraines that were usually severely disabling or incapacitating; or atypical migraine with aura subtypes, cluster headache, or serious causes of headache other than migraine.

**Klapper 2000**	Migraine with or without aura (IHS criteria).	Not reported.
**Klapper 2004**	Aged 18 to 65 years; an established diagnosis of migraine as define by International Headache Society (IHS)-criteria with an age of onset <50 years; ability to recognize a migraine headache while intensity is mild; migraine attacks that are initially mild but progress to become moderate or severe; experienced at least 1 migraine headache per month in the last 3 months; ability to differentiate between migraine and non-migraine headache; moderate or severe migraine-associated disability as assessed using the Migraine Disability Assessment Scale (MIDAS) questionnaire.	History of basilar, ophthalmoplegic or hemiplegic migraine headache; frequent nonmigraine headaches; history of, or symptoms suggestive of, ischaemic heart disease or other vascular disease including Prinzmetal's angina, Wolff–Parkinson–White syndrome or other cardiac accessory conduction pathways or arrhythmias; systolic BP ≥150 mmHg or diastolic BP ≥95 mmHg; use of monoamine oxidase type A inhibitors, methysergide or methylergonovine; breast-feeding or pregnancy; hypersensitivity to zolmitriptan or commonly used pharmaceutical excipients; severe hepatic impairment; participation in another clinical trial.
**Klassen 1997**	Males and non-pregnant females using adequate contraception were eligible if they were between 18 and 65 years of age (inclusive), were able to give informed consent. had a history of migraine with or without aura (as defined by the 1988 International Headache Society criteria) for at least 12 months prior to the study, had 1 to 6 attacks per month of moderate to severe migraine in each of the two months (60 days) prior to screening, and were able to distinguish migraine headaches from other headache types.	Subjects were excluded if they had uncontrolled hypertension (sitting diastolic blood pressure ≥95 mmHg or systolic blood pressure ≥160 mmHg) at screening; a history of epilepsy; a history of Raynaud's syndrome; confirmed or suspected cardiovascular or cerebrovascular disease. impaired hepatic or renal function; basilar or hemiplegic migraine; or tension-type headache ≥ 10 episodes or 15 days/month in either of the two months (60 days) before screening. Other exclusion criteria included known hypersensitivity to the drug and use of ergotamine-containing drugs within 24 hours before or after treatment with study medication. Pre-study experience with triptan therapy was not an exclusion criterion.
**Kolodny 2004**	Men and women in good health aged ≥ 18 years with at least a 6-month history of migraine with or without aura, as defined by the International Headache Society diagnostic criteria for migraine.	Patients were excluded from study participation if they used monoamine oxidase inhibitors, methysergide, or propranolol; however, standard antimigraine prophylactic medications (with the exception of non-steroidal anti-inflammatory drugs, daily analgesics, or propranolol) were permitted. Women who were pregnant or nursing were not eligible for the study. Patients also were excluded if they had participated in the previous comparison study.
**Kramer 1998**	Patient had at least a 6-month history of migraine, with or without aura. Patient was male, or if female must have been postmenopausal, surgically sterilized, or taking adequate contraceptive precautions. Patient was judged to be in good health, apart from migraine.	Patient was pregnant or a nursing mother. Patient had abused drugs or alcohol within 12 months prior to entering the study. Patient had a history of cardiovascular disease. Patient had clinically significant electrocardiogram (ECG) abnormality. Patient had a resting systolic blood pressure of greater than 145 mm Hg or diastolic of less than 95 mmHg at screening. Patient received treatment with an investigational device or compound within 30 days of the study start. Patient typically suffered from less than 1 or more than 8 attacks of migraine per month. Patient had difficulty in distinguishing his/her migraine attacks from tension or interval headaches. Patient had prior exposure to rizatriptan.
**Kuca 2018**	Able and willing to give written informed consent. Participants with migraine with or without aura fulfilling the International Headache Society (IHS) diagnostic criteria 1.1 and 1.2.1 (International Headache Classification (ICHD) 2004). History of disabling migraine for at least 1 year. Migraine Disability Assessment (MIDAS) score ≥11. Migraine onset before the age of 50 years.	Pregnant or breast-feeding women. Women of child-bearing potential not using or not willing to use highly effective contraception. Known coronary artery disease, clinically significant arrhythmia or uncontrolled hypertension. History or evidence of hemorrhagic stroke, epilepsy or any other condition placing the participant at increased risk of seizures. History of recurrent dizziness and/or vertigo including benign paroxysmal positional vertigo (BPPV), Ménière's disease, vestibular migraine, and other vestibular disorders. History of diabetes mellitus with

	History of 3 - 8 migraine attacks per month (<15 headache days per month). Male or female, aged 18 years or above. Females of child-bearing potential must be using or willing to use a highly effective form of contraception (e.g. combined oral contraceptive, intrauterine device (IUD), abstinence or vasectomized partner). Able and willing to complete an electronic diary.	complications (diabetic retinopathy, nephropathy or neuropathy). History within the previous three years or current evidence of abuse of any drug, prescription or illicit, or alcohol. History of orthostatic hypotension with syncope. Significant renal or hepatic impairment. Participant is at imminent risk of suicide (positive response to question 4 or 5 on the C-SSRS) or had a suicide attempt within six months prior to the screening visit. Known Hepatitis B or C or human immunodeficiency virus (HIV) infection. History, within past 12 months, of chronic migraine or other forms of primary or secondary chronic headache disorder (e.g. hemicranias continua, medication overuse headache) where headache frequency is greater than 15 headache days per month. Use of more than 3 doses per month of either opiates or barbiturates. Initiation of or a change in concomitant medication to reduce the frequency of migraine episodes within three (3) months prior to Screening/Visit 1.
**Kudrow 2005**	Patients ranged in age from 18 to 65 years (inclusive) and had been clinically diagnosed with migraine according to International Headache Society (IHS) criteria, with or without aura, at least 1 year prior to screening. Additional entry criteria included onset of migraine prior to age 50 and a history of 2 to 8 migraine attacks per month (with at least two moderate or severe migraine attacks per month) for at least 3 months prior to entering the study. Patients were also required to be able to distinguish between migraine and other types of headache and to have a history of migraine headache responding to symptomatic pharmacotherapy (eg, nonspecific NSAIDs, ergotamine, and triptans).	Patients with the following conditions were excluded from entry into the trial: history of migraine variants, Raynaud phenomenon associated with migraine, recent head or neck trauma (within 6 months), or neurologic disorder other than migraine. Patients with history, symptoms, or signs of cardiovascular or peptic ulcer disease were also excluded. Any patient who initiated treatment with migraine prophylactic medication less than 2 weeks prior to the baseline visit or who reported chronic use (>3 consecutive days per week) of any of the following medications was not permitted to enter the trial: analgesics, aspirin, COX-2 specific inhibitors, or nonspecific NSAIDs. Aspirin ≤325 mg per day for cardiovascular prophylaxis was allowed. Also excluded were patients who had used ergotamine-containing or ergot-type medication, 5-hydroxytryptamine $(5\text{-HT})_{1D}$ or 5-HT_{1B}/medication, or COX-2 specific inhibitors within 48 hours of receiving study medication and those who had not previously used sumatriptan.
**Landy 2004**	Non-pregnant female subjects using adequate contraception were eligible for this study if they were 18 to 65 years of age (inclusive), were able to give informed consent, had at least a 1- year history of migraine, with or without aura, as defined by 1988 International Headache Society criteria 1.1 and 1.2 and demonstrated a 6-month history of regularly occurring menstrually-associated migraine (MAM). MAM was defined as any migraine beginning on Day -2, -1, 1, 2, 3, or 4, with Day 1 = the first day of flow. Sites were instructed to enroll only subjects who reported MAM in at least 2 of their last 3 perimenstrual periods prior to screening, who could reliably predict the onset of menstrual flow and who typically experienced moderate to severe MAM pain preceded by a mild pain phase.	Subjects were excluded if they experienced >6 migraine attacks per month in either of the 2 months prior to screening; uncontrolled hypertension (sitting diastolic blood pressure =95mm Hg or systolic blood pressure =160mm Hg) at screening; a history of epilepsy or structural brain lesions which lowered the convulsive threshold; confirmed or suspected cardiovascular, cerebrovascular, peripheral vascular, or ischemic bowel disease; impaired hepatic or renal function; ophthalmoplegic, basilar, or hemiplegic migraine; or tension-type headache on 15 or more days per month in any of the 2 months before screening. Other exclusion criteria included known hypersensitivity to the drug, the use of migraine prophylactic medication containing ergotamine, an ergot derivative, or methysergide; use of a monoamine oxidase inhibitor within 2 weeks before the study; and, in countries where the combination of a selective serotonin reuptake inhibitor and a triptan is not allowed, the need for a selective serotonin receptor inhibitor.
**Lange 2000**	Eligible patients had to be ambulatory male or female patients (18 to 65 years) requiring oral pain treatment for an acute migraine attack according to the classification of the International Headache Society. Prior to enrolment the patients gave their informed consent in writing. In order to be included into the study, patients had to suffer from migraine for more than 1 year with a minimum of one attack per month, but no more than six attacks per month.	Patients meeting at least one of the following criteria were not included in the study: hypersensitivity to salicylates, peptic ulceration or gastric bleeding, impairment of renal, hepatic, endocrine, pulmonary, cardiac, neurologic or cerebral function, vomiting more than 20% of the time during migraine attacks, usually so incapacitated as to require bed rest during the attack, drug or alcohol abuse, pregnancy or lactation, intake of analgesic or anti-migraine drugs within 24 h prior to the intake of the study medication, participation in a clinical study within the previous month or previous enrolment into this study.

**Lee 2001**	International Classification of Headache Disorders (ICHD) criteria, male and female between 18 to 65, under 50 years at onset, able to differentiate tension type headache from migraine	No plans regarding pregnancy, or current pregnancy, or nursing mothers. No drug or alcohol abuse within the last year, monoamine oxidase (MAO) inhibitors, methysergid or methylergonovin within 2 weeks before randomization, heart or peripheral vascular disease or cerebrovascular disease. Patients with cerebral palsy, basilar oftalmoplegic or hemiplegic migraine. hypertensive patients with uncontrolled blood pressure, acquired immunodeficiency, or sexually transmitted disorders and hepatitis etc. Abnormal findings at clinicial examination or laboratory investigations.
**Lines 2001**	Male or non-pregnant female 18 to 65 years old, with at least a 6-month history of migraine according to International Headache Society (IHS) criteria, with 1-8 migraines per month.	Basilar or hemiplegic migraine, significant medical illness (including cardiovascular, heart, kidney, liver, neurological, endocrine, gastrointestinal, hypertension), electrocardiogram (ECG)-abnormality, resting blood pressure of greater than 145 mm Hg or diastolic of less than 95 mmHg at screening, had received treatment with an investigational device or compound within 30 days of the study, typically suffered from 1-8 attacks of migraine per month, had difficulty in distinguishing his/her migraine attacks from tension or interval headaches, was currently taking monoamine oxidase inhibitors, methsergide or lithium, pregnancy or nursing, alcohol abuse.
**Lipton 2000a**	International Classification of Headache Disorders (ICHD)-criteria for migraine without aura/migraine with aura at least 18 years old, good general healthy, at least 1 episode every 2 months. Headaches had to be of at least moderate pain intensity when left untreated.	Not more than 6 headache episodes per month. Subjects who experienced severely incapacitating migraines requiring bed rest or precluding daily activities more than 50% of the time were excluded. Subjects who experienced vomiting with more than 20% of their migraine attacks also were excluded, because of the probability that they would vomit and, therefore, not absorb the study medication. Subjects reporting nausea without vomiting or vomiting less than 20% of the time were not excluded.
**Lipton 2000b**	Adults 18 to 65 years of age who met International Headache Society (IHS) criteria for migraine with (1.2) or without aura (1.1) were eligible for study participation. Patients completed written informed consent prior to study enrollment. Patients were required to have had a 1-year history of migraine with onset before the age of 50 years and to have experienced at least one migraine, but no more than 10 migraine attacks per month in the 6 months prior to study enrollment. This study also included patients with clinical diagnoses of migrainous headache and episodic tension-type headache (ETTH). Only patients who were disabled by their migraines, as defined by an Headache Impact Questionnaire (HIQ) score of 250 or greater at screening, were eligible for study enrollment.	Patients were excluded from the study if they had a history of confirmed or suspected ischemic heart disease; uncontrolled hypertension; organic heart or central nervous system disease; seizure disorder requiring anticonvulsant medication; headache as a result of traumatic head or neck injury; impaired hepatic or renal function; history of migraine variants; evidence of alcohol, drug, or substance abuse within the previous year; or current monoamine oxidase inhibitor (MAOI) therapy. Women were excluded if they were pregnant, breast-feeding, or of childbearing potential and not using adequate contraceptive measures.
**Lipton 2005**	18 to 50 years of age, migraine with or without aura (International Classification of Headache Disorders [ICHD] defined), at least moderate pain, at least one, but not more than six, migraine per month for the previous year.	Vomiting at least 20% of the time, subjects started on preventive treatment during prior 3 months, subjects taking prescription drugs for anti-coagulation, gout, or arthritis, or alkaloids to treat migraine, nonresponsive to over-the-counter (OTC) analgesics or prescribed medications for migraine. Headache caused or aggravated by recent trauma, other head or neck disease, or severe emotional disorders. No allergies or contraindications to aspirin, including ulcer disease, gastrointestinal bleeding, bleeding, or coagulation abnormality, asthma, inflammatory bowel or pancreatic disease, serious cardiac, renal, hepatic, metabolic, or neurologic disease, diabetes, uncontrolled hypertension, or active malignancy. Females

		were required to be practicing effective birth control methods and could not be pregnant or nursing.
**Lipton 2010**	An established one-year history of migraine with or without aura according to the diagnostic criteria of the International Classification of Headache Disorders second edition (ICHD-II). Male and female subjects between the ages of 18 and 65 participated in the trial and had an average of one to six migraine attacks per month for the previous 12 months. To qualify the subject must have had a history. on average of at least one migraine attack per month. but an average of no more than six migraine attacks monthly during the previous year. Subjects had to have 10 or fewer headache days per month.	Pregnancy; risk of pregnancy (not on birth control); lactating; hypersensitivity or allergy to NSAIDs, prostaglandin-synthase inhibitors, aspirin, diclofenac or excipients of study medication products; headache symptoms likely due to, or aggravated by, traumatic injury to the head or neck region, such as whiplash, within the last six months; secondary headache or abnormal findings with neurological exam; or any clinically significant medical history. These criteria were aimed to exclude individuals so disabled that they would be unable to complete the tasks required for study participation (e.g. recording diary card information; use of rescue medications). Subjects with a history of vomiting 20% of the time during migraine attacks or were usually so incapacitated as to require bed rest during the attack were also excluded. These criteria were designed to exclude individuals who were potentially unable to perform or complete the study protocol or were likely to vomit after taking this liquid medication.
**Lipton 2019a**	Subjects had to be able to read, speak, and understand English proficiently; provide written informed consent; and be male or female, 18 to 75 years of age, inclusive, at screening. Females had to have a negative serum pregnancy test at screening, not plan to become pregnant during the study, and not be lactating, and they had to have a negative urine pregnancy test at all subsequent study visits after the screening visit and, unless surgically or otherwise sterile or postmenopausal for more than 1 year, agree to practice a reliable form of contraception or abstinence during the study (eg, implants, injectables, combined oral contraceptives, an intrauterine device, a bilateral tubal ligation, a vasectomized partner, an exclusively female partner, and double-barrier methods); males had to agree to practice a reliable form of contraception or abstinence. Subjects also had to have at least a 12-month history of episodic migraine characterized by 2 to 8 attacks per month, with no more than 14 monthly headache days and at least 48 hours of headache-free time between attacks, an age of onset before age 50 years, and a usual untreated migraine pain intensity of moderate or severe (i.e., 2 or 3 on a scale of 0 to 3). For the duration of the study, subjects had to be able to evaluate and record pain, migraine symptoms, and study drug effectiveness information, as well as each instance of the use of study drug and rescue medication, in real-time using an electronic diary and comply with all other study procedures and scheduling requirements.	Prior exposure to DFN-15; had taken opioids, opioid-barbiturate fixed combinations, triptans, or ergot alkaloids on at least 10 days or NSAIDs or other simple medications on more than 14 days per month during the 90 days before screening; had been treated with onabotulinumtoxinA for migraine within 4 months before screening; were on unstable dosages of migraine preventive medications within 30 days before and through screening; had taken mini-prophylaxis for menstrual migraine; or were on chronic warfarin sodium or equivalent. Subjects were also excluded if they had a history of any condition that might interfere in any way with the study conduct, outcomes, or interpretation of results.

**Lipton 2019b**	Age 18 to 75 years. At least a 1-year history of migraine with or without aura consistent with a diagnosis according to the International Classification of Headache Disorders, 3rd edition, beta version. Migraine onset before age 50. History of migraines typically lasting between 4 and 72 hours if untreated or treated unsuccessfully and migraine episodes are separated by at least 48 hours of headache pain freedom. History of 2 to 8 migraine attacks per month with moderate to severe headache pain in each of the previous 3 months.	Difficulty distinguishing migraine headache from tension-type other headaches. Has taken medication for acute treatment of headache (including acetaminophen, nonsteroidal anti-inflammatory drugs [NSAIDs], triptans, ergotamine, opioids, or combination analgesics) on 10 or more days per month in the previous 3 months. Has a history of migraine aura with diplopia or impairment of level of consciousness, hemiplegic migraine, or retinal migraine. Has a current diagnosis of new persistent daily headache, trigeminal autonomic cephalgia (eg, cluster headache), or painful cranial neuropathy. Required hospital treatment of a migraine attack 3 or more times in the previous 6 months. Has a chronic non-headache pain condition requiring daily pain medication. Has a history of malignancy in the prior 5 years, except for adequately treated basal cell or squamous cell skin cancer, or in situ cervical cancer. Has a history of any prior gastrointestinal conditions (eg, diarrhoea syndromes, inflammatory bowel disease) that may affect the absorption or metabolism of investigational product; participants with prior gastric bariatric interventions which have been reversed are not excluded. Has a history of hepatitis within previous 6 months.
**Lipton 2019c**	Men and women 18 years of age or older were recruited by referral from physicians and other health care professionals and by standard methods of recruitment, including enrollment from clinical practices and through advertising. Treatment settings included clinics, institutions, and private office practices. Eligible participants had migraine, with or without aura, that met the criteria specified in the International Classification of Headache Disorders, 3rd edition (beta version); had a 1-year history of migraine, with an onset before the age of 50 years; had two to eight migraine attacks of moderate or severe intensity per month; and had any headache on fewer than 15 days per month during the previous 3 months. Persons who were receiving preventive migraine medication had to be receiving a stable dose for at least 3 months before trial entry.	History of any clinically significant or unstable medical condition, including alcohol or drug abuse and substance-use disorder, that would expose patients to an undue risk of an adverse event or that could interfere with assessments of safety or efficacy. Patients were also excluded if they had received nonbiologic investigational agents within 30 days before the baseline visit or if they had received biologic investigational agents within 90 days before the baseline visit.
**Lipton 2021**	Patients were required to be previously diagnosed with episodic migraine, with or without aura, as defined by International Classification of Headache Disorders, 3rd edition (ICHD-3), and to have had the diagnosis for ≥1 year. Additional inclusion criteria were as follows: male or female, aged 18 to 75 years; migraine onset prior to 50 years of age; history of episodic migraine (ICHD-3) with 2 to 8 monthly attacks (with or without aura), ≤14 headache days per month, and ≥48 hours of headache-free time between attacks; reported usual migraine pain (without treatment) of 2 (moderate) or 3 (severe) on headache pain severity scale (range 0–3); able to differentiate between migraine and a tension-type or cluster headache; if female and of childbearing potential, a negative pregnancy test was required at all study visits; female patients were required not to be lactating, not to be planning to become pregnant, and to practice reliable birth control or abstinence throughout the study. Participants had to read, speak, and understand English proficiently, to provide written, informed consent, and to be willing	Prior exposure to celecoxib oral solution; intolerance to any celecoxib or sulfonamide formulation, or significant AEs or contraindications related to other NSAIDs (eg, due to gastrointestinal bleed, ulcer, or history of acute renal failure); treatment with an investigational drug or device within 30 days of randomization, or participation in a central nervous system clinical trial within 2 months of randomization. Patients were also excluded if they had experienced medication overuse in the 90 days before screening, defined as opioids or combination medications, including barbiturates, used ≥10 days; NSAIDs or other simple medications (such as over the counter medications) >14 days per month; and triptans or ergots ≥10 days per month. Alcohol or substance use disorder identified within 12 months of screening, a positive urine drug screen for recreational drugs or alcohol, or prescription drug use not explained by disclosed concomitant medication use also excluded patients from participation. Patients with a history of the following drug treatments were also excluded: onabotulinum toxin A for migraine in the 4 months before screening (cosmetic use was acceptable); unstable dosage of migraine prophylactic medication use in the 30 days before and/or throughout screening; use of mini-prophylaxis for menstrual migraine; history of cluster headaches, or only a "probable migraine" diagnosis (per ICHD-3); history of cerebrovascular events, including but not limited to stroke or transient ischemic attack;

	and, in the opinion of the investigator, able to comply with study procedures and scheduling.	chronic use of warfarin sodium or an equivalent drug; current antipsychotic use, or use within 30 days of randomization; treatment with cytochrome P450 (CYP)2C9 inducers, or with CYP2D6 substrates with a narrow therapeutic window (ie, thioridazine), within 7 days of randomization. Patients with the following medical conditions were excluded: concurrent seizure disorder or history of migralepsy (seizure following migraine); history of ischemic coronary artery disease or congenital heart disease; insulin-requiring diabetes mellitus or diabetes with A1C >7.9%; positive screening test for human deficiency virus, hepatitis B surface antigen, or hepatitis C virus antibody; history of cancer within the past 5 years, except adequately treated basal cell or squamous cell skin carcinoma, or in situ cervical cancer; any medical condition or procedure that, in the judgment of the investigator, would have confounded the study objectives. Last, patients with the following vital signs or laboratory values were excluded: uncontrolled hypertension or screening systolic/ diastolic blood pressure (SBP/DBP) >140/90 mm hg; Fridericia's corrected QT interval >450 msec, serum creatinine >1.5x upper limit of normal (ULN), and/or serum total bilirubin >1.5x ULN; serum aspartate aminotransferase (AST), alanine aminotransferase (ALT), or alkaline phosphate >2.5x ULN; or, any clinical laboratory or electrocardiogram (ECG) abnormality that could endanger the participant or interfere with study conduct.
**Loder 2005**	Patients between 18 years and 65 years of age with an established diagnosis of migraine headache (onset age <50 years), as defined by International Headache Society (IHS) criteria for migraine, were eligible to participate in this study. Patients had to have had at least 2 migraine attacks per month, with or without aura, and non-migraine headache symptoms on fewer than 10 days of each month for 3 months prior to study entry. To ensure that the efficacy data would reflect the effect of the study medication, patients also had to be able to distinguish non-migraine from migraine headache. During the trial, women of childbearing potential were required to use a reliable method of birth control.	Patients with a history or symptoms suggestive of ischemic heart disease or other vascular diseases, clinically significant electrocardiographic abnormalities, or uncontrolled hypertension (systolic blood pressure ≥160 mmHg or diastolic blood pressure ≥90 mm Hg) were excluded from the study. Patients with a history of basilar, ophthalmoplegic, or hemiplegic migraine or a serious neurological condition associated with headaches were also excluded. Patients were not eligible if they were receiving concomitant treatment with propranolol or cimetidine or had used a monoamine oxidase inhibitor within 2 weeks of randomization. Patients who initiated therapy with a selective serotonin reuptake inhibitor without a stabilized dose within 2 weeks of randomization were also excluded. Other exclusion criteria included prior serious adverse events or hypersensitivity to any 5-hydroxytryptamine $(5\text{-HT})_{1B/1D}$ agonist, prior history of alcohol or drug abuse, pregnancy or lactation, severe renal or hepatic impairment, history of phenylketonuria, or use of any experimental medication within 30 days of the study.
**MacGregor 2002**	18 years of age or older; had a diagnosis of migraine with or without aura according to International Headache Society diagnostic criteria; had experienced one to six migraine attacks monthly within the previous 3 months, with at least 48 hours of freedom from headache between attacks; had a history of migraine extending more than 1 year; and were otherwise fit and healthy, with no known contraindications to use of aspirin.	Vomiting during the majority of migraine attacks (thus prohibiting the use of oral medication) or regular use of nonsteroidal anti-inflammatory drugs (NSAIDs) or other drugs that might interact with the trial medication.
**Mannix 2007a**	Women aged ≥18 years who had both a ≥6-month history of International Classification of Headache Disorders (ICHD)-II migraine and a ≥6-month history of menstrual migraine (MM) as proposed in the 2004 update of ICHD-II guidelines were eligible for the studies. Additionally, a history of monthly menses and a recent history of MM occurrence in at least two of the most recent three menstrual periods were required. If MM typically preceded	Patients with ischaemic heart disease, uncontrolled hypertension, coronary artery vasospasm (including Prinzmetal's variant angina) or other significant underlying cardiovascular disease were excluded. Patients agreed to discontinue using monoamine oxidase inhibitors and propranolol 2 weeks before receiving study medication; any 5-hydroxytryptamine $(5\text{-HT})_{1B/1D}$ agonist, ergot-type medication (e.g. methysergide, dihydroergotamine), opiates or barbiturates 24 h before receiving study medication; and non-opiate analgesics and anti-emetics 6 h before receiving study medication. Patients using agents for perimenstrual

	menstrual flow, the patient attested to her ability to predict the onset of menstrual flow within 1 day. Patients had to agree to use adequate contraception during the study. Patients with other headache disorders were required to be able to distinguish clearly migraine attacks from other headaches.	migraine prophylaxis were excluded. In addition, daily analgesics taken for any reason were not permitted (except for aspirin ≤325 mg/day for cardioprotection). Patients were not eligible to participate in both studies.
**Mannix 2007b**	Women aged ≥18 years who had both a ≥6-month history of International Classification of Headache Disorders (ICHD)-II migraine and a ≥6-month history of menstrual migraine (MM) as proposed in the 2004 update of ICHD-II guidelines were eligible for the studies. Additionally, a history of monthly menses and a recent history of MM occurrence in at least two of the most recent three menstrual periods were required. If MM typically preceded menstrual flow, the patient attested to her ability to predict the onset of menstrual flow within 1 day. Patients had to agree to use adequate contraception during the study. Patients with other headache disorders were required to be able to distinguish clearly migraine attacks from other headaches.	Patients with ischaemic heart disease, uncontrolled hypertension, coronary artery vasospasm (including Prinzmetal's variant angina) or other significant underlying cardiovascular disease were excluded. Patients agreed to discontinue using monoamine oxidase inhibitors and propranolol 2 weeks before receiving study medication; any 5-hydroxytryptamine $(5\text{-HT})_{1B/1D}$ agonist, ergot-type medication (e.g. methysergide, dihydroergotamine), opiates or barbiturates 24 h before receiving study medication; and non-opiate analgesics and anti-emetics 6 h before receiving study medication. Patients using agents for perimenstrual migraine prophylaxis were excluded. In addition, daily analgesics taken for any reason were not permitted (except for aspirin ≤325 mg/day for cardioprotection). Patients were not eligible to participate in both studies.
**Marcus 2014**	The study included male and female subjects, 18 to 65 years of age, who had at least a one-year history of migraine with or without aura. In addition, the migraine should have started prior to 50 years of age, with an average duration of about four to 72 hours if untreated. Patients also were required to have between two and seven attacks of moderate to severe intensity in each of the three months prior to the screening visit. Patients were to have less than 15 days with headache per month in each of the three months prior to the screening visit and during the screening period, and had to be able to distinguish migraine attacks from other headache types, such as tension-type. Preventive migraine medication was permitted while on study therapy provided the dose was a stable dose for at least three months prior to study entry. Serotonin norepinephrine reuptake inhibitors, selective serotonin reuptake inhibitors, and monoamine oxidase inhibitors required a 14-day wash-out period prior to study entry.	In view of the sumatriptan arm, patients with a history of basilar-type migraine or hemiplegic migraine were excluded from the study, as were patients who did not receive migraine relief from triptans. Patients were excluded if they had a history or evidence of stroke/ transient ischemic attacks, ischemic heart disease, coronary artery vasospasm, other significant underlying cardiovascular diseases, uncontrolled hypertension (high blood pressure), uncontrolled diabetes, or human immunodeficiency virus (HIV) disease. Patients with a current diagnosis of major depression, other pain syndromes, psychiatric conditions (e.g. schizophrenia), dementia, or significant neurological disorders, other than migraine, that in the investigators' opinion might interfere with study assessments, were excluded. Patients with a history of, treatment for or evidence of alcohol or drug abuse within the past 12 months or patients who have met Diagnostic and Statistical Manual of Mental Disorders, fourth edition text revision (DSM-IV-TR) criteria for any significant substance use disorder within the past 12 months from the date of the screening visit were excluded. Concomitant drugs metabolized by CYP3A with narrow therapeutic margin with theoretical potential for drug interaction were not permitted in this study, as potent CYP3A inhibitors and inducers could cause changes in the pharmacokinetics of BMS-927711. Medications that may alter the pH of the stomach, such as H2-receptor antagonists, proton pump inhibitors, and antacids, were prohibited. The use of barbiturates, opioids, triptans, ergotamines, and muscle relaxants were prohibited two days prior to randomization and during the course of this study. Women of child-bearing potential who were unwilling or unable to use an acceptable contraceptive method or abstinence to avoid pregnancy for the entire study period and for up to eight weeks after the study were not permitted to enroll, as were women who were pregnant or were breastfeeding. Women with a positive pregnancy test on enrollment or prior to study drug administration were excluded.

**Massiou 2005**	A menstrually-related migraine (MRM) was defined as a migraine headache occurring in a 6-day window between -2 and +4 days from the start of menses (D1) and preceded by 24 pain-free hours. The subjects were female outpatients aged 18 or over but not more than 65 years at inclusion with a minimum 1-year history of migraine, with or without aura, diagnosed according to the IHS criteria (Headache Classification Committee of the International Headache Society, 1988) (categories 1.1 or 1.2). The inclusion criteria required the women to report regular MRM headaches in at least two of the last three menstrual cycles preceding inclusion, that started with mild pain (score 1) and progressing to moderate or severe pain (scores 2 and 3). Patients were also required to be able to distinguish migraine from other types of headache, such as tension headache, and to be able to predict the start of menses within 1 day for patients with MRM headaches starting at day) 2, day)1 or day 1, to understand the information provided and to be able to fill in a questionnaire and complete the diary record. A negative pregnancy test was required and those women who might become pregnant during the study had to practice effective contraception with the dose of any oral contraceptives being stable for the 2 months before inclusion.	Confirmed or suspected cardiovascular pathologies: ischemic heart disease (angina pectoris, history of myocardial infarction, documented silent ischemia), Prinzmetal's angina, cardiac arrhythmia requiring medication or a significant electrocardiogram abnormality, peripheral vascular disease or Raynaud's syndrome, history of cerebrovascular pathology including stroke, congenital heart disease or uncontrolled arterial hypertension. Also excluded were patients with migraine headaches occurring more frequently than six episodes in the 2 months preceding the trial, or with tension headaches for at least 15 days/month or with atypical forms of migraine or with substance abuse (without any specific recommendation for triptan use). Other exclusion criteria included antecedents of epilepsy, impaired renal or liver function, alcohol abuse, known contra-indications to naratriptan or other 5-hydroxytryptamine (5HT)1 receptor agonists, participation in another clinical trial in the 4 weeks preceding the study or during the 3-month study duration.
**Mathew 1997**	Males and nonpregnant females using adequate contraception were eligible if they were between 18 and 65 years of age (inclusive), and could understand and complete the diary card, had at least a 1-year history of migraine with or without aura as defined by the 1988 International Headache Society criteria, had 1 to 6 migraines monthly during the 2 months (60 days) preceding the screening visit, and had a history of moderate to severe pain during migraine attacks, and were able to distinguish migraine attacks from other headaches e.g. tension headaches.	Uncontrolled hypertension (sitting diastolic blood pressure ≥95 mmHg or systolic blood pressure ≥160 mmHg) at screening; a history of epilepsy; confirmed or suspected cardiovascular, cerebrovascular, or peripheral vascular disease; cardiac arrhythmias requiring medication, atherosclerotic disease, or congenital heart disease; impaired hepatic or renal function; basilar or hemiplegic migraine; or tension-type headache 10 episodes or 15 or more days per month in any of the 2 months (60 days) before screening; history of Raynaud's syndrome. Other exclusion criteria included known hypersensitivity, intolerance, or contraindication to the use of naratriptan or any other 5-hydroxytryptamine (5HT)1 receptor agonist, such as sumatriptan; ergotamine-containing drugs within 24 hours before or after treatment with study medication; evidence of alcohol, drug or substance abuse within the last year, which, in the investigator's judgment, would likely interfere with the study conduct, subject cooperation, or evaluation and interpretation of the study results; opiate or simple analgesics and anti-emetics (or any other medication for migraine) within 6 hours before or 4 hours after treatment with study medication; participation in an investigational drug trial within the previous 4 weeks or plans to participate in another study any time during this study; any severe concurrent medical condition which may have affected the interpretation of efficacy and safety data or which otherwise may have contraindicated participation in a clinical trial with a new chemical entity. Sumatriptan medication within 24 hours before or after treatment with study medication was an exclusion criterion.

**Mathew 2003**	The study sample consisted of men and women, aged 18 to 65 years, who met the International Headache Society (IHS) criteria for migraine with or without aura, and who reported a monthly frequency of one to six attacks.	Patients were excluded for the following reasons: presence of frequent concurrent non-migrainous headache or treatment-resistant migraine (or both) or migraine variants (eg, familial hemiplegic or basilar migraine); coronary artery disease, heart failure, uncontrolled hypertension, or abnormal electrocardiogram (ECG); any clinically significant medical illness or laboratory abnormality; severe reduction in gastrointestinal absorption; hypersensitivity or known contraindication to treatment with eletriptan or sumatriptan; concomitant use of potent CYP3A4 inhibitors or use of monoamine oxidase (MAO) inhibitors in the 2 weeks prior to study entry; misuse or abuse of alcohol or other substances including analgesics or ergotamine; use of any experimental drug within the past month; and women who were pregnant or breast-feeding.
**Mathew 2004**	Patients aged 20 to 64 years were eligible for the study if they met the International Headache Society (IHS) criteria for migraine or migraine with aura and had a history of headache progressing to moderate or severe pain when no intervention was used.	Not reported.
**Mathew 2007**	Patients to be 18 to 65 years of age and to have a history of International Headache Society (IHS)-defined migraine of at least moderate pain intensity with or without aura for at least 1 year and an average migraine headache frequency of 2 to 6 each month for the past 3 months. Patients needed to be able to differentiate migraine headache from an interval (eg, tension type) headache. Effective single medication for migraine prophylaxis was allowed if patients were on a maintenance dose for at least 30 days prior to the screening visit and remained on a stable dose for the study duration. Female patients could not be pregnant or lactating and had to be postmenopausal for at least 1 year, surgically sterile, or practicing effective birth control.	Average of at least 15 headache days per month in the previous 6 months, onset of migraine after age 50 years, contraindication to almotriptan or other trip tan or previous discontinuation of almotriptan therapy including Prinzmetal's variant angina) or peripheral vascular disease, uncontrolled hypertension, abnormal renal, and/or hepatic function, or significant mental dis order. Previous use of triptans was not an exclusion criterion due to an AE or lack of efficacy, routine nonmigraine headaches that would confound discrimination from migraine headache, hemiplegic or basilar migraine, mi graine aura without headache, and migraines typically accompanied by vomiting or which occur predominantly upon awakening in the morning. Other exclusion criteria were overuse of opioids, ergotamine-type medications, nonsteroidal anti-inflammatory drugs, cyclooxygenase type II selective inhibitors, aspirin, acetaminophen, benzodiazepine sedative hypnotics, anti-emetics or 5-hydroxytryptamine $(5\text{-HT})_{1B/1D}$ receptor agonists or current, recent or suspected history of alcohol or substance dependence/abuse, significant unstable medical dis ease, including symptomatic coronary artery disease, ischemic heart disease (angina pectoris, history of myocardial infarction), coronary artery vasospasm (including Prinzmetal's variant angina) or peripheral vascular disease, uncontrolled hypertension, abnormal renal, and/or hepatic function, or significant mental disorder. Previous use of triptans was not an exclusion criterion.
**Misra 2010**	Patients aging between 16 to 65 years with established diagnosis of migraine with or without aura were included in the study. International Headache Society Diagnostic Criteria were used to define the intensity of migraine as moderate (pain influencing patient's job performance, but he does not miss work) or severe (pain influencing work, school & social situations and he loses time for activities).	The patients with a history of basilar, ophthalmoplegic or hemiplegic migraine, with organic or structural brain lesion, ischemic heart disease, Prinzmetal's angina, WPW syndrome, cardiac conduction defect or arrhythmias, uncontrolled hypertension, were excluded. Other exclusion criteria were patients currently on prophylactic medication, pregnancy or amenorrhea, severe renal or hepatic disease and severe vomiting requiring parenteral drug administration.

**Mitsikostas 2010**	Male and female migraineurs, 18 to 65 years old, with a current history of migraine with or without aura, were eligible to participate. The diagnosis of migraine was made according to the International Headache Society (IHS) criteria after direct interview. Patients had to have a history of 1 to 10 migraine attacks per month of any severity for the last 2 months prior to enrolment.	Pregnant or lactating women as well as women of childbearing potential who refused to use a valid method of contraception were excluded. Subjects with a history of basilar, hemiplegic or ophthalmoplegic migraine, cardiovascular disease, Raynaud's disease, epilepsy or structural brain lesions, impaired renal or hepatic function were also excluded.
**MOMENTUM 2019**	Male or female, 18 to 65 years of age. Established diagnosis (at least 1 year) of migraine with or without aura as defined by the International Classification of Headache Disorders (ICHD)-3 criteria. An average 2 to 8 moderate to severe migraines per month, on average. History of inadequate response as assessed by a score of ≤ 7 on the mTOQ-4	Cluster headaches or other types of migraines. Chronic daily headache (≥15 non-migraine headache days per month). History of significant cardiovascular disease. Uncontrolled hypertension.
**Moon 2010**	Men and women, aged 18 to 65 years, with a history of migraine with or without aura for at least 12 months, as defined by the International Classification of Headache Disorders-II (ICHD-II) criteria. All patients had experienced one to eight moderate-to-severe migraine episodes per month for at least two consecutive months prior to enrollment.	1) A history of basilar, ophthalmoplegic, or hemiplegic migraine, 2) >15 headache days per month prior to screening, 3) cerebrovascular, cardiac, hepatic, or renal disease, 4) being pregnant or breast-feeding, 5) a history or current evidence of drug or alcohol abuse, 6) a history of exposure to frovatriptan, 7) treatment with any other investigational compound or device within 30 days of the start of this study, and 8) initial onset of migraine after the age of 50 years.
**Munjal 2017**	Patients with a history of episodic migraine (as defined by International Classification of Headache Disorders [ICHD]-2) who experience an average of 2 to 6 migraine attacks a month for the past 12 months with no more than 14 headache days per month, and with at least 48 hours of headache-free time between migraine attacks; 2.Patients with onset of migraine with or without aura before age 50; 3.Patients who have migraine with or without aura, in which the aura cannot last longer than 60 minutes; 4.Patients who report usual migraine pain of 2 (moderate) or 3 (severe) on headache pain severity scale.	1.Patients with medication overuse headache (MOH) as defined by ICHD-2: Opioids ≥ 10 days a month during the 90 days prior to screening, Combination medications (eg, Fiorinal® ≥ 10 days a month), Nonsteroidal anti-inflammatory drugs (NSAIDs) or other simple medications ≥ 14 days a month during the 90 days prior to screening, Triptans or ergots ≥ 10 days a month during the 90 days prior to screening. 2.Patients on chronic warfarin sodium; 3.Patients taking monoamine oxidase-A (MAO-A) inhibitors; 4.Patients on unstable dosages of chronic medications during the 3 months prior to and through screening, or who are not willing or able to maintain a stable pre-study dose throughout study participation; 5.Patients with more than 6 migraine attacks a month and/or more than 14 headache days a month (based upon patient self-report); 6.Patients with hemiplegic migraine or migraine with brain stem aura or other forms of neurologically complicated migraine; 7.Patients with atypical aura; 8.Patients with prolonged aura (more than 1 hour). 9.Patients with a history of stroke or transient ischemic attack; 10.Patients with a history of migralepsy or a concurrent diagnosis of seizure disorder; 11.Patients who cannot differentiate between a migraine headache and a tension-type or cluster headache or any other non-migraine headache; 12.Patients with a history of more than 10 tension-type headaches per month; 13.Patients with a history of cluster headache; 14.Patients with a diagnosis of ICHD-2 "probable migraine"; 15.Patients with uncontrolled hypertension (screening blood pressure ≥140/90 mmHg despite appropriate pharmacotherapy); 16.Patients with severe renal impairment (defined as serum creatinine > 1.9 mg/dL); 17.Patients with serum total bilirubin > 1.9 mg/dL; 18.Patients with serum aspartate aminotransferase (AST), alanine aminotransferase (ALT), or alkaline phosphatase > 3 times the upper limit of normal; 19.Patients with positive serology for human immunodeficiency virus (HIV), Hepatitis B surface antigen, Hepatitis C antibody. 20.Patients with a history of alcohol or substance abuse (including marijuana and medical marijuana) within 1 year that would compromise data collection; 21.Patients with a history of or current

		neurological or psychiatric impairment, or cognitive dysfunction that, in the opinion of the investigator, would compromise data collection; 22. Patients with any other medical condition that, in the judgment of the investigator or medical monitor, would confound the objectives of the study (eg, cancer history [except basal cell carcinoma], systemic lupus erythematosus); 23. Patients who have participated in a clinical trial involving any medication during the past 30 days or 5 half-lives of the study medication, whichever is longer.
**Myllylä 1998**	Patients of both sexes were eligible if aged between 18 and 65 and if they met the diagnostic criteria for migraine with or without aura as defined by the International Headache Society (IHS). Only patients for with a history of migraine more than 1 year and with more than one but less than four attacks per month, characterized by severe or moderate headache, were admitted.	Not reported.
**Nappi 1994**	Adults, otherwise healthy, between the ages of 18 and 65 years were eligible for the study if they met the International Headache Society classification of migraine with or without aura and if they had experienced migraine attacks of moderate or severe intensity for at least 1 year.	Migraine prophylaxis. Pregnant or lactating. Hypertensive (untreated supine diastolic blood pressure > 95 mm Hg). Ischaemic heart disease or any other medical condition that might interfere with interpretation of the study results or if they had a current or recent history of drug or alcohol abuse.
**NCT00471952 2008**	Subject is 18 to 65 years of age. Diagnosis of migraine with or without aura. 1 year history of migraine with 1 to 6 migraine attacks per month in the three months prior to screening. Medication for migraine prevention with a stable dose for at least 1 month prior to screening. Has successfully treated a migraine attack with a triptan medication.	Confirmed or suspected ischemic heart disease. History of congenital heart disease. History of cerebrovascular disease, including stroke. History of ischemic abdominal disease. Uncontrolled hypertension. History of epilepsy. History of basilar or hemiplegic migraine. Impaired hepatic or renal function. Greater than 15 headache days per month. Subjects on an monoamine oxidase inhibitor (MAOI). Subjects taking and ergotamine, or ergot containing preventive medication. Subject is pregnant, trying to become pregnant or breast feeding. Evidence of alcohol or substance abuse in the last year. History of caffeine withdrawal headache. Consumes more than 275 mg of caffeine on daily basis from dietary and medication sources.
**NCT00821483 2008**	1. Adults aged 18 to 65 years. 2. The patients must have a history of migraine according to the criteria of the Headache. Classification Committee of the International Headache Society (IHS), over the previous 1 year. The patient must have experienced one to eight moderate or severe migraine attacks (with or without aura) each month over at least the previous two months. 3. Onset of migraine disease must have occurred before the patients was 50 years of age. 4. Able and willing to sign informed consent, and able and willing to comply with study procedures, including the completion of diary cards.	Pregnant or lactating females, or women intend to become pregnant or breast feed during the study period, or women of childbearing potential not using adequate contraception. Females of reproductive potential must have a negative pregnancy test at screening Clinically significant renal dysfunction (creatinine ≥2.0mg/dl) or hepatic dysfunction (ALT, AST ≥2ULN). Patients with a diagnosis of vertebrobasilar or hemiplegic (prolonged atypical aura) migraine (IHS criteria). Potentially uncooperative patients, those unable to provide informed consent, and those unable. to complete the diary. patients who habitually abuse headache medication including ergotamine-containing compounds, and patients with a history of alcohol and/or medicine abuse, in the Investigator's opinion. Patients who are not able to tell that they are having a migraine headache Patients who have 15 or more headache days per month, on average, or those taking symptomatic medication for headaches on more than two days per week, on average. Treatment with a monoamine-oxidase inhibitor (MAOI) within two weeks of the screening visit. Patients who are taking prophylactic migraine medication, unless dose has been stabilized for 30 days and it expected to continue for the duration of the study. Patients with clinically significant abnormal electrocardiograms (ECGs) or with resting diastolic blood pressure above 95mmHg. Patients with clinically significant cardiovascular or

		cerebrovascular disease. Patients with a history of clinically relevant allergy including allergy to triptan. Previous treatment with Frovatriptan at any time or treatment with an investigational drug within 30 days before screening visit.
**NCT00920686 2010**	Male or female migraineurs between 18 and 65 years old; Subjects must have a headache history of migraine with aura as defined by: Aura consisting of at least one of the following, but no muscle weakness or paralysis. Fully reversible visual symptoms; Fully reversible sensory symptoms. Fully reversible dysphasia; Aura has at least two of the following characteristics: Visual symptoms affecting only one side of the field of vision and/or sensory symptoms affecting only one side of the body. At least one aura symptom that develops gradually over more than 5 minutes and/or different aura symptoms occur in succession over more than 5 minutes. Each symptom lasts from 5 to 60 minutes. Headache begins during the aura or follows aura within 60 minutes. Headache not attributable to another disorder. Migraine pain following aura in at least 75% of occurrences. Headache frequency of at least 1 migraine attack (with or without aura) per month for the past 3 months but not more than 8 migraines in any 30-day period. Subjects must have at least 1 migraine headache with aura per month; Each migraine attack should last at least 4 hours (without treatment) and not longer than 72 hours; At the time of the study migraine, prior to dosing with study medication, the headache severity, as judged and documented by the subject, is either moderate or severe (on a 4-point categorical scale); Body mass index (BMI) range of 18 to 35; The subject is in general good health; ALT cannot be above 1.5x upper limit of normal; creatinine and urea must be within normal limits; Speak, read, and understand English, French, or Spanish sufficiently to understand the nature of the study, to provide written informed consent, and complete all study assessments; The subject is willing and able to comply with all testing requirements defined in the protocol; All females will avoid pregnancy at least 10 days before randomization, and up until 3 months after dosing; All subjects/partners must use a double-barrier method of birth control during the study and for 3 months after dosing.	A diagnosis of headaches that is not consistent with migraine with aura. Presence of any risk factors that would preclude the use of triptans. Known allergy or hypersensitivity to triptans or history of any serious side effect with a triptan which would preclude further dosing with a triptan. Presence of any clinically significant condition that would preclude study participation, as evaluated by the investigator. Pregnancy or lactation. History of significant neurological, hepatic, renal, endocrine, cardiovascular, gastrointestinal, pulmonary, rheumatologic, autoimmune, or metabolic disease. Use of the following if taken for migraine prevention: Cardiovascular drugs (acceptable if reason for use is for treatment of cardiovascular disease and the subject has been on a stable dose for 3 months). Valproate, topiramate, cyproheptadine, montelukast, or botulinum toxin. Use of monoamine oxidase inhibitors within 30 days of randomization. Initiation of therapy with selective serotonin reuptake inhibitors (SSRIs) or serotonin and noradrenaline reuptake inhibitors (SNRIs) for depression or other approved indication within 90 days of randomization (subjects on stable dose for >3 months for treatment of depression or other approved indication may be included). Are known to or suspected to be currently abusing alcohol or drugs, or have a history (within the past 12 months) of active alcohol or drug abuse. Participation in another drug or biologic study within 30 days of randomization into this study or during participation in this study. Subjects who are unable or unwilling, in the opinion of the Investigator, to comply with all study procedures and cooperate fully with study center staff.

**NCT01248468 2011**	1. Male or female aged 18 years and over. 2. International Headache Society (IHS) diagnosis of migraine without aura or typical aura with migraine headache. 3. History of experiencing at least 1, but not more than 8, acute migraine attacks monthly during the previous year. 4. History of at least moderate migraine pain intensity, if left untreated.	1. Headache symptoms which may be due to or aggravated by: Recent (within 6 months) head or neck trauma (e.g., whiplash). Head or neck pain secondary to an orthopedic abnormality. Cluster headache. Specific migraine variants (e.g., basilar-type artery migraine, ophthalmoplegic migraine, hemiplegic migraine, migraine aura without headache). Other serious, non-migraine causes of headache (e.g., increased intracranial pressure, intracranial bleeding, meningitis, malignancy). Non-serious, non-migraine causes of headache (e.g., cold, flu, hangover). 2. Routine use (≥ 10 days per month, on average) of any medication having the potential to interfere with the pharmacologic effects or evaluation of the study medications (e.g., narcotic and non-narcotic analgesic products (prescription or over-the-counter), ergotamine-containing and ergot-type medication, anxiolytics, hypnotics, sedatives, 5-hydroxytryptamine (5HT)-1 agonists, anti-emetics, or prokinetic drugs). 3. History of vomiting during more than 20% of migraine episodes or confined to bedrest for more than 50% of migraine episodes. Other protocol-defined inclusion/exclusion criteria may apply.
**NCT01657370 2012**	At least 12 months of migraine with or without aura, migraines last between 4 to 72 hours if untreated, 2 or more and 8 or less moderate or severe migraines per month in each of the two months prior to screening, male, female not of reproductive potential or female with beta human choriogonadotropin (HCG) level serum not consistent with pregnancy and agreeing to use acceptable contraception.	Pregnant or breast-feeding, or is a female expecting to conceive within the projected duration of study participation. Participant has difficulty distinguishing his/her migraine attacks from tension-type headaches. History of predominantly mild migraine attacks or migraines that usually resolve spontaneously in less than two hours. More than 15 headache-days per month or has taken medication for acute headache on more than 10 days per month in any of the three months prior to screening. Basilar-type or hemiplegic migraine headache. > 50 years old at age of migraine onset. Taking migraine prophylactic medication where the prescribed daily dose has changed during the 3 months prior to screening and during the study. Taking a proton pump inhibitor (PPI) or a histamine receptor 2 (H_2) blocker on a daily or near daily basis (> 3 days per week). Taking the following medications from 1 month prior to screening through study period: potent cytochrome P450 (CYP) 3A4 inhibitors (e.g., cyclosporine, itraconazole, ketoconazole, fluconazole, erythromycin, clarithromycin, nefazodone, telithromycin, cimetidine, quinine, diltiazem, verapamil, modafinil and human immunodeficiency virus [HIV] protease inhibitors), moderate or marked CYP3A4 inducers (e.g., rifampicin, rifabutin, barbiturates [e.g., phenobarbital and primidone], systemic glucocorticoids, nevirapine, efavirenz, pioglitazone, carbamazepine, phenytoin, and St. John's wort), or drugs with narrow therapeutic margins and potential for drug interactions in the CYP2C family (e.g., warfarin). Participant is unable to refrain from consumption of grapefruit or grapefruit juice during study. History of hypersensitivity to, or has experienced a serious adverse event in response to 3 or more classes of drugs (prescription and over-the-counter). Clinical or laboratory evidence of uncontrolled diabetes, HIV disease, or significant pulmonary, renal, hepatic, endocrine, or other systemic disease. Other confounding pain syndromes, psychiatric conditions such as uncontrolled major depression, dementia or significant neurological disorders other than migraine. Patients who are currently being treated with non-prohibited medication for depression and symptoms are well controlled are eligible to participate. Participant is at imminent risk of self-harm. History of malignancy ≤5 years prior to study, except for adequately treated basal cell or squamous cell skin cancer, or in situ cervical cancer. History of gastric or small intestinal surgery (including gastric bypass surgery or banding), or presence of a disease that causes malabsorption. History or current evidence of any condition, therapy, lab abnormality or other circumstance that might confound the results of the study, or interfere with subject's participation for the full duration of the study. Participant has recent history

		(within the last year) of drug or alcohol abuse or dependence or is a user of recreational or illicit drugs. Participant is legally or mentally incapacitated. Donation of blood products or phlebotomy of >300 ml within 8 weeks of study, or intent to donate blood products or receive blood products within 30 days of screening and throughout study. Intent to donate eggs or sperm within the projected duration of the study. Current participation in or participation within 30 days of screening in a study with an investigational compound or device, with the exception of MK-1602 Protocol 006. Previous exposure to MK-0974 and/or MK-3207. Use within the past 2 months of an opioid- or barbiturate-containing analgesic for migraine relief. Inpatient or emergency department treatment of an acute migraine attack within the past 2 months.
**NCT01986270 1998**	Subjects, male/female who expected to suffer at least one acute attack of migraine, with or without aura, each 6 weeks The diagnosis of migraine was to comply with the criteria proposed by the International Headache Society (IHS). Subjects capable of taking medication as outpatients, and recording the effects of such medication.	Migraine subjects who also suffered from concomitant frequent (non-migrainous) headache, defined as more than six attacks per month on average.
**NCT03235479 2018**	1. Patient has at least 1 year history of migraines (with or without aura), consistent with a diagnosis according to the International Classification of Headache Disorder, 3rd Edition, Beta version, including the following: Not more than 8 attacks of moderate or severe intensity per month within last 3 months. Consistent migraine headaches of at least 2 migraine headache attacks of moderate or severe intensity in each of the 3 months prior to the Screening Visit and maintains this requirement during the Screening Period. 2. Less than 15 days with headache (migraine or non-migraine) per month in each of the 3 months prior to the Screening Visit and maintains this requirement during the Screening Period. 3. Patients on prophylactic migraine medication are permitted to remain on therapy provided they have been on a stable dose for at least 3 months prior to study entry. 4. Patients with contraindications for use of triptans may be included provided they meet all other study entry criteria.	1. Patient history of HIV disease. 2. Patient history with current evidence of uncontrolled, unstable or recently diagnosed cardiovascular disease, such as ischemic heart disease, coronary artery vasospasm, and cerebral ischemia. Patients with Myocardial Infarction (MI), Acute Coronary Syndrome (ACS), Percutaneous Coronary Intervention (PCI), cardiac surgery, stroke or transient ischemic attack (TIA) during the 6 months prior to screening. 3. Uncontrolled hypertension (high blood pressure), or uncontrolled diabetes (however patients can be included who have stable hypertension and/or diabetes for 3 months prior to being enrolled). 4. Patient has a current diagnosis of major depression, other pain syndromes, psychiatric conditions (eg, schizophrenia), dementia, or significant neurological disorders (other than migraine) that, in the Investigator's opinion, might interfere with study assessments. 5. Patient has a history of gastric, or small intestinal surgery, or has a disease that causes malabsorption. 6. The patient has a history or current evidence of any significant and/or unstable medical conditions (eg, history of congenital heart disease or arrhythmia, known suspected infection, hepatitis B or C, or cancer) that, in the investigator's opinion, would expose them to undue risk of a significant adverse event (AE) or interfere with assessments of safety or efficacy during the course of the trial. 7. History of, treatment for, or evidence of, alcohol or drug abuse within the past 12 months or patients who have met DSM-V criteria for any significant substance use disorder within the past 12 months from the date of the screening visit.
**NCT04218162 2020**	Able and willing to give written informed consent. Male or female, aged 18 years or above. Participants with migraine with or without aura fulfilling the International Headache Society (IHS) diagnostic criteria 1.1 and 1.2.1 (International Headache Classification (ICHD-3). History of disabling migraine for at least 1 year. Migraine Disability Association (MIDAS) score ≥11. Migraine onset before the age of 50 years. History of 3 - 8 migraine attacks per month (< 15 headache days per month). Females of child-bearing potential must be using or willing to use a highly effective	Any medical condition or clinical laboratory test which in the judgment of the Investigator makes the participant unsuitable for the study. Pregnant or breast-feeding women. Known hypersensitivity to lasmiditan or to any excipient of lasmiditan oral tablets, or any sensitivity to lasmiditan. History or evidence of hemorrhagic stroke, epilepsy or any other condition placing the participant at increased risk of seizures. History of recurrent dizziness and/or vertigo including benign paroxysmal positional vertigo (BPPV), Ménière's disease, vestibular migraine, and other vestibular disorders. History of diabetes mellitus with complications (diabetic retinopathy, nephropathy or neuropathy). History within the previous three years or current evidence of abuse of any drug, prescription or illicit, or alcohol. History of orthostatic

	form of contraception (e.g. combined oral contraceptive, intrauterine device (IUD), abstinence or vasectomized partner). Able and willing to complete an electronic diary to record details of the migraine attack treated with study drug.	hypotension with syncope. Significant renal or hepatic impairment. Participant is at imminent risk of suicide (positive response to question 4 or 5) on the Columbia-Suicide Severity Rating Scale (C-SSRS) or had a suicide attempt within six months prior to screening. Participation in any clinical trial of an experimental drug or device in the previous 30 days. Known Hepatitis B or C or human immunodeficiency virus (HIV) infection. History, within past 12 months, of chronic migraine or other forms of primary or secondary chronic headache disorder (e.g. hemicranias continua, medication overuse headache) where headache frequency is ≥15 headache days per month. Use of more than 3 doses per month of either opiates or barbiturates. Initiation of or a change in concomitant medication to reduce the frequency of migraine episodes within three (3) months prior to Screening/Visit 1.
**NCT04384367 2022**	Patients of both sexes; Aged between 18 and 65 years old; Capable and willing to give free and informed consent in writing; Migraine patients with or without aura, according to the International Headache Society (IHS).	Chronic migraine; Headache other than migraine (that is, tension-type headache, sinusitis, etc.).
**NCT05399459 2022**	Subject has at least 1 year history of migraines (with or without aura), consistent with a diagnosis according to the International Classification of Headache Disorder, 3rd Edition, including the following: 1. Migraine attacks present for more than 1 year with the age of onset prior to 50 years of age. 2. Migraine attacks, on average, lasting about 4-72 hours if untreated. 3. Not more than 8 attacks of moderate to severe intensity per month within the last 3 months. 4. Ability to distinguish migraine attacks from tension/cluster headaches. 5. Consistent migraine headaches of at least 2 migraine headache attacks of moderate or severe intensity in each of the 3 months prior to the Screening Visit and maintains this requirement during the Screening period. 6. Less than 15 days with headache (migraine or non-migraine) per month in each of the 3 months prior to the Screening Visit and maintains this requirement during the Screening Period. 7. Subjects on one prophylactic migraine medication are permitted to remain on therapy provided they have been on a stable dose for at least 3 months prior to screening visit and the dose is not expected to change during the course of the study. 8. Subjects with contraindications for use of triptans may be included provided they meet all other study entry criteria.	1. Subject has a history of basilar migraine or hemiplegic migraine. 2. History of use of analgesics (e.g. nonsteroidal anti-inflammatory drugs [NSAIDs] or acetaminophen) on = 15 days per month during the 3 months (12 weeks) prior to the Screening Visit. 3. Subject with a history of HIV disease 4. Subject history with current evidence of uncontrolled, unstable or recently diagnosed cardiovascular disease, such as ischemic heart disease, coronary artery vasospasm, and cerebral ischemia. subjects with Myocardial Infarction (MI), Acute Coronary Syndrome (ACS), Percutaneous Coronary Intervention (PCI), cardiac surgery, stroke or transient ischemic attack (TIA) during the 6 months prior to screening 5. Uncontrolled hypertension (high blood pressure), or uncontrolled diabetes (however subjects can be included who have stable hypertension and/or diabetes for at least 3 months prior to being enrolled)6. Subject has a current diagnosis of major depression, other pain syndromes, psychiatric conditions (e.g., schizophrenia), dementia, or significant neurological disorders (other than migraine) that, in the Investigator's opinion might interfere with study assessments. 7. Subject has a history of gastric, or small intestinal surgery (including Gastric Bypass, Gastric Banding, Gastric Sleeve, Gastric Balloon, etc.), or has disease that causes malabsorption 8. The subject has a history of current or evidence of any significant and/ or unstable medical conditions (e.g., history of congenital heart disease or arrhythmia, known suspected infection, hepatitis B or C, or cancer) that, in the investigator's opinion, would expose them to undue risk of a significant adverse event (AE) or interfere with assessments of safety or efficacy during the course of the trial. 9. History of, treatment for, or evidence of, alcohol or drug abuse within the past 12 months or subjects who have met DSM-V criteria for any significant substance use disorder within the past 12 months from the date of the screening visit. 10. Participation in any other investigational clinical trial while participating in this clinical trial.

**NCT05509400 2022**	1. Signed Written Informed Consent: a. Written informed consent must be obtained from the subject in accordance with requirements of the study center's institutional review board (IRB) or ethics committee, prior to the initiation of any protocol-required procedures b. Subjects must be able to read and communicate with site staff. 2. Target Population: Minimum 1-year documented history of migraine attacks (with or without aura) consistent with a diagnosis according to the International Classification of Headache Disorders, 3rd Edition per self-report, with confirmation from Investigator / supporting medication record, subjects must have: a. Migraine attacks present for more than 1 year with the age of onset prior to 50 years of age. b. Migraine attacks, on average, lasting about 4 - 72 hours if untreated. c. 4 to 14 migraine days per month on average across the 3 months prior to the Screening Visit (month is defined as 28 days for the purpose of this protocol). d. Subjects must be able to distinguish migraine attacks from tension/cluster headaches. e. Subjects on prophylactic migraine medication (excluding CGRP antagonists) are permitted to remain on therapy if they have been on a stable dose for at least 3 months (12 weeks) prior to the Screening Visit, and if the dose is not expected to change during the course of the study. 3. Triptan unsuitable. 4. Age and Reproductive Status: a. Male and Female subjects 18 - 65 years of age. b. Women of childbearing potential (WOCBP) with non-sterile male partners and non-sterile men with female partners of childbearing potential must use two methods of contraception (with at least 1 highly effective method and 1 additional method) to avoid pregnancy throughout the study in such a manner that the risk of pregnancy is minimized. c. At the Baseline Visit prior to dispensing investigational study drug, WOCBP must have a negative urine pregnancy test (minimum sensitivity 25 IU/L or equivalent units of HCG). 5. Subjects must be able to fully comply with the prohibitions and restrictions on the concomitant use of medications and therapies (including moderate to strong inhibitors and inducers of the CYP3A4 enzyme and strong inhibitors of the P-gp transporter).	1. Target Disease Exclusion, a) History of cluster headaches, basilar migraine (with aura), or hemiplegic migraine. b) Current medication overuse headaches. c) Headaches occurring 15 or more days per month (migraine or non-migraine) in any of the 3 months prior to Screening Visit (SV). 2. Medical History and Current Diseases: a) History of gastric or small intestinal surgery or disease or conditions that causes malabsorption. b) BMI ≥ 35kg/m2. c) Hematologic or solid malignancy diagnosis within 5 years prior SV. d) Alcohol or drug abuse within the past 12 months or subjects with any significant substance use disorder within the 12 months from the SV. e) Current diagnosis schizophrenia, bipolar, or borderline personality disorder. f) History or current evidence of other major psychiatric disorder that might interfere with the ability to properly report clinical outcomes. g) Major depressive (MDD) or any anxiety disorder which requires more than 1 daily medication for each disorder, or major depressive episode within last 12 months. h) Active chronic pain syndrome. i) Other pain syndromes, dementia, or significant neurological disorders (other than migraine). j) Current diagnosis of MDD requiring treatment with atypical antipsychotics. k) History with current evidence of uncontrolled, unstable or recently diagnosed cardiovascular disease during 24 weeks prior to SV. l) Systolic blood pressure >160 mmHg or diastolic blood pressure > 100 mmHg after 10 minutes of rest. m) History or current evidence of any unstable medical conditions. n) Positive for drugs of abuse that in the investigator's judgment is medically significant, in that it would impact the safety of the subject or the interpretation of the study results. 3. Allergies and Adverse Drug Reactions: History of drug or other allergy which, in the opinion of the investigator, makes the subject unsuitable for participation in the study. Rimegepant is contraindicated in subjects with a hypersensitivity to any component of its formulation. 4. Sex and Reproductive Status: a) WOCBP, and non-sterile males, who are unwilling or unable to use required contraception or abstinence to avoid pregnancy for the entire study period and for 60 days (WOCBP) and 90 days (non-sterile males) after the last dose of study drug. b) Women who are pregnant, lactating or breastfeeding. c) Women with a positive pregnancy test at SV or prior to study drug administration. 5. Electrocardiogram (ECG) and Laboratory Test Findings - as specified in the protocol. 6. Prohibited Medications and Devices: a) Non-Narcotic Analgesics taken at least 15 days per month for a non-migraine indication during the 12 weeks prior to SV. b) Other CGRP antagonists (beyond rimegepant), including: i. CGRP antagonist monoclonal antibodies taken within 24 weeks prior SV. ii. CGRP antagonist small molecules taken within 10 days prior SV. c) Botox taken within 12 weeks prior SV. d) Cefaly or any other device for migraine treatment or prevention used within 12 weeks prior SV. e) Ergotamine taken at least 10 days per month on a regular basis for at least 12 weeks in the year prior SV. f) Narcotics, such as opioids or barbiturates taken at least 4 days per month during 12 weeks prior SV. g) Permitted acute migraine medication taken at least 15 days per month for a non-migraine indication during 12 weeks prior SV. 7. Other: a) Prisoners or subjects who are involuntarily incarcerated. b) Subjects who are compulsorily detained for treatment of either a psychiatric or physical illness. c) Exposure to non-biological investigational agents within 30 days prior SV. d) Exposure to biological investigational agents within 24 weeks prior SV. e) Subjects who meet criteria for C-SSRS Suicidal Ideation Items 4 or 5 within the last 12 months OR subjects who endorse any of the 5 C-SSRS Suicidal Behavior Items within the last 10 years, OR subjects who, in the opinion of the Investigator, present a serious risk of suicide. f) Previous enrollment in any multiple

		dose BHV3000 study. Subjects may be considered for BHV3000-406 if the subject participated in any of the following single-dose studies: BHV3000-301, BHV3000-302, BHV3000-303, but did not participate in any multiple dose rimegepant study. g) Participation in any other investigational clinical trial while participating in this clinical trial. Subjects in a COVID-19 mRNA vaccine study (vaccine must be authorized) who are at least 30 days post last dose are permitted to be screened for this study. h) Past participation in a clinical study within 30 days prior SV. i) Failure to complete the Baseline Visit within the timeframe specified in the schedule of assessments. j) Subject considered to be otherwise clinically unsuitable for participation in the study.
**NCT05685225 2023**	Male or female ages 18 to 75 years, inclusive. At least 1-year of history of migraine with or without aura as defined by the International Classification of Headache Disorders 3rd edition (ICHD-3). Migraine onset before age 50 years. Able to not use opioids (including methadone and buprenorphine) during the study and for 7 days after taking the study medication.	Pregnant or nursing women or those planning a pregnancy. Use of opioids in the past 6 months. Use of medications to treat headache more than 10 days per month in the past 3 months. Use of barbiturate-containing medications, muscle relaxants, benzodiazepines, or marijuana in the past 6 months. Positive results for cocaine, marijuana, opiates, methamphetamines, and oxycodone in a urine drug screen. Symptoms consistent with chronic migraine, cranial neuropathy, and other pain syndromes. Headaches lasting more than 2 days in the past 3 months. More than one emergency care treatment for migraine in the past 12 months. Body mass index (BMI) equal to or greater than 37 kg/m2. Uncontrolled cardiovascular or cerebrovascular disease or a recent history of heart failure, atrial fibrillation, or myocardial infarction. Uncontrolled hypertension or diabetes. Current diagnosis of major depression, schizophrenia, dementia, significant neurological disorders, or other pain syndromes. Gastric or small intestinal surgery or malabsorption and hepatic disease. Significant hematologic, endocrine, pulmonary, renal, or gastrointestinal disease or history of malignancy in the past 5 years. History of alcohol or drug abuse or current cannabis use in the past 12 months. Known allergy to naltrexone or acetaminophen. Participation in another clinical trial. Immediate family members or same household members participating in the study. Site personnel, their friends, and family. Paid referral fees are not allowed. Abnormal laboratory or electrocardiogram (ECG) results such as bundle branch block, liver or kidney function tests, hemoglobin level, neutrophil count, cholesterol, or triglycerides.
**Nett 2003**	Non-pregnant female subjects using adequate contraception were eligible for this study if they were 18-65 years of age (inclusive), were able to give informed consent, had at least a 1- year history of migraine, with or without aura, as defined by 1988 International Headache Society criteria 1.1 and 1.2 and demonstrated a 6-month history of regularly occurring menstrually-associated migraine (MAM). MAM was defined as any migraine beginning on Day -2, -1, 1, 2, 3, or 4, with Day 1 = the first day of flow. Sites were instructed to enroll only subjects who reported MAM in at least 2 of their last 3 perimenstrual periods prior to screening, who could reliably predict the onset of menstrual flow and who typically experienced moderate to severe MAM pain preceded by a mild pain phase. Stable dose of oral contraceptive for at least 2 months if taking.	Subjects were excluded if they experienced >6 migraine attacks per month in either of the 2 months prior to screening; uncontrolled hypertension (sitting diastolic blood pressure ≥95mm Hg or systolic blood pressure ≥160mm Hg) at screening a history of epilepsy or structural brain lesions which lowered the convulsive threshold; confirmed or suspected cardiovascular, cerebrovascular, peripheral vascular, or ischemic bowel disease; impaired hepatic or renal function; ophthalmoplegic, basilar, or hemiplegic migraine; or tension-type headache on 15 or more days per month in any of the 2 months before screening. Other exclusion criteria included known hypersensitivity to the drug, the use of migraine prophylactic medication containing ergotamine, an ergot derivative, or methysergide; and use of a monoamine oxidase inhibitor within 2 weeks before the study.

**NTR33 2006**	1. At least 18 years of age at visit 1. 2. Current history of migraine with or without aura according to the International Headache Society (IHS) criteria. 3. Experienced an average of at least one migraine attack per month for six months prior to entry to the study. 4. Naïve to the use of 5HT1 agonists and ergotamine. 5. Willing and able to understand and complete questionnaires. 6. Willing and able to give informed consent prior to entry into the study.	1. A history suggestive of ischaemic heart disease (IHD) (e.g. angina pectoris) or any atherosclerotic disease which places them at increased risk of coronary ischaemia. 2. A history of cerebrovascular accident (CVA) or transient ischaemic attack (TIA). 3. A history of hypertension or a current blood pressure above 160/95 mmHg (measured three times). 4. A history of basilar, hemiplegic or ophthalmoplegic migraine. 5. Impaired hepatic or renal function. 6. A history of gastrointestinal disease. 7. A history of asthma. 8. Have a known or suspected hypersensitivity to, intolerance of, or contraindications to any component of the study medication. 9. Currently use propanolol as a prophylactic agent. 10. Currently use monoamine oxidase (MAO) inhibitors. 11. Currently abuse alcohol, analgesics or psychotropic drugs. 12. A history of hypertension. 13. Any severe concurrent medical condition, which may affect the interpretation in a clinical trial. 14. Females who are pregnant or breastfeeding, and females of childbearing potential who are not using a medically acceptable form of contraception. 15. Have participated in a clinical trial within the previous month or are currently participating in any other clinical research study or clinical trial.
**NTR34 2005**	1. The subject is greater than or equal to 18 years of age at visit 1. 2. The subject has a current history of migraine with or without aura according to the International Headache Society (IHS) criteria. 3. The subject has experienced an average of at least one migraine day per month for six months prior to entry to the study. 4. The subject is naïve to the use of 5HT1 agonists and ergotamine. 5. The subject is willing and able to understand and complete questionnaires. 6. The subject is willing and able to give informed consent prior to entry into the study.	1. Subjects with a history suggestive of ischaemic heart disease (IHD) (e.g. angina pectoris) or any atherosclerotic disease which places them at increased risk of coronary ischaemia. 2. Subjects with a history of cerebrovascular accident (CVA) or transient ischaemic attack (TIA). 3. Subjects who currently abuse alcohol, analgesics or psychotropic drugs. 4. Subjects who have any severe concurrent medical condition which may affect the interpretation in a clinical trial. 5. Subjects with a history of basilar, hemiplegic or ophthalmoplegic migraine. 6. Subjects with impaired hepatic or renal function. 7. Subjects who have a known or suspected hypersensitivity to, intolerance of, or contra-indications to any component of the study medication. 8. Females who are pregnant or breastfeeding, and females of childbearing potential who are not using a medically acceptable form of contraception. 9. Subjects who have participated in a clinical trial within the previous month or are currently participating in any other clinical research study or clinical trial. 10. Subjects with a history of hypertension or a current blood pressure above 160/95 mmHg (measured three times).
**Padma 1998**	Adults less than 55 years old diagnosed with migraine as per International Headache Society Classification (1988). Required at least one or more attack of acute migraine per month for at least one year and able to recognize the early signs of migraine.	More than 12 attacks per month, ischemic heart disease, diastolic blood pressure greater than 95 mmHg, psychiatric illness, Raynaud's phenomenon, pregnant or lactating mother, past history of hypersensitivity reactions to sumatriptan, taking more than 10 mg of ergotamine per week, or receiving calcium channel antagonists in preceding 4 weeks.
**Pascual 2000a**	Patients between 18 and 65 years of age suffering from migraine with or without aura, according to the criteria established by the International Headache Society, were recruited. In addition, patients had suffered from migraine attacks for at least 1 year and had one to six attacks per month.	Migraine with prolonged aura, familial hemiplegic migraine migrainous infarction or vertebrobasilar migraine; clinically relevant abnormal screening results; breastfeeding or pregnant women and women of child-bearing potential without adequate contraceptive protection; any chronic physiological or neuropsychiatric illness; antipsychotic or antidepressant medication taken during the 3 months period prior to the study; prophylactic drugs for migraine taken during the 2 weeks prior to the study; and intolerance or known hypersensitivity to sumatriptan or other related compounds.

**Pascual 2000b**	A total of 882 women and men who met International Headache Society (IHS) criteria for migraine with or without aura were enrolled. Patients had to have at least a 6-month history of migraine and usually experienced 1 to 8 attacks per month.	Patients were excluded if they had clinical evidence of cerebrovascular or cardiovascular disease including significant electrocardiogram (ECG) abnormality, or if they had a history within 1 year or current evidence of drug or alcohol abuse. Patients with any contraindication or sensitivity to 5-hydroxytryptamine $(5\text{-HT})_{1B/1D}$ agonists, or those who had received treatment with any other investigational compound or device within the past 30 days were also excluded, as were pregnant women or nursing mothers.
**Pfaffenrath 1998**	Men and women 18 to 65 years old experiencing one to six moderate or severe attacks of migraine, with or without aura (diagnosed according to International Headache Society (IHS) criteria), per month during the previous 12 months were eligible for the study.	Patients were excluded from the study for any of the following reasons: history of ischemic heart disease, hypertension, or atherosclerosis; supine diastolic blood pressure >95 mm Hg and/or systolic blood pressure >160 mm Hg; epilepsy or other brain disorder; pregnancy; lactation; current abuse of opiate analgesics; past year or current ergotamine abuse; and current use of lithium, monoamine oxidase inhibitors, serotonin reuptake inhibitors ergotamine-containing migraine prophylactic medications.
**Pini 1995**	Men and women from 18 to 65 years of age were admitted to the study if they had at least a 5-month history of severe or moderately severe migraine attacks, were able to distinguish migraine from other headaches and met the International Headache Society's (IHS) diagnostic criteria for migraine with or without aura.	Patients were excluded if they regularly used or abused opioid analgesics or other psychotropic drugs, if they had abused ergotamine within the last year, or if there was evidence of alcohol abuse. Women not using adequate contraceptive measures, or who were pregnant or breast feeding, were also excluded. Other exclusion criteria included ischaemic heart disease, uncontrolled hypertension, serious psychiatric illness or other systemic disease, and hypersensitivity to, intolerance of, or other contraindications against taking sumatriptan.
**Prior 2010**	The study inclusion criteria were: (1) men or women 18 years of age or older; (2) history of migraine with at least moderate pain intensity on a scale of none, mild, moderate, or severe that met the International Headache Society diagnostic criteria for migraine with or without aura;15 (3) fulfillment of the criteria for migraine after administration of an 11-page semi-structured diagnostic headache interview that assessed the following headache parameters: types, frequency and duration, intensity, exacerbation, pain location, quality of pain, nausea/vomiting, photophobia and phonophobia, visual aura, and sensorimotor aura (Clinical Assessment Form for Migraine Headache, Innovative Medical Research, Baltimore, MD, USA); (4) migraine headache attack frequency of at least 1 episode every 2 months but not more than 6 episodes per month in the past year before study entry; (5) history of treatment of previous migraines with over-the-counter medications; (6) ability to differentiate a migraine headache from an interval (tension-type) headache; and (7) if a woman, post-menopausal or using an effective form of birth control for at least 3 months before study entry.	Patients were excluded if they met any of the following conditions: (1) history of severely incapacitating migraines with more than 50% of episodes requiring bed rest or prohibiting performance of daily activities; (2) more than 20% of migraine episodes included vomiting; (3) routine experience of any other type of headache that would confound discrimination from migraine headache; (4) history of headaches because of other underlying pathology or related to head or neck trauma; (5) history of alcohol abuse, drug dependency, or history of significant psychiatric illness in the 12 months before study entry; (6) history of clinically significant renal or hepatic disease; uncontrolled hypertension; clinically significant coronary vascular disease not stable for the past. 6 months; history of seizures, cerebral vascular ischemia, infarct, hemorrhage, or other central nervous system disease; unstable metabolic disease, hypoglycemia, or diabetes; malignancy within the past 5 years; active tuberculosis; or prior gastrointestinal surgery which could influence the absorption, metabolism, or excretion of study medication; (7) history of allergy or sensitivity to acetaminophen; (8) current use of nonsteroidal anti-inflammatory drugs (NSAIDs), aspirin or analgesics on a regular basis (more than 15 days per month), except low-dose (325 mg daily or less) aspirin for cardiovascular prophylaxis; or (9) previous enrollment in this study, a recent (within 1 year) McNeil migraine headache study, or any other investigational drug study (within 30 days).

**Rapoport 1997**	Male and female outpatients, 12 to 65 years old, with migraine with or without aura (as defined by International Headache Society criteria) were enrolled in the study. All patients were required to have a migraine history of at least 1-year duration, with an age at onset of less than 50 years, and an average of one to six migraine headaches per month over the preceding 6 months.	Pregnancy; lactation; inadequate contraception; any laboratory result that in the opinion of the investigator represented a clinically important abnormality; concomitant medical or psychiatric conditions likely to interfere with treatment; usual blood pressure of 160/95 mm Hg; nonmigraine headaches on more than 10 day/month during the preceding 6 months; ischemic heart disease or other vascular disease including Prinzmetal’s angina, Wolff-Parkinson-White syndrome, or other cardiac accessory conduction pathways; basilar, ophthalmoplegic, or hemiplegic migraine; arrhythmias; alcohol/drug abuse; participation in another clinical trial in the previous 30 days; hypersensitivity to any ingredient of the zolmitriptan tablet or any serotonin-like drugs; or concurrent treatment with nmonoamine oxidase inhibitors, methysergide, or methylergonovine.
**Rapoport 2002**	Men and women, aged 18 to 65 years, were eligible for inclusion if they had a history of moderate or severe migraine for at least 1 year, with onset before the age of 50 years. In addition, they were required to have experienced one to six attacks per month for at least 2 months immediately prior to enrollment.	Patients with basilar or hemiplegic migraine, 15 or more headache days per month, or a diagnosis of migraine with headaches of other etiology that could not be reliably distinguished from migraine at onset were excluded from the studies. Other exclusion criteria were clinically significant cerebrovascular, cardiac, hepatic, or renal disease. Women who were pregnant or breast-feeding were also excluded.
**Rederich 1995**	Adult migraineurs at least 18 years old who provided written informed consent were eligible. Patients were required to have at least a 1-year history of migraine with aura or without aura diagnosed according to International Headache Society criteria, and to have had two to six migraine attacks monthly during the previous 60 days. They were also required to have a normal electrocardiogram (ECG) at screening.	Patients with a history suggestive of ischemic heart disease or Raynaud's syndrome, diastolic blood pressure greater than 95 mmHg or systolic blood pressure greater than 160 mmHg at screening, or chronic tension-type headache diagnosed according to IHS criteria were excluded. Women with a positive urine pregnancy test were also excluded.
**Ryan 2002a**	18 to 65 years old, have at least 12 months history of migraines, with or without aura, according to International Headache Society (IHS)-criteria; and have a frequency of migraine attacks between 1 to 8 per month over at least the previous 2 months and be <50 years of age at initial migraine diagnosis.	Clinical reasons for exclusion included significant renal, hepatic, cardiovascular, or cerebrovascular disease, including vertebrobasilar or hemiplegic migraine; pregnancy; breast-feeding, or more than 15 headache days per month.
**Ryan 2002b**	18 to 65 years old, have at least 12 months history of migraines, with or without aura, according to International Headache Society (IHS)-criteria; and have a frequency of migraine attacks between 1 to 8 per month over at least the previous 2 months and be <50 years of age at initial migraine diagnosis	Clinical reasons for exclusion included significant renal, hepatic, cardiovascular, or cerebrovascular disease, including vertebrobasilar or hemiplegic migraine; pregnancy; breast-feeding, or more than 15 headache days per month.
**Ryan 2002c**	18 to 65 years old, at least 12 months history of migraines, with or without aura, according to International Headache Society (IHS)-criteria; and a frequency of migraine attacks between 1 to 8 per month over at least the previous 2 months and be <50 years of age at initial migraine diagnosis.	Clinical reasons for exclusion included significant renal, hepatic, cardiovascular, or cerebrovascular disease, including vertebrobasilar or hemiplegic migraine; pregnancy; breast-feeding, or more than 15 headache days per month.

**S2WA4003 1998**	Males and nonpregnant females using adequate contraception were eligible if they were between 18 and 65 years of age (inclusive), had at least a 1-year history of migraine with or without aura as defined by the 1988 International Headache Society criteria, had 1 to 6 migraines monthly during the 2 months (60 days) preceding the screening visit, had a history of moderate to severe pain during migraine attacks, was able to distinguish migraine attacks as discrete attacks from other headaches (i.e. tension-type headaches), was able to understand and complete the subject questionnaires and diary cards, and could read and write English.	Subjects were excluded if they: had a history of 5-hydroxytriptamine (5-HT1) specific agonist use on more than 3 occasions and/or had received a prescription for sumatriptan succinate tablets any time prior to screening; had confirmed or suspected ischemic heart disease (angina pectoris, history of myocardial infarction, or documented silent ischemia), or Prinzmetal's angina, or signs/symptoms consistent with any of the above; had evidence or history of ischemic abdominal syndromes, peripheral vascular disease or Raynaud's Syndrome; had cardiac arrhythmias requiring medication or a clinically significant electrocardiogram abnormality; had a history of cerebrovascular pathology including stroke; had a history of congenital heart disease; had uncontrolled hypertension at screening (sitting systolic pressure ≥160 mmHg, diastolic pressure ≥95 mmHg; was, in the investigator's opinion, likely to have unrecognized cardiovascular or cerebrovascular disease; had a history of epilepsy; had basilar or hemiplegic migraine; had tension-type headaches >15 days/month in either of the two months (60 days) prior to screening; had impaired hepatic or renal function; had any severe concurrent medical condition which could affect the interpretation of efficacy and safety data or otherwise contraindicated participation in a clinical trial; had evidence or history of ergotamine abuse in the 3 months preceding screening; had evidence of alcohol, drug or substance abuse within the previous year which, in the investigator's judgement, would likely interfere with the study conduct, subject cooperation, or evaluation and interpretation of the study results; had hypersensitivity, intolerance, or contraindication to the use of naratriptan or any of its components; had a previous allergic reaction to prescription as well as to over-the-counter products containing naproxen, or had aspirin, or other nonsteroidal anti-inflammatory/analgesic drugs induce the syndrome of asthma, rhinitis, and nasal polyps; had prior history of peptic ulcer disease or gastrointestinal bleeding; had participated in an investigational drug trial within the previous four weeks, or planned to participate in another study at any time during the study.
**S2WA4004 1998**	Males and nonpregnant females using adequate contraception were eligible if they were between 18 and 65 years of age (inclusive), had at least a 1-year history of migraine with or without aura as defined by the 1988 International Headache Society criteria, had 1 to 6 migraines monthly during the 2 months (60 days) preceding the screening visit, had a history of moderate to severe pain during migraine attacks, was able to distinguish migraine attacks as discrete attacks from other headaches (i.e. tension-type headaches), was able to understand and complete the subject questionnaires and diary cards, and could read and write English	History of 5-hydroxytriptamine (5-HT1) specific agonist use on more than 3 occasions and/or had received a prescription for sumatriptan succinate tablets any time prior to screening; had confirmed or suspected ischemic heart disease (angina pectoris, history of myocardial infarction, or documented silent ischemia), or Prinzmetal's angina, or signs/symptoms consistent with any of the above; had evidence or history of ischemic abdominal syndromes, peripheral vascular disease or Raynaud's Syndrome; had cardiac arrhythmias requiring medication or a clinically significant electrocardiogram abnormality; had a history of cerebrovascular pathology including stroke; had a history of congenital heart disease; had uncontrolled hypertension at screening (sitting systolic pressure > =160 mmHg, diastolic pressure ≥95 mmHg); was, in the investigator's opinion, likely to have unrecognized cardiovascular or cerebrovascular disease; had a history of epilepsy; had basilar or hemiplegic migraine; had tension-type headaches >15 days/month in either of the 2 months (60 days) prior to screening; had impaired hepatic or renal function; had any severe concurrent medical condition which could affect the interpretation of efficacy and safety data or otherwise contraindicated participation in a clinical trial; had evidence or history of ergotamine abuse in the 3 months preceding screening; had evidence of alcohol, drug or substance abuse within the previous year which, in the investigator's judgement, would likely interfere with the study conduct, subject cooperation, or evaluation and interpretation of the study results; had hypersensitivity, intolerance, or contraindication to the use of naratriptan or any of its

		components; had a previous allergic reaction to prescription as well as to over-the-counter products containing naproxen, or had aspirin, or other nonsteroidal anti-inflammatory/analgesic drugs induce the syndrome of asthma, rhinitis, and nasal polyps; had prior history of peptic ulcer disease or gastrointestinal bleeding; had participated in an investigational drug trial within the previous 4 weeks, or planned to participate in another study at any time during the study.
**S2WB3002 1996**	Males and nonpregnant females using adequate contraception of any race were eligible if they were between 18 and 65 years of age (inclusive), had at least a 12-month history of migraine with or without aura as defined by the 1988 International Headache Society criteria, had 1 to 6 migraines monthly during the 6 months prior to the study, had a history of severe or moderately severe pain during migraine attacks, and could distinguish migraine headaches from other headaches (e.g. tension-type headache).	Subjects were excluded if they had a history of ischaemic heart disease (IHD) (i.e. angina pectoris, history of myocardial infarction or documented silent ischaemia), Prinzmetal's angina, coronary vasospasm, symptoms or signs consistent with IHD on the screening electrocardiogram (ECG); history of atherosclerotic disease (coronary vascular disease, pulmonary vascular disease or Raynaud's disease) which placed them at increased risk of coronary ischaemia; cardiac arrhythmias requiring medication; uncontrolled hypertension (treated or untreated), supine blood pressure =160mmHg and/or supine diastolic blood pressure of =95mmHg (lowest of 3 measurements after 15 minutes supine rest at the time of entry to the study); evidence of end organ damage; any concurrent medical condition which might affect the interpretation of efficacy and safety data or which otherwise contraindicated participation in a clinical study of a new chemical entity; a history of epilepsy or structural brain lesions which would lower their convulsion threshold; a history of basilar or hemiplegic migraine; impaired hepatic/renal function; concurrent use of monoamine oxidase (MAO) inhibitors or lithium or use of any of these medications in the previous 2 weeks; concurrent use of selective-5-hydroxytryptamine (5HT) re-uptake inhibitors (SSRIs) or use in the previous 2 weeks (only in those countries in which it was contra-indicated for use with sumatriptan i.e. Germany, Italy, Norway, Spain, Sweden and United Kingdom); current abuse of opiate analgesia/other psychoactive drugs or history of abuse during the previous 12 months; current abuse of ergotamine (>10mg/week) or a history of abuse during the previous 12 months; current abuse of alcohol (>315g/week); known hypersensitivity to, intolerance of, or contraindications to the use of sumatriptan, naratriptan or other unlicensed drugs in this class; known hypersensitivity to sulphonamides; participation in a clinical study with an unregistered product within 1 month prior to entry in the study or participation in another clinical study with an unregistered product during the period of the study.
**S2WB4001 1998**	Males and nonpregnant females using adequate contraception were eligible if they were between 18 and 65 years of age (inclusive), had at least a 6-month history of migraine with or without aura as defined by the 1988 International Headache Society criteria, had experienced between one to six attacks severe (grade 3) or moderate (grade 2) migraine attacks per month for at least 2 months prior to entry to the study, and were able to distinguish migraine headaches from other headache types (e.g. tension-type headache). Subjects were eligible if the major reason for discontinued treatment with oral sumatriptan 100mg or subcutaneous sumatriptan 6mg was either tolerability or efficacy.	Subjects were excluded if they had uncontrolled hypertension (sitting diastolic blood pressure >95 mmHg or systolic blood pressure >160 mmHg) at screening; a history of epilepsy, confirmed or suspected cardiovascular, cerebrovascular, or peripheral vascular disease, impaired hepatic or renal function; basilar, hemiplegic, or ophthalmoplegic migraine. Other exclusion criteria included known hypersensitivity to naratriptan or sumatriptan, use of migraine prophylactic medication containing ergotamine, an ergot derivative, or methysergide. Subjects were excluded if they had lapsed from use of sumatriptan 50mg or naratriptan 2.5mg.

**S2WB4003 UN**	Not reported.	Not reported.
**S98-073 UN**	Acute migraine attack of moderate to severe pain intensity with or without aura (International Headache Society 1.1 or 1.2).	Not reported.
**S98-074 UN**	Acute migraine attack of moderate to severe pain intensity with or without aura (International Headache Society 1.1 or 1.2).	Not reported.
**Sakai 2002**	Male and female patients aged 18-64 years, with an established diagnosis of migraine with or without aura (according to International Headache Society criteria), were included in the study. Eligible patients had an age at migraine onset of <50 years, a history of migraine symptoms for at least 1 year and experienced one to six migraine attacks per month in the 3 months prior to the study.	A history of basilar, ophthalmoplegic or hemiplegic migraine; non-migraine headaches reported on >10 days per month during the previous 6 months; ischaemic heart disease, dysrhythmias or cardiac accessory pathway disorders (e.g. Wolff-Parkinson–White syndrome); severe liver or renal impairment; uncontrolled hypertension; pregnancy or lactation; severe allergies or hypersensitivity to drugs; participation in a clinical study during the past 3 months; or required use of ergotamine preparations.
**Sakai 2021**	Eligible participants were aged ≥18 years who had a history of migraine with or without aura for ≥1 year (International Headache 1.2.111); Society [IHS] diagnostic criteria 1.1 and/or onset at <50 years; three to eight migraine attacks per month (<15 headache days/month for the past 3 months); and disabling migraine defined as Migraine Disability Assessment (MIDAS) score ≥11.	Participants with known lasmiditan sensitivity; history of chronic migraine or other chronic headache disorders with ≥15 headache days/month within the past 12 months; hemorrhagic stroke, epilepsy, or any other condition placing the participant at increased risk of seizures, recurrent dizziness, and/or vertigo; diabetes mellitus with complications; orthostatic hypotension with syncope; significant renal or hepatic impairment; and participants who, in the investigator's judgment, were a significant suicide risk.
**Sandrini 2002**	Eligible patients were men and women 18 years of age (in Canada there was also an age limit of 65 years) who were expected to have at least one attack of migraine with or without aura, as defined by the International Headache Society (IHS) criteria,15 every 6 weeks. Patients had to be capable of taking study medication as outpatients and recording the effects.	Patients who had previously taken oral eletriptan or any formulation of sumatriptan were excluded from the trial, as were patients who had taken any experimental drug within the previous month. In addition, patients could not use sumatriptan, ergotamine, or any ergotamine like agent within 48 hours before, or 24 hours after, taking the study medication. Patients could not use any proprietary analgesic or antiemetic within 6 hours before, or 2 hours after, taking the study medication. Pregnant or breast-feeding women and those not using adequate contraception were excluded from the trial. Patients with frequent non-migrainous headache, atypical migraine that had not previously responded to therapy, migraine with prolonged aura, familial hemiplegic migraine, basilar migraine, or migrainous infarction were excluded from the trial. Patients with a history of heart disease, uncontrolled hypertension, cardiac arrhythmias, abnormalities on laboratory tests or electrocardiogram (ECG), documented allergic reactions to drugs, or any other clinically significant disease were also

		excluded. Informed consent was obtained from all patients, and independent ethics committees in the appropriate countries approved the study prior to implementation.
**Saper 2006**	The study was conducted in outpatients aged ≥18 years diagnosed with migraine with or without aura as defined by the International Headache Society. Patients must have had, on average, 1 to 8 migraine attacks per month in the 6 months prior to enrollment.	Patients having difficulty in distinguishing migraine attacks from tension or interval headaches; pregnant or nursing women; sexually active women of childbearing potential who were unwilling to use barrier or oral contraception; hypersensitivity or history of a serious adverse event in response to rofecoxib or ibuprofen; evidence of uncontrolled hypertension or significant pulmonary, renal, hepatic, endocrine, neurologic (apart from migraine), psychiatric or other systemic disease, or laboratory abnormality; history of drug or alcohol abuse within 1 year prior to study start; and the use of treatment with an investigational compound or device within 30 days of study start.
**Sargent 1995**	Age 18 to 65 with a 1-year history of migraine. 1 to 6 migraine attacks per month within past 2 months.	No diastolic pressure greater than 95 or systolic greater than 160. No pregnant or breast-feeding women.
**Savani 1999**	Males and nonpregnant females using adequate contraception were eligible if they were between 18 and 65 years of age (inclusive), were able to give informed consent. were sumatriptan naïve, had at least a 12-month history of migraine with or without aura as defined by the 1988 International Headache Society criteria, had 1 to 6 migraines per month of moderate or severe intensity, and were able to distinguish migraine headaches from other headache types.	Supine diastolic blood pressure >95 mmHg or supine systolic blood pressure >160 mmHg at screening; confirmed or suspected history or evidence of ischaemic heart disease, history of atherosclerotic disease which would place the subject at an increased risk of coronary ischemia (eg. coronary or peripheral vascular disease), previous myocardial infarction, Prinzmetal's angina/coronary vasospasm, obliterative vascular disease or Raynaud's syndrome. Other exclusion criteria included known hypersensitivity to the drug, concurrent use of a monoamine oxidase inhibitor, serotonin reuptake inhibitor or lithium; and current, or history of, abuse of ergotamine and current abuse of opiate analgesics or other psychotropic drugs.
**Savi 2011**	The study included subjects of male or female gender, 18 to 65 years old, with a current history of migraine with or without aura, according to International Headache Society (IHS) 2004 criteria, and with at least one, but no more than six migraine attacks per month for 6 months prior to entering the study.	(a) Uncontrolled hypertension; (b) ischemic heart disease; (c) cardiac arrhythmias or symptomatic Wolff–Parkinson–White syndrome; (d) previous stroke or transient ischemic attack; (e) severe liver or renal impairment; (f) any other severe or disabling medical condition; (g) history of alcohol or analgesic or psychotropic drug abuse; (h) known hypersensitivity to study drugs; (i) previously demonstrated inadequate response to at least two triptans; (j) current use of propranolol or ergotamine (and its derivatives) as a prophylactic agent; (k) current use or use in the previous 2 weeks of monoaminoxidase (MAO)-inhibitors; (l) use of either test medication to treat any one of the last three episodes of migraine; and (m) other headaches that have been lasting for more than 6 days. Pregnant women and breast-feeding mothers were excluded as well, while women with childbearing potential but not practicing an effective method of birth control were to be submitted to a pregnancy test, if clinically indicated.

**Savi 2014**	Patients included both male and female subjects aged between 18 and 55 years, with a current history of migraine with or without aura, according to International Headache Society (IHS) 2004 criteria, and with at least one but no more than six migraine attacks per month in the 6 months prior to entering the study.	Patients could not be enrolled in the study in case of: 1) uncontrolled hypertension; 2) ischemic heart disease; 3) cardiac arrhythmias or symptomatic Wolff–Parkinson–White syndrome; 4) previous stroke or transient ischemic attack; 5) severe liver or renal impairment; 6) any other severe or disabling medical condition, 7) a history of alcohol, analgesic, psychotropic drug abuse; 8) known hypersensitivity to study drugs, 9) previously demonstrated inadequate response to at least two triptans, 10) current use of propranolol or ergotamine (and its derivatives) as a prophylactic agent; 11) current use or use of in the previous 2 weeks of monoamine oxidase inhibitors; 12) use of either test medication to treat any one of the last three episodes of migraine; and 13) other headaches that had lasted for more than 6 days (at the time of presentation for the study); 14) pregnant women and breastfeeding mothers were excluded as well.
**Seeburger 2012**	The target population was men and women 18 years of age with a history of migraine for more than 1 year, with or without aura, as defined by the International Headache Society. Eligible participants had, by self-report, a minimum of 2 moderate-to-severe migraine attacks per month during the 3 months before randomization while taking a stable dose of topiramate for migraine prophylaxis (minimum dose of 50 mg). One additional medication for migraine prophylaxis was permitted, provided that the prescribed dose was stable for 3 months before randomization. Eligible participants had to be able to clearly distinguish migraine attacks from other types of headache. Female participants of childbearing potential agreed to use adequate contraception during the study.	Participants with more than 15 headache-days/month or who had taken medication for acute headache on more than 10 days/month in any of the previous 3 months were excluded. Participants with ischemic heart disease, uncontrolled hypertension, coronary artery vasospasm (including Prinzmetal's variant angina), or other significant underlying cardiovascular diseases were also excluded.
**Sheftell 2003**	Men and women over 18 years of age. History of at least one typical attack of migraine with or without aura every 6 weeks. as defined by the International Headache Society (IHS). Patients also had to be capable of taking study medication as outpatients and recording its effects.	Patients who had previously taken oral eletriptan were excluded from the trial, as were patients who had taken any investigational drug within the previous month. Potentially fertile, sexually active women not using adequate contraception were excluded from the trial, as were women who were pregnant or breastfeeding. Patients with frequent non-migrainous headache, atypical migraine that had not consistently responded to therapy, migraine with prolonged aura, familial hemiplegic migraine, basilar migraine, or migrainous infarction were excluded from the trial. Patients with a history of heart disease, hypertension or arrhythmias, clinically significant abnormalities in laboratory tests or an electrocardiogram (ECG), documented allergic reactions to drug, or any other clinically significant disease were also excluded.
**Sheftell 2005a**	Males and non-pregnant females using adequate contraception were eligible if they were between 18 and 65 years, were able to give informed consent, had at least a 6-month history of migraine, with or without aura as defined by the 1988 International Headache Society criteria 1.1 and 1.2, had the ability to distinguish migraine attacks from other headaches and typically experienced moderate to severe migraine pain, had 1 to 6 moderate/severe migraines per month in each of the previous 3 months, and had or had not used 5-hydroxytryptamine (5-HT1) agonists to treat migraines in the past.	Subjects were excluded if they had uncontrolled hypertension (diastolic blood pressure ≥95mm Hg or systolic blood pressure ≥160mm Hg) at screening; a history of epilepsy or structural brain lesions which lowered the convulsive threshold; confirmed or suspected cardiovascular, cerebrovascular, peripheral vascular, or ischemic bowel disease; impaired hepatic or renal function; basilar or hemiplegic migraine; or headache on 15 or more days per month in any of the 3 months before screening. Other exclusion criteria included known hypersensitivity to the drug, use of migraine prophylactic medication containing ergotamine, an ergot derivative, or methysergide; and use of a monoamine oxidase inhibitor within 2 weeks before screening. Subjects had to be willing and able to use the electronic diary.

**Sheftell 2005b**	Males and non-pregnant females using adequate contraception were eligible if they were between 18 and 65 years, were able to give informed consent, had at least a 6-month history of migraine, with or without aura as defined by the 1988 International Headache Society criteria 1.1 and 1.2, had the ability to distinguish migraine attacks from other headaches and typically experienced moderate to severe migraine pain, had 1 to 6 moderate/severe migraines per month in each of the previous 3 months, and had or had not used 5-hydroxytryptamine (5-HT1) agonists to treat migraines in the past.	Subjects were excluded if they had uncontrolled hypertension (diastolic blood pressure ≥95mm Hg or systolic blood pressure ≥160mm Hg) at screening; a history of epilepsy or structural brain lesions which lowered the convulsive threshold; confirmed or suspected cardiovascular, cerebrovascular, peripheral vascular, or ischemic bowel disease; impaired hepatic or renal function; basilar or hemiplegic or ophthalmoplegic migraine ; or headache on 15 or more days per month in any of the 3 months before screening. Other exclusion criteria included known hypersensitivity to the drug, use of migraine prophylactic medication containing ergotamine, an ergot derivative, or methysergide; current use of a selective serotonin re-uptake inhibitor (SSRI) in participating countries where they are contraindicated for use with sumatriptan, and use of a monoamine oxidase inhibitor within 2 weeks before screening. Subjects had to be willing and able to use the electronic diary.
**Smith 2005**	Males and non-pregnant females 18 years of age and older with a diagnosis of migraine with or without aura, according to the International Headache Society (IHS) Classification Criteria (and were recruited for participation. Eligible subjects had a history of at least 2, but not more than 6 migraine attacks per month during the preceding 12 months. Subjects had a history of tolerating oral treatment with a 5-hydroxytryptamine (5-HT) agonist (triptans or ergotamine derivatives) for migraine.	Not reported.
**Solomon 1997**	Female and male patients between 12 and 65 years of age who were being treated for migraine were recruited from neurology clinics and primary care centers. All patients had migraine with or without aura, as defined by the International Headache Society criteria." Eligible patients had migraine for ≥1 year, an average of one to six migraines per month over the past 6 months that lasted 4 to 72 hours, and age at onset <50 years. Patients needed to be able to distinguish nonmigraine from typical migraine headaches. Specifically, they were required to identify migraine attacks as those headaches characterized by at least two of the following four criteria: unilateral location; pulsating quality; moderate or severe intensity; aggravation by physical activity; and nausea, vomiting, or photophobia and phonophobia.	For study inclusion, during the screening visit (screening phase) patients had a 12-lead electrocardiogram (ECG) performed that was judged to show no evidence of ischemic heart disease, arrhythmia (e.g., atrial fibrillation/flutter, frequent premature ventricular contractions, atrioventricular block), or accessory conduction pathways (e.g., Wolff-Parkinson-White syndrome). Patients who had an acceptable screening-phase ECG and gave their informed consent were recruited into the study. Key exclusion criteria were as follows: 1. previous medical or psychiatric condition judged by the investigator to put the patient at a potential risk with exposure to zolmitriptan or that may have interfered with the safety or efficacy assessments; 2. hypertension (usual systolic 165 mm Hg or diastolic 95 mm Hg); history of basilar, ophthalmoplegic, or hemiplegic migraine headaches or nonmigraine headaches occurring an average of >10 days month for the preceding 6 months; use of monoamine oxidase inhibitors; hypersensitivity to any ingredient of zolmitriptan or serotonin-like drugs; pregnancy, breast-feeding, or planning pregnancy; participation in another clinical trial or any zolmitriptan clinical study within 30 days before screening. Patients randomized to treatment were instructed not to take study medication if they had used nonsteroidal anti-inflammatory agents, analgesics, sedatives, or antiemetics in the previous 6 hours or had used.
**Spierings 2001**	Men and women were eligible for the study if they were between 18 and 65 years, suffered from migraine with or without aura, as defined under codes 1.2 and 1.1, respectively, by the International Headache Society (IHS), and were otherwise healthy. They had to be of sound mind; able to read and understand the informed consent form, written in English; able to comply with the requirements of the study; and voluntarily consent to it. They had to have had a history of migraine headaches for at least 6 months, with an onset before the age of 50 years, and no history of head or neck trauma	The subjects could not have uncontrolled hypertension, defined as a diastolic blood pressure higher than 95 mm Hg or a systolic blood pressure higher than 160 mm Hg, or clinically significant disease affecting any system but especially the cardiovascular or gastrointestinal tract. They could not have (a history of) gastrointestinal disease or surgery that would affect the absorption of medications taken orally and had to be mentally stable, without significant psychiatric disease, or have a history of substance abuse as defined by the Diagnostic and Statistical Manual of Mental Disorders, Fourth Edition, within the preceding year. They also could not be using opioids or tranquilizers to the extent that, in the investigator's opinion, it would interfere with the determination of the efficacy, tolerability, or safety of the study

	within the preceding 6 months. They also had to have had an average of at least 2 moderate or severe migraine headaches per month during the preceding 3 months, with an interval of at least 24 hours between consecutive attacks. The subjects could not suffer from migraine with prolonged aura (IHS code 1.2.2), migrainous infarction (IHS code 1.6.2), hemiplegic (IHS code 1.2.3), or basilar migraine (IHS code 1.2.4). Subjects were allowed to have other than migraine headaches, for example, tension or sinus headaches, but had to be able to distinguish between them and their migraine headaches. Women were required to be either menopausal or agree to avoid pregnancy and not be nursing for the duration of the study. The non-menopausal women had to have used a reliable method of contraception for at least 2 months before enrolling in the study. In addition, they had to have a negative result for a serum pregnancy test at screening and at day 30 of the study, if applicable. Also at screening, they were required to have a clinically acceptable physical examination, blood tests, and electrocardiogram (ECG); the ECG was considered clinically unacceptable if the corrected QT interval was longer than 450 milliseconds for men or 470 milliseconds for women.	medications. Preventive migraine treatment was allowed with the exclusion of monoamine oxidase inhibitors, lithium carbonate, cyproheptadine hydrochloride, methysergide maleate, ergotamine tartrate, and dihydroergotamine mesylate; taking of these medications had to be discontinued at least 2 weeks before enrollment. Subjects who were triptan naive were also excluded from the study, as well as those with hypersensitivity to or contraindications for the use of triptans, in particular sumatriptan, or ergots, that is, ergotamine and dihydroergotamine. They also could not have had exposure to almotriptan or have participated in an investigational drug or device study within 1 month before screening.
**Spierings 2004**	We enrolled patients between 18 and 65 years of age (inclusive), who had an established diagnosis of migraine, with or without aura, as defined by International Headache Society (IHS)-criteria. Patients were eligible if the age at migraine onset was <50 years, and if they had at least two migraine headaches per month and <10 days of non-migraine headaches per month for the 3 months prior to study entry. In addition, individuals were enrolled only if they were able to distinguish migraine from non-migraine headaches. Women of childbearing potential were required to use a reliable method of birth control.	Patients were excluded from the study if they had a history or symptoms suggestive of ischaemic heart disease or any other vascular disease, clinically significant electrocardiogram (ECG) abnormalities, or uncontrolled hypertension (systolic blood pressure ≥160mm Hg or diastolic blood pressure ≥90mm Hg). Patients with basilar, ophthalmoplegic or hemiplegic migraine or a serious neurological condition associated with headaches were also excluded. Patients were also not eligible if they required concomitant treatment with propranolol or cimetidine or had used a monoamine oxidase inhibitor (MAOI) within 2 weeks of randomisation, or initiated therapy with a selective serotonin reuptake inhibitor (SSRI) and the dose had not stabilised within 2 weeks of randomisation.
**Stark 2002**	Male and female patients aged over 18 years, who had a history of at least one migraine attack every 6 weeks as defined by the International Headache Society (IHS) diagnostic criteria for migraine with or without aura.	Patients with concomitant frequent non-migrainous headache (i.e. more than 6 attacks per months on average), atypical migraine that had consistently failed to respond to migraine therapy, or a history of migraine with prolonged aura, familial hemiplegic migraine, basilar migraine, or migrainous infarction. In addition, patients with clinical evidence or history of other significant (including cardiovascular) disease, severely limited gastrointestinal absorption, documented drug allergy, drug or alcohol misuse, or who intended to donate blood for up to 1 month following completion of the study, or were likely to be non-complaint with the study procedures, were excluded. Female patients who were pregnant, breast-feeding or not taking effective contraceptive precautions were not considered for entry. Concomitant treatment with any investigational drug, including eletriptan, in the month before entry was prohibited. Treatment with analgesics or ergotamine on more than 2 days in any 7-day period during the study was prohibited. Patients taking migraine prophylactic treatments were eligible for entry; however, treatment with analgesics or antiemetics in the 6 h before dosing or with sumatriptan or ergot derivatives in the 48 h before dosing was not permitted.

**Steiner 2003**	Male or female patients aged 18 to 65 years were included, meeting International Headache Society (IHS)-criteria for migraine with or without aura and with attacks at least once every 6 weeks. All gave informed consent in line with recommendations of IHS prior to a physical examination, 12-lead ECG and (in women of child-bearing potential) a urine pregnancy test.	Migraine that had been consistently resistant to all treatments; basilar migraine; hemiplegic migraine; frequent non-migrainous headaches; any clinically significant medical illness or laboratory abnormalities, especially those indicative of coronary artery disease, heart failure or uncontrolled hypertension; other contraindications to treatment with eletriptan or zolmitriptan including use of potent CYP3A4 inhibitors concomitantly or of MAO inhibitors within 2 weeks of entry; severe reduction in gastrointestinal absorption; misuse of alcohol or other substances including analgesics. ergotamine or triptans; pregnancy or breast-feeding. Women who might become pregnant were required to use effective contraception.
**Stronks 2003**	1) Between 18 and 65 years of age at the beginning of the study, 2) had experienced 1 to 6 moderate or severe migraine attacks per month for at least 2 months before entry to the study, 3) did not experience more than 6 days of tension-type headache per month, 4) had not used 5-hydroxytryptamine (5-HT1) agonists or naproxen during the previous year in the treatment of acute migraine, with the exception of when participating in a double-blind study, 5) had the ability to distinguish migraine headache from other headache types (eg, tension-type headache) early in the onset of a migraine attack.	1) Had a history of drug or alcohol abuse or a positive history of psychiatric illness, 2) had a current illness, other than migraine, interfering with locomotion and physical activities, 3) used prophylactic antimigraine medication or other medication that could influence the subjective or objective (or both) outcome measures, 4) had experienced recent circadian shifts, including working at night, 5) were contraindicated for a treatment with naratriptan or naproxen according to the local instruction sheets, 6) were, in the opinion of the investigator, unsuitable for treatment with a 5-HT1 agonist, 7) had a history of cardiovascular or neurologic (or both) disease.
**SUM20033 2003**	Males and nonpregnant females using adequate contraception were eligible if they were between 18 and 65 years, were able to give informed consent, had at least a 1-year history of migraine, with or without aura as defined by the 1988 International Headache Society criteria 1.1 and 1.2), had the ability to distinguish migraine attacks from other headaches and typically experienced moderate to severe migraine pain, had 1 to 6 moderate/severe migraines per month in each of the previous 3 months, and had or had not used 5-hydroxytryptamine (5-HT1) agonists to treat migraines in the past.	Subjects were excluded if they had uncontrolled hypertension (diastolic blood pressure ≥95mm Hg or systolic blood pressure ≥160mm Hg) at screening; a history of epilepsy or structural brain lesions which lowered the convulsive threshold; confirmed or suspected cardiovascular, cerebrovascular, peripheral vascular, or ischemic bowel disease; impaired hepatic or renal function; basilar or hemiplegic migraine; or headache on 15 or more days per month in any of the 3 months before screening. Other exclusion criteria included known hypersensitivity to the drug, use of migraine prophylactic medication containing ergotamine, an ergot derivative, or methysergide; and use of a monoamine oxidase inhibitor within 2 weeks before screening.
**SUMA4016 1998**	Males and nonpregnant females using adequate contraception were eligible if they were between 18 and 65 years of age (inclusive), had at least a 1-year history of migraine with or without aura as defined by the 1988 International Headache Society criteria, had 1 to 6 moderate or severe migraines monthly during the 2 months (60 days) preceding the screening visit, were able to distinguish migraine attacks as discrete attacks from other headaches (i.e. tension-type headaches); were able to understand and complete the subject questionnaires and diary cards, and could read and write English.	Patients were excluded if they had uncontrolled hypertension (sitting diastolic blood pressure ≥95 mmHg or systolic blood pressure ≥160 mmHg) at screening; a history of epilepsy; confirmed or suspected cardiovascular, cerebrovascular, or peripheral vascular disease; impaired hepatic or renal function; basilar or hemiplegic migraine; tension-type headache on 15 or more days per month; or prior history of peptic ulcer disease or gastrointestinal bleeding. Other exclusion criteria included known hypersensitivity to sumatriptan; a previous allergic reaction to prescription as well as to over-the-counter products containing naproxen, or had aspirin, or other nonsteroidal anti-inflammatory/analgesic drugs induce the syndrome of asthma, rhinitis, and nasal polyps; abuse of ergotamine in the 3 months before screening; evidence of alcohol, drug or substance abuse within the previous year; use of a monoamine oxidase inhibitor within 2 weeks before screening. Subjects with a history of sumatriptan succinate tablet or other 5-hydroxytryptamine (5HT1) specific agonist use on more than 3 occasions and/or had received a prescription for sumatriptan succinate tablets any time prior to screening were also excluded.

**SUMA4017 1998**	Males and nonpregnant females using adequate contraception were eligible if they were between 18 and 65 years of age (inclusive), had at least a 1-year history of migraine with or without aura as defined by the 1988 International Headache Society criteria, had 1 to 6 migraines monthly during the 2 months (60 days) preceding the screening visit, had a history of moderate to severe pain during migraine attacks, was able to distinguish migraine attacks as discrete attacks from other headaches (i.e. tension-type headaches), was able to understand and complete the subject questionnaires and diary cards, and could read and write English.	Patients were excluded if they had uncontrolled hypertension (sitting diastolic blood pressure ≥95 mmHg or systolic blood pressure ≥160 mmHg) at screening; a history of epilepsy; confirmed or suspected cardiovascular, cerebrovascular, or peripheral vascular disease; impaired hepatic or renal function; basilar or hemiplegic migraine; tension-type headache on 15 or more days per month; or prior history of peptic ulcer disease or gastrointestinal bleeding. Other exclusion criteria included known hypersensitivity to sumatriptan; a previous allergic reaction to prescription as well as to over-the-counter products containing naproxen, or had aspirin, or other nonsteroidal anti-inflammatory/analgesic drugs induce the syndrome of asthma, rhinitis, and nasal polyps; abuse of ergotamine in the 3 months before screening; evidence of alcohol, drug or substance abuse within the previous year; use of a monoamine oxidase inhibitor within 2 weeks before screening. Subjects with a history of sumatriptan succinate tablet or other 5- hydroxytryptamine (5HT1) specific agonist use on more than 3 occasion s and/or had received a prescription for sumatriptan succinate tablets any time prior to screening were also excluded.
**Tazaki 1993a**	Migraine with or without aura according to the International Classification of Headache Disorders. At least one attack per month within the last 3 months. Age from 18 to 65.	Regular use of ergotamines, suspicion of ischaemic heart disease or hypertension that cannot be controlled with serious hypertensives, severely decreased liver or kidney function, pregnancy or potentially pregnant, nursing mothers, the investigating physician.
**Tazaki 1993b**	Migraine without aura or migraine with aura based on the diagnostic criteria for migraine by the International Headache Society. Diagnosed with migraine headaches for at least 1 year. Patients with an attack frequency of 1 to 6 times per month and whose attack severity interfered with work and daily life were included. As a general rule, applicants had to be between 18 and 65 regardless of gender.	Patients who regularly use ergotamine preparations and need to stop using them. Patients with suspected ischemic heart disease or moderate or severe hypertension. Patients with severe liver or renal disorder. Pregnant or possibly pregnant patients and lactating patients. Patients who are judged by the attending physician to be inappropriate for this study.
**Teall 1996**	Men and women 18 to 65 were eligible for entry. Patients must have at least a 6-month history of migraine (with and without aura) as defined by International Headache Society (IHS)-criteria a frequency of one to eight migraine attacks per month that could be distinguished from tension or interval headaches and be in generally good health.	Patients in the following categories were excluded: those with uncontrolled hypertension, a clinically significant electrocardiogram (ECG) abnormality (eg ischemia, myocardial infarction, left bundle block) or significant cardiovascular risk factors, pregnant women or nursing mothers, a history within the prior year or current evidence of drug or alcohol abuse, those prior exposure to rizatriptan and those who had received treatment with any other investigational compound or device within 30 days of the study start. Additionally, patients with a disease or disease history which might have confounded the results or have posed an additional risk were also ineligible.
**Tfelt-Hansen 1995**	Patients (18 to 65 years) were eligible for the study if they met the International Headache Society (IHS) diagnostic criteria for migraine with or without aura. They were required to have a history of migraine more than one year's duration, with two to six attacks per month within the last three months.	Not reported.

**Tfelt-Hansen 1998**	Men and women (18 to 65 years), who met International Headache Society (IHS)-criteria for migraine with or without aura, were enrolled. All patients had at least a 6-month history of migraine, typically experienced one to eight attacks per month which could be distinguished from tension or interval headaches, and were in good health.	Patients were excluded if there was clinical evidence of cardiovascular disease, hypertension, or significant electrocardiogram (ECG) abnormality, or if they had a known history (within 1 year) or current evidence of drug or alcohol abuse. Women who were pregnant or breast-feeding, patients with prior exposure to rizatriptan, those with any contraindication or sensitivity to sumatriptan, or those who had received treatment with any other investigational compound or device within the past 30 days were also excluded.
**Tfelt-Hansen 2006**	Patients were eligible for the study if they were between 18 and 65 years of age, suffered from migraine with or without aura as defined by the 1988 International Headache Society criteria (IHS) for at least a year, had a history of 6 to 12 migraine attacks per year, had the experience that the headache became moderate or severe following a mild phase, were able to differentiate migraine from other headaches and had not treated a migraine with a triptan within the last 6 months.	Patients were excluded if they had uncontrolled hypertension (diastolic blood pressure >95 mmHg or systolic blood pressure >160 mmHg); had cardiovascular disease; suffered from chronic tension-type headache; had ophthalmoplegic, basilar and hemiplegic migraine; or had suspected or confirmed cerebrovascular or cardiovascular disease.
**The Diclofenac-K/Sumatriptan Migraine Study 1999**	Adult patients meeting the International Headache Society diagnostic criteria for migraine with or without aura. Disease duration of at least 1 year and attack frequency of 2 to 6 per month over the past 6 months.	Other types of migraine or non-migrainous interval headaches.
**The Oral Sumatriptan Dose-defining Study 1991**	Migraine history at least 1 year. Moderate or severe migraine without aura or migraine with aura according to International Headache Society.	Aged less than 18 or more than 60. History of drug abuse incl. ergotamine or regularly required narcotic analgesics. Hypertensive or had a history of ischaemic heart disease (IHD). Pregnant or lactating women.
**Toledano 2021**	Patients 18 to 75 years of age with a history of migraine with or without aura for at least one year consistent with the diagnosis criteria of the International Classification of Headache Disorders (ICHD)-3rd edition (beta version) and have experienced between 2 and 8 migraine days in each of the 3 months before screening. Migraine onset before age 50 years was required. A history of migraine typically lasting 4 to 72 hours if untreated or treated unsuccessfully, and migraine episodes separated by at least 48 hours of headache pain-freedom were required.	Patients with neurologically complicated migraine or cluster headaches were excluded. Patients using opioid medications or patients who had a history within the previous 3 years of abuse of any drug were excluded. Patients with a clinically significant hematologic, endocrine, cardiovascular, cerebrovascular, pulmonary, renal, hepatic, gastrointestinal, or neurologic disease; or had medication overuse headache to the investigator's opinion were excluded.
**Tuchman 2006**	Women were eligible for the study if they were aged ≥18 years, had regular menstrual periods and a diagnosis of menstrual migraine headache. Participants were required to have had at least three menstrual migraine headaches of moderate or severe intensity within the previous 3 months, a history of ≤15 non-migraine headaches per month and ≥75% of all menstrual periods associated with migraine.	A medical or psychiatric condition that may interfere with data collection; a history of, symptoms of or significant risk factors for cardiovascular disease; uncontrolled hypertension; a history of basilar, ophthalmoplegic or hemiplegic migraine or any serious neurological condition associated with headache; use of monoamine oxidase A inhibitors in the 2 weeks prior to the study; where the dosage was not stabilised; pregnancy or lactation; a history of poor compliance with treatment regimens.

**Tullo 2010**	Male or female subjects, aged 18 to 65 years, with a current history of migraine with or without aura, according to International Headache Society (IHS) criteria, and with at least one migraine attack per month for 6 months prior to entering the study, were eligible for participation in the study.	Patients with uncontrolled hypertension, cardiac, vascular, liver and renal impairment, or any other severe or disabling medical condition could not be enrolled. Individuals with history of alcohol or analgesic or psychotropic drug abuse, known hypersensitivity to study drugs, previous inadequate response to at least two triptans, currently using ergotamine (and its derivatives) or monoamine oxidase (MAO)-inhibitors, or suffering from headaches that have been lasting for >6 days, were excluded as well. Pregnant women, breastfeeding mothers, and women with childbearing potential having a positive or missing pregnancy test were not eligible.
**Visser 1996a**	Men and women between 18 and 55 years of age. At least 6 months history of migraine with or without aura, and typically 8 or fewer migraine attacks per month.	History, clinical evidence or electrocardiogram (ECG) that was suggestive of a significant cardiovascular disease; hypertension (at screening: resting systolic blood pressure >160 mm Hg or diastolic blood pressure >95 mm Hg); or renal, gastrointestinal, pulmonary, hepatic, endocrine, neurological (other than migraine), or other systemic disease. Women who were pregnant, breast-feeding, or not using adequate contraceptive precautions were excluded, as were patients who had a history of alcohol or other drug abuse, who had previously experienced an adverse reaction to sumatriptan, or who were treated with an investigational device or compound within 1 month prior to the study.
**Visser 1996b**	Men and women between ages 18 and 55 years were included if they suffered from migraine with or without aura as defined by the International Headache Society criteria. Patients were eligible for inclusion if they had migraine for 21 years, with age of onset <40 years and an average of no more than six attacks per month.	Patients were excluded if they suffered from interval headaches that they could not distinguish from migraine without aura and if they regularly vomited early in their migraine attack. We also excluded patients who used prophylactic treatment in the month before the study day or regularly required or abused other drugs or alcohol. Patients were excluded if they had an electrocardiogram (ECG) or personal or strong family history suggestive of coronary artery disease; peripheral vascular disease; hypertension (known blood pressure ≥150/90 mm Hg or 170/100 mm Hg just before drug administration); known hypercholesterolemia (>6.5 mmol/L); renal, hepatic, or psychiatric disorders; as well as women who were pregnant, not using adequate contraceptive measures, or breast feeding. In addition, patients were excluded if they had participated in two or more clinical trials involving different experimental drugs within the previous 3 years. On the study day, we excluded patients who had experienced a previous migraine attack in the last 48 hours or had taken ergot-containing drugs or sumatriptan in the last 48 hours or analgesics in the last 6 hours.
**Voss 2016**	More than 1 year history of migraine with or without aura as defined by International Headache Society (IHS) criteria 1.1 and/or 1.2 Migraines typically last between 4 to 72 hours, if untreated ≥ 2 and ≤ 8 moderate or severe migraine attacks per month in each of the two months prior to screening. Male, female who is not of reproductive potential, or female of reproductive potential with a screening serum β-human chorionic gonadotropin (β-hCG) level consistent with a not-pregnant state, and who agrees to use acceptable contraception.	Pregnant or breast-feeding, or is a female expecting to conceive within the projected duration of study participation. Participant has difficulty distinguishing his/her migraine attacks from tension-type headaches. History of predominantly mild migraine attacks or migraines that usually resolve spontaneously in less than two hours. More than 15 headache-days per month or has taken medication for acute headache on more than 10 days per month in any of the three months prior to screening. Basilar-type or hemiplegic migraine headache. >50 years old at age of migraine onset. Taking migraine prophylactic medication where the prescribed daily dose has changed during the 3 months prior to screening and will not be changed during the study. Taking a proton pump inhibitor (PPI) or a histamine receptor 2 (H_2) blocker on a daily or near daily basis (>3 days per week). Taking the following medications from 1 month prior to screening through study period: potent cytochrome P450 (CYP) 3A4 inhibitors (e.g., cyclosporine, itraconazole, ketoconazole, fluconazole, erythromycin, clarithromycin, nefazodone, telithromycin, cimetidine, quinine, diltiazem, verapamil, and human immunodeficiency virus [HIV] protease inhibitors), moderate or marked CYP3A4 inducers (e.g., rifampicin, rifabutin, barbiturates [e.g., phenobarbital and primidone], systemic

		glucocorticoids, nevirapine, efavirenz, pioglitazone, carbamazepine, phenytoin, and St. John's wort), or drugs with narrow therapeutic margins and potential for drug interactions in the CYP2C family (e.g., warfarin). Participant is unable to refrain from consumption of grapefruit or grapefruit juice during study. History of hypersensitivity to, or has experienced a serious adverse event in response to 3 or more classes of drugs (prescription and over-the-counter). Clinical or laboratory evidence of uncontrolled diabetes, human immunodeficiency virus (HIV) disease, or significant pulmonary, renal, hepatic, endocrine, or other systemic disease. Other confounding pain syndromes, psychiatric conditions such as uncontrolled major depression, dementia or significant neurological disorders other than migraine. Patients who are currently being treated with non-prohibited medication for depression and symptoms are well controlled are eligible to participate. Participant is at imminent risk of self-harm. History of malignancy ≤ 5 years prior to study, except for adequately treated basal cell or squamous cell skin cancer, or in situ cervical cancer. History of gastric or small intestinal surgery (including gastric bypass surgery or banding), or presence of a disease that causes malabsorption. Participant has recent history (within the last year) of drug or alcohol abuse or dependence or is a user of recreational or illicit drugs. Participant is legally or mentally incapacitated. Donation of blood products or phlebotomy of >300 ml within 8 weeks of study, or intent to donate blood products or receive blood products within 30 days of screening and throughout study. Intent to donate eggs or sperm within the projected duration of the study. Current participation in or participation within 30 days of screening in a study with an investigational compound or device. Previous exposure to MK-0974 and/or MK-3207. Use within the past 2 months of an opioid- or barbiturate-containing analgesic for migraine relief. Inpatient or emergency department treatment of an acute migraine attack within the past 2 months.
**Wentz 2008**	Male and non-pregnant female subjects, aged 18 to 65 years with at least a 6-month history of migraine with or without aura according to the 1988 International Headache Society (IHS) criteria, were eligible for inclusion. The population was defined in accordance with the IHS guidelines for the conduct of acute migraine clinical trials. Subjects were required to have experienced one to six moderate or severe migraine episodes per month for three consecutive months prior to screening.	Any subject experiencing headache for ≥15 days/month in any of the 3 months prior to screening was excluded. Enrolled subjects were otherwise healthy and free from any clinically significant illness or disease. Medications prohibited during the study included migraine prophylaxis agents (within 1 month prior to study entry and during the study); acute migraine treatments (within 24 h prior to study drug treatment); gastro-protective agents (within 1 week prior to study entry); proton pump inhibitors, H2-blockers or antacids at prescribed doses; digitalis preparations, anticoagulants, anti-platelet aggregation agents (except low dose aspirin 325 mg/day) or digoxin; antineoplastics; human immunodeficiency virus [HIV] suppressives; a combination of diuretic with either an angiotensin-converting enzyme inhibitor or an angiotensin receptor blocker; and any medications known to be an inhibitor or inducer of CYP3A4.
**Winner 2003a**	Males and nonpregnant females using adequate contraception were eligible if they were between 18 and 65 years of age (inclusive), had at least a 1-year history of migraine with or without aura as defined by the 1988 International Headache Society criteria, had 1 to 6 migraines monthly during the 2 months preceding the screening visit, and had a history of moderate to severe migraine pain preceded by a mild pain phase.	Subjects were excluded if they had uncontrolled hypertension (sitting diastolic blood pressure ≥95 mmHg or systolic blood pressure ≥160 mmHg) at screening; a history of epilepsy; confirmed or suspected cardiovascular, cerebrovascular, peripheral vascular, or ischemic heart or abdominal disease or Raynaud's syndrome; impaired hepatic or renal function; basilar or hemiplegic migraine or cluster headache; or evidence within 3 months of screening of a rebound headache pattern caused by ergotamines or analgesics. Other exclusion criteria included use of a monoamine oxidase inhibitor within 2 weeks before screening. Pre-study experience with triptan therapy was not an exclusion criterion. Subjects were also excluded: if there was evidence within the year prior to screening of alcohol or substance abuse that in

		the investigator's judgment could interfere with the conduct of the study, the subject's cooperation, or the evaluation and interpretation of results of the study; if they had any concurrent medical condition that may have affected the interpretation of efficacy and safety data or that otherwise contraindicated the subject's participation or if they had participated in an investigational drug trial within the 4 weeks prior to screening or planned to participate in another study at any time during the current study.
**Winner 2003b**	Males and nonpregnant females using adequate contraception were eligible if they were between 18 and 65 years of age (inclusive), had at least a 1-year history of migraine with or without aura as defined by the 1988 International Headache Society criteria, had 1 to 6 migraines monthly during the 2 months preceding the screening visit, and had a history of moderate to severe migraine pain preceded by a mild pain phase.	Subjects were excluded if they had uncontrolled hypertension (sitting diastolic blood pressure ≥95 mmHg or systolic blood pressure ≥160 mmHg) at screening; a history of epilepsy; confirmed or suspected cardiovascular, cerebrovascular, peripheral vascular, or ischemic heart or abdominal disease or Raynaud's syndrome; impaired hepatic or renal function; basilar or hemiplegic migraine or cluster headache; or evidence within 3 months of screening of a rebound headache pattern caused by ergotamines or analgesics. Other exclusion criteria included use of a monoamine oxidase inhibitor within 2 weeks before screening. Pre-study experience with triptan therapy was not an exclusion criterion. Subjects were also excluded: if there was evidence within the year prior to screening of alcohol or substance abuse that in the investigator's judgment could interfere with the conduct of the study, the subject's cooperation, or the evaluation and interpretation of results of the study; if they had any concurrent medical condition that may have affected the interpretation of efficacy and safety data or that otherwise contraindicated the subject's participation or if they had participated in an investigational drug trial within the 4 weeks prior to screening or planned to participate in another study at any time during the current study.
**Yu 2023**	Subject has at least 1 year history of migraines (with or without aura), consistent with a diagnosis according to the International Classification of Headache Disorder, 3rd Edition, Beta version including the following: Migraine attacks present for more than 1 year with the age of onset prior to 50 years of age. Migraine attacks, on average, lasting about 4 to 72 hours if untreated. Not more than 8 attacks of moderate to severe intensity per month within the last 3 months. Consistent migraine headaches of at least 2 migraine headache attacks of moderate or severe intensity in each of the 3 months prior to the Screening Visit and maintains this requirement during the Screening period. Less than 15 days with headache (migraine or non-migraine) per month in each of the 3 months prior to the Screening Visit and maintains this requirement during the Screening Period. Subjects on prophylactic migraine medication are permitted to remain on therapy provided they have been on a stable dose for at least 3 months prior to screening visit and the dose is not expected to change during the course of the study. Subjects with contraindications for use of triptans may be included provided they meet all other study entry criteria.	Subject with a history of human immunodeficiency virus (HIV) disease. Subject history with current evidence of uncontrolled, unstable or recently diagnosed cardiovascular disease, such as ischemic heart disease, coronary artery vasospasm, and cerebral ischemia. subjects with Myocardial Infarction (MI), Acute Coronary Syndrome (ACS), Percutaneous Coronary Intervention (PCI), cardiac surgery, stroke or transient ischemic attack (TIA) during the 6 months prior to screening. Uncontrolled hypertension (high blood pressure), or uncontrolled diabetes (however subjects can be included who have stable hypertension and/or diabetes for at least 3 months prior to being enrolled). Subject has a current diagnosis of major depression, other pain syndromes, psychiatric conditions (e.g., schizophrenia), dementia, or significant neurological disorders (other than migraine) that, in the Investigator's opinion might interfere with study assessments. Subject has a history of gastric, or small intestinal surgery (including Gastric Bypass, Gastric Banding, Gastric Sleeve, Gastric Balloon, etc.), or has disease that causes malabsorption. The subject has a history of current or evidence of any significant and/ or unstable medical conditions (e.g., history of congenital heart disease or arrhythmia, known suspected infection, hepatitis B or C, or cancer) that, in the investigator's opinion, would expose them to undue risk of a significant adverse event (AE) or interfere with assessments of safety or efficacy during the course of the trial. History of, treatment for, or evidence of, alcohol or drug abuse within the past 12 months or subjects who have met DSM-V criteria for any significant substance use disorder within the past 12 months from the date of the screening visit. Subjects are excluded if they have previously participated in any study of rimegepant or other experimental calcitonin gene-related peptide (CGRP)-antagonist study, or have been prescribed CGRP-antibodies within the last 6 months. Participation in any other investigational clinical trial while participating in this clinical trial.

Table S5. CINeMA (certainty of evidence) – Pain freedom at 2 hours

Comparison	Studies (n)	Within-study bias	Reporting bias	Indirectness	Imprecision	Heterogeneity	Incoherence	Confidence rating
placebo:rimegepant	5	No concerns	Low risk	No concerns	No concerns	No concerns	No concerns	High
acetylsalicylic acid:placebo	3	Some concerns	Some concerns	No concerns	No concerns	No concerns	No concerns	Moderate
almotriptan:placebo	5	Some concerns	Low risk	No concerns	No concerns	No concerns	No concerns	Moderate
eletriptan:naratriptan	1	Some concerns	Some concerns	No concerns	No concerns	No concerns	No concerns	Moderate
naproxen sodium:placebo	4	Some concerns	Low risk	No concerns	No concerns	No concerns	No concerns	Moderate
naratriptan:rizatriptan	1	Some concerns	Low risk	No concerns	No concerns	No concerns	No concerns	Moderate
naratriptan:sumatriptan	1	Some concerns	Some concerns	No concerns	No concerns	No concerns	No concerns	Moderate
paracetamol:placebo	4	Some concerns	Low risk	No concerns	No concerns	No concerns	No concerns	Moderate
paracetamol:rizatriptan	1	Some concerns	Some concerns	No concerns	No concerns	No concerns	No concerns	Moderate
placebo:rizatriptan	17	Some concerns	Some concerns	No concerns	No concerns	No concerns	No concerns	Moderate
placebo:sumatriptan	40	Some concerns	Some concerns	No concerns	No concerns	No concerns	No concerns	Moderate
placebo:zolmitriptan	12	Some concerns	Low risk	No concerns	No concerns	No concerns	No concerns	Moderate
rimegepant:sumatriptan	1	No concerns	Low risk	No concerns	No concerns	Some concerns	No concerns	Moderate
acetylsalicylic acid:eletriptan	0	Some concerns	Low risk	No concerns	No concerns	Some concerns	No concerns	Moderate
almotriptan:sumatriptan	2	Some concerns	Low risk	No concerns	No concerns	Major concerns	No concerns	Low
almotriptan:zolmitriptan	1	Some concerns	Low risk	No concerns	Some concerns	Some concerns	No concerns	Low
diclofenac potassium:placebo	2	Some concerns	Low risk	Some concerns	No concerns	No concerns	No concerns	Low
eletriptan:placebo	14	Some concerns	High risk	No concerns	No concerns	No concerns	No concerns	Low
naproxen sodium:sumatriptan	3	Some concerns	Low risk	No concerns	No concerns	Major concerns	No concerns	Low
placebo:ubrogepant	4	No concerns	Low risk	Major concerns	No concerns	No concerns	No concerns	Low
rizatriptan:sumatriptan	3	Some concerns	Some concerns	No concerns	Some concerns	Some concerns	No concerns	Low
rizatriptan:zolmitriptan	1	Some concerns	Some concerns	No concerns	Some concerns	Some concerns	No concerns	Low
acetylsalicylic acid:rizatriptan	0	Some concerns	Low risk	No concerns	No concerns	Major concerns	No concerns	Low
almotriptan:eletriptan	0	Some concerns	High risk	No concerns	No concerns	No concerns	No concerns	Low

celecoxib:rizatriptan	0	Some concerns	Some concerns	Some concerns	No concerns	No concerns	No concerns	Low
celecoxib:sumatriptan	0	Some concerns	Low risk	Some concerns	No concerns	Some concerns	No concerns	Low
eletriptan:frovatriptan	0	Some concerns	Low risk	No concerns	No concerns	Major concerns	No concerns	Low
eletriptan:ibuprofen	0	Some concerns	Low risk	Some concerns	No concerns	No concerns	No concerns	Low
eletriptan:naproxen sodium	0	Some concerns	High risk	No concerns	No concerns	No concerns	No concerns	Low
eletriptan:paracetamol	0	Some concerns	High risk	No concerns	No concerns	No concerns	No concerns	Low
eletriptan:rimegepant	0	Some concerns	High risk	No concerns	No concerns	No concerns	No concerns	Low
frovatriptan:naratriptan	0	Some concerns	Low risk	No concerns	No concerns	Major concerns	No concerns	Low
ibuprofen:rizatriptan	0	Some concerns	Low risk	Some concerns	No concerns	Some concerns	No concerns	Low
naproxen sodium:zolmitriptan	0	Some concerns	Low risk	No concerns	Some concerns	Some concerns	No concerns	Low
naratriptan:zolmitriptan	0	Some concerns	High risk	No concerns	No concerns	No concerns	No concerns	Low
paracetamol:rimegepant	0	No concerns	Low risk	No concerns	Major concerns	No concerns	No concerns	Low
paracetamol:sumatriptan	0	Some concerns	Low risk	No concerns	No concerns	Major concerns	No concerns	Low
paracetamol:zolmitriptan	0	Some concerns	Low risk	No concerns	No concerns	Major concerns	No concerns	Low
rimegepant:rizatriptan	0	Some concerns	High risk	No concerns	No concerns	No concerns	No concerns	Low
rimegepant:zolmitriptan	0	Some concerns	Low risk	No concerns	No concerns	Major concerns	No concerns	Low
acetylsalicylic acid:sumatriptan	1	Some concerns	Some concerns	No concerns	Major concerns	No concerns	No concerns	Very low
celecoxib:placebo	3	No concerns	Low risk	Some concerns	No concerns	Major concerns	No concerns	Very low
eletriptan:sumatriptan	3	Some concerns	High risk	No concerns	No concerns	Major concerns	No concerns	Very low
eletriptan:zolmitriptan	1	Some concerns	Some concerns	No concerns	No concerns	Major concerns	No concerns	Very low
frovatriptan:placebo	5	Some concerns	Some concerns	No concerns	No concerns	No concerns	Major concerns	Very low
frovatriptan:sumatriptan	1	Some concerns	Some concerns	No concerns	Major concerns	No concerns	Major concerns	Very low
ibuprofen:placebo	3	Some concerns	High risk	Some concerns	No concerns	No concerns	No concerns	Very low
lasmiditan:placebo	5	Major concerns	Low risk	Major concerns	No concerns	No concerns	No concerns	Very low
naratriptan:placebo	5	Some concerns	Some concerns	No concerns	No concerns	Major concerns	No concerns	Very low
phenazone:placebo	1	Some concerns	Low risk	Major concerns	No concerns	Major concerns	No concerns	Very low
sumatriptan:zolmitriptan	2	Some concerns	Some concerns	No concerns	Major concerns	No concerns	No concerns	Very low

acetylsalicylic acid:almotriptan	0	Some concerns	High risk	No concerns	Major concerns	No concerns	No concerns	Very low
acetylsalicylic acid:celecoxib	0	Some concerns	High risk	Some concerns	Major concerns	No concerns	No concerns	Very low
acetylsalicylic acid:diclofenac potassium	0	Some concerns	High risk	Some concerns	Major concerns	No concerns	No concerns	Very low
acetylsalicylic acid:frovatriptan	0	Some concerns	Low risk	No concerns	Major concerns	No concerns	No concerns	Very low
acetylsalicylic acid:ibuprofen	0	Some concerns	Low risk	Some concerns	Major concerns	No concerns	No concerns	Very low
acetylsalicylic acid:lasmiditan	0	Some concerns	High risk	Some concerns	Major concerns	No concerns	No concerns	Very low
acetylsalicylic acid:naproxen sodium	0	Some concerns	High risk	No concerns	Major concerns	No concerns	No concerns	Very low
acetylsalicylic acid:naratriptan	0	Some concerns	Low risk	No concerns	Major concerns	No concerns	No concerns	Very low
acetylsalicylic acid:paracetamol	0	Some concerns	High risk	No concerns	Major concerns	No concerns	No concerns	Very low
acetylsalicylic acid:phenazone	0	Some concerns	High risk	Some concerns	Major concerns	No concerns	No concerns	Very low
acetylsalicylic acid:rimegepant	0	Some concerns	High risk	No concerns	Major concerns	No concerns	No concerns	Very low
acetylsalicylic acid:ubrogepant	0	Some concerns	High risk	Some concerns	Major concerns	No concerns	No concerns	Very low
acetylsalicylic acid:zolmitriptan	0	Some concerns	High risk	No concerns	Major concerns	No concerns	No concerns	Very low
almotriptan:celecoxib	0	Some concerns	Low risk	Some concerns	Major concerns	No concerns	No concerns	Very low
almotriptan:diclofenac potassium	0	Some concerns	Low risk	Some concerns	Major concerns	No concerns	No concerns	Very low
almotriptan:frovatriptan	0	Some concerns	High risk	No concerns	Major concerns	No concerns	No concerns	Very low
almotriptan:ibuprofen	0	Some concerns	High risk	Some concerns	Major concerns	No concerns	No concerns	Very low
almotriptan:lasmiditan	0	Some concerns	Low risk	Some concerns	Major concerns	No concerns	No concerns	Very low
almotriptan:naproxen sodium	0	Some concerns	Low risk	No concerns	Major concerns	No concerns	No concerns	Very low
almotriptan:naratriptan	0	Some concerns	High risk	No concerns	No concerns	Major concerns	No concerns	Very low
almotriptan:paracetamol	0	Some concerns	Low risk	No concerns	Major concerns	No concerns	No concerns	Very low
almotriptan:phenazone	0	Some concerns	Low risk	Major concerns	Major concerns	No concerns	No concerns	Very low
almotriptan:rimegepant	0	Some concerns	Low risk	No concerns	Major concerns	No concerns	No concerns	Very low
almotriptan:rizatriptan	0	Some concerns	High risk	No concerns	No concerns	Major concerns	No concerns	Very low
almotriptan:ubrogepant	0	Some concerns	Low risk	Major concerns	Major concerns	No concerns	No concerns	Very low
celecoxib:diclofenac potassium	0	No concerns	Low risk	Some concerns	Major concerns	No concerns	No concerns	Very low
celecoxib:eletriptan	0	Some concerns	High risk	Some concerns	No concerns	No concerns	No concerns	Very low

celecoxib:frovatriptan	0	Some concerns	High risk	Some concerns	Some concerns	Some concerns	No concerns	Very low
celecoxib:ibuprofen	0	Some concerns	High risk	Some concerns	Major concerns	No concerns	No concerns	Very low
celecoxib:lasmiditan	0	Some concerns	Low risk	Some concerns	Major concerns	No concerns	No concerns	Very low
celecoxib:naproxen sodium	0	Some concerns	Low risk	Some concerns	Major concerns	No concerns	No concerns	Very low
celecoxib:naratriptan	0	Some concerns	Some concerns	Some concerns	Major concerns	No concerns	No concerns	Very low
celecoxib:paracetamol	0	No concerns	Low risk	Some concerns	Major concerns	No concerns	No concerns	Very low
celecoxib:phenazone	0	Some concerns	Low risk	Major concerns	Major concerns	No concerns	No concerns	Very low
celecoxib:rimegepant	0	No concerns	Low risk	Some concerns	Major concerns	No concerns	No concerns	Very low
celecoxib:ubrogepant	0	No concerns	Low risk	Some concerns	Major concerns	No concerns	No concerns	Very low
celecoxib:zolmitriptan	0	Some concerns	Low risk	Some concerns	No concerns	Major concerns	No concerns	Very low
diclofenac potassium:eletriptan	0	Some concerns	Some concerns	Some concerns	Major concerns	No concerns	No concerns	Very low
diclofenac potassium:frovatriptan	0	Some concerns	Some concerns	Some concerns	Major concerns	No concerns	No concerns	Very low
diclofenac potassium:ibuprofen	0	Some concerns	Some concerns	Some concerns	Major concerns	No concerns	No concerns	Very low
diclofenac potassium:lasmiditan	0	Some concerns	Low risk	Some concerns	Major concerns	No concerns	No concerns	Very low
diclofenac potassium:naproxen sodium	0	Some concerns	Low risk	Some concerns	Major concerns	No concerns	No concerns	Very low
diclofenac potassium:naratriptan	0	Some concerns	High risk	Some concerns	Major concerns	No concerns	No concerns	Very low
diclofenac potassium:paracetamol	0	Some concerns	Low risk	Some concerns	Major concerns	No concerns	No concerns	Very low
diclofenac potassium:phenazone	0	Some concerns	Low risk	Major concerns	Major concerns	No concerns	No concerns	Very low
diclofenac potassium:rimegepant	0	No concerns	Low risk	Some concerns	Major concerns	No concerns	No concerns	Very low
diclofenac potassium:rizatriptan	0	Some concerns	High risk	Some concerns	Major concerns	No concerns	No concerns	Very low
diclofenac potassium:sumatriptan	0	Some concerns	Low risk	Some concerns	Major concerns	No concerns	No concerns	Very low
diclofenac potassium:ubrogepant	0	No concerns	Low risk	Some concerns	Major concerns	No concerns	No concerns	Very low
diclofenac potassium:zolmitriptan	0	Some concerns	Low risk	Some concerns	Major concerns	No concerns	No concerns	Very low
eletriptan:lasmiditan	0	Some concerns	High risk	Some concerns	No concerns	No concerns	No concerns	Very low
eletriptan:phenazone	0	Some concerns	High risk	Major concerns	Major concerns	No concerns	No concerns	Very low
eletriptan:rizatriptan	0	Some concerns	Low risk	No concerns	Major concerns	No concerns	No concerns	Very low
eletriptan:ubrogepant	0	Some concerns	High risk	Major concerns	No concerns	No concerns	No concerns	Very low

frovatriptan:ibuprofen	0	Some concerns	Low risk	Some concerns	Major concerns	No concerns	No concerns	Very low
frovatriptan:lasmiditan	0	Some concerns	High risk	Some concerns	Major concerns	No concerns	No concerns	Very low
frovatriptan:naproxen sodium	0	Some concerns	High risk	No concerns	Major concerns	No concerns	No concerns	Very low
frovatriptan:paracetamol	0	Some concerns	High risk	No concerns	Major concerns	No concerns	No concerns	Very low
frovatriptan:phenazone	0	Some concerns	High risk	Some concerns	Major concerns	No concerns	No concerns	Very low
frovatriptan:rimegepant	0	Some concerns	High risk	No concerns	No concerns	Major concerns	No concerns	Very low
frovatriptan:rizatriptan	0	Some concerns	Low risk	No concerns	Major concerns	No concerns	No concerns	Very low
frovatriptan:ubrogepant	0	Some concerns	High risk	Some concerns	Major concerns	No concerns	No concerns	Very low
frovatriptan:zolmitriptan	0	Some concerns	High risk	No concerns	Major concerns	No concerns	No concerns	Very low
ibuprofen:lasmiditan	0	Some concerns	High risk	Major concerns	Major concerns	No concerns	No concerns	Very low
ibuprofen:naproxen sodium	0	Some concerns	High risk	Some concerns	Major concerns	No concerns	No concerns	Very low
ibuprofen:naratriptan	0	Some concerns	Low risk	Some concerns	Major concerns	No concerns	No concerns	Very low
ibuprofen:paracetamol	0	Some concerns	High risk	Some concerns	Major concerns	No concerns	No concerns	Very low
ibuprofen:phenazone	0	Some concerns	High risk	Major concerns	Major concerns	No concerns	No concerns	Very low
ibuprofen:rimegepant	0	Some concerns	High risk	Some concerns	Major concerns	No concerns	No concerns	Very low
ibuprofen:sumatriptan	0	Some concerns	High risk	Some concerns	No concerns	Major concerns	No concerns	Very low
ibuprofen:ubrogepant	0	Some concerns	High risk	Major concerns	Major concerns	No concerns	No concerns	Very low
ibuprofen:zolmitriptan	0	Some concerns	High risk	Some concerns	No concerns	Major concerns	No concerns	Very low
lasmiditan:naproxen sodium	0	Some concerns	Low risk	Some concerns	Major concerns	No concerns	No concerns	Very low
lasmiditan:naratriptan	0	Some concerns	High risk	Major concerns	Major concerns	No concerns	No concerns	Very low
lasmiditan:paracetamol	0	Some concerns	Low risk	Some concerns	Major concerns	No concerns	No concerns	Very low
lasmiditan:phenazone	0	Some concerns	Low risk	Major concerns	Major concerns	No concerns	No concerns	Very low
lasmiditan:rimegepant	0	Some concerns	Low risk	Some concerns	Major concerns	No concerns	No concerns	Very low
lasmiditan:rizatriptan	0	Some concerns	High risk	Some concerns	No concerns	Some concerns	No concerns	Very low
lasmiditan:sumatriptan	0	Some concerns	Low risk	Major concerns	No concerns	Major concerns	No concerns	Very low
lasmiditan:ubrogepant	0	Some concerns	Low risk	Major concerns	Major concerns	No concerns	No concerns	Very low
lasmiditan:zolmitriptan	0	Some concerns	Low risk	Some concerns	No concerns	Major concerns	No concerns	Very low

naproxen sodium:naratriptan	0	Some concerns	High risk	No concerns	Major concerns	No concerns	No concerns	Very low
naproxen sodium:paracetamol	0	Some concerns	Low risk	No concerns	Major concerns	No concerns	No concerns	Very low
naproxen sodium:phenazone	0	Some concerns	Low risk	Major concerns	Major concerns	No concerns	No concerns	Very low
naproxen sodium:rimegepant	0	Some concerns	Low risk	No concerns	Major concerns	No concerns	No concerns	Very low
naproxen sodium:rizatriptan	0	Some concerns	High risk	No concerns	No concerns	Major concerns	No concerns	Very low
naproxen sodium:ubrogepant	0	Some concerns	Low risk	Some concerns	Major concerns	No concerns	No concerns	Very low
naratriptan:paracetamol	0	Some concerns	High risk	No concerns	Major concerns	No concerns	No concerns	Very low
naratriptan:phenazone	0	Some concerns	High risk	Major concerns	Major concerns	No concerns	No concerns	Very low
naratriptan:rimegepant	0	Some concerns	High risk	No concerns	Major concerns	No concerns	No concerns	Very low
naratriptan:ubrogepant	0	Some concerns	High risk	Major concerns	Major concerns	No concerns	No concerns	Very low
paracetamol:phenazone	0	Some concerns	Low risk	Major concerns	Major concerns	No concerns	No concerns	Very low
paracetamol:ubrogepant	0	No concerns	Low risk	Some concerns	Major concerns	No concerns	No concerns	Very low
phenazone:rimegepant	0	Some concerns	Low risk	Some concerns	Major concerns	No concerns	No concerns	Very low
phenazone:rizatriptan	0	Some concerns	High risk	Major concerns	Major concerns	No concerns	No concerns	Very low
phenazone:sumatriptan	0	Some concerns	Low risk	Major concerns	Major concerns	No concerns	No concerns	Very low
phenazone:ubrogepant	0	Some concerns	Low risk	Major concerns	Major concerns	No concerns	No concerns	Very low
phenazone:zolmitriptan	0	Some concerns	Low risk	Major concerns	Major concerns	No concerns	No concerns	Very low
rimegepant:ubrogepant	0	No concerns	Low risk	Some concerns	Major concerns	No concerns	No concerns	Very low
rizatriptan:ubrogepant	0	Some concerns	High risk	Major concerns	No concerns	No concerns	No concerns	Very low
sumatriptan:ubrogepant	0	Some concerns	Low risk	Major concerns	No concerns	Major concerns	No concerns	Very low
ubrogepant:zolmitriptan	0	Some concerns	Low risk	Some concerns	No concerns	Major concerns	No concerns	Very low

Table S6. CINeMA (certainty of evidence) – Sustained pain freedom from 2 to 24 hours

Comparison	Studies (n)	Within-study bias	Reporting bias	Indirectness	Imprecision	Heterogeneity	Incoherence	Confidence rating
placebo:rimegepant	5	No concerns	Low risk	No concerns	No concerns	No concerns	No concerns	High
almotriptan:placebo	5	Some concerns	Low risk	No concerns	No concerns	No concerns	No concerns	Moderate
naproxen sodium:placebo	4	Some concerns	Low risk	No concerns	No concerns	No concerns	No concerns	Moderate
placebo:sumatriptan	17	Some concerns	Low risk	No concerns	No concerns	No concerns	No concerns	Moderate
placebo:zolmitriptan	3	Some concerns	Low risk	No concerns	No concerns	No concerns	No concerns	Moderate
eletriptan:naratriptan	1	Some concerns	Low risk	No concerns	No concerns	No concerns	Some concerns	Low
eletriptan:placebo	7	Some concerns	High risk	No concerns	No concerns	No concerns	No concerns	Low
naproxen sodium:sumatriptan	3	Some concerns	Low risk	No concerns	Some concerns	Some concerns	No concerns	Low
placebo:rizatriptan	8	Some concerns	Some concerns	Some concerns	No concerns	No concerns	No concerns	Low
rimegepant:sumatriptan	1	No concerns	Low risk	No concerns	Major concerns	No concerns	No concerns	Low
almotriptan:sumatriptan	1	Some concerns	Low risk	No concerns	Major concerns	No concerns	No concerns	Very low
almotriptan:zolmitriptan	1	Some concerns	Low risk	No concerns	Major concerns	No concerns	No concerns	Very low
celecoxib:placebo	2	Some concerns	Low risk	Some concerns	No concerns	Major concerns	Major concerns	Very low
diclofenac potassium:placebo	1	Some concerns	Low risk	Some concerns	No concerns	No concerns	Major concerns	Very low
eletriptan:sumatriptan	1	Some concerns	High risk	No concerns	No concerns	Major concerns	Major concerns	Very low
eletriptan:zolmitriptan	1	Some concerns	High risk	No concerns	No concerns	Major concerns	No concerns	Very low
ibuprofen:placebo	1	Some concerns	Some concerns	Some concerns	No concerns	No concerns	Major concerns	Very low
lasmiditan:placebo	4	Major concerns	Low risk	Major concerns	No concerns	No concerns	Major concerns	Very low
naratriptan:placebo	1	Some concerns	Some concerns	No concerns	Major concerns	No concerns	Major concerns	Very low
paracetamol:placebo	2	Some concerns	Some concerns	Some concerns	Major concerns	No concerns	No concerns	Very low
paracetamol:rizatriptan	1	Some concerns	Some concerns	Some concerns	Major concerns	No concerns	No concerns	Very low
placebo:ubrogepant	4	Some concerns	Low risk	Major concerns	No concerns	No concerns	Major concerns	Very low
rizatriptan:zolmitriptan	1	Some concerns	Some concerns	No concerns	Major concerns	No concerns	No concerns	Very low

sumatriptan:zolmitriptan	1	Some concerns	Low risk	No concerns	Major concerns	No concerns	No concerns	Very low
almotriptan:celecoxib	0	Some concerns	Low risk	Some concerns	Major concerns	No concerns	Major concerns	Very low
almotriptan:diclofenac potassium	0	Some concerns	Low risk	Some concerns	Major concerns	No concerns	Major concerns	Very low
almotriptan:eletriptan	0	Some concerns	High risk	No concerns	No concerns	Major concerns	Major concerns	Very low
almotriptan:ibuprofen	0	Some concerns	High risk	Some concerns	Major concerns	No concerns	Major concerns	Very low
almotriptan:lasmiditan	0	Some concerns	Low risk	Major concerns	Major concerns	No concerns	Major concerns	Very low
almotriptan:naproxen sodium	0	Some concerns	Low risk	No concerns	Major concerns	No concerns	Major concerns	Very low
almotriptan:naratriptan	0	Some concerns	High risk	No concerns	Major concerns	No concerns	Major concerns	Very low
almotriptan:paracetamol	0	Some concerns	Low risk	Some concerns	Major concerns	No concerns	Major concerns	Very low
almotriptan:rimegepant	0	Some concerns	Low risk	No concerns	Major concerns	No concerns	Major concerns	Very low
almotriptan:rizatriptan	0	Some concerns	High risk	No concerns	Major concerns	No concerns	Major concerns	Very low
almotriptan:ubrogepant	0	Some concerns	Low risk	Major concerns	Major concerns	No concerns	Major concerns	Very low
celecoxib:diclofenac potassium	0	Some concerns	Low risk	Some concerns	Major concerns	No concerns	Major concerns	Very low
celecoxib:eletriptan	0	Some concerns	High risk	Some concerns	No concerns	No concerns	Major concerns	Very low
celecoxib:ibuprofen	0	Some concerns	High risk	Some concerns	No concerns	No concerns	Major concerns	Very low
celecoxib:lasmiditan	0	Some concerns	Low risk	Some concerns	Major concerns	No concerns	Major concerns	Very low
celecoxib:naproxen sodium	0	Some concerns	Low risk	Some concerns	Major concerns	No concerns	Major concerns	Very low
celecoxib:naratriptan	0	Some concerns	High risk	Some concerns	Major concerns	No concerns	Major concerns	Very low
celecoxib:paracetamol	0	Some concerns	Low risk	Some concerns	Major concerns	No concerns	Major concerns	Very low
celecoxib:rimegepant	0	Some concerns	Low risk	Some concerns	Major concerns	No concerns	Major concerns	Very low
celecoxib:rizatriptan	0	Some concerns	High risk	Some concerns	No concerns	Major concerns	Major concerns	Very low
celecoxib:sumatriptan	0	Some concerns	Low risk	Some concerns	No concerns	Major concerns	Major concerns	Very low
celecoxib:ubrogepant	0	Some concerns	Low risk	Some concerns	Major concerns	No concerns	Major concerns	Very low
celecoxib:zolmitriptan	0	Some concerns	Low risk	Some concerns	Some concerns	Some concerns	Major concerns	Very low
diclofenac potassium:eletriptan	0	Some concerns	High risk	Some concerns	Major concerns	No concerns	Major concerns	Very low
diclofenac potassium:ibuprofen	0	Some concerns	High risk	Some concerns	Major concerns	No concerns	Major concerns	Very low
diclofenac potassium:lasmiditan	0	Some concerns	Low risk	Some concerns	Major concerns	No concerns	Major concerns	Very low
diclofenac potassium:naproxen sodium	0	Some concerns	Low risk	Some concerns	Major concerns	No concerns	Major concerns	Very low

diclofenac potassium:naratriptan	0	Some concerns	High risk	Some concerns	Major concerns	No concerns	Major concerns	Very low
diclofenac potassium:paracetamol	0	Some concerns	Low risk	Some concerns	Major concerns	No concerns	Major concerns	Very low
diclofenac potassium:rimegepant	0	Some concerns	Low risk	Some concerns	Major concerns	No concerns	Major concerns	Very low
diclofenac potassium:rizatriptan	0	Some concerns	High risk	Some concerns	Major concerns	No concerns	Major concerns	Very low
diclofenac potassium:sumatriptan	0	Some concerns	Low risk	Some concerns	Major concerns	No concerns	Major concerns	Very low
diclofenac potassium:ubrogepant	0	Some concerns	Low risk	Some concerns	Major concerns	No concerns	Major concerns	Very low
diclofenac potassium:zolmitriptan	0	Some concerns	Low risk	Some concerns	Major concerns	No concerns	Major concerns	Very low
eletriptan:ibuprofen	0	Some concerns	Some concerns	Some concerns	Major concerns	No concerns	Major concerns	Very low
eletriptan:lasmiditan	0	Some concerns	High risk	Some concerns	No concerns	Some concerns	Major concerns	Very low
eletriptan:naproxen sodium	0	Some concerns	High risk	No concerns	No concerns	Some concerns	Major concerns	Very low
eletriptan:paracetamol	0	Some concerns	High risk	Some concerns	No concerns	Major concerns	Major concerns	Very low
eletriptan:rimegepant	0	Some concerns	High risk	No concerns	No concerns	Major concerns	Major concerns	Very low
eletriptan:rizatriptan	0	Some concerns	Low risk	No concerns	Major concerns	No concerns	Major concerns	Very low
eletriptan:ubrogepant	0	Some concerns	High risk	Major concerns	No concerns	No concerns	Major concerns	Very low
ibuprofen:lasmiditan	0	Some concerns	High risk	Some concerns	No concerns	Some concerns	Major concerns	Very low
ibuprofen:naproxen sodium	0	Some concerns	High risk	Some concerns	No concerns	Some concerns	Major concerns	Very low
ibuprofen:naratriptan	0	Some concerns	High risk	Some concerns	No concerns	No concerns	Major concerns	Very low
ibuprofen:paracetamol	0	Some concerns	High risk	Some concerns	No concerns	Some concerns	Major concerns	Very low
ibuprofen:rimegepant	0	Some concerns	High risk	Some concerns	No concerns	Major concerns	Major concerns	Very low
ibuprofen:rizatriptan	0	Some concerns	Some concerns	Some concerns	Major concerns	No concerns	Major concerns	Very low
ibuprofen:sumatriptan	0	Some concerns	High risk	Some concerns	Major concerns	No concerns	Major concerns	Very low
ibuprofen:ubrogepant	0	Some concerns	High risk	Some concerns	No concerns	No concerns	Major concerns	Very low
ibuprofen:zolmitriptan	0	Some concerns	High risk	Some concerns	Major concerns	No concerns	Major concerns	Very low
lasmiditan:naproxen sodium	0	Some concerns	Low risk	Some concerns	Major concerns	No concerns	Major concerns	Very low
lasmiditan:naratriptan	0	Some concerns	High risk	Some concerns	Major concerns	No concerns	Major concerns	Very low
lasmiditan:paracetamol	0	Some concerns	Low risk	Major concerns	Major concerns	No concerns	Major concerns	Very low
lasmiditan:rimegepant	0	Some concerns	Low risk	Some concerns	Major concerns	No concerns	Major concerns	Very low
lasmiditan:rizatriptan	0	Some concerns	High risk	Major concerns	Major concerns	No concerns	Major concerns	Very low

lasmiditan:sumatriptan	0	Some concerns	Low risk	Major concerns	Some concerns	Some concerns	Major concerns	Very low
lasmiditan:ubrogepant	0	Some concerns	Low risk	Major concerns	Major concerns	No concerns	Major concerns	Very low
lasmiditan:zolmitriptan	0	Some concerns	Low risk	Some concerns	Major concerns	No concerns	Major concerns	Very low
naproxen sodium:naratriptan	0	Some concerns	High risk	No concerns	Major concerns	No concerns	Major concerns	Very low
naproxen sodium:paracetamol	0	Some concerns	Low risk	Some concerns	Major concerns	No concerns	Major concerns	Very low
naproxen sodium:rimegepant	0	Some concerns	Low risk	No concerns	Major concerns	No concerns	Major concerns	Very low
naproxen sodium:rizatriptan	0	Some concerns	High risk	No concerns	Major concerns	No concerns	Major concerns	Very low
naproxen sodium:ubrogepant	0	Some concerns	Low risk	Some concerns	Major concerns	No concerns	Major concerns	Very low
naproxen sodium:zolmitriptan	0	Some concerns	Low risk	No concerns	Major concerns	No concerns	Major concerns	Very low
naratriptan:paracetamol	0	Some concerns	High risk	No concerns	Major concerns	No concerns	Major concerns	Very low
naratriptan:rimegepant	0	Some concerns	Some concerns	No concerns	Major concerns	No concerns	Major concerns	Very low
naratriptan:rizatriptan	0	Some concerns	Low risk	No concerns	Major concerns	No concerns	Major concerns	Very low
naratriptan:sumatriptan	0	Some concerns	Some concerns	No concerns	Major concerns	No concerns	Major concerns	Very low
naratriptan:ubrogepant	0	Some concerns	Some concerns	Some concerns	Major concerns	No concerns	Major concerns	Very low
naratriptan:zolmitriptan	0	Some concerns	Some concerns	No concerns	Major concerns	No concerns	Major concerns	Very low
paracetamol:rimegepant	0	No concerns	Low risk	No concerns	Major concerns	No concerns	Major concerns	Very low
paracetamol:sumatriptan	0	Some concerns	Low risk	Some concerns	Major concerns	No concerns	Major concerns	Very low
paracetamol:ubrogepant	0	Some concerns	Low risk	Major concerns	Major concerns	No concerns	Major concerns	Very low
paracetamol:zolmitriptan	0	Some concerns	Low risk	No concerns	Major concerns	No concerns	Major concerns	Very low
rimegepant:rizatriptan	0	Some concerns	Some concerns	No concerns	Major concerns	No concerns	Major concerns	Very low
rimegepant:ubrogepant	0	No concerns	Low risk	Some concerns	Major concerns	No concerns	Major concerns	Very low
rimegepant:zolmitriptan	0	Some concerns	Low risk	No concerns	Major concerns	No concerns	Major concerns	Very low
rizatriptan:sumatriptan	0	Some concerns	Some concerns	Some concerns	Major concerns	No concerns	Major concerns	Very low
rizatriptan:ubrogepant	0	Some concerns	Some concerns	Major concerns	Some concerns	Some concerns	Major concerns	Very low
sumatriptan:ubrogepant	0	Some concerns	Low risk	Major concerns	Some concerns	Some concerns	Major concerns	Very low
ubrogepant:zolmitriptan	0	Some concerns	Low risk	Some concerns	Major concerns	No concerns	Major concerns	Very low

Table S7. Risk of bias – Pain freedom at 2 hours

Author, year	1. Randomization process	2. Deviations from the intended interventions	3. Missing outcome data	4. Measurement of the outcome	5. Selection of the reported result	Overall risk of bias
0462-039 1996	Some concerns	Low	Low	Low	Some concerns	Some concerns
103 UN	Some concerns	Low	Low	Some concerns	Some concerns	High
Ahrens 1999	Low	Low	Some concerns	Low	Some concerns	Some concerns
Ashina 2021	Low	Low	High	Some concerns	Low	High
Barbanti 2012	Low	Low	Low	Low	Low	Low
Bomhof 1999	Low	Low	Low	Low	Some concerns	Some concerns
Brandes 2005	Low	Low	Low	Low	Some concerns	Some concerns
Brandes 2007a	Low	Low	Low	Low	Some concerns	Some concerns
Brandes 2007b	Low	Low	Low	Low	Some concerns	Some concerns
Brauneis 1994	Some concerns	High	High	Low	Some concerns	High
Bussone 2000	Low	High	Low	Low	Some concerns	High
Cady 2006a	Low	Low	Low	Low	Some concerns	Some concerns
Cady 2006b	Low	Low	Low	Low	Some concerns	Some concerns
Cady 2009	Low	Low	Low	Low	Low	Low
Carpay 2004	Low	Low	Low	Low	Low	Low
Croop 2019	Low	Low	Low	Low	Low	Low
Cutler 1995	Some concerns	Low	Low	Low	Some concerns	Some concerns
Dahlöf 1998	Low	Some concerns	Some concerns	Low	Low	Some concerns
Dahlöf 2001	Low	Low	Low	Low	Some concerns	Some concerns
Dahlöf 2009	Low	Low	Low	Low	Some concerns	Some concerns
Diener 2002	Low	Low	Low	Low	Some concerns	Some concerns
Diener 2004a	Low	Low	Low	Low	Some concerns	Some concerns
Diener 2005	Low	Low	Low	Low	Low	Low

Diener 2011	Low	Low	Low	Low	Low	Low
Dodick 2019	Low	Low	Low	Low	Low	Low
Dowson 2002a	Low	Some concerns	Low	Low	Some concerns	Some concerns
Dowson 2002b	Low	Low	Low	Low	Some concerns	Some concerns
Eletriptan Steering Committee in Japan 2002	Low	Low	Some concerns	Low	Some concerns	Some concerns
Ensink 1991	Some concerns	High	High	Low	Some concerns	High
Freitag 2008	Low	Low	Some concerns	Low	Some concerns	Some concerns
Färkkila 2003	Low	Low	High	Low	Some concerns	High
Färkkila 2012	Low	Low	Low	Some concerns	Low	Some concerns
Garcia-Ramos 2003	Low	Low	Low	Low	Some concerns	Some concerns
Geraud 2000	Low	Low	Low	Low	Some concerns	Some concerns
Gijsmant 1997	Low	Low	Low	Low	Some concerns	Some concerns
Goadsby 2000	Low	Low	High	Low	Some concerns	High
Goadsby 2007	Low	Low	Low	Low	Some concerns	Some concerns
Goadsby 2008	Low	Low	Low	Low	Some concerns	Some concerns
Goadsby 2019	Low	Some concerns	Some concerns	Some concerns	Low	High
Goldstein 2006	Low	Low	Low	Low	Some concerns	Some concerns
Gomez-Mancilla 2014	Low	Low	Low	Low	Low	Low
Gruffyd-Jones 2001	Low	Some concerns	Some concerns	Low	Some concerns	Some concerns
Göbel 2004	Low	Low	Low	Low	Some concerns	Some concerns
Ho 2008a	Low	Low	Low	Low	Some concerns	Some concerns
Ho 2008b	Low	Low	Low	Low	Some concerns	Some concerns
Jelinski 2006	Low	Low	Low	Low	Some concerns	Some concerns
Kaniecki 2006	Low	Low	Low	Low	Low	Low
Kellstein 2000	Low	Low	Low	Low	Some concerns	Some concerns
Klapper 2004	Low	Low	Low	Low	Some concerns	Some concerns

Klassen 1997	Low	Low	Low	Low	Some concerns	Some concerns
Kramer 1998	Low	Low	Low	Low	Some concerns	Some concerns
Kuca 2018	Low	Low	High	Some concerns	Low	High
Landy 2004	Low	Low	Low	Low	Low	Low
Lange 2000	Some concerns	Low	Low	Low	Some concerns	Some concerns
Lines 2001	Some concerns	Low	Low	Low	Some concerns	Some concerns
Lipton 2000a	Low	Low	Some concerns	Low	Some concerns	Some concerns
Lipton 2005	Low	Low	Low	Low	Some concerns	Some concerns
Lipton 2010	Low	Low	Low	Low	Some concerns	Some concerns
Lipton 2019a	Low	Low	Low	Low	Low	Low
Lipton 2019b	Low	Low	Low	Low	Low	Low
Lipton 2019c	Low	Low	Low	Low	Low	Low
Lipton 2021	Low	Low	Low	Low	Low	Low
Loder 2005	Low	Low	Low	Low	Some concerns	Some concerns
Marcus 2014	Low	Low	Low	Low	Low	Low
Massiou 2005	Some concerns	Low	Low	Low	Some concerns	Some concerns
Mathew 2003	Low	Low	Some concerns	Low	Some concerns	Some concerns
Mathew 2007	Low	Low	Some concerns	Low	Some concerns	Some concerns
MOMENTUM 2019	Low	High	Low	Low	Low	High
Moon 2010	Low	Low	Low	Low	Some concerns	Some concerns
Munjal 2017	Low	Low	Low	Low	Low	Low
Myllylä 1998	Low	High	High	Low	Some concerns	High
Nappi 1994	Some concerns	Low	Low	Low	Some concerns	Some concerns
NCT00920686 2010	Some concerns	Low	High	Low	Some concerns	High
NCT01248468 2011	Some concerns	Some concerns	Some concerns	Low	Low	High
NCT01657370 2012	Low	Low	Low	Low	Low	Low
NCT01986270 1998	Some concerns	Low	Some concerns	Low	Some concerns	High

NCT03235479 2018	Low	Low	Low	Low	Low	Low
Nett 2003	Low	Low	Low	Low	Low	Low
Pascual 2000a	Low	Low	Low	Low	Some concerns	Some concerns
Pascual 2000b	Low	Low	Low	Low	Some concerns	Some concerns
Prior 2010	Low	Low	Low	Low	Some concerns	Some concerns
Rapoport 1997	Low	Some concerns	Some concerns	Low	Some concerns	Some concerns
Rapoport 2002	Low	Low	Low	Low	Some concerns	Some concerns
Ryan 2002a	Low	Low	High	Low	Some concerns	High
Ryan 2002b	Low	Low	Some concerns	Low	Some concerns	Some concerns
Ryan 2002c	Low	Low	Some concerns	Low	Some concerns	Some concerns
S2WB3002 1996	Some concerns	Low	Low	Low	Some concerns	Some concerns
Sakai 2002	Some concerns	High	High	Low	Some concerns	High
Sakai 2021	Low	Low	Low	Some concerns	Low	Some concerns
Sandrini 2002	Low	Low	Low	Low	Some concerns	Some concerns
Saper 2006	Low	Low	Low	Low	Some concerns	Some concerns
Sargent 1995	Low	Low	Low	Low	Some concerns	Some concerns
Savani 1999	Some concerns	Low	Low	Low	Some concerns	Some concerns
Sheftell 2003	Low	Low	Some concerns	Low	Some concerns	Some concerns
Sheftell 2005a	Low	Low	Low	Low	Some concerns	Some concerns
Sheftell 2005b	Low	Low	Low	Low	Low	Low
Smith 2005	Some concerns	Low	Low	Low	Some concerns	Some concerns
Solomon 1997	Low	High	Low	Low	Some concerns	High
Spierings 2001	Low	Low	Low	Low	Some concerns	Some concerns
Stark 2002	Low	Low	Low	Low	Some concerns	Some concerns
Steiner 2003	Low	Low	Some concerns	Low	Some concerns	Some concerns
SUM20033 2003	Low	Low	Low	Low	Low	Low
Tazaki 1993a	Low	High	Low	Low	Some concerns	High

Teall 1996	Low	Low	Low	Low	Some concerns	Some concerns
Tfelt-Hansen 1995	Low	Some concerns	Low	Low	Some concerns	Some concerns
Tfelt-Hansen 1998	Low	Low	Low	Low	Some concerns	Some concerns
Tfelt-Hansen 2006	Low	Low	Low	Low	Some concerns	Some concerns
Toledano 2021	Low	Low	Low	Low	Low	Low
Visser 1996a	Low	Low	Low	Low	Some concerns	Some concerns
Visser 1996b	Low	Low	Low	Low	Some concerns	Some concerns
Voss 2016	Low	Low	Low	Low	Low	Low
Wentz 2008	Low	Low	Low	Low	Some concerns	Some concerns
Winner 2003a	Low	Low	Low	Low	Some concerns	Some concerns
Winner 2003b	Low	Low	Low	Low	Some concerns	Some concerns
Yu 2023	Low	Low	Low	Low	Low	Low

Table S8. Risk of bias – Sustained pain freedom from 2 to 24 hours

Author, year	1. Randomization process	2. Deviations from the intended interventions	3. Missing outcome data	4. Measurement of the outcome	5. Selection of the reported result	Overall risk of bias
Ashina 2021	Low	Low	High	Some concerns	Low	High
Barbanti 2012	Low	Low	Low	Low	Low	Low
Brandes 2005	Low	Low	Low	Low	Some concerns	Some concerns
Brandes 2007a	Low	Low	Low	Low	Some concerns	Some concerns
Brandes 2007b	Low	Low	Low	Low	Some concerns	Some concerns
Cady 2006a	Low	Low	Low	Low	Some concerns	Some concerns
Cady 2006b	Low	Low	Low	Low	Some concerns	Some concerns
Cady 2009	Low	Low	Low	Low	Low	Low
Carpay 2004	Low	Low	Low	Low	Low	Low
Croop 2019	Low	Low	Low	Low	Low	Low
Dahlöf 2001	Low	Low	Low	Low	Some concerns	Some concerns
Diener 2011	Low	Low	Low	Low	Low	Low
Dodick 2019	Low	Low	Some concerns	Low	Low	Some concerns
Dowson 2002a	Low	Some concerns	Low	Low	Some concerns	Some concerns
Eletriptan Steering Committee in Japan 2002	Low	Low	High	Low	Some concerns	High
Freitag 2008	Low	Low	Some concerns	Low	Some concerns	Some concerns
Garcia-Ramos 2003	Low	Low	Some concerns	Low	Some concerns	Some concerns
Goadsby 2007	Low	Low	Low	Low	Some concerns	Some concerns
Goadsby 2008	Low	Low	Low	Low	Some concerns	Some concerns
Goadsby 2019	Low	Some concerns	Some concerns	Some concerns	Low	High
Gomez-Mancilla 2014	Low	Low	Low	Low	Low	Low
Gruffyd-Jones 2001	Low	Some concerns	Some concerns	Low	Some concerns	Some concerns
Ho 2008a	Low	Low	Low	Low	Some concerns	Some concerns

Ho 2008b	Low	Low	Low	Low	Some concerns	Some concerns
Jelinski 2006	Low	Low	Low	Low	Some concerns	Some concerns
Kaniecki 2006	Low	Low	Low	Low	Low	Low
Kuca 2018	Low	Low	Some concerns	Some concerns	Low	Some concerns
Landy 2004	Low	Low	Low	Low	Low	Low
Lipton 2010	Low	Low	Low	Low	Some concerns	Some concerns
Lipton 2019a	Low	Low	Some concerns	Low	Some concerns	Some concerns
Lipton 2019b	Low	Low	Some concerns	Low	Low	Some concerns
Lipton 2019c	Low	Low	Low	Low	Low	Low
Lipton 2021	Low	Low	Some concerns	Low	Low	Some concerns
Marcus 2014	Low	Low	Low	Low	Low	Low
Mathew 2007	Low	Low	Low	Low	Some concerns	Some concerns
MOMENTUM 2019	Low	High	High	Low	Low	High
NCT01657370 2012	Low	Low	Low	Low	Some concerns	Some concerns
NCT03235479 2018	Low	Low	Low	Low	Low	Low
Nett 2003	Low	Low	Low	Low	Low	Low
Pascual 2000a	Low	Low	Low	Low	Some concerns	Some concerns
Pascual 2000b	Low	Low	High	Low	Some concerns	High
Sakai 2021	Low	Low	Low	Some concerns	Low	Some concerns
Sandrini 2002	Low	Low	Low	Low	Some concerns	Some concerns
Saper 2006	Low	Low	Low	Low	Some concerns	Some concerns
Sheftell 2003	Low	Low	High	Low	Some concerns	High
Sheftell 2005a	Low	Low	Low	Low	Some concerns	Some concerns
Sheftell 2005b	Low	Low	Low	Low	Low	Low
Smith 2005	Some concerns	Low	Low	Low	Some concerns	Some concerns
Steiner 2003	Low	Low	Some concerns	Low	Some concerns	Some concerns

Tfelt-Hansen 2006	Low	Low	Low	Low	Some concerns	Some concerns
Toledano 2021	Low	Low	Low	Low	Low	Low
Voss 2016	Low	Low	Low	Low	Low	Low
Wentz 2008	Low	Low	Low	Low	Some concerns	Some concerns
Winner 2003a	Low	Low	Low	Low	Some concerns	Some concerns
Winner 2003b	Low	Low	Low	Low	Some concerns	Some concerns
Yu 2023	Low	Low	Low	Low	Low	Low

References

The complete reference list of studies included in the systematic review is available in Appendix 4.

1. Kellstein DE, Lipton RB, Geetha R, Koronkiewicz K, Evans FT, Stewart WF, et al. Evaluation of a novel solubilized formulation of ibuprofen in the treatment of migraine headache: A randomized, double-blind, placebo-controlled, dose-ranging study. Cephalalgia. 2000;20(4):233–43.

2. Misra M, Sharma T, Kalra J, Goel D, Dhasmana DC. Comparative efficacy and tolerability of sumatriptan, ergotamine, naproxen and rizatriptan in moderate to severe acute attack of migraine. JK Sci. 2010;12(4):175–9.

3. Rapoport AM, Ramadan NM, Adelman JU, Mathew NT, Elkind AH, Kudrow DB, et al. Optimizing the dose of zolmitriptan (Zomig,* 311C90) for the acute treatment of migraine: A multicenter, double-blind, placebo-controlled dose range-findings study. Neurology. 1997;49(5):1210–8.

4. Solomon GD, Cady RK, Klapper JA, Earl NL, Saper JR, Ramadan NM. Clinical efficacy and tolerability of 2.5 mg zolmitriptan for the acute treatment of migraine. Neurology. 1997;49(5):1219–25.

5. Diener HC, Tassorelli C, Dodick DW, Silberstein SD, Lipton RB, Ashina M, et al. Guidelines of the International Headache Society for controlled trials of acute treatment of migraine attacks in adults: Fourth edition. Cephalalgia Int J Headache. 2019 May 1;39(6):687–710.

6. Higgins J, Thomas J, Chandler J, Cumpston M, Li T, Page M, et al. Cochrane Handbook for Systematic Reviews of Interventions version 6.3 (updated February 2022) [Internet]. Cochrane; 2022. Available from: Available from www.training.cochrane.org/handbook

7. Olesen J, Steiner TJ. The international classification of headache disorders, 2nd edn (ICDH-II). J Neurol Neurosurg Psychiatry. 2004 Jun 1;75(6):808–11.

8. Headache Classification Committee of the International Headache Society (IHS). Classification and diagnostic criteria for headache disorders, cranial neuralgias and facial pain. Cephalalgia. 1988;8(Suppl. 7):1–96.

9. Headache Classification Committee of the International Headache Society (IHS). The International Classification of Headache Disorders, 2nd edition. Cephalalgia. 2004;24(Suppl. 1):1–160.

10. Headache Classification Committee of the International Headache Society (IHS). The International Classification of Headache Disorders, 3rd edition (beta version). Cephalalgia Int J Headache. 2013;33(9):629–808.

11. Headache Classification Committee of the International Headache Society (IHS). The International Classification of Headache Disorders, 3rd edition. Cephalalgia. 2018 Jan;38(1):1–211.

12. Kelley NE, Tepper DE. Rescue Therapy for Acute Migraine, Part 1: Triptans, Dihydroergotamine, and Magnesium. Headache J Head Face Pain. 2012 Jan;52(1):114–28.

13. Ashina M, Buse DC, Ashina H, Pozo-Rosich P, Peres MFP, Lee MJ, et al. Migraine: integrated approaches to clinical management and emerging treatments. Lancet Lond Engl. 2021 Apr 17;397(10283):1505–18.

14. Tomlinson A, Efthimiou O, Boaden K, New E, Mather S, Salanti G, et al. Side effect profile and comparative tolerability of 21 antidepressants in the acute treatment of major depression in adults: protocol for a network meta-analysis. Evid Based Ment Health. 2019 May;22(2):61–6.

15. Efthimiou O, Debray TPA, van Valkenhoef G, Trelle S, Panayidou K, Moons KGM, et al. GetReal in network meta-analysis: a review of the methodology: reviewNMA. Res Synth Methods. 2016 Sep;7(3):236–63.

16. Efthimiou O, Rücker G, Schwarzer G, Higgins JPT, Egger M, Salanti G. Network meta-analysis of rare events using the Mantel-Haenszel method. Stat Med. 2019 Jul 20;38(16):2992–3012.

17. Peters JL, Sutton AJ, Jones DR, Abrams KR, Rushton L. Contour-enhanced meta-analysis funnel plots help distinguish publication bias from other causes of asymmetry. J Clin Epidemiol. 2008 Oct;61(10):991–6.

18. Chaimani A, Salanti G. Using network meta-analysis to evaluate the existence of small-study effects in a network of interventions. Res Synth Methods. 2012 Jun;3(2):161–76.

19. Chaimani A, Higgins JPT, Mavridis D, Spyridonos P, Salanti G. Graphical Tools for Network Meta-Analysis in STATA. Haibe-Kains B, editor. PLoS ONE. 2013 Oct 3;8(10):e76654.

20. Turner RM, Davey J, Clarke MJ, Thompson SG, Higgins JP. Predicting the extent of heterogeneity in meta-analysis, using empirical data from the Cochrane Database of Systematic Reviews. Int J Epidemiol. 2012 Jun;41(3):818–27.

21. White IR, Barrett JK, Jackson D, Higgins JPT. Consistency and inconsistency in network meta-analysis: model estimation using multivariate meta-regression. Res Synth Methods. 2012 Jun;3(2):111–25.

22. Veroniki AA, Vasiliadis HS, Higgins JP, Salanti G. Evaluation of inconsistency in networks of interventions. Int J Epidemiol. 2013 Feb 1;42(1):332–45.

23. König J, Krahn U, Binder H. Visualizing the flow of evidence in network meta-analysis and characterizing mixed treatment comparisons: J. KöNIG *ET AL.* Stat Med. 2013 Dec 30;32(30):5414–29.

24. Rücker G, Schwarzer G. Ranking treatments in frequentist network meta-analysis works without resampling methods. BMC Med Res Methodol. 2015 Dec;15(1):58.

25. Nikolakopoulou A, Higgins JPT, Papakonstantinou T, Chaimani A, Del Giovane C, Egger M, et al. CINeMA: An approach for assessing confidence in the results of a network meta-analysis. PLOS Med. 2020 Apr 3;17(4):e1003082.

26. Salanti G, Marinho V, Higgins JPT. A case study of multiple-treatments meta-analysis demonstrates that covariates should be considered. J Clin Epidemiol. 2009 Aug;62(8):857–64.

27. Ostinelli EG, Efthimiou O, Naci H, Furukawa TA, Leucht S, Salanti G, et al. Vitruvian plot: a visualisation tool for multiple outcomes in network meta-analysis. Evid Based Ment Health. 2022 Dec;25(e1):e65–70.

28. Seo M, Furukawa TA, Veroniki AA, Pillinger T, Tomlinson A, Salanti G, et al. The Kilim plot: A tool for visualizing network meta-analysis results for multiple outcomes. Res Synth Methods. 2021 Jan;12(1):86–95.

29. Trevena LJ, Zikmund-Fisher BJ, Edwards A, Gaissmaier W, Galesic M, Han PK, et al. Presenting quantitative information about decision outcomes: a risk communication primer for patient decision aid developers. BMC Med Inform Decis Mak. 2013 Nov;13(S2):S7.

30. Leucht S, Siafis S, Engel RR, Schneider-Thoma J, Bighelli I, Cipriani A, et al. How Efficacious Are Antipsychotic Drugs for Schizophrenia? An Interpretation Based on 13 Effect Size Indices. Schizophr Bull. 2022 Jan 21;48(1):27–36.

31. Balduzzi S, Rücker G, Nikolakopoulou A, Papakonstantinou T, Salanti G, Efthimiou O, et al. netmeta: An R Package for Network Meta-Analysis Using Frequentist Methods. J Stat Softw. 2023;106(2):1–40.

32. Sterne JAC, Savović J, Page MJ, Elbers RG, Blencowe NS, Boutron I, et al. RoB 2: a revised tool for assessing risk of bias in randomised trials. BMJ [Internet]. 2019 [cited 2022 May 27];366. Available from: https://pubmed.ncbi.nlm.nih.gov/31462531/

33. Papakonstantinou T, Nikolakopoulou A, Higgins JPT, Egger M, Salanti G. CINeMA: Software for semiautomated assessment of the confidence in the results of network meta-analysis. Campbell Syst Rev. 2020 Mar;16(1):e1080.

34. Chiocchia V, Nikolakopoulou A, Higgins JPT, Page MJ, Papakonstantinou T, Cipriani A, et al. ROB-MEN: a tool to assess risk of bias due to missing evidence in network meta-analysis. BMC Med. 2021 Dec;19(1):304.
